# Mixed Script Identification Using Automated DNN Hyperparameter Optimization

**DOI:** 10.1155/2021/8415333

**Published:** 2021-12-10

**Authors:** Muhammad Yasir, Li Chen, Amna Khatoon, Muhammad Amir Malik, Fazeel Abid

**Affiliations:** ^1^School of Information Science and Technology, Northwest University, Xi'an, Shaanxi, China; ^2^Department of Information Engineering, Chang'an University, Xi'an, Shaanxi, China; ^3^Department of Computer Science, Islamic International University, Islamabad, Pakistan; ^4^Department of Information System, University of Management and Technology, Lahore, Pakistan

## Abstract

Mixed script identification is a hindrance for automated natural language processing systems. Mixing cursive scripts of different languages is a challenge because NLP methods like POS tagging and word sense disambiguation suffer from noisy text. This study tackles the challenge of mixed script identification for mixed-code dataset consisting of Roman Urdu, Hindi, Saraiki, Bengali, and English. The language identification model is trained using word vectorization and RNN variants. Moreover, through experimental investigation, different architectures are optimized for the task associated with Long Short-Term Memory (LSTM), Bidirectional LSTM, Gated Recurrent Unit (GRU), and Bidirectional Gated Recurrent Unit (Bi-GRU). Experimentation achieved the highest accuracy of 90.17 for Bi-GRU, applying learned word class features along with embedding with GloVe. Moreover, this study addresses the issues related to multilingual environments, such as Roman words merged with English characters, generative spellings, and phonetic typing.

## 1. Introduction

Code-mixing is defined as “the embedding of linguistic components such as phrases, words, and lexemes from one language into an expression from another language.” Code-mixing refers to the use of linguistic units' words, phrases, clauses from different languages at a sentence level. One or more languages have been combined to form an intelligible new language. This hybrid language is known as a fused lect. “Code-switching” is considered as unregulated choice by linguists, and is also known as “language mixing,” or as “fused lects” in cases where grammar is rigid.

Where code-switching between two or more languages is prevalent, terms from both languages may become common in sentences. Instead of switching codes at semantically or sociolinguistically significant points, this code-mixing has no particular value in the immediate context. Because they are completely grammaticalized, fused lects allow for less variety than a mixed language because of their semantics and pragmatics. The grammar of the fused lect determines which source-language parts may be included in the fusion. It is observed in an informal setting, like social media commonly. With the abundance of social media platforms available for people to communicate, the quota of code-mixed data available to us is tremendous. The content shared in social media discussions is frequently mixed with stylistic and misspelled versions of original words. POS tagging and named entity identification suffer due to the noisy input. In addition, social media users often utilize mixed scripts of Roman text.

The use of Roman script leads to the generation of informal mixed language amalgamation of two or more languages. This phenomenon is observed on social media specifically. The multilingual users are using the roman script with English characters, which are indifferent from the native language and explain the particular views. For instance, “Rahat is eating angor and playing with Rita.” Here, the word “angor” meant Grapes in English borrowed from Hindi and modified with the English characters. Such a case is complex to handle and considered noisy data in NLP systems [1].

This study tackles the issue of developing text classifiers whose function is to detect multiple mixed scripts independent of both the domain and the language. For successful text classification, a variety of fundamental text transformations, features, weighting parameters, and four deep learning classifiers as classifiers are orchestrated together into a single system. A metaheuristic is used on a search space that contains all possible combinations of different text transformations, features, and parameter weighting procedures with their respective parameters to find a configuration that produces highly effective mixed script identification. This model selection procedure adopted in this study is described as hyperparameter optimization.

Looking at small text fragments from a variety of languages is key to determining the language. The work in this paper, for mixed scripts including both cursive and normal like Hindi-English-Bengali, Roman Urdu, and Saraiki, was performed by using recurrent neural networks (RNN) variants-based approaches used along with the GloVe embedding and word class features. Specifically, we trained 300-dimensional Global vectors and augmented mixed-script sentences with code-mixed data from other sources to increase the robustness of the trained word embeddings. These two techniques for combined feature engineering (CFE) composing GloVe and word-class features are taken to train and test the system. The work on language identification in the code-mixed text using CFE is a novel approach for word-level identification of mixed scripts' situations where text is written in Roman script. The motivation behind work on language identification in the code-mixed text using CFE is a novel approach for word-level identification of mixed scripts situations where text is written in Roman script. Therefore, dataset consisting of five different languages (3 cursive and 2 noncursive) is selected for effective validation of the proposed method

The significance of this study is to explore (1) two kinds of word embeddings; (2) four classifiers (LSTM, Bi-LSTM, GRU, and Bi-GRU); (3) various deep neural network architectures; (4) optimal value of different hyperparameters to find the optimal language detection for the mixed-script dataset consisting of Roman Urdu, English, Saraiki, Hindi, and Bengali languages. In the parameter optimization process, DNN structures and hyperparameter values were automatically tuned.

This paper is organized as follows.  Section 2 is about the related work of mixed script identification. The optimized architecture of recurrent neural networks for mixed script identification is presented in Section 3. In Section 4, the experiment and results are described.  Section 5 includes the discussion. And Section 6 is the conclusion of this paper.

## 2. Related Work

Word embedding and neural networks approaches have yielded impressive results for various code-mixed language classification tasks [2]. Word embedding has been popular in understanding words as points in real-valued multidimensional vector space. It refers to the vector representation of the given data capturing the semantic relation between the words in the data. Popular word embedding features like GloVe are used for representing each word, whereas recurrent neural networks and their variants [3–6] are a natural extension of neural networks for processing sequential (or partially sequential) data, such as natural language. Personal opinions are expressed on social media as a means of communication using a mixed code while writing. Since it is difficult to switch between different keyboard interfaces, this code-mixing is written in the same Romanized script by the majority.

A code-mixing between English and Hindi on Facebook posts was analyzed in the research [7]. They analyzed the difficulty of distinguishing Roman script. Machine learning algorithms have been proposed to solve the problem of tagging words with language identifiers in recent times. Language recognition tools (such as langid.py [8]) solve this problem and use different classification algorithms to solve the problem at the sentence level. Many methods have been utilized in [9–12] to handle the problem of classifying code-mixing by using different frameworks, such as *n*-gram [13], Malayalam-English used Bi-LSTM and Hindi-English used KNN in [14, 15], parts of speech (POS) [16] on multiple languages pairs, hidden Markov model [17], combined Support Vector Machine and CRFs [2] applied on code-mixed languages pairs such as Spanish-English [18], Dutch-Turkish [19], Maltese-English [20], Romanized Arabic Moroccan (Darija), French-English [21], current standard Egyptian-Arabic dialect [22], English-Mandarin [23, 24], and English-Malay [25]. Balazevic et al. in [26] presented the integration of user-specific information to enhance the recognition of Twitter dataset in 16 languages. Furthermore, [27] performed language detection in Hindi-English-Bengali. They used SVM with linear kernels by using word context features, minimum-edit distance-based weights, dictionary-based weights, and *n*-grams with weights. This system was proposed by [28]; code-switching was identified in Spanish English and Nepali English. These features include word length, capital letters, character *n*-gram, contextual information, and dictionary-based features.

Deep learning is based on a neural network such as a recurrent neural network (RNN), and its variants like Long Short-Term Memory (LSTM) and bidirectional LSTM are used for the identification of code-mixed languages presented in [26]. Work on the English-Hindi and English-Spanish code-mixed texts based on tweets using Bi-LSTM for word-characterizing with fast-text. It is classified through conditional random fields (CRFs) presented in [29]. An LSTM (Long-Term Short-Term Memory) neural network with CRF is proposed by [30] for language detection in code-switching conversion characterized by character *n*-gram and morphology. Another experiment used GRU and LSTM on multilingual text identification compared with the machine learning model and achieved better performance using GRU [31]. Therefore, works towards a word-class feature focus on repetition related to the same characters to replace a single character [32]. Another work on [33] deep learning-based architecture for code authorship identification system (DL-CAIS) was proposed by [34] to assist the huge-scale of language-oblivious, for identification by using TF-IDF with RNN. Different code-switched metrics for multilingual language identification were explored by [35]. Embedded features associated with the text investigate the semantic information. Word embedding is used to identify the extent of aggressive text that is included for the extraction and encoding. For this purpose, GloVe vectors are utilized by [36] to extract vector representations of words. Another work, the information retrieval system by [37], focuses on the bilingual code-mixed search queries based on English and Chinese datasets. Utilization of Word2vec By [38] via pretrained embedding model with recurrent neural network for English-Spanish and English-Nepali for code-mixed languages.

Work done by [39] achieved reliable accuracy on many techniques and reported different problems in multilingual language identification in neural networks, such as word order, generative spellings, and phonetic typing. To address these problems, optimized deep learning algorithm through word embedding using GloVe and word-class features was utilized. Different features combinations are then fed into the recurrent neural network architectures to find an optimized classification model. To the best of our knowledge, the recurrent neural network can handle sequential information and long-term dependencies followed by GloVe and word class features accomplishing the desired results efficiently.

## 3. Corpus Collection and Dataset Statistics

The dataset used in this study is a complex dataset with a good mix of cursive (Hindi, Bengali, and Saraiki) and noncursive scripts (English and Roman Urdu) shown in Tables 1 and 2. The dataset is obtained from different social media platforms (Twitter, Facebook, and Whatsapp). Different Facebook posts were collected using Facebook API while a java-based application is created over Twitter API for collection of tweets from Twitter from specified accounts in local languages. For this purpose, Tweepy is used to read Twitter stream after applying appropriate filters on hashtags and regions. For languages like Saraiki and Roman Urdu, the data collection method was random Whatsapp groups and Facebook groups. English-Hindi and English-Bengali were collected in the same way. After the collection of the corpus, the data was tokenized using a manual tokenizer developed in Java. This manual tokenizer is a modified version of the CMU tokenizer [40]. CMU tokenizer was developed for English. However, it is modified for its use for Roman Urdu, Hindi, and Saraiki. After that, the tokens are manually tagged with their respective languages using two different annotators. Clashes of linguistics tags are removed from the dataset, and the final dataset statistics are described in the following.

### 3.1. Combinatorial Framework for Mixed Script Identification

#### 3.1.1. Test Data Cleansing

In this study, text data consisted of unlabeled data gathered from various sources having noisy data to verify the framework efficiency. Therefore, the text is preprocessed in different steps by removing hashtags, HTML tags, diacritics, and other unimportant signs. These are performed for cleansing test data. Further, preprocessing is performed towards primary texts and HTML tags. A separate module is developed based on Natural Language Tool Kit (NLTK) and Sci-Kit Learn functions. Regular expressions are used to clean the mentioned objects and tags throughout parsing given regular expressions and arrange them in a structured form. The preprocessed dataset is then classified into feature and label sets utilization of the SCI-KIT learn. The performance of different algorithms is evaluated on both preprocessed and unprocessed noisy data.

### 3.2. Methodology

**Figure 1:**
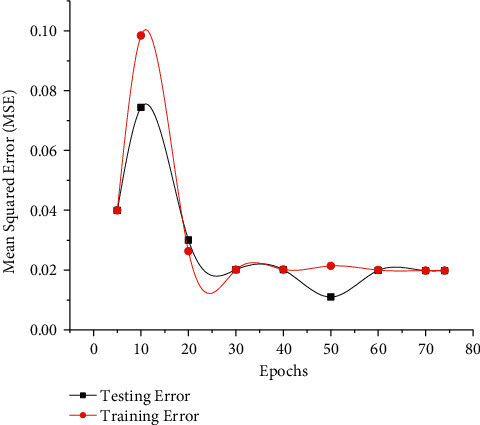
MSE comparison with training epochs.

The nature and structure of the dataset (given in Section 3) allow using supervised machine learning [41] approaches for training and testing of the proposed method. The presented problem is a multilabel text classification problem formulated as follows:

For a given dataset *D* consisting of different instances (*d*_1_, *d*_2_, *d*_3_,…, *d*_*n*_} with different class labels defined over a set *L* = {*l*_1_, *l*_2_, *l*_3_,…, *l*_*n*_}. Each instance *d*_*i*_ is associated with one class label *l*_*i*_. Therefore, relation *D* has one-one relation over set *L* resulting in single-label classification. The objective is to train a deep learning classifier on the dataset *D* to find the accurate label *L* where each *d*_*i*_ correspond to *l*_*i*_ exactly once. The DNN classifiers are implemented of different word embeddings to explore the results and find an efficient technique. The core problem is the correct selection of embedding scheme, vector type, classification algorithm, the architecture of the neural network, and hyperparameter values. Therefore, the experimentation focused on the following:

(i )Word class features and global vectorization (GloVe) for informal test inputs are composed of *n*-gram vectors. It can handle misspelled words and disambiguation problems also.
(ii) LSTM [42], Bi-LSTM [43], GRU [44], and Bi-GRU [45] are investigated as classifiers (with one-dimensional convolution layer). These classifiers can process sequential data to overcome the short memory problem of recurrent neural networks. Thus, vanishing gradient problem of RNN is solved in these classifiers. LSTM and Bi-LSTM follow text processing in the forward stream only and backward stream simultaneously. Therefore, the impact of text at the current moment in text corpus is perceived with accuracy by these two classification algorithms.
(iii) GRU has no cell state like LSTM and therefore seeks influential word sequences (*n*-grams). On the other hand, Bi-GRU consists of two gated recurrent units. One processing is sequential input in a forwarding while the other is in a backward manner.
(iv) Deep neural network architecture is shown in Figure 1, consisting of different parameter values as follows:
  - Count of hidden layers [simple LSTM/Bi-LSTM or stacked LSTM/Bi-LSTM]
  - Count of neurons in network architecture [100–400]
  - Dropout parameter [typically between +0 and +1]
  - Kind of activation function (softmax, ReLU)
  - Different optimizers (ADAM, AdaGrad)
  - Batch size (typically 16, 24, 32, and 64)
  - Epochs count (10–50)
  - Therefore, the available options for adjusting an accurate neural network architecture become enormous. It becomes difficult for an expert to rely on its predetermined knowledge for the selection of parameters. The parameter tuning is performed using the Hperas library [46] automatically. The following two algorithms are iterated 100 times for finding optimized values.
  - Parzen Estimator [47] is used to tune hyperparameters in a treelike manner. The estimator is invoked through the suggested command. Parzen estimator follows the Bayesian modeling approach to decide parameters value iteratively over a predefined distribution.
  - A random estimator (random suggest) is used in tuning the parameters randomly on a given set of hyperparameters.
  - Both algorithms are implemented using Keras [48] and TensorFlow [49] in python.

#### 3.2.1. The Recurrent Neural Network Architecture

**Figure 2:**
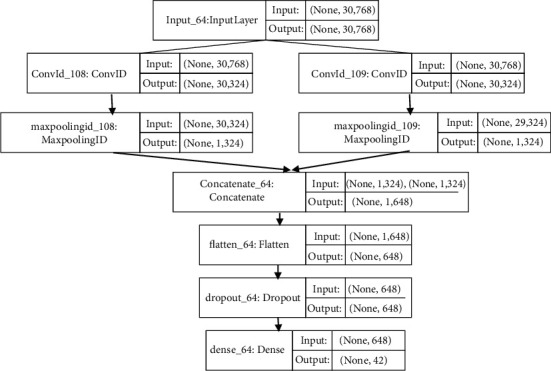
Optimized DNN architecture implemented with GloVe.

This section explains the framework of our proposed RNN architecture for mixed script identification. It includes data preprocessing, word vector representation utilizing GloVe along with word-class features, and recurrent neural network. It is done with its variants LSTM, Bi-LSTM, GRU, and Bi-GRU. (i) Data preprocessing is the first phase, which includes vector representation using GloVe with word class features. (ii) Representation into RNN architecture for classification as shown in Figure 2.

#### 3.2.2. Word Vector Representation with Word-Class Feature

A typical mode to use phrase embedding is utilizing GloVe to train the model and generate the correct embedding for vector representations. These phrases are obtained through GloVe [36], pretrained embedding, including the 2B tweets, 27B tokens, 1.2M vocab, uncased, and 200 dimensions vectors for both code-mixed corpus. A GloVe is a global log bilinear regression model for unsupervised learning of word representations. The model follows the count of global word-word co-occurrences statistically. The number of times a word occurs in a context is counted. For every particular context of interest, the co-occurrence probability is counted globally. A meaningful model is produced with the help of word vector spaces along with their substructures in the result. The GloVe provides better representation among other unsupervised algorithms with better accuracy while tested on word analogy, word similarity, and NER [36] sentiment analysis [50]. Afterward, we normalized the words through the word-class feature [32]. This feature makes sure that the words take a similar structure. It exists in the same class (e.g., words contain AAAAaaaa characters). Subsequently, the word-class feature replaced the repeating characters into a single character as Aa. This feature abled architecture to improve better representation. Further, it can extract meaningful words from the corpus. This capability enhances the performance of the evaluation metric related to the code-mixed language identification task.

#### 3.2.3. Recurrent Neural Network

A recurrent neural network (RNN) is a class of artificial neural networks, where connections among nodes shape a directed graph alongside a temporal collection. It builds in a nonlinear and complicated encoder version that can store a significant amount of information. Different feedforward neural networks, RNNs can use their memories for sequences of inputs. RNN has been used in multiple language processing applications, like question answering [51], speech recognition [52], conversation modeling [53], handwriting recognition [54], language modeling [55], and machine translation [56]. Though RNN is better for sequential information, yet it neglects word order. RNN was also used in different fields, such as mathematics [57, 58]. Further, RNN is affected by the problem of vanishing and exploding gradient [59], which causes slow learning and training of the model. These issues are resolved by considering its variants such as LSTM, which works on gated mechanisms. Through these gates, LSTM can hold long-term dependencies and overcome the problem of training. More variations in standard LSTM such as Bi-LST [60], GRU [5], and Bi-GRU [61] are found to be adequate to address the mentioned issues.

*Long-Short Term Memory*. A Long Short-Term Memory (LSTM) network is a type that is fit for learning request reliance in succession prediction issues among different variants of RNN. LSTM is an RNN network that is trained with the help of the backpropagation algorithm with time and resolves the vanishing gradient problem [59]. LSTM work with memory block connected with layers. Every block has a memory for new sequences and gates that maintains the block state and output. To control block conditions, three gates such as input, forget, and output are applied on the input sequences by working with sigmoid activation.

An LSTM gives input through hidden state/layer and calculates the outcome [3] as in the following expressions:

(1) $$\begin{array}{c} i_{t}=\sigma \left(wv_{xi}x_{t}+wv_{hi}h_{t-1}+wv_{ci}c_{t-1}+b_{i}\right), \end{array}$$

(2) $$\begin{array}{c} f_{t}=\sigma \left(wv_{xf}x_{t}+wv_{hf}h_{t-1}+wv_{cf}c_{t-1}+b_{f}\right), \end{array}$$

(3) $$\begin{array}{c} o_{t}=\sigma \left(wv_{xo}x_{t}+wv_{ho}h_{t-1}+wv_{co}c_{t-1}+b_{o}\right), \end{array}$$

(4) $$\begin{array}{c} c_{t}=f_{t}c_{t-1}+i_{t}\tanh\left(wv_{xc}x_{t}+wv_{hc}h_{t-1}+b_{c}\right), \end{array}$$

(5) $$\begin{array}{c} h_{t}=o_{t}\tanh\left(c_{t}\right), \end{array}$$

where *I*, *f*, and *o* are input, forget and output gate, *c* is a cell of these gates. The sigmoid function is denoted by *σ*, *x*_*t*_ for a given input, and *h*_*t*_ is a hidden state.

*Bidirectional Long-Short Term Memory*. A Bidirectional Long-Short Term Memory (Bi-LSTM) is an advanced class of LSTM. Bi-LSTM also works on the same method of LSTM and works to identify the content of the grouping issue. A Long-Short Term Memory (LSTM) worked in one sequence or forward direction. According to [4, 45, 62], Bi-LSTM can capture or calculate both directions of contexts, such as upcoming and previous hidden layers.

Backward layer:

(6) $$\begin{array}{c} h_{b}=h\left(wv_{xhb}x_{t}+wv_{h_{b}h_{b}}h_{b_{t-1}}+b_{h_{b}}\right). \end{array}$$

Forward layer:

(7) $$\begin{array}{c} h_{f}=h\left(wv_{xh_{f}}x_{t}+wv_{h_{f}h_{f}}h_{f_{t-1}}+b_{h_{f}}\right), \end{array}$$

where *h*_*b*_ is a backward hidden state, *h*_*f*_ for the hidden forward state, and *y*_*t*_ combine the following [63] backward and forward layers into a single layer of Bi-LSTM for the outcome.

(8) $$\begin{array}{c} y_{t}=\left.wv_{h_{f}}h_{f_{t}}+wv_{h_{b}}h_{b_{t}}+b_{y}\right). \end{array}$$

*Gated Recurrent Unit*. A Gated Recurrent Unit (GRU) joints the variant LSTM, input, and forgotten gate into a single update gate, and it is based on the purely LSTM and provides a better model compared with LSTM. A GRU contains two gates: the first gate is for input, and the second gate of GRU is called forget gate [5].

A Gated Recurrent Unit (GRU) also joints the cell state with the hidden state due to other changes.

(9) $$\begin{array}{c} r=\sigma \left(wv_{r}i_{t}+xrhs_{t-1}\right), \end{array}$$

(10) $$\begin{array}{c} u=\sigma \left(wvui_{t}+xrhs_{t-1}\right), \end{array}$$

(11) $$\begin{array}{c} h_{t}=\tanh\left(wvi_{t}+x\left(r⊙hs_{t-1},x_{t}\right)\right), \end{array}$$

(12) $$\begin{array}{c} hs_{t}=\left[\left(1-u\right)hs_{t-1}\right]+uh_{t}. \end{array}$$

Here, *r* is a reset gate and *u* update gate, *σ* is a logistic function, *h*_*t*_ for hidden state, element multiplication denoted by ⊙.

*Bidirectional Gated Recurrent Unit*. A bidirectional GRU is the architecture of long-short term memory, while the Bi-GRU is quicker than LSTM, BI-LSTM, and GRU and has ability to capture the long-term dependencies. Bidirectional GRU increased the ability to hold the sequence of information to both sides of the direction, like upcoming and previous [6].

(13) $$\begin{array}{c} h_{t}=\left({\overset{⟶}{h}}_{t}\|{\overset{⟵}{h}}_{t}\right). \end{array}$$

Here, *h*_*t*_ for output states, ${\overset{\leftarrow }{h}}_{t}$ a backward and ${\overset{⟶}{h}}_{t}$ forward state in the opposite direction.

Moreover, the backpropagation algorithm through time (BPTT) has been utilized in the training of the neural network. In BPTT, errors are dealt with over repetitive connections back majorly through chain rule and error having backpropagation [64]. Backpropagation through time adding, recalling data is easy to call, which resides in hidden layers with infrequent steps. BPTT is also computationally controllable to obtain the gradients at the end [65].

#### 3.2.4. Classification Layer

The last layer is the classification layer having a logistic regression function applied. This function classifies the data based on code-mixed languages. On behalf of the minimization of destructive log probability, stochastic gradient descent is exploited [66]. In our work, the input is captured by tweet as the token as an underlying layer of RNN variants as LSTM, BI-LSTM, GRU, and Bi-GRU as word embedding. It can get the information regarding the current tokens as an initial and the previous upcoming as well. After obtaining the output of the RNN variants, it is sent to the classification layer for language identification in terms of code-mixing.

## 4. Experimentation and Results

### 4.1. Experimental Setup

The feature vectors in our model building are for the analysis of our selected datasets. It is required to test the best parameter in different situations for continuity. Experimentation in this study leads towards the analysis of the embedding dimension, batch size; epoch, learning rate, filter windows, and the dropout rate for these best parameters integration mentioned in Table 3. In permitting and reducing the large dataset into smaller portions in different training scenarios, we can train with the minibatches promptly in a neural network.

### 4.2. Experimentation

All the datasets are divided into two divisions of the training set and testing set for training of the LSTM network. Data were split into 80%–20% ratios for training and testing purposes simultaneously. Because the datasets were obtained from social media sites majorly. Therefore, preprocessing was performed before training purposes. Dimensionality reduction is performed during the preprocessing. In the end, the input and target arrays are generated for training purposes. In a single training example, an array consisted of 192 characters length vectors. The target array consisted of the target language labels. Classifier building, training, and testing were performed with the help of the Keras library. Default LSTM sequential class was modified according to the given models for the addition of different layers. Different classification algorithms are investigated with GloVe embedding with a two-parameter tuning algorithm.

**Figure 3:**
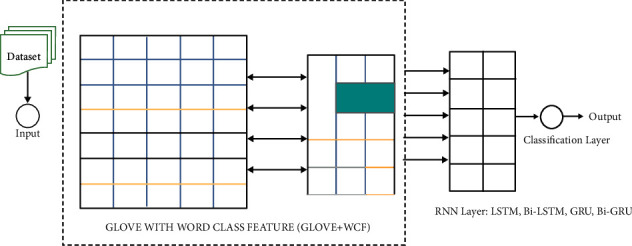
A proposed architecture for code-mixed language identification.

MSE function defines its learning performance and affects the results shown in Figure 3. Error reduction is necessary for the efficiency of the system. Mean square error is calculated as a difference between the desired output and actual output. In this system, the desired output is described in the mathematical model as while the actual output is defined as the difference of both variables, calculated as MSE. Moreover, during the training phase, the training error is also observed to confirm the performance of classification. Mean Squared Error (MSE) is a representation of both training and testing errors. MSE value represents the difference of errors in training and testing subsets for the used dataset.

During the experimentation, the MSE values are observed to go down with the iterations progressing and the final value of MSE is observed to be 0.01985, which is interpreted as a good value for the accuracy of the estimator. In Table 4, the training error rate is mentioned, which is observed to be reduced as the system progress in its learning iterations.

Table 5 describes the results and compares the performance of the LSTM network. Root mean squared error (RMSE) is the measure of regression fitness over the dataset and is calculated as the standard deviation of residuals. normalized root mean squared error (nRMSE) helps in the comparison of models irrespective of units. nRMSE is measured as RMSE concerning the mean of data. Mean absolute error (MAE) indicates the average error between the forecasted and actual estimate. MBE describes the average prediction and helps in finding whether models fit or underfits the data. *R*^2^ is a measure to describe the correlation strength between the actual and predicted results. The value 1 of *R*^2^ represents the strongest relationship, while 0 means no relationship at all. For more improvement of representation of the word, we include predefined word-class features for the correction of undefined word recognition with GloVe. The architecture gets data and performs on dataset word-by-word analysis and feeds the representation into LSTM, Bi-LSTM, GRU, and Bi-GRU. After that, it is sent towards the classification layer for the identification of the languages in which all results are shown in the above tables.

According to the results presented in Tables 5–8, the GloVe implementation with bi-GRU has achieved with highest maximum accuracy with the random estimator. The highest accuracy was achieved (i.e., 0.825 for Saraiki-Roman Urdu mixed scripts). On the other hand, for the Parzen estimator, highest accuracy is achieved by Bi-GRU implemented on top of GloVe for Eng-Bengali scripts. It is observed that for the complex mixed scripts data (i.e., Eng-Hindi-Bengali-Roman Urdu-Saraiki with language count = 5) the accuracy achieved by all algorithms is lower than other scripts (for language count<5). Therefore, it is assumed that with the increase in language count, the accuracy of identification is adversely affected.

**Figure 4:**
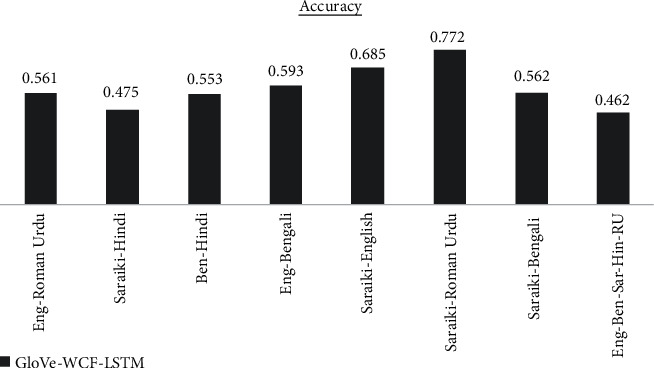
Results comparison for different datasets for GloVe-WCF-LSTM model.

**Figure 5:**
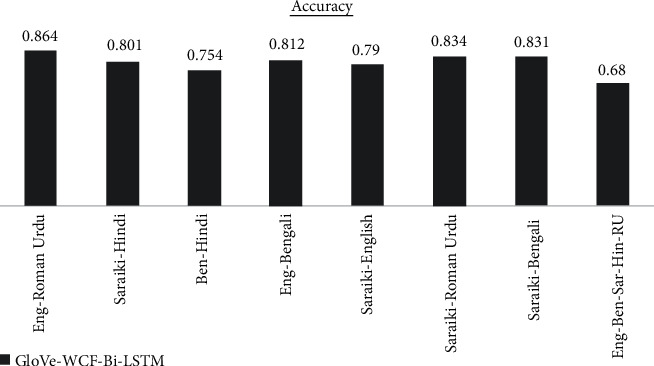
Results comparison for different datasets for GloVe-WCF-Bi-LSTM model.

**Figure 6:**
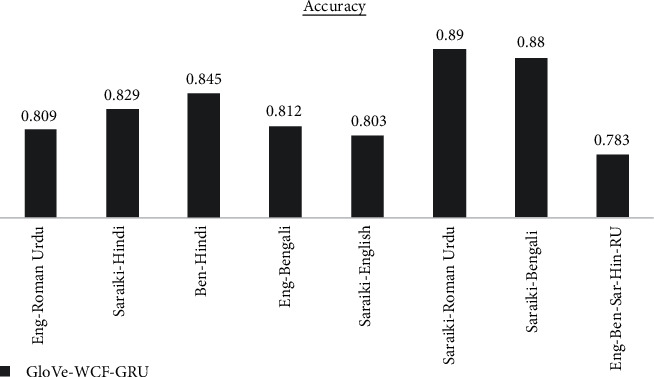
Results comparison of the different datasets for GloVe-WCF-GRU model.

**Figure 7:**
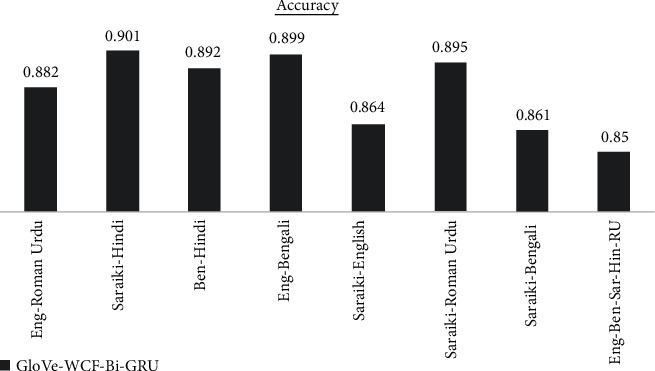
Results comparison of the different datasets for GloVe-WCF-Bi-GRU model.

The results are analyzed for each dataset differently for each of the proposed models. GloVe-WCF-LSTM model maximum accuracy is achieved for Saraiki-Roman Urdu dataset, which is 77.2% in Figure 4 while the model resulted with minimum accuracy (i.e., 46.2 for English-Bengali-Saraiki-Hindi-Roman Urdu mix dataset).  Figure 5 describes results for the GloVe-WCF-Bi-LSTM model, maximum accuracy is achieved for the English Roman-Urdu dataset while the English-Bengali-Saraiki-Hindi-Roman Urdu mix dataset achieved a minimum accuracy of 69%. Results of GloVe-WCF-GRU model Figure 6 describe that maximum accuracy (89%) is achieved for the Saraiki-Roman Urdu dataset while the English-Bengali-Saraiki-Hindi-Roman Urdu mix dataset achieved minimum accuracy (i.e., 78.3%). Maximum average accuracy is achieved by the GloVe-WCF-Bi-GRU model, which is 90.41% for the Saraiki-Hindi dataset, while for the English-Bengali-Saraiki-Hindi-Roman Urdu mix dataset, minimum accuracy is observed (i.e., 85% in Figure 7).

## 5. Discussion

Different models are evaluated on different mixed-script datasets to find an optimized DNN model for the identification of mixed-script textual data. These models are implemented with GloVe and word-class features for their assessment. It is observed that training G1oVe on the mixed-script datasets is best especially when the corpus consists of multiple cursive scripts. By using GloVe vectors and word-class features, the model can search and identify words effectively. The GloVe is trained with word features and features are represented at the sentence level. Then these features are given to RNN variants to hold sequential order of input. Furthermore, we observed that the evaluation metric such as the accuracy of the model is not merely relying on the classifier but also numerous factors like a feature extractor, vanishing gradients, and particularly corpus size since it enhanced the overall model performance.

To the best of our knowledge, we are the first ones who used word-class features with G1oVe to get the benefit of proper identification of words written in multilingual aspects. With this combination of features, the identification of words having wrong spellings is also handled for a dataset consisting of more than two languages.

In this paper, we presented optimized DNN to achieve maximum accuracy of language identification for the mixed-script dataset. The accuracy was calculated by comparing a preannotated test dataset with the resultant output. If the output of the system (i.e., detected language of text token by system) matches the preannotated language label in test dataset, the classification is considered as an accurate classification.

The optimized hyperparameter architecture is presented in Figure 1. The dataset is divided (80% training) and shuffled for training. The optimized model (with the highest achieved accuracy) was experimentally evaluated on the testing dataset. The highest average accuracy is achieved for the GloVe-WCF-Bi-GRU model for which the optimized approach is presented in Figure 7. However, not all the hyperparameters can be presented and plotted. Some important hyperparameters are reported for the model. After the Conv1 layer, the SELU activation function is used. While after the Dense layer, Softmax activation function is used. Optimized batch size is 64 with Nadam optimizer and 0.462 dropout rate.

The overall time complexity of concerning RNN variants activates less noise when LSTM, Bi LSTM, GRU, and Bi-GRU are used to hold and capture the long-range of word order when equipped with learning through GloVe. Further, we contributed word-class features for the effective representation of text. We have another aspect of our model related to the occurrence of the expansive corpus as the word embedding trained on large corpora to perform better [36]. However, in our case, classification performance is enhanced by fine-tuning a subset of data. Usually, in neural networks, the vast corpus of training will enhance the model execution as compared to the domain-specific corpus in our case, fundamentally enhancing the working of word embedding that has substantial impacts on training. On the other hand, training with large corpora demands extra training time, which is a possible barrier to restrict performance.

Moreover, deep learning is considerably affected by the configuration of hyperparameters. The cost for the setting of hyperparameters is expensive when the dataset is large. We optimized hyperparameters using the suggest and random suggest algorithms available in Keras library. Moreover, it is also observed that random suggest has better optimization results when compared with the suggest. The performance results are presented in Table 5, which linearly depicts the evaluation of the proposed architecture. Results in Table 5 show that the proposed architecture can effectively be employed for the task of mixed-script language identification with accuracy.

## 6. Conclusions

The results and comparison with other methods mentioned in Tables 5, 6, 7, and 8 help assess the average accuracy of the optimized DNN method evaluated on mixed script data on multiple models. It is observed that with the increase in the number of languages in the cursive mix script dataset, accuracy decreases. While for the dataset where scripts with different cursive patterns are mixed, more precision is achieved for optimized DNN architecture. For example, a mixed script dataset of Hindi–Saraiki languages achieved more accuracy when compared with Bengali–Hindi dataset enhancement of features. Similarly, Roman–Urdu– Saraiki dataset achieved more accuracy when compared with Saraiki–Bengali and Bengali–Hindi datasets. This phenomenon is probably due to the dissimilarity of cursive patterns. Furthermore, mixed datasets consisting of similar cursive patterns are more difficult to learn for the DNN models. This work is part of ongoing research on multi-lingual script processing. In the future, we aim to use this work in further processing such as multi-lingual transliteration, multi-lingual emotion detection, and customer reviews identification.

## Figures and Tables

**Table 1:** Corpus statistics (Eng = English, Hin = Hindi, Sar = Saraiki, Ben = Bengali, and RU = Roman Urdu).

Dataset	Type	Source FB = Facebook W = Whatsapp T = Twitter	Token count	Total	Sentence
Eng-Roman Urdu	Space oriented	FB + T + W	ENG (102311) + HIN (97235)	199546	3558
Saraiki-Hindi	Cursive	FB + T + W	SAR (78412) + HIN (87563)	165975	4256
Bengali-Hindi	Cursive	FB + T + W	BEN (85672) + HIN (87563)	173235	3801
Eng-Bengali	Mix	FB + T + W	ENG (102311) + BEN (85672)	187983	4065
Saraiki-English	Mix	FB + T + W	SAR (78412) + ENG (102311)	180723	3457
Saraiki-Roman Urdu	Mix	FB + T + W	SAR (78412) + RU (97235)	175647	3962
Saraiki-Bengali	Cursive	FB + T + W	SAR (78412) + BEN (85672)	164084	2864
Eng-Bengali-Saraiki-Hindi-Roman Urdu	Mix	FB + T + W	ENG (102311) + BEN (85672) + SAR (78412) + HIN (87563) + RU (97235)	451193	4539

**Table 2:** Corpus statistics show that (tokens and %age in corpus).

Corpus statistics		
Language	Tokens	%age in corpus
Eng	102311	22.7
Roman Urdu	97235	21.6
Hindi	87563	19.4
Bengali	85672	19.0
Saraiki	78412	17.4
Total tokens in corpus	451193	

**Table 3:** Experimentation setup.

CPU	3.0 GHz (Intel I-10)
Operating system	Windows 10
RAM	16 GB
Tool used	Python 3.6
Training epochs	52

**Table 4:** MSE comparison with training epochs.

Training error	
No. of training iterations	Mean squared error
5	0.03996
10	0.0744
20	0.03004
30	0.02011
40	0.02013
50	0.01989
52	0.01985

**Table 5:** Accuracy comparison with parameter optimization though Parzen estimator.

Glove embedding with Parzen estimator				
Dataset	Classification algorithms			
	LSTM	Bi-LSTM	GRU	Bi-GRU
Eng-Roman Urdu	0.552	**0.843**	0.792	0.851
Saraiki-Hindi	0.456	0.791	0.81	0.823
Bengali-Hindi	0.376	0.754	**0.812**	0.851
Eng-Bengali	0.552	0.758	0.792	**0.889**
Saraiki-English	0.612	0.802	0.797	0.841
Saraiki-Roman Urdu	**0.756**	0.812	0.779	0.862
Saraiki-Bengali	0.521	0.723	0.782	0.834
Eng-Bengali-Saraiki-Hindi-Roman Urdu	0.379	0.669	0.76	0.802

Bold indicates the maximum performance of the algorithm.

**Table 6:** Accuracy comparison with parameter optimization though random estimator.

Glove embedding with random estimator (random suggest)				
Dataset	Classification algorithms			
	LSTM	Bi-LSTM	GRU	Bi-GRU
Eng-Roman Urdu	0.561	**0.864**	0.809	0.882
Saraiki-Hindi	0.475	0.801	0.829	**0.901**
Bengali-Hindi	0.553	0.754	0.845	0.892
Eng-Bengali	0.593	0.812	0.812	0.899
Saraiki-English	0.685	0.79	0.803	0.864
Saraiki-Roman Urdu	**0.772**	0.834	**0.89**	0.895
Saraiki-Bengali	0.562	0.831	0.88	0.861
Eng-Bengali-Saraiki-Hindi-Roman Urdu	0.462	0.68	0.783	0.85

Bold indicates the maximum performance of the algorithm.

**Table 7:** Comparison of accuracy.

Reference	Technique	Dataset							
		Eng-Roman Urdu	Saraiki-Hindi	Ben-Hindi	Eng-Bengali	Saraiki-English	Saraiki-Roman Urdu	Saraiki-Bengali	Eng-Ben-Sar-Hin-RU
This paper	GloVe-WCF-LSTM	0.561	0.475	0.553	0.593	0.685	0.772	0.562	0.462
	GloVe-WCF-Bi-LSTM	0.864	0.801	0.754	0.812	0.79	0.834	0.831	0.68
	GloVe-WCF-GRU	0.809	0.829	0.845	0.812	0.803	0.89	0.88	0.783
	GloVe-WCF-Bi-GRU	0.882	0.901	0.892	0.899	0.864	0.895	0.861	0.85

[36]	GloVe-LSTM	—	—	86.97	88.27	—	—	—	—
	GloVe-Bi-LSTM	—	—	85.49	87.57	—	—	—	—

[57]	Char-LSTM	—	—	57.8	—	—	—	—	—
	Subword-LSTM	—	—	69.7	—	—	—	—	—

**Table 8:** Test results for Bi-GRU model.

Dataset	RMSE	nRMSE (%)	MBE	MAE	*R* ^2^
Eng-Roman Urdu	355.9	7.70	12.5	158	0.89
Saraiki-Hindi	380.7	9.10	3.5	174	0.91
Bengali-Hindi	410.6	6.73	6.4	191	0.87
Eng-Bengali	392.4	4.35	9.76	127	0.87
Saraiki-English	282.8	3.90	5.76	141	0.81
Saraiki-Roman Urdu	365.9	6.34	6.56	136	0.8
Saraiki-Bengali	401.2	6.90	8.54	189	0.86
Eng-Bengali-Saraiki-Hindi-Roman Urdu	673.6	11.50	9.32	174	0.75

## Data Availability

The authors collected Facebook posts from different pages; groups using the IDM Grabber tool while a java-based application is created over Twitter API for the collection of tweets from Twitter from specified accounts in local languages. The Urdu-Eng FB posts were collected from the Respected Student of GCUF Layyah Campus 1, ASR-Eng FB posts collected from The Islamia University of Bahawalpur (Main Campus) Official IUBianz Updates2, for FB posts HIN-Eng3 and BEN4.The WhatsApp messages were also collected for the dataset. The Twitter tweets collected included @MamataOfficial, @imrankhanoffical, @sujoy_g, @rituparnas11, @shahmehmoodqurashioffical, and @virendersehwag, through java-based Twitter API5. They also collected the Roman Urdu-Eng dataset from Kaggle.com6. These links are provided in these statements. All links are given below: (1) https://www.facebook.com/groups/169698994948802; (2) https://www.facebook.com/groups/251190693255235; (3) http://www.facebook.com/Confessions.IITB; (4) https://www.facebook.com/JU-Confessions-1609256459297929/; (5) http://twitter4j.org/; (6) https://www.kaggle.com/smat26/roman-urdu-dataset.

## Supplementary Materials

Supplementary Materials Supplementary materials (e.g., datasets or results outcomes in the form of graphs) from different stages are provided with the manuscript. The graphs including system training, validation, and the testing outcome of all RNN variants are included in Supplementary Materials.
