# Supplementary material for: Mixed Script Identification Using Automated DNN Hyperparameter Optimization
Source: Comput Intell Neurosci. 2021 Dec 10;2021:8415333. doi: 10.1155/2021/8415333 (PMC8683192; doi:10.1155/2021/8415333)
Supplement: Supplementary Materials — (e.g., datasets or results outcomes in the form of graphs) from different stages are provided with the manuscript. The graphs including system training, validation, and the testing outcome of all RNN variants are included in Supplementary Materials. [file 8415333.f1.zip › 8415333.f1/codemixed.pdf]

1 Ye song nahi hi Ye MODI Ji ka mehnat ka rang hi wo ek din pura world me dikhe ga ye to sirf Turkmenistan. hi hai.. Great MODi ji 2 2
3 Love u sir love u soo much urs I'ts beautyful vedio ... 2 2
4 Arae sur jee pahelae hamare bharat ke bachho ka parao.... Firr vedesh may jao yaar 1 1
5 Wah! Jitni sundar geet ke bhao hain utnihi sundar aur sureeli aawaz hai . 2 2
8 Sundar ekdam sahi Gaya Hua gana.chhotisi gudiya ko naman 2 2
9 Wao lata mangekar 2 2
10 Aatma aur mun ki pavitrata ki jhalak hai is beti ki aawaz mei, khush raho beti. 2 2
11 Joy hind joy hindMeri bhatan tuje salamModi ji aapko salam 2 2
12 Kash Modiji Aap Manmohan Singh se phly aa gy hoty....aj 2015 humara Desh kha phuch gya hota 2 2
13 India me student pe atyachaar karra hai result nahi dikhaijara hai aur yaha ye inke aankho me dhul jhokra hai 0 0
14 :o :o :o 1 1
15 Ye song nahi hi Ye MODI Ji ka mehnat ka rang hi wo ek din pura world me dikha denge ye to sirf Turkmenistan. hi hai.. Great MODi ji ....great 2 2
16 modiji duniya k masiha he...kafi ku6 kiya he or aage bhi karna he.... 2 2
17 BaH Guruji Kya style hai apka duniyako hila ke chhore hain. 2 2
18 Awsome...isey kehte hain hindustan ki chaap chodna poore duniya me... 2 2
19 Namaste MAHA MODI ji!! 1 1
20 Modi ji aap ko shat shat naman hai jo aap ne pure universe me apne hindustan ka parcham laharaya...... 2 2
22 V good Modi ji aap lege rahe hum aapke sath he 2 2
23 fantabulous... We proud of you Modi Ji...Hamare desh ki tasvir bhi sudharti nazar aa rahi h, sablogon me badlao dikh raha h... India ko apke jaisa hi PM chahiye..Bharat ki kuch zamini samsyaye bhi h jaha dhyan dene ki zarurat h. ..asha h ki jald hi unme bhi sudhar ayega. 2 2
24 Wah sir g ab desh sahi hatho me gya h apko dekh kar aisa hi lagata h 2 2
26 its not hindi its urdu and bolywood ager kamyab hai tu Urdu ki waja say 1 1
27 Narendra Modi Yeh kya bakchodi pel rahe ho facebook pe, kaam karlo plz! 0 0
28 Kash ye sab bangladesh aur pakistan b gate toh kitna acha hota 1 1
29 wah wah Turkes 1 1
32 masha allah good....yeh hain meri hindi 2 2
33 Wah wah maja aa gya !! Indian Govt. should host an International Hindi song compitition !! Tarrakki bahut ho gayi ab thoda entertainment ho jai, 'Kyo bhayio aur behno' ??? 2 2
35 Mujhe gussa un saale hinduo par aata hai jo nasmjhi me modi ji ka virodh karte hain .. 0 0
37 bharat ko vishwa guru banne se koi nahi rok sakta......vande matram 2 2
38 Sir yeh tho sirf aap hi kar sakte hai. Great sir 2 2
39 Modi jigood preiminister hia 2 2
40 Jai sri ram nama namo 1 1
41 Jan Jan ka viswas kam ho na jai hind 1 1
42 modi ji aage badho pura desh aapke ssath hai. 2 2
43 RAJ KAPOOR KI FILMO KE BAARE ME SUNA THA KI USSR ME PASAND KI JAATI HAI.LAGTA HAI VAH AAJ BHI KAYAM HAY. 2 2
45 we are proud our pm ; ab lg rha h ki hmara desh ek achchi rah pr chal para hai jaha se hm puri world me apna jhanda gaar skte hai 2 2
46 modi ji aap ki soch bahut sahi lgti hai progres n hona hi sabki wajah hai is liye sabse zaroori hai ki desh ka vikas ho 2 2
47 bhrat ko no.1 banne me ab jyada samy nahi lagega,, kyo ki modi ji jaisa pm bade bhagya se milte hai,,jo bharat ki rftar ko bdane me koi kasar nahi chodege,,,,,,,,,jai hind,,,,,, 2 2
48 Baccho k Dilon dimag ko satya k raste pe lane k liya Is geet ko schoolo me vandana k rup me anivarya karna chaheye . 2 2
49 KARTIKEY KUMAR modi hi des ko agÅ bada sakate hai 2 2
50 Hahaha budha bachiyo ko taar raha h 0 0
51 Aisa PM naa hua hai aur naa hee hoga kabhi. . 2 2
52 koi hindu nhi koi muslim nhi hm sab hundustani hai 1 1
54 Modi tujhe jaan se maarna mera kaam hai , main Khalistani huuuuuuu 0 0
55 Modije mera request hai ke ap apna whatsapp no de take mai live pic apko dekha saku 1 1
56 Modiji ap apne desh ka tirnga sari duni me lahraya hum sabko Garv hai ap per maa bharti ka man duniya me bad

haya apne ap hum bhartvasio ke dil me bas gaye ho salute sir apko jai hind 2 2
57 Hum Bihar ka to jeet gaye aur UP ka election jeeta dena.Abhi to hum 12seaton se jeete hai hume aur humen 65se aton se jeeta dena. 1 1
58 Pm ho to aisa modi jaisa ji hind jay varat 2 2
59 manka biswas kam jor hona... 0 0
60 Modi is tarah so dekhne se kuchh nahi hota 0 0
61 Modi ji hi aise ekmatra person hai jinme desh ko age Le Jane ki sakti aur jajba hai 2 2
62 Mahan bharat ka mahan beta har har modhi 2 2
65 modi ji aage chalo haem aapki jarurat hai 2 2
66 jai ho bharat mata ki w hamari sanskriti ki jo videshi bhi apnate hai 2 2
67 modi sir app pahale OBC president ho. OBC ke liye kuch karo. bad me koi kuch nahi karega 0 0
68 Sab ka malik ek. 1 1
70 Modi Sab Pranam App bahut Achya kam kar rahe hai is kam me ham sab apke sath hai or meri apse vinti hai ki a pp apni tabiyat ka khayal rakiye app or app jivo hajharo sal vande matram bharat mata ki jai 2 2
74 Modi ji kale kuto Ka ilaj ker do bhut bimar hai 2 2
75 Sir app ke jo soch he subko sath me leke chalna ek din ye soch appko itna upper lejayega chahe koye appko bhul ne ki kosis kerenge to unnke samne appko bhulna nehi balki desh ko bhulna nazar aayega. 2 2
76 Jai hind modi sir 2 2
77 Kiu app apne desh ki paisa ko kharab kar rahe ho..apne desh to ghum ke dekha nehi..aap chale dushre ki desh ghu mne..koi vala kam aapse nehi hoga modi g.. 0 0
78 bahut ho gaya ghumna phirna. ab paani sar se upar jaa raha hai. 0 0
81 jai hind mera bharat mera hindusthan 2 2
82 World best pm narendar modi Ji mira bhart mahan 2 2
83 Lageraho modiji pura des aapke sath he. 2 2
85 Sir ji you r great,Ap sirf bidesh Bhraman korenge ya Desh Ki bachhe ko v khayal rakhenge,Kitna garub hai hum l og,Diya koro sahab 2 2
89 PM ka samman..........mera samman....JAY HIND 2 2
90 JAY JAY HO BHARAT 2 2
92 Kya baat hai g 1 1
93 nice pic modi ji 2 2
94 jaldi aa kar vyapam par kuch kijiye nahi 45 ke paar hattyayen pahuchne wali hai 0 0
95 Salute you sir app bahut mehnat karte hai 2 2
96 Congrats modi jee 2 2
97 Modi ji aap apna kaam kijiye, kisi ki baat ko mat suniye agar 5 baccho me se do bigad jaayen to bhi paalna to pad ta hi hai 2 2
98 Sir hm sab digitial india ke platform pe chalna chahte hai.mai samghta ho kifirst priority hmre force ko hny chahi ye becaymuse unhe sabse jyada digital hona must hai india ke liye. 1 1
101 Wah modi sab 2 2
104 grt step 4grt relation...nic job sir 2 2
105 Kuch nahi karoge tum india ke liye 0 0
106 Addd mee frnds 1 1
108 Jab tak suraj chaand rahega modi ji ka naam rahega 2 2
110 Unstoppable modi sir 2 2
111 Jada ghumte mat raho hamlok ko madat karo hamar bare socho jara ham madat magte to mili nahi qq 0 0
112 Modiji apne desh ka tho khayal rakho sirf gumne ke pm bane ho kiya falthi pm hai 0 0
113 Modi ji ek aisa star hai wo jaroor India ka star badlega kya khete ho mera bhayio or bheno 2 2
114 Modi ji namashkarSorry to say but aap jab v kahin Facebook pe kuch share karte hain to status ko English me u pdate karte hai...Khusi hogi jab aaapka updates hindi me 1 1
115 Jai hind ks.chouhan bhinmal jalore 1 1
116 Duniya ki technology lane par hi dess ka vikas hoga 2 2
118 Prnam ji kuch desh ke liye mai bhi krna chahta hu bas aapka thoda sath chahiye agar mil jaye to mera kaam aasa n or khubshurat hmara desh hoga. 1 1
119 Faltu time nahi hai 0 0
121 Sir aap to India KO bhul gye or gov k paiso PR holiday PR ghum rhe ho aap India me toh dikhai hi nai dete 0 0
122 Hello modi ji 1 1

123 app desh ka dadkhan jaihind 2 2
124 Bhakt se bada anuyai hota hai. 1 1
125 Modi ji hamara hindustan ka naam saada ucha rakhna.jai bharat mata jai hind.. 2 2
129 Namsthe sir jai gurudhev 1 1
130 j banda kam kar raha hai ham maze le rahe hai jab sey india ke pm bane hai tab sey laga gai great man in the wo rld ....best pm in the world..... only one on one...... 2 2
131 Modi Ji. App mearaku thoda call krdejia. 0096555218960 1 1
133 Is desh ko jarurat h guidelines ki Varna itani jayada aabadi ka desh world no.1 nahi ho sakta.jo vikas ke raste ma i aata hai. Usko hamesha ke liye khatam kar do. Chahe wo koi party ho ya galat insan desh ko tarakki chahiye kisi b hi keemat par. 1 1
134 Lage raho sir. 2 2
135 Sir, Namaskar. Mai Bihar se hun aur aapka jabardash prasansak hu, Aap jo desh ke liye niswarth bav se kam kar rahe hai, aapko sadaib best p.m ke roop me jana jaega. salute to you sir. 2 2
138 Humari sabhayata humari pehchaan... 1 1
139 Maza to tab hai jab kohinoor wapis lao 1 1
140 ache log ko ache gifts hi milte hain jaise indians ko aap mila sir ji. 2 2
143 Respected sir aap aaj pure Bharat ki ummid ho ...ek naya savera aapne hi janta ko dikhaya he...ap dhup bhi nikal iye... 2 2
144 Onli modi sarkar 1 1
145 Modi ji apne ye to kaha diya ki Jo saksham hai vo subsiti na le Main sahamat hoo per please Ek bar himmat juta ker bol dijiye Jo saksham hai vo arakshan na le 2 2
146 jai ho modi jii 2 2
149 Sir aap se koch baat krna he zarori 1 1
150 sir aap kya cheez ho? kamal!!!! 2 2
152 desh gourav badha rahe ho ayushman bhav 2 2
153 sir agar aapke pas thoda sa time ho toh kripa karke es desh ki nojawaano ke liye bi kuch kariye.. jyada jankari ke liye kripyaa msg kariye mujhe 1 1
155 Great PM sahab yeh sab only hmare modi ji hi kar skte h 2 2
156 Apna desh chlane ke liye bola apni myiya chuda rhe ho ghum ghum ke.. 0 0
157 UP me kuchh kro nhe to 2017 meBSP aa jayeg 0 0
158 modi saab ye konsi country hai??? 1 1
159 Jai hind maniyo pradhan manteri ji me sena bharti nhi hua lekin me desh ki sena ko salam karta hunJAI HIND J AI MATA KI 2 2
160 Modiji aapko hajaro salam.bahut pm aaya gaya karod lekhe gaya but aaptho imndar pm best of luck 2 2
161 Namastey sir, Jis tarah ap desh ki halat sudhar rhe h. Usi tarah please hum jese peoples pr bi dhyan dijiye. 1 1
162 Modi Ji Kay sare kam acche hay par ek kaam bura hay inko aapan ne jitaya election hay ya World Tour package Purana PM to silent mode or naye PM flight mode hamare desh ki to kismat hi kharab hay 0 0
163 Modhiji se India ki janta ko jo umide thi vo abhi tak jaha ki taha hai kuch esa strong kare jo anewale samay me bhi bana rahe kiyki samay ab kam he 1 1
165 Har har modi ghar ghar modi modi ji aap cha gye congresiyo ke pet me dard ho raha hai apki yattra dekh ke 2 2
167 ghum le puri Dunia chutia bana raha pure india ko kutta sala 0 0
168 Modiji des ne aap pr bharosa jtaya hai aap hmari ummido pr khra utrna 2 2
169 Pradhanmantriji Me sapath leta hu ki jis din humare desh ke sare neta subsidy khana band kar dege us din me bh i apne hisse ki subsidy return kar duga. 1 1
170 India ke sabse lokpriy pm modi ji mai aapse request kartahu pm banne ke baad aap mp ke satna nahi aaye please aap jab bhi mp aaye satna aagman ko acsept kar lijiye. Thanku 1 1
173 Modiji nice parson abi survat hai age age dekho hota hai kay 2 2
174 Modi ji aap badhey chalo janta apke sath hai. 2 2
175 Hindustan ka sher p.m modi jo har country ko apne desh khobiyo se intro karwana chahata haijay hind 2 2
178 Gandhi ji aur aapne apni chhavi se na kewal desh me balki pure wiswa me bharat ka nam gauranwit kiya hai wis h u all the best 2 2
180 bharat ka shan narendar modi lakin bihar ka kya hoga modi jee 1 1
181 urgent wrk hai aapke saath modi g 1 1
183 Bharat mata ki jai 2 2
184 Ghandhi gandhi gandhi aakhir ye gandhi hi kya ek logo ko bewkuf bana kr des ko bechne wala 0 0

185 Jai hind jai bharat mata ki jai 2 2
187 Poojjya Narendra modiji good evning mera ek swpna hi aapkasaat ek selpy pooto chaiea anugrha deejia ples 1 1
188 Modi jee, aap jaha jaatey ho vaha sabko apna bna latey ho, good wishes for you 2 2
190 Jiao hind jai bharat 2 2
192 Modi ji desh ka nam bda rhe he. 2 2
193 maharani laxmi bai, tatya tope , Subhas chandra bos , ajad , Bhagat etc are the true leaders 2 2
194 Gandhiji ka sanman itnato congress ne bhi nahi kiya 2 2
195 desh ko ap ki jarurat hai aage le jane ke liye 2 2
196 (gandhi)he iz a murderer. he killed subhas ch. bose 0 0
197 Don't forget godse 1 1
198 ek aayas admi tha nehru 1 1
199 india kbhi bhi china ko peeeche nhi kr paaega..... 0 0
200 P.M.NAMO NAMO Aaj aapki vajha se saare Duniya Ghunj uthe hai only MODI MODI Thanks 2 2
202 Modi ser desh me kharab ho rha anaaaj ???? ???? iske bare me kya soch rhe hy aap 1 1
203 hindustan ke dusre mhatma gandhi nerndr modi jee hai har har modi ghar ghar modi 2 2
204 Jai hind.mera desh mhan 2 2
205 Ek naya itihas banayega Modi ji ka karyekaal. 2 2
206 aaj ke mahatma gandhi P.M.Modi jee modi jee me swami vivekanand ki chhavi bhi dikhtee hai 2 2
209 Kabhi SUBHAS CH Bose & Bhagat Singh ki bhi baat kiya karo sirji....Jisne desh k tukde kar diye us aadmi (Gandhi) ki murti anvaran karte phir raheho @ narendra modiji 0 0
210 maine kai bar suna ki bharat ek din bishwa guru banega. lekin yakeen nahin hota tha. per aap kee lagan aur karya shaily se ab poora yakeen ho gaya,bahut jaldi bharat maha guru ban jayega. bharat maa ki jai. 2 2
211 Mast lag rhe ho modi sab 2 2
212 apne desh ki mahanta ko jitna bhi desh me charcha kiya jaya bhahut hi acha hai 2 2
213 modiji aap itna paysa ghumne me lagarahe ho desh girvi rakhana hai kya 0 0
214 kal modiji nagpur jar nam len sir he vinti aahe sir 1 1
215 Mei meri puri,,, family modi sir se bahoot,,, peyar karte hai 2 2
216 Vande mataram. ..Jai hind. .Jai bharath 2 2
217 modi ji bangladash sa sab hendu ko india ma la chaleya plz eha musalman hendu ka vume dakal karlata ha or ve bahot aneti karta ha 1 1
218 Modiji to apne desh ko aage le jane ke liye bhut kuch kr rhe hai ab desh ke youngest ko jag ne ki bari hai ..... jay hind 2 2
220 Modi sir welcome aapko or hum aap jaise sir ki jarurat 2 2
224 Sir ji aap se bat karna hai hame 1 1
226 Bharat Nav nirmaan karenge MODI Ji,shaktishaali rastra banaaven MODI Ji 2 2
227 Jai hind modi ji 2 2
229 Udar hamare indian faoj ko pakistan goliyo se bhuj raha hai or navaj se hath mila rahe hai 0 0
230 bas isi tarah aap age badate rahiye hum aapke sath hai ... 2 2
231 Modi ji sorry bidesoon main mat ghoomo apne naitaoo saansdon ko pakdo belagaam hote ja tahe hain gareeboun ki socho 1 2 Rs ki policiyon se kuch nahi hoga moti aasamiyoun se paisa nikalo gareboun se nahi 0 0
232 Good deed modi bhai ji......hme hmara desh fir se golden sparrow bnana h....wo spna sch hoga...jiske liye god n apko select kiya h...thnku god...4 modi ji 2 2
234 modi ji. Assam and megalaya me ak khel hota he jiska nam he ter plz usha bond kijea. 1 1
235 May pm supar dupar nmo nmo 2 2
236 modi ji jay ram ji ki 2 2
237 Lega rhia purei takt k sath sbhi bharrt venseyo ki duaey ap k sath hai 2 2
238 modi ji pakishtan ko muhtod jabab dena hoga 2 2
240 Koi fayda hai inko ye sab dikhane se???? 0 0
241 Kya pm bihar me aana. chahte hai 1 1
242 Sir ji kamal kardi apne vides me bhi. 2 2
243 Chankya ne kha hai .. Agar aapko ek kadam aage badhna hai to pahle 10 kadam piche rakho 1 1
246 PM ji ne toh poore vishwa mein ye saabit kar diya hai ki India sab ke sath badhega. 2 2
247 Modi ji grat 2 2
249 Modi sir yeh kaunsi jagah chale gaye ho, mere se toh naam bhi sahi se nai liya ja raha...lol :-D 1 1
250 Modi sirf naam he kahi hei 2 2

252 Itni energy vaala perhaps he koi p.m.raha ho india ka, aapkey liyye bhagvaan se prey kertey hai, sabka saath... S abka vikas....jai ho 2 2
253 Modi mission : 2 establish world brotherhood...grt man grt thinking @ Modi : a boon 2 earth 2 2
254 Modi ji aap aage bado hum tumhare shath he.. 2 2
256 Modi g jaha bhi gaya apna jhanda gaar kar aya. 2 2
257 Modi ji bharat ki badhti abaadi ki fikar kijiye Jo bharat ko patan ke maarg per lejaa rahi h 0 0
258 Hamare desh ko aage badhane vale, logo me utsah jagane, narendra modi ji ko bhagvan hamesha khush rakhe. 2 2
259 Apka kya khna PM ji 1 1
261 I love u.modi sir jankibhi jarurat ho to mang lena haste haste.dedege aap ka subh sintak 2 2
262 Yehi jadu hai modiji ka jaha jate log pyaar karte hai 2 2
263 Modi ji desh ke kanun me kuch Sridhar karyein 2 2
264 Lov u modi ji 2 2
267 Yes yes yes 2 2
269 Me bhe aap ka bahut bada dewana hu 2 2
270 modi ji apko sahi dimag ko me salute karta hu 2 2
271 Mr. Pm hme aap par graw hai. Manish kumar, nasriganj 2 2
275 Lambe samay ke baad desh ko ek samarth n samarthyavan neta mila hai jo videsho me bhi lokpriya hai. 2 2
277 india ka log ka vote ka valu delhi me samach ayyena.india me avo modiji,idhar bhahuth problams he solve karn e ka 0 0
281 akele modi kya ker sakte hai.........akela aadmi pahad nhi khod sakta 1 1
282 Joy hind, Varot mata ki joy 2 2
283 modiji aap great ho but dekhna kahin bahr rishte ache karte karte aapke 5 saal na nikal jayen ..:P 2 2
285 Aap great ho modi ji aapki wajah se hamara sir fakar se ucha ho gaya dunia me jay ho 2 2
286 sicha vibhag ki tarah koi dhyan q n deta jo most important hai log apne baccho ko sarkari college ki jagah privat e collego me q pdhate hai 0 0
287 agr is desh me badlaw lane hai to sampark kre 9451551081, pe mere pass sujhaw aur tarika dono hai teachero ka vetan bhar bdhate jao jo sote hai collego me soche fir comment kre 1 1
288 ghumna band karo modiji ab thoda sarkar ko bhi dekhlo 0 0
290 Great prime minister modi ji jai hind 2 2
291 PM MODI JI BAHUT UDARWADI VIKAS PURUS HAIN 2 2
293 Modi ji aapnea teo kamaal kar diya ji aap ka kaam deakhatea hi muchha majaa aa jata hai ,tv. Per dekhea nhi ki mud fresh ,congratulations ji 2 2
294 Mananiy & sammananiy narendra modi ji is yug k Aap Lauh Purush hai mujhe aap par garv hai Aapko koti-koti Naman 2 2
295 modi ji hame app per garv h lakin desh ka vikas tab hoga jab sabi bhartiyo ki soch Appke jaisi hogi 2 2
296 AAJ mere desh ko anteraatma SE jaga hua honable PM mila 2 2
298 Modi ji aap jesa koi nhi dhanywad aap desh ki kitni sewa karte hai jese ak beta apni ma ko poojta hai 2 2
300 Narendra damodar modi g acche din kab ayenge babu. Hai koi upaye. 0 0
301 ppp model chirman in rajesthan is lalit modi 1 1
303 Tajikistan ne Gurudev Tagore,Mahatma Gandhi ki peetho ki sthapanaa karake Bharat k bhaavaatmak ekataa ko sudridh kiya h 2 2
304 Hamre vote nahi milenge aapko agar us din muslim ki cap pehni to 0 0
305 mahan bibhutiyo ko naman karna sacchi dhridhata ka sabse anmol marg hai .aapko sadar naman 2 2
306 Namaskar app ki vikash bahut achhi gurdev sab se achhi 2 2
307 Aap Hindutva kabhi na bhuliyega 2 2
308 hindustan ka raja he bhai 2 2
310 good soch pm narendra modi 2 2
311 Plz sir aap se baat kar ne hai 1 1
312 Abki baar phir modi sarkar 2 2
315 oh great,,..har din apko dekh ke nai urja milta hai.ap jio hajoro sal sir ji or india ko biswa bijai banao.. 2 2
316 Tusi bast pm ho modi ji Sade I'm panjabi 2 2
317 sir apne rastriya rojgar youjana me sms job ke liye news paper me add dya he kya??? 1 1
318 mene ap ke paas mail kiya hai mughhhhhe aap se answer chahiye 1 1
319 modi ke maki chooooooooth me mera boooooooooooolaaaaaaaaaaaaaaa 0 0

321 Bharat mata ke ji 1 1
325 Ise share karo plz 1 1
326 BJP VIJAY hoooooooooooo 2 2
327 Tu bhi jhootha nikla ,Vaada tera vaada hi nikla... 0 0
329 apna pm modi g ka jalwa... 2 2
330 PM Saab aapkey swatch BHARAT mission ke Himachal Ki Raajdhaani sey ek tasweer bhejee hai mainey kal. U sey jarur dekh Lena 1 1
332 priya narandra modi pm ji apkaisa ho good night 1 1
338 pm uncle abki bar naya bihar bihar ki janta banayegi bjp sarkaar 2 2
339 Devlope sirf sahro me hota hai gawo me nahi. 0 0
341 Mee atalji ko bahat pasand kiya tha. Unka adarsh great tha. Uske bad app ayee sir i salute you 2 2
343 Kabhi ghar me bhi raha karo modi ji? 0 0
344 PM MODI JI KI JAI HO 2 2
348 kyonki aapke baare main jaanta hu to aap bhi mere baare me saari jaankari jaane 1 1
349 Bahut sundar. Hardik subhkamnaye 2 2
350 modiji....humane apko wot diya lekin muze ab lag raha he ki me rong tha 0 0
357 PRANAM,JAI HO 1 1
358 GOOD MORNING TO ALL OF UP M SHRI NARENDRA BHAI MODI JI JAY SHRI KRISHNATHANKS T O BOTH OF USANGITA PATIL MLAVIJAY KUMAR Vicky Mavan SoniJAY SHRI KRISHNA 1 1
361 Modi ji ek aise shashank hai jo wacky ki hindustan ko hindustan he bana rahi hai ab jaake hindustan taraqqi kar raha hai I love modi sarkar 2 2
362 modi sir ko namo namo.humko garb hotahe kiuki humare matrubhumi ne yesa birpurus ko janam diyahe. 2 2
363 apka koi v yojna-Plan sahi Trah se upyog nahi ho paya h..... 0 0
367 mai aap se milta chahta hu lekin mai mil nahi pawga 1 1
369 modi ji kese ho ap.. videsh me apka dheyan rakha ja raha hai na sir.. sir ap please jaldi se ajao fir maan ki baat k arna I'm waiting for it sir.. take care sir 1 1
370 Narendra modi ji plz court me jo cases pending hai use jldi nyai mile aisi koi upai yojna kre 0 0
371 Election jantak chunne k hota h ya neta juth bolte h bade2 wade karte h,lakin wahi'ENGRAGI SASAN'Ise badle ga kon jise humne chunna h lakin wo badlega to wo satta sukh,bade2 ghotale,janta ka pesa janta k leya kyo pryoge n ahi ja raha, 0 0
372 Bhai ghumta rahio kaaam koi na kario 0 0
373 Or kuch ho ja na ho par j bnda sarkari paise par duniya jarur ghum lega. 0 0
374 Jb chunane ka aadhikr janta ko h to,jb unhe lage ke galat aadmi chuna gaya h to,5yr kyo sahe uske tanasahi,rejec t kre usa,isse neta janta k pirti jyda jimmadare honge. 1 1
375 Sunil ji Brahmand ke Sher hain hamarein Modi Ji 2 2
376 India ka hal aisa hote jaiega to india may koi educated person nehi nicklegi 0 0
380 plz modi ji aapke desh basioko samjhaiya ki buddha ka janam nepalmain huwa hain na ki india me. 1 1
381 modi sir....you come bihar.because neccessary to bjp government in bihar.......har har modi ghar ghar modi..... 1 1
384 Kewal ghumte raho aur india ka ko narak me badal do tumhara modi yahi kahani 0 0
385 Modi ne kuch kiya ki nahi... wo pata nhi.. pr wo 5 sal me pura world jrur ghum kr aayega 0 0
386 background music plz..Phul mangu na bahar mangu Mai to sanam tera pyar mangu ? 1 1
387 Modi ji aap dash kahenhe sbheka diloke P.M.ho 2 2
388 Jabtak surj chand rhega tabtak modi ji sarkar rhega ....jay hind 2 2
390 Har har modi ghar ghar modi all world modi ji gud work sir ji 2 2
391 kitni khoobsurat ye tasveer h 2 2
392 modi ji roj hajaro gaaye kat rhi he uanka kuc kro jo bi suvar gaayo ko katata he un ko fasi do ya haat per kat kr c orahe pr fek do salo ko 0 0
393 Bade dhayan se dekh rahe ho 1 1
394 Jitni daulat ye neta log vides ghumne me kharch kar dete agar wo garibo me bat de to kitno ka pet bhar jayega 1 1
395 sir gay mat aki hatyaye roko 2 2
396 waqt sahi, nirnay sahi, sachi mein sahi waqt par sahi nirnay lenevala hamare saath hai tho, hamare bharath ko ou r bharat vaasiyon ko our kya chahiye sir ji. hum sab aapke saath hai sir ji. 2 2
397 Kyo pade ho chakar me koi nahi hai takar me. Modi is best pm . 2 2

398 Bapu g nirdhos h auko jaldi bahar lao 2 2
399 abhi dobara vote ho jaay bjp 7 seat bhi nahi mlaygi jai rajesthan 0 0
400 Pm ky sath videsh mantri bhi h modi 1 1
402 SRIJI Jantar mantra padhare !aapko nyota hai ! 1 1
404 ati uttam modi ji 2 2
410 Modi ji apne kaha tha ki assam se sab bangladesi jald hi jayenge. Par apne to unko dawat pe bulawa diya . Jo na ukile kata tar se assam ata he uske liye to apne volvo bus bhej diye. Apse hamko ye ummid nehi thi. 0 0
411 Apke niti se ane wale salo me bharat sabse age hoga... Or apki widesh niti bahot hi badhiya h... 2 2
412 Enjoy krlo humare paise se ...world tour waw. 0 0
413 modiji yehi voh desh hai jo hume aur mazbut banayenge 2 2
415 Nari samman Desh ka samman,Nariki unnati Desh ki unnat 2 2
416 Pehli bar desh ki "AAN BAN SHAN"dikhth he , jispe hame "NAZ" he.salam to p.m. 2 2
417 Welcome modi jee 1 1
418 Dhyaan se sir bike se mt gir jaana hahaha 0 0
420 great modi ji 2 2
422 please sir apke rhte vito power dila dena 2 2
423 kise kaam.ki.nh hai jo .. 0 0
427 Sir aap aage chalo hum aapke saat he 2 2
429 Sir,kuch apne desh me bhi ghoom liya jaye, kahin bijli to kahin pani,aur kahin road hi nahi haiAchhe din jab aay enge aate rahenge,aap to aao 0 0
431 Bharat desh ke liye avatar hai pm modi ji 2 2
433 Mullo ko bhagao...TP mat karo 0 0
434 Jo aap ke sat rat din lga rha 1 1
435 Sir reconnection ke liye fir se jaana pad sakta hai kya ? 1 1
436 Kailash vijaywargiya ko nikaal bahar karenge aap ya apni saari mehnat or haamare bharoshe pe paani phirwaiye ga 0 0
437 india ka mal jitna paroge ghum lo 5 sal ke bad aap nehin aaoge 0 0
439 modiG u r great pm.lakin un sainiko ka bhi koi solutation h jo jantr mantr pat 29 din se bhukh hardtal oar baitha h.aur hamara media is news ko dikha bhi nhi raha h.WaH india ka media wah 2 2
441 Saare jahan se achha hindustaa hamara 2 2
442 Jo gau hatya band ni kar sakta woh hamare kam ka nehi 0 0
447 Thank you sir apka yatra se desh ko jarur fayada hoga hame garv hai app par aur apaki nitiyo par 2 2
448 Great sir" jabtak suraz chand rahega modiji ka naam rahega.... 2 2
450 Modi ji zindabaad hamare desh ka strong hain joh India ki image badlega 2 2
451 Tan aur Mann desh Kay liye pranam modi ji . 2 2
452 Great modi ji welcomes 2 2
453 Aaj har nagarik ke man main ek hi chahat hai ki narendra modi kuch karenge.aaj haar yuba 2 2
455 Sat sat naman 1 1
456 Welcome back Mr pm.ab kitne din rahenge India me???ye paisa kisika baap ka nehi hai.hamara hai.hamare pais on Se bidesh ja kar ghumte ho saram nehi aati??? 0 0
458 Swagatam Maa bharti apne pass bula rahi h 1 1
459 sar in pakistanio ka kuch kijiye...... kuch pakistani log daily aapka naam lekar india walo ko gaaliya dete h 1 1
461 P m ji roj yhi khre milte ho 0 0
462 Sehar aa gaya 1 1
463 sar aap se jaruri baat kar na he sar mera no 9747375375 1 1
464 Punjab me disst... ferozpur 1 1
465 One rank one pension ke liye Pease nahi hai sharm karo bhul gaye Rewadi ki pehli relli ko PM SAAB 0 0
466 Sir aap jo kam country ke lea kar rahe hai uske lie aapko badhi 2 2
467 P.m modi jee.aj mgnrega sab se top par hai.garamin chhetro mai esse playan ruka hai.lekin esme gram rojgar se wk, j.e. in sabka vawisy aap ke hath mai hai. Hum apne priwar ka laln paln dhang se nhi kr skte.bacho ko achhi shik sha bhi nhi de skte. 0 0
468 Jai hind shar 2 2
469 Sir, Abhi desh ke sabhi state ka tour laga ne to nikle. Jaise 2014 ke election me aap nikle the. Aab dekhye kitna parivartan/ change hua hai ke nahi. 0 0
470 modi g ke jajbe ko koti koti naman 2 2

471 aabay india k sowar. cow ka peshab penay walay . pakistan aja. tujhe karachi ka peshab khane ly jaounga. roz 3 liter pelaounga. or khane hai tujhe cow ka gobar bhe khelaounga. maa ke choot tere or teray bhen l loray BAGWAN ki 0 0
473 Sir g hum pad likh kar be barujgar ghum rhaye hain .....hun etni mahnat kar kye pade or etna padne k babjud be koi kaam nhi mil rha sab paise kye piche hain sir g ....plz hlp me sir g 0 0
474 Future ki baat mat karo .Hamara aaj bigad raha hai. 0 0
476 I trust you so very nice improvement kabhi humne apne pm se nahi bola tha pehli baar hamari baat jaan pa rahi hai mujhe asahi pm hindustan ki zameen pe chahiye tha 2 2
477 sir desh wasiyo ke liye kuch karne ka irada h ya phir is air india me hi gumte rahoge 0 0
478 Safal videsh yatra ke liye badhai ho sir 2 2
479 Sir kya berojgaari ka koi illaj hai aapke paas 0 0
480 JAI HO MODI JI 2 2
482 Ye photographer thak gya h kya .. blurred photo aayi h ... 1 1
483 ghar me padhare gajanand ji ab Ghar me padhare.......... 1 1
484 Log apna sapna Pura karne ke liye puri duniya ko bhi dokha de sakta h 1 1
486 garib ro rahe h or tum jalse kr rhe ho 0 0
487 mananiy pradhan mantriji apko salam our ramajan eid mubarak 2 2
489 bharatakke bapa randi magana illi raitaru sayakattaru 0 0
490 ohhhh bsss krrrr Modi tenu ghuman toh sivva horr koi kmmm h k 0 0
492 Iske pas or to kam hai nhi chla hai for en country's 0 0
493 desh ki badkismati .. chai wala p.m...or padha likha p.m maun 0 0
495 congratulation sir ji 2 2
498 Ghen ke Lund apni bhen Chuda rha h randi ke 0 0
499 Extremist prick with hateful ideologies and hostile intentions towards neighbors. Ultimate dueschbag. 0 0
500 Hiii kam chhe 1 1
501 I heartly salute you Mr. Prime minister aapka karyakal Bharat varsh ke itihas me savarnim yug sabit Hoga Jai hi nd 2 2
502 Modi ji aap ko sabhi ne vishvas kiya hai but us par pani phirte dekh rahe hai mp balaghat mai pani ki tenk kitne dino se saf nahi hui hai bahot log bimariyo ke ghere mai sab aap hi thik kar sakte ho 0 0
503 Chaye bechate bechate desh mat bej Dena 0 0
504 Mera bharat mahan , Modi ji saccha insan, Des ki Dilayange sahi pehchan.jai hind jai Bharat 2 2
505 Modiji govt wonderful programs; jaadu apka safai aapka-swach Bharat,paisa apka account apka-jhan dhan,body apka mat apka-yoga day,beti apka responsibility apka-beti bachao beti padau,mobile apka beti apka aur selfie be ap ka-save girl child program.. Amazing fekuji.. 2 2
506 acche din aa gye 2 2
507 hiii modi jee kyai say ho aap hamarai bihar say aapko jada vott millai to bihar ko hi vull gyai hai pleace bihar ko taraki mai kijeeyai jaai say gujart ko tarrki mai kyahai 2 2
508 Kabhi to indiya me raho modi ji kasmir hamara janam shid adikar he 67 sal kangres ne bevkuf banaya ap ko jita ya he ab visvas mat todana piliz sar jitni bhi galt dhara he kasmir me use hatao baki des to ham yowa chalahi lenge 0 0
509 Aap ki tarah hi kisaan b hard work kar raha h lekin bachche plane m dikkat ho rahi h kisaano ko. 0 0
510 Ham log apane aap ko bhagy sali samajte hai ki desh ko aap jaise PM mile jay hind 2 2
511 desh aapko pakar dhanya hua the iron man 2 2
512 sir m aapka chuta sa fan hu par kuch bhe kar sakta hu 2 2
513 Kesi randi ka bacha, 0 0
514 mujhe apna bussnies start Karen keep lite 5 lakes ki jarurat hai 1 1
515 apke aane se sir hamaredesh ko freedom mil gayi hai 2 2
516 Ek bar Bihar ko bhi dakhie 1 1
518 modiji hamari ek gujaris hain ke hum ek sangsthan khulna chahati hain ke nij nij elakaomain sari lokoko help ka rna or iske liye help ki proyjon hain...... 1 1
520 Puraa Vishwa aaj Bharat ki or dekh rahaa h, 2 2
521 Bharatmata lovely son modi jaihoo 2 2
522 kya hua apka wada woh kasam woh irada 0 0
523 Kya ji modi ji mast lag rhe ho 2 2
526 Home guard logo ki bhalaye karo pechele baad me bade bade kaam kerna.Chotta sa kaam kerdo pechele 2 2

527 Bhus bhar 2. Ink daath milkar dushyon kaa 2 2
528 Coro or kutto pr brosa mt kro 0 0
529 Sir plz JHANSI ki bijli vyavstha thik Krane ki kripa kre, dhnyevad. 1 1
530 sir aapne skill devlopment programe lunch kiye jisase karoro logo ko rojgar milega !!! thanks sir 1 1
531 Sir swach bharat mission ko gawon se shahro ki taraf lao Sir mission ki jinmedaari nagar nigam or parishd ko ut tardaitav do Thanks 2 2
532 Sabka sath sabka vikas...Gud 2 2
533 Kyo modi ji bihar ke yua berojgar hi rhenge. Bihar ki gandi rajniti me sare yua piss rhe h 0 0
534 P m sab Aap ips abitabh thakur ki madd kigiye ga 2 2
535 Vikas ke liye govt ko kathor niym banana chahiye 1 1
536 dear prime minister job ke liye b kuch kriye sirf nitiya bnane se rojgar ki samsya hl nhi hogi 0 0
537 Modi saab g,, galla da krah bnayi jayoge, k kuj kroge v.. 0 0
539 Sir ab bihar me lalu nitish ka supda saf kro 1 1
541 Ghar Ghar modi hi modi .Jaha logo ki soch khatm hoti hai modi Ji waha se sochana suru karte hai . 2 2
542 Modi jorj aage bado ham tumhare sath hai jai Bharat.... 2 2
543 BJP ko lao beiapam karao phir mader ker aatam hatya batao 0 0
544 Modi ji aap ka sv asakeam fail hai... 0 0
545 Aapki skill scheme bhut achi helog kam krna sikhenge to km se km berojgar to ni rhenge....Thanks to pm for it 2 2
546 Modi j jitna aap ki yojna greatest hai hum aap k sath hai sir j 2 2
547 Desh ka vikas har koi petrol or diseal ke rate bda kar hi kyu krna chahta hai 0 0
548 bikaner kb tur h. halat kharab h bikaner k . surdhna gav jane wali sadak ka bhi kbi chal ke dekho us sadak pe.. 0 0
549 india digital bana dena modi sarkar pm 2 2
550 Thnx sir ji 2 2
551 Kam me visvas karti hai bjp 2 2
552 Today I am totally confuse........bcoz mai ATAL JI Ko best PM manta thaAb lagta hai Modi ji best PM of. BHA RAT Bolna parega,,,, jai jai ho 2 2
553 Hum bhuke mur rahe hai sir 0 0
555 danyavad bahi ji,shubharatri ji 2 2
556 Modi ji .....ye galat baat hai jo aapn 3 crore ka stage lagwa raha han ..... please think about it 2 2
557 Mane ajtak kisi PM se ITI ke vikas ki bat nahi suni thi muze lagta hai ye ache dino ki survat ho gai hai 2 2
558 Aadarniy pm ji aapke pas feuchar plan bahuthi aachha hai aapko salam. 2 2
559 BHARAT "MODI" ke Saath 2 2
560 Great modi ji,duja koi nhi aap jaisa 2 2
561 mission acchha hai bas lagu sahi se ho 2 2
562 Bhul jawo ki satna ka kuchh hoga 2 2
563 Modi ji agar 10 sal pahle pm bane hote to aaj india ki tasbeer dusari hoti sayad india china ke barabar me hota. 2 2
564 Ground par karo ye jyada Bakwaas karna band karo.....abhi tak logo ki life par koi fark nahi huwa. ....koi bhi p lan ya policy ka ground par jakar feedback liya karo.....policy launch karne se ya paise dene se janta ka bhala nahi ho ta. ....kyuki middle me khane waale bahut hai..... 0 0
565 Sir ji namo namoHumari muskile kab door hogiKaushambi ki light aur road ka sudhar pichle 10 year se nahi hu a 2 2
566 Modi ji aachi suruwat hai skill India program we heartly congratulated. 2 2
567 Bahut achchhakarya aur bahut achchha Hamara sarkar 2 2
568 Sir kabi Jammu Kashmir k ITI k haalat toh dekho koi staff nahi koi metrial nahi aur aap skill development ki ba at karte ho 0 0
570 Ati sundar sir sabse hatkar Vande~~~~~ sir 2 2
571 agar modi ji 10 saal pahle pm bane hote to ham aaj 20 saal aage hote 2 2
573 BJP youth ki skill niche video me dekhiye. 1 1
574 sir ji namskar , hamari (BJP) party ke kuchha netaon ne party ki chhavi ko badnam kar diya hai ese me unhain u nke pado se hataya ja skta hai , jisase dusra koi bhi esa kaam na kare, jai hind, vandematarm 2 2
576 sir hum apka sath hain 2 2
577 Sir aap ghumne ke waza Jo bhi ho aap agar odisha ka electricity me sudhar layege to kuch acha hoga or Jo baat

kiye the aap bo to kabhi nehi ho sakta bas electricity me sudhar dijiye bas 2 2
578 Aap jaisa PM har desh ko milna chahiye,jo apne desh ke future ke liye itni kadi mehnat karke aisi scheme launc h kar rhe hai.AAPKO PRAMAAM HAI MERA PM JI. 2 2
579 Sbi congresio se request h wo skill india mai addmission lekar kuch kaam seekh le.. khali baithe dimag khurapat krta h.. ghotale ko apni skill na samze. 2 2
580 Skil india to tab hoga jab india rahega modi g. Kanhi seej fayar kahi isi. Naksal waad. Jai hind. 0 0
581 Bhai jo apno se dur ho toh unka toh yahi ek zariya hai touch me rehne ka 2 2
582 Then indeed with hardship comes ease. Indeed with hardship comes ease! ~Quran 94:5-6 1 1
583 kya baat h..manwa khush krr diye.. 2 2
584 Kash khuda unhe vo samaj do jo mazhab ka galat istmal karke ek ki jagah char char bibio se shadi karke, aurat k i majburi ka faida uthate hai 2 2
585 Uncle! Uncle! har chiz mein religion..religion..kaiko? :-) Quran ho..ya bible.. aap ache hona pata chalta hai..bas. .. ushme bhi..ye sab ghuser diya kaiko? :-D 2 2
586 Ya allaha sab ko sahe rasta dikha 2 2
590 paye kuppi puthe vichil 1 1
591 sarkar ko dub marna chahiye ki iss umar mein unhe kaam karna padta hai 0 0
592 Respect to this Madam. No offence but Gujarat ko to kisi Mahapurush ne aisa change kr diya tha ki koi bhi insa n slum me ni rehta. 2 2
593 Jai jai garvi GUJARAT 2 2
594 jai bajrang bali.. 2 2
595 aaj to bhakt bhaut khush hongey . 1 1
596 Ye batao 60 Saal main congress ne kya kiya ;) 2 2
597 Desh chodo pahaley yeh media ko change Karo ... !! ? 1 1
598 Bhai yeh sab har police station pe hona chahiya facebbok pe nahi.. 0 0
601 Wo sab toh theek hai .. But yeh green apple mojito k saath Ras malayi kisne mangayi ?? 1 1
602 Doosro ka maar ke bana thaa, doosro ka maarne ke chakkar me phasa.... 0 0
603 The Logical Indian Kuch bhi likhte ho .. ek bar soch bhi liya kro likhne se pehle 0 0
604 Samsung galaxy Grand max 14000 Ka tha sale ke din 18000 Ka ho gaya , idr hum pagal nai behte hai sunlo com pany walon 0 0
605 Damn thm invisible ninjas cuttin onions.. 0 0
606 i love u salman vai.i like i so much.khuda hamesha apki saath rahegi vai.. 2 2
607 Sale salman khan abki dekhna hai ki teri pic kaise hit hogi bina muslim ke sale tum log muslim per daag ho 0 0
608 Kaheytey ho ki muslim fans pic nahi dekhey to bhi hit hogi 0 0
609 Salman bhai 1 mulaqat zaroori Hai apse milnii kii lv u bro.. 2 2
610 salman itni problem mei bhi desh ki duty ker rhe hai so why not.. 2 2
611 Salman Khan bhai ap ko likes or comments km milte hain shah rukh khan ki nisbat 0 0
612 hamara bhi kartavya hai clean bharat karana 2 2
613 jai ho bharat ki 2 2
614 salman khan app next film ki shooting agra city se karna plz sir 1 1
615 apun aap ka bahut bada se bada fen h 2 2
617 i love salman sir.....dost na koi manjil he na koi sathi he fir bhi nikal pada udhar se sayad jis ki talash he wahi sat hi he wahi manjil he......o o jane jana dhunde tujhe diwana........salman sir i love you 2 2
618 abe majid se chapal chori nahi hoti jise jarurt hoti he wo lekar jata he 1 1
619 ram ram bhai salman 2 2
620 Salman bhai aapke nagpur bb k promotion aane ka intezar hai 1 1
622 humara desh swach rahe ga tabhi humara india sab se uper hoga 2 2
623 meko itna gussa araha hai vomit kardugi iske muh mai tabi sudarega kutt# 0 0
624 bye sleeping bol de tere tak rahe muj toh bakwas lagta hai naam toh sunna hai bhut bar 0 0
625 usse bol de keep distance wid me nonsense admi jo sirf apna kam banta rakhe 0 0
626 hath mai hath rakhe baithe milo gye 2 2
627 ja rahi hu ek bottle pani pine ab pandey ji khabardar jo issko kabi mere samne laye bhi 0 0
628 tum kab jwab doge mujhe 1 1
630 meri jaan jo b km krti he vo nice hi hota he 2 2
631 tum milna chahte hi nahi kya mujhse 1 1
632 Jaanma main bol rahi hu ki,tum mere twits dekho :/ 2 2

633 maine kya galti ki hai mujhe kis bat ki sja de rahe ho tum 0 0
634 kam hai khatm kar leti hun bad me bat karungi 1 1
635 Bollywood bhi swachch kara do wo to abhi bhi nanga hi hai... 0 0
636 mai life me kya karna chahti hun tumse sadi karne ke alawa 2 2
637 Han businesses women banna chahti hun tumhare chakkar me sab bhul gai hun ek tum ho jise shirf mai hi yad n
ahi hun 2 2
640 Salman ji hey 2 2
641 salman bhai jan 2 2
642 tum q kar rahe ho aisa phone tak nahi karte kya hai ye 1 1
643 Kya yar hum Milne aaye or aap to mile hi nhi 1 1
644 Suna he ki ap actors log aam janta ko reply nahi karte aisa nahi karna chahiye 1 1
645 Salman khan Aapko Aap ka feyn .taranam q stail ma dakhna chahta hai . 1 1
646 salman sir aap kaise ho plz reply b de diya karo 1 1
647 super bajrangibhai jaan super hit movie 2 2
648 Punjab mein Babbu maan aur bombay mein salman jaisa koi nhi hai .. In dono jaisa koi aur nhi,paida ho sakta ..
Salman bhai ek song Babbu maan ka to banta hai apki next movie mein 2 2
649 Salman bhai agar aap ne apni movie mein Babbu maan ka song le liya to kam se kam 20 laakh fan ek din mein j
yada ho jayenge 2 2
650 Bohat khub Salman bhai 2 2
651 Bhai ak bar haryana me aajao aapko bhut achi khani duga 2 2
652 i sapourt u saloo bhai i hole u r bajrangi bhaijan it's going 2 2
653 i love ur film joi ho bhai hope ur flim bajrangi bhaijan 2 2
654 haha.... im selected... <3 mzaaa aa gya... umahhhhh bhai luvv uhh 2 2
655 maine bol tum so jao mai subh mangalugi 1 1
656 ache se samjha ke chali gyi aur mai so gayi 1 1
657 thanx salman bhai..love u forever 2 2
658 India no1 star salman bhai 2 2
659 Bhai jan jindabad 2 2
660 hi my cute bandar kha ho kya ho rha hai thik ho miss u sona miss u so much 2 2
661 plz cal sallu bhai +8801719447771 bangladesh fan plz cal met u sallu bhai 1 1
662 hiii mai ok huiii :) 1 1
663 Abe tu kutta hai be...teri movie flop hogi...dkhna tere kitne fan kam ho gaye... 0 0
664 hmmm taliyao ki awaz abi se meko sunayii se rahi hai hall mai sab sirf ek hi baat bolte hai wah bhai wahi mai s
harmake chupke se hasti hu aur love u dilse 2 2
665 teri picture dekhne jao nah maja a aajaatah hai such iiiiiii 2 2
666 mai suchii boliii sab hall mai bhai bhai wah bhaiii hi bolte hai haste hai josh mai aate hai bhut maja aata hai 2 2
667 Ek zaroori baat he k koi bhi musalmaan bhai salman khan ki (BAJRANGI BHAIJAAN) movie dekhne k liye na
a jaae. Us harami ne musalmano ko challenge kiya hai k wo ... 0 0
668 Abe tu ye sab kisse bat kar rhi hai yar 1 1
669 Sona last time tu jab ayi thi tab kitna kuch kiya bas ab 1 chij bach gya hai tu aa ja jaldi plzzzz 1 1
670 bhai koi photo jo aap ke dil ke karib ho 2 2
671 Allah se dua hai ke teri film supper supper supper flap ho malon 0 0
672 Main Hindu hu Bhai bajrang Bali bhi Hindu god hai gau mata ki Bali dete ho sir aur bajrangi banate ho 2 2
673 Aap to supar hit ho aor aapki film supar hit hai 2 2
674 meraa paarsal miila 1 1
675 Sacha bharat INDIA 2 2
676 hlooo shalu bhai kya hal h 1 1
677 dil kahta h 1 1
678 muje pta he aap reply nhi kroge phir bhi apki film achi rhe and mey docter bnna chatha hu dua kro 1 1
679 :'(:'(:'(:'(:'(tum gande pati ho bhot gande I hate u I really hate u 0 0
680 meri koi ijjat thodi hai 0 0
681 Soch or bichar bhai ki tarah rahna chahiye 2 2
682 kya baat hain 2 2
683 Aap jaisa kimti mind sabke pas hona chiye issse bimari nai banti hai 2 2
684 nich bhai g 2 2

685 Meri akhri khosies h Ek br tujse sirf milna chate bs 2 2
686 i love you salman sir...aap ki movie dekh raha hu abhi judawa .. 2 2
687 Mera toilet bhi saf ker jana Salman Khan 0 0
688 Realllllly loooovvv u salman 2 2
689 Asli swachhta safai krne se hoti hai sir photo khinchwane se nhi 0 0
690 SirAapne Jo musalmaano ke khilaaf statement diye wo galat he 0 0
691 kiya kismat hai 1 1
692 Isko itna time knha hoga k aap sab ko comments par nazar daleHa ha ha ha ha ha 2 2
693 Hum ko b tho chance dedo yaar salman 2 2
694 salei suwar ka pilla .... mar jaa aishwarya ke moot mei doob ke ..cheeh Modi Aur Rss ka Nya Kutta 0 0
695 Sir mat bolo sale ko harami hai wo muslman ke name par dhabba hai sala saitan 0 0
696 Lv u bhai I cnt wait fst dy fst shw bhaiiiiiii 2 2
697 kya karne wale ho kuch soche bhi ya koi phark nahi padta 1 1
698 Bhaiya me bhi kuch karna chata hu pliZz 1 chans me Bhai 1 1
699 Grt super star sallu bhai 2 2
700 GREAT SALMAN BAI 2 2
701 looking soo handsm??? 2 2
702 harami sala salman... 0 0
703 Mera ko bhi ek chance de do bajrangi bhaijaan 1 1
704 teri movie flop ho jaye.... 0 0
705 Sale tum hrami ho moslim nahi tum saytan ho 0 0
706 I love u salman khan ek bar apsa milna cahti hu ya mara sapna ha bas ek bar love u love u love u love u love u s o mach salman khan 2 2
707 Kamal karte ho yr bhai 1 1
708 Wow kya baat h itne comment maza aagya re baba re from khushi hasan nd my real name ruby hasan 2 2
709 Kya ye abhiyan abhi bhi jaari h 1 1
710 Thik h bahi jaan ham ko bhul gye naa 0 0
711 Hum bhi h raho me kl mene haji ali dargha pe salman bhaijan ko baijat bari hone ki duwa mangi 2 2
712 hello sir aap online ataa ho pata b nai chalta kab ataa ho aur chale jaate ho :(1 1
714 arey yo toh apna hi balak se 1 1
715 sem 2 u 1 1
716 Me Delhi se hu aapke security gourd se kaha na usne milne diya or n aaptak msg pahuchaya plz meet me 1 1
717 Bhar do jholi meri y Muhammad mujhko Salman sir. S milado, jb tak aap bana d n bigri dr s n jao khli 2 2
718 salman ke haters ko koi comment hi mat karo..tottaly ignored..great salman 2 2
720 Bhar do jholi meri ya Muhammad mujhko salman sir. Se milado 2 2
721 jabtak public nahi sudrega tabtak kuch nahi ho sakta. 0 0
722 Salman sir. Me apse milna chahta hu plz mujhe apointment dijiye me realy aapse milna chahta hu plz 1 1
723 pls mane call karo pla pls 1 1
724 Salman sir. Mera aims pura kardo me aapse milna chahta hu mujh se mil lijiye 1 1
725 pls mane call karo 1 1
726 Sir aap mere lye mere roll model h ..mai aaapse hi inspire hokar aaj delhi police mai hu and logo ki help karta h u...kbhi aapse mila to vo mere lye bhagwan se milne k brabar hoga.. 2 2
727 mera bharat mahaan padhega India tabhi to aage badhega India 2 2
728 8527106761 plz Salman sir. Mujhe aapse milne ka moka den 1 1
729 Bhar do jholi meri ya Muhammad mujhko Salman bhai se milado 2 2
730 Please Salman sir. Is garib ke lye bhi time nikalen aapse milne ka apointment den 1 1
731 Bhar do jholi meri ya Muhammad mujhe Salman bhai se milado, 2 2
732 Salman sir. Aapki life ke 2 mint meri life anek mint me khushya la sakte he plz sir. Mil lijiye 2 2
733 Salmaan sir bole or kaam na ho..sawal hi nhi ho skta...god sir Aap to sir..my god 2 2
734 Ek rply to kar do salman g ... ab to dekho sab ko pta chal gya.... lakin aap ko kab pta chalega 1 1
735 Aap to yaar supr hiro ho 2 2
736 I Iike and love u salman khan aur ha kya such me aap hamare comment padhte ho aap tv par bol rahe the mai sar e comment padhta hu ya u hi fake rahe the bhai ?? 2 2
738 Apne aqirat ki fiqar karo wanha kon bachega sirf allha tobakarle nada 0 0
739 apka fan ho bangladesh me plz cal sallu bhai met u +8801719447771 cal bhai 2 2

740 Flop jaaigi movie teri....aehsan framosh.. 0 0
742 Mami papaa , aur bacha party aur Sab kaise hai 1 1
743 Kya socha hai shaadi k bare mai 1 1
744 Mai jaanti agar tumne use shaadi Ki to WO mujhe tum he kabhi baat tak karne nhi degi mujhe pata hai aaj usko Meri zarurart hai to WO mujhe itna importants deri 0 0
745 Aur WO achi ladki hai 2 2
746 koi aur pasand aagai ye ek jagah tik nahi sakte tum 0 0
747 Bhaijan main aapka baut bada fan ho i love u bhaijan jabtak bhaijan he taptak main ho 2 2
748 Salman g meri asiqi sirf tumse hai 2 2
749 Kya kah diya sallu bhaijaan 2 2
750 Asslamu alyekum bhai ek bar jwab jarur do bhai 1 1
751 Salman bhai sirf sohrat kamane se kuch hoga. thoda bahut khuda ko bhi yaad kiya karo.mahe ramjaan bada hi p ak aur touba karne vala mahina.plz ibadat bhaijaan 0 0
752 Salmaan bhai maine hay kaha tumne javab nhi diya 0 0
754 sarukh or amir khan bhi apke samne fel; he 2 2
755 Mortath kafir hy tu salman kanjr 0 0
756 Kafir hy tu salman knjr marwasy k btty 0 0
757 salman sir aap london kab aao gaye plz aik baar ajoa i want to meet u once in life u are cororo mein aik sir 2 2
758 Salman ek baar jawab to do yaar 1 1
759 salman aap to hmare jaise ho eek baar kuch bolo 1 1
760 salman khan ji you looking owsm i love you salman me bta nhi skti ki me apki kitni bri fan hu 2 2
761 salman khan nice 2 2
762 Abhi saheri Ka waqt bhi khatam nhi hua aur azaan hona bhi baaki hai 1 1
763 Kya karu samaj nhi aara bhot neend aari hai par namaaz is very important s 1 1
764 Namaaz padk dua karte ho k nhi 1 1
765 K bas subha subha uthe aur kasrat karna chaalu 2 2
766 Raat mai late soie thi uYe Abba haina raat late aaey 1 1
767 Mare favroute sallu vi... 2 2
768 assalamualikum jumma mubarak 2 2
769 Salman khan sir tayyar ha aaj ke party ke liye 2 2
770 jiske pas ilm he wo sabse amir he 2 2
771 Anjana Dhamodharan hi suweet hart buhat achi lag rahi h0 2 2
772 h0w are y0u my freind kese h0. 1 1
773 Real hero sallu bhai ^_^ 2 2
776 Bhai mujhe bhi kardo nominate plz 2 2
777 bhai duva me yad.mahi 2 2
778 Bhai app bollywood king hokar apne app ko bajrangi bhaijan kya late ho Eid me a sob ko aacha nahi laga bohut sms araha ha is move ko mat dikho appne majhab ko chota kara ha kay a chsch ha? 0 0
779 Bhai kya app ka mobile number muje milega a mera mobile number 9662821362 1 1
780 bhai kya hal hai 1 1
781 Swachchh bharat aviyaan ke liye danyabaad.... 2 2
782 Ramzan ka mahina dil khol kar Ibadat karo 2 2
783 bhai lv u......aapne zindgi me ek baar milna h...plz bhai kbhi ye msg pdho to.....plz mujhe ek call krke.... meri lif e khushiyon se bhar dena... 2 2
784 Ap shdi kr loooo 1 1
785 Ap salman ich hai 1 1
786 salam vai jan 1 1
788 u welakam saluu 1 1
789 Kiya meri ap se bat ho sakti hai kisi wkt fb par 1 1
790 Mr. KHAN maine aap ki sari baatein suni aur use follow bhi kiya. Trust me. 2 2
791 Okk boss apka hukum ser ankho ma:) 1 1
792 aap mere liye rkhe na naam2 1 1
793 apun kapda khole apna body dekh ke ldkiya kpda phna istrt kr degi colr tak usi ko sikahne ke li apna kpda khole nge 2 2
794 Bhai bajrangi bhaijan to samjho ki HIT HAI 2 2

795 Jane kab Meri neend gai 1 1
796 Chalo zayada smile karne Ki zarurat nhi hai 1 1
798 Bby i love you hmasha milungi yhe mrta dam tak kbhi to rply doga 2 2
799 salman i luv u alot n i wanna meet uh...plzz mjhse milo ya chat kro mjhse plzzzz i luv u ummaahhh 2 2
800 sir plz london aajoa plz 1 1
801 dhasu lag rahe ho salman bro miss you........... 2 2
802 Kiya bhai jaan 1 1
803 Agar aap na media mein ya kha hoga ki musalmano ki bigar bajrangi bhaijaaan hit karunga then u are a foll 0 0
804 Kya be salman bhadve 0 0
805 Jumma mubarak bhaijaan... Kbhi hume b ghar eid pe bulao... Jst joking... God alwz bless u, allah ki rehmat rahe, ganeh ji ka hath sada apke sir pe rahe, vaeguruji k shatrachaya me aage badhte jao, all gods wid u alwz.... 2 2
806 Kar lo bhai sadi acha moka h 1 1
807 Salman khan criminal. 0 0
808 what loook bhai jaan 2 2
809 Kiya huwa bewde muhe kiyo latka ke behtha hai 0 0
810 mai salman ka frnd 2 2
811 Salman bhai boleto rock star 2 2
812 Ek ch bhai salman bhai 2 2
813 Bajrangi Bhai hamse bhi guftgu kar liya karo........ 2 2
814 Han raakhi bhi tum sab hi bandhana ...Gazzab ladkiyan hain yaar!!! 2 2
815 i love u toooo sooooo mache salman khan 2 2
816 hy sir me apka bahut bada fen hoo 2 2
817 Hamein kaash tumse muhabbat na hoti :(2 2
818 my salman khan 2 2
819 Nice pic kaise lagte ho aap itne handsome 2 2
820 Bhai jaan tho bhai jaan he rahage koi aur unke copy nahi ker sakhta yaro yes or no 2 2
821 Hallo ji kya hall hia me Ramu kuma 1 1
822 sir aap kuch dhyan nahi dete pLz plz plz 1 1
823 aaj ki party meri taraf se nice song bhaijaan 2 2
824 Good night sir.....capil comedy..me aaye mamko bahut achha laga...Good.night.sahb g.... 2 2
825 Masti he mohl me chahi khumari he saarey thak ke beth gye per apni party jaari he @@FEELING HAPPY WITH THE SALMAAN@@ COMING SOON BAJRANGI BHAIJAN ON THIS @EID@@ 2 2
826 bhai kabhi to apne pas bhi to dekho , khudko bhul jaoge he he 2 2
827 mast ha salman khan sir songs 2 2
828 Muslim ka nam mat kharab karna salman bhai chahe kuch bhi karo koi tention nahi 0 0
829 sallu bhai <3 2 2
830 Hiiii.... Bhaijaan kaiae hai aap...aap ki pic bahut achhi lag rahi hai 2 2
831 Main aapka bahut bada fan hu 2 2
832 luv u salman khan 2 2
833 bhai aap ki bhaijaan movi bhi 200 cr karegi 2 2
834 Eid mubarak songs bajrangi bhaijaan me hona chaiye 2 2
835 Looking katilana andaaz 2 2
836 Salu bhai dabangg 3 kab release kar rahe ho 2 2
837 Salman khan ke pe sari duniya marti h ehi 1 2 2
838 Sallo bhai roze kase hy 1 1
839 Tu muslman nahi h kiyo ki tu mandir jata h tu kadiyani h salman 0 0
840 dashing salman khan .. 2 2
841 App ko ramzan ka mahina mobarrak ho....or app ho allah tala or tarraki de.... 2 2
842 Indian's star salmankhan 2 2
843 agr tom na hoty to bollywood ka maza bi na hota haha 2 2
845 Meri zindingi aapko lag jaye bhaijaan yeh dua hai meri 2 2
846 Abe salman agar tere me itna hi dum h to IED me film mat release kiya kar fir dekhte. Dekhte h kaise hit hoti h Teri film. 0 0
847 Salman Bhai yeh bajrangi bhaijan pe comment aa rhe hai is movie ko nhee dekhna 0 0
848 Saale salman tu musalman ke khilaf hokar bohot niche girega 0 0

849 Salman bhai masha allah aap ek achchhe act ho ek achchhe insan ho kya aap ek achchhe muslman ho 2 2
850 Salman always kind heartnt cmpairjo log muslman ,hindu kr rhe hun logo ki nichi soch pe mje gheenn aati h yr 2 2
851 Salman bai ap kese hen ma b ap ka fen hun 2 2
852 Apne naam se khan hatalo 0 0
853 Aapse baat karna chahta hu bhai .bahut badi problem mae hu.aapki help chahta hu 1 1
854 bhai jan ekdam kadak 2 2
855 Nice bhai WHATSAAP number 03101419966 palz 2 2
856 Salman khan ki jai ho 2 2
857 Nice pic abdul rashid saleem (salman khan) 2 2
858 BHAI. BHAI... nice pic 2 2
859 Sir aap humanbieng chalate hai aap paise dete kab tak jab tak aap hai.mai apko aisa rasta bataunga sab karodpati ban jayege. 1 1
860 Bhai apky chryti so me allho pyyr keep pas he apmuslim hokr galt kr rhy ho khuda hafij 0 0
861 salman ji aapse apni life me ek baar jaru milna chate hai..... aapse baat karna chate hai..... jo aap logo ki help kar te ho uske liye thank yu so much....... aapko meri bhi umar lag jaye 2 2
862 Tere naam ke ladke diban h pata nahi 2 2
863 sirji...pls visit to Kanpur oncem...plssss. 2 2
864 Salman realy good yar 2 2
865 Bahi ke zalak sab sa aalag..... Ma to superman salman ka fan.... 2 2
866 Insan jab tarki karleta hai to wo apne mazhab KO bul jata .hai jab thokar lagti hai use to use apni galti ka ahsas h one lagta hai .....koch aisy hai salman ki khani mery Kuban I....... 0 0
867 Beta salman khbis tera film falop hoga beeee suar 0 0
868 plz cal sallu bhai bangladesh big fan me plz cal +8801719447771 jai ho 2 2
869 Salaman to harami he......tu musalman hoke kafir ban gaya he....sale kutte...samne to aa jaan se maar du tuje.... 0 0
871 Abi tak jag rhe ho sab log? 1 1
872 Love u jend actor wo jo har lock mein sweet beautiful guineas hot sohran lagey osy khtey hain. 2 2
873 Aaj tak par news sache hai plss reply ......Ansar 1 1
874 bulawa ho na ho ham yayege or entjar ke liye chale jaye ge 1 1
875 Thu thu thu kuttey... 0 0
876 Salman khan dest actar bollywood 2 2
877 Handsom to hai mera bhai bhai he mera 2 2
878 Hi bhai aap kaise ho bhai aap ka fan h 2 2
879 Dost apki har ada achi lagti h 2 2
880 salman bhai look to bahut achcha hi bhi 2 2
881 very very very very very very very very very very very very very very very very niceeeeeeeeeeeeeeeeeeeeeeeee eeeeeeeeeeeeeeeeeeeeeeeee and i loveeeeeeeeeeeeeeeeeeeeeeeeee uuuuuuuuuuuuuuuuuuuu bhai jaan 2 2
882 swgat nahi karo ge hamara 2 2
883 Idoit...salman aap muslim hai...Aisa faission show kyon karwaya..allah likkar 0 0
884 Ap ne kya kia h Jo log itne bure coments de rhe han.mje ni pta.kya ap btae ge? 0 0
885 salman khan rock's ss 2 2
886 Merit Maya ka oppreation hai app ki madaat melaga 2 2
887 Bahot accha hai 2 2
888 Nice pic salman bhai aapne to sab ko diwana bana rakha hai 2 2
889 sab ka Bhai meri Jan Salman khan 2 2
890 Bhaiya main aapka bahut bada fan hu 2 2
891 mast hai bahi 2 2
892 Hye meri cutey 2 2
893 ara bhai jaan hum bhi to aap ke bhai hai chote bhai jaan... 2 2
894 Sale ne muslmano ko chalenge kiya ki tumhare bina hit karunga 0 0
895 Salman murda bad 0 0
896 Ye to hogi super supar hit dum hai to KOI TO ROOK LO 2 2
897 Yeh ki yeh namard hai 0 0
898 ku salman ka naam bina use kare kam nhi hota kya bhai logo 2 2

899 Kute ka bacha hai 0 0
900 jo dusaro ko ganda bolte hai woh khud wahi hote hai 0 0
901 Kay kahna chah rahi ho 1 1
902 Bhadra hai sala 0 0
903 job chahiye hm kuchh ldke jobless h, or hone wale h pdhe b km h bt work krna jante h 0 0
904 bhai ur evergreen 2 2
905 plz aap mere frnd baniye 2 2
906 Sir Mera sapna hai ki mai aapse milu salman sir ek baar.pls Nagpur Me. 2 2
907 Bajrangi bhaijaan ek no. 2 2
908 Akbarudin ovasi ek Sacha Muslim he or tu sale Musalmano ke naam pe kalank he sale suvar jese saqal Vale 0 0
909 salman khan fans is gaandu 0 0
912 Budha hai salman khan 0 0
913 De tera aur salman ka character hi waisa hai chip 0 0
914 Salmon bhai mai Apki boooooohat bari fan hn. 2 2
915 apne aap ko kya samjhte ho mushalman ko challage dete ho ab dekho hum mushlman bhi kuch kam nai he 0 0
916 Agar mushalman ko challange de rahe ho to bro movi m bhar de jholi wali kavali kyo aad ki ????????? 0 0
917 woh ganna sab mere liye banna rahi hai mai suune gi 1 1
918 Gr8....I love salman.....challenge bhi good or challenge dene vala bhi good........ 2 2
919 salman khan bet hiro mai yuske sath pkchr me ka krna chta hu 1 1
922 bhai tum shahi ho 2 2
923 Nazar na lage 2 2
924 bhai apko nahi lagta ki kareena ne bhot zyada make up kr liya hai movie mein.. #Salmankhan 2 2
925 Mujhe shadi karni salman apse plzzzzzzzzzzzzzzzz marry me 2 2
926 Bhaijaan Allah ki ibadt aap bani h bani rhe bani rhegi bajrangi bhaijaan jrur hit hogi 2 2
927 musalman ho k... apne log ki baja rha h... ise flop kr do bhadwe ko... 0 0
928 Koi frnshp kre ga? 1 1
929 Bhai apks bazrangi bhaijan movi song verey nice 2 2
930 Harami kutta sala lanat ho tujpar 0 0
931 Teri film flop hogi dekh tu ab salman khan 0 0
932 hi sallu bhai <3 2 2
934 Iska film mat dekhna dosto 0 0
935 Chlo new frndz bnate hn 1 1
937 Yo yo ka song hona chaiye tha 1 1
938 Hallo salman sir 1 1
939 hello 22 jaan 1 1
940 Salman bhai ab to ap shadi kr lain... 1 1
941 bhai salman bhai 1 1
942 as salamolay kum vai jan 2 2
944 teri aukat kya hai khabbis jo ham musalmano ko nich bolta hai 0 0
945 salman bhai aslamolikum ... 1 1
946 Vani ap salok se raho muj ko jo b bura kahe ga allah pak deketa hai 0 0
948 Salman khan sir suna ha ap PAKISTAN ah rahy ha 1 1
949 Salman bhai aap ye comments padhte ya reply karte ho ya nahi? 1 1
951 Chutiya salman.....khan banna hai to pahle shahrukh ban 0 0
952 Galt baat ke muslmano kai khilaf ovisey ka sath de na de.....galt q bola...? 0 0
954 Duniya me sabse acha kon hai to o sirf hamara veer Q ki o sirf Dil ki sunta hai 2 2
955 Bhikari eid Ka jkate sedka fitra lene aa rha he 0 0
956 Hai salman bhai 1 1
957 Hi salman ji me apka bahut bada frend hu 2 2
958 tumhare dil me kuch na kuch khot to hai phone nahi karne ka region samajh me nahi aata aisa kis relationship m
e hota hai 2 2
959 Gud morning bhaijaan 2 2
960 galti tum kar rahe ho aur saja mujhe mil rha hai 0 0
962 SALMAN khan KI maaki chit uski maaki chutme modi KA lawda saala harami KI janwaad naaliya kholia KI p
aidaas uski bahen KI naak me ganpati KA land WO chinaal Randi maadarchod hai 0 0

963 Love love love 2 2
964 Han bro roza to rahkata hun 1 1
965 Chutiya no 1......jo apne dharam ki nahi ho saka o kisi bhi dharam ka nahi......kehte hai na log k..jo khud ko pyar nahi karta o kisiko bhi pyar nahi karta 0 0
966 mai tumhari jitni achhi nahi dikhti par tumse bhot achhi hun tumhari trah jhutte pyar ka natak nahi karti 0 0
967 na tum mujhse pyar karte the aur na hi ab karte ho sadi to karna hi nahi chahte isliye na phone karte ho aur na hi milte ho jhute makkar dhokhebaj ho tum 0 0
968 bb me jo kuch kie kahe sab bhul gae natak tha na sab camera ke liye kie the na 0 0
970 Vidya gupta pagal ho gai h kay,.,,.,. 1 1
971 Mujay is baat ka fakhar hai kay ap muslman hai. 2 2
972 Hi Salman sir love u piz ek bar muje se bat Karo na sir piz piz piz 2 2
973 hi bhai aap ki film super ho jaye bajrangi bhai jaan 2 2
975 Naice naice naice.... 2 2
976 jo ganda log hai woh ganda hi rahega according to pychology u cant change anyones behavior woh act karege p ar change nhi hote ! 0 0
981 tum se pyaar <3<3<3 2 2
983 Salman vai pls apne dare rakey..vai app ko boss boss lage ga.. 1 1
984 Oye tu kyu badi badi baate karra hi...... Pagal ho kya... 0 0
985 Assalamu Alaikum Salman KhanHum aapke fans chaahte hai ki aap ek baar haj ya umra karke aaye . Inshaa All ah aapki life ki mushkile aasaan hojaaye . Aameen. 2 2
987 Salman g agar ye msg dekh rahe hoto reply karo plz 1 1
988 Pata nehi ap msg dekh rahe ho ya nehi 1 1
989 Par me salman g se itna pyar karti hu k ye page to unka hai,isi liye jyada comment me karti hu,taki unhe pata ch ale k me unki kitni bari diwani hu 2 2
990 Love u salmaan khan ji love u so much mithuuuuu...mmuuaahh 2 2
991 tum kis mitti se bane ho koi tumhare sath 3.5 se bat kar rha hai 3.5 sal se ek hi chij se ek hi bat kah rahi hai tumh e koi phark nahi padta 0 0
992 sare aade tede mere hi kimat me likha hai upar wale ne chun chun ke lga tha tum to sayad sahi hoge lekin nahi 0 0
993 woh twitter pe painting tu kia acha tha 2 2
995 Kutta hai to na to muslim na to hindu 0 0
996 Nhi nhi thanks nhi 3 logo ko aur bta dena 1 1
999 but Salman bhai aap shadi q nahi karna chahte 1 1
1000 Girna samal nai lage 1 1
1002 Koun bolhata hai Saadi nahi hogi ..meri aur Salman ki saadi hogi 2016 ..October ..first week :) :) 2 2
1003 jaan meko bol tuje koi pareshani mai apne bhagwan ko chahta hai jawab de 1 1
1006 mar jaye woh log jo mujse jalte hai mere bharmini hone se jalte hai unka wenaash ho jaye 0 0
1007 Salman handsome king 2 2
1008 meri baat par kisi bhi hamazade ko koi bhi doubt ho shock se aye mere village 0 0
1009 salman fans addme <3 1 1
1011 jo mujhe req bhejenga mai usse apna nr dungi promise 1 1
1013 Salmaan khan bollywood rss or bjp ka naya kutta 0 0
1014 sidhi vinayak ganesh ji ki jai hoooooo 2 2
1017 Kutta harami madar chod bighrat salman to agar mje mil jai teri maa bhin ek kardonga 0 0
1018 Salman khan g kasy ho ap eid par jo movie ha wo tek ha par name tek ney ha mara msg dak lana g ok 1 1
1022 Muskurane ki waja tum ho 2 2
1023 Hay sallumeya eid mubark ho 2 2
1024 Salman meri family boht sharif he hm apni bf k sath ayashi waste apne maa baap ki izet khak me nai mila sekte 1 1
1025 salmaan khan tumhare naam k pichey khan accha nahi lagtahai hata de muslaman ke naam ko jo itna hi bura ka gta hai islamic dharm to abhi chod de zarurat nahi hai tumhari islaam ko 0 0
1026 bb ki liye toh maine already bol rakha hai abi se 1 1
1027 meri bas ek dua hai ek bar mujhe milna hai apne bhaijan se 1 1
1028 kal lakshmi mata ki aarti hogi subha ke waqt namaste jii 2 2
1029 jo jo gali diya hai sabko sunna hoga ab 0 0

1030 ami salman ke valobasi...i love u SALMAN 2 2
1033 No.diziye whatsupp ka photo bej dete hai 1 1
1034 aap mere gao ki kahani p ek film bana do... 1 1
1035 Ek baat btao 1 1
1038 ALLAH aap ko har taraf se kameyabi de 2 2
1039 Helo salman bhai jaan. 2 2
1040 Salman Bai Agar ap ya Coments par ra han to plz ans b dan ap kasy han Aur rozay kasy guzar raha han. 1 1
1041 Hi salman i'm sameer baloch in balochistan i'm biggggg fan of you 2 2
1042 bahut ganda msg tha 0 0
1044 bajrangi veer putra dal de 2 2
1045 loveu tooooo ur vey handsam 2 2
1047 ye sab kuch nahi hai exal aur coral me bhot sare formula and short cut hai wo yad rakhna padta hai 1 1
1049 swal kai hai jwab kab doge 1 1
1050 Etni lambi speech sa kuch mi hotta sirf 2 word khna or unka suna sa frk atta h .... sekho i love you sallu?? 2 2
1052 sabse bada fan hu salman bhai aap ka 2 2
1053 Rfta rfta dakho akh mari kadi h akh jisa lgi mera samna vo khda h i love you salman ji?????????? 2 2
1055 bas bhai 24-25 ke lag rahe aap to . 2 2
1057 hello sir kaise ho plz reply tou de dou aaj plz sir 1 1
1058 sir orissa pe kavi aao na.plz... 1 1
1059 Jaldi btao bhai jaan 1 1
1060 bhaijan kolkata ka har log ready.....up ekbar kolkatame aajao 1 1
1061 Aslma alikum bhai jaan 2 2
1062 ye sabka record todne wali he 2 2
1063 Bollywood king my bro salman khan 2 2
1064 Bajrangi bhaijaan sab record todegi salman bhai jiii 2 2
1065 Salman aapse milne ke liye Hume kya kare 2 2
1066 Bhai jaan apse ek request tha.... ek baar apki koi dance functions me dance karne ka moka dete toh.... ye jindegi ki sabse bada dream pura ho jata 2 2
1067 lov u sallu bhai 2 2
1069 Ramzan me sabki madad karte ho aap isiliye vo aaye the 2 2
1070 350 cr kamati ab, 1 1
1071 tayyar ho gaye kab se,ab jaldi aao! 1 1
1072 salman ji hm jarur dekhenge aaj aapka teaser and 17th july in cinema Bajrangi Bhaijaan...love you salman ji. 2 2
1073 Han bhai aaj raat party to bnti h 1 1
1074 kuchh reply to dijiye salmaan bhai.. abhi fauran...... 1 1
1075 Ha bhai kab se tyaaar hai Patiy ke liye 1 1
1076 Eid ke bina salman khan ki movie flop..akshay kumar ki movie chahe jab release ho hit hoti hai.. 0 0
1077 Yessss my cuteee salman 2 2
1078 Salman Bhai aap cinta mat karo apki movie ham hit kare ge 2 2
1081 bhai plzzzzz apni movie 27 ramzan ko release na kiya kro. its my request q k ramzan ki 27 v rat(holy night) ko shbe kadr hoti he. sare muslman ibadat krty he. ap b muslim ho na. #beingsalmankhan #bbthiseid #aajkiparty #bajrangibhaijan 1 1
1083 iftar party mere taraf se aajao 1 1
1084 Aap log bahot amir he aapke liye party karna aam baat he par un logo ka kya Jo aapna ilaj bhi nahi karva sakhte jese ke meri friend use apki help chahiye aor aap party kar rahe he aap uski khuch madad karde pliz 1 1
1085 eid baad hogi bhai party to wo bhi bajrangi bhaizan movie ke saath sbhi no tv no movie 2 2
1086 are salman sir agar mere coment ko padhma to plzzz ek bar reply karna 1 1
1087 kya yar kuch muslim bhai salman bhai se q naraz ho rahe ho salman bhai acha insan he 2 2
1089 Pehle roze to rkh le fir eid manana... 1 1
1090 Bilkul kab se net pe search kar rahe he aapke gaano k liye bajrangi bhaijaan. .. 1 1
1092 Kyu nahi ap eid per ap bolaye or hum na aye hum jaror aye g 1 1
1093 Kaum ke gaddaro ke sath parti nahi krte hum 0 0
1094 Salman ki diwaniya muj se ek baar chat krlo plzz...... 1 1

1095 Sory main nhe aasg t main pakistan main ho??? 1 1
1096 Movie flop ham sab ke tarfse aap ko bhai mmubarak 0 0
1097 Mjh say bat karo na sweeeto 1 ber bus 1 ber 1 1
1098 Yunki party k liye to hum ready hain par kya karen nahi aa sakte kyonki invitation sirf bhaiyo aur behno k liye hai......... 1 1
1100 no way...pura ek mahina ramzan ki ibadat k baad eid mey aisi movie kon dekhega jaha hanuman chalisa padha a ja raha ho.... 1 1
1101 Râ€žj'Å’v Vâ€¹Vâ€ â€˜Â¤ Ready ho bhai aaj ki party ke liye 1 1
1102 Main to nahi ye movie dekhuga Salman ki har ek movie dekhi but ye nahi dekhuga 0 0
1103 koi bhi larki fedda ho jaiga app ka upar dekhna app 1 1
1106 ye vi bolne ki baat h ap bulayen aur hum na ayee #bhaiyjan 1 1
1107 Big brother party de or ye chota bhai party me nhi aae aase ho skta hai kya kbhi #Salu bhai(bajrangi bhaijan) 1 1
1108 Bhai hamara naseeb kha hum aapke sath party kar sake 1 1
1111 nar ja kutte 0 0
1112 Ap riyel salmankan hai to sun no ke pahele ye socho ke msalman hai ke nahi hai to akhirat ki fika kar 1 1
1113 Lovly sallu bhaijine 2 2
1114 bhai iftaar farty hai kya? 1 1
1115 Kis khushi me tyar hojae bhai shadi ki party dere ho 1 1
1116 yrr ap bhi kiss ke movie dekhne jaoge jab is ne he bol diya ki mere movies musalmano ke bina bhi chalte hn 1 1
1117 aaj ki party bhai k taraf se 1 1
1118 kiski party bewakoof badtameez 0 0
1120 Salman ab hindu bangya he bhayoo koi movie dhkhne nhi jaaye 0 0
1123 ek dum tayyar 2 2
1124 bhaio ye khabar afwah he Muslims ko challenge karne wale 1 1
1125 Super duper flop...inshallah nich hai Salman Khan tu..? 0 0
1126 Hm toh taiyar bs apke tasrif rakhne ki deri h. 1 1
1130 Tum.sala.hindu. 0 0
1131 is kutte ki filam dekhne koi bhi schha muslim nhi jayega 0 0
1132 Haaa bhai hoooo gyi party aaj ki 1 1
1135 Aaja bhan k loda apna baap sa baat kr tare maa ka maru bhoxda sala cutta 0 0
1136 Ma teyar houn jnb 1 1
1137 Teri party tere baab ko de na layak 0 0
1138 Tanik kaisen ho 1 1
1141 Mere kuch samaj mai nhi aara m kya kari hoon 1 1
1143 Khan bole tho salman khan, dil tadap ra bhaijaan ke liye 2 2
1144 Bhai apne bola air Maine comment maar dia...Eid Mubarak ho 1 1
1145 Uski party kisey hona bhai 1 1
1146 Pak mei KB hogi party with salman:(1 1
1147 Salman bhai es songs ko movie me bhi dalo plz 1 1
1148 salman is me dono hi maire favourite hai hanuman ji or salman ap ye movie hit nhi super hit hogi 2 2
1149 Kise khushi main party de hai aap ne apne kaha tha aap sare comment padhte ho to ek reply to banta hai 1 1
1150 Ha bhai party kaha h kon si jagha per kiya timing h party ki or kon kon aa raha h party me 1 1
1152 Bhai song utna acha ni tha jitna socha tha but nyc song 1 1
1153 Sala kutta tera movi kon dekhe ga harami 0 0
1154 Salman bhai ji ko Namastey Salaam.ye Eid apki superhit ho.jai bajrangbali. 2 2
1157 very very very very very nice 2 2
1158 Eid Mubarak bajrangi bhijaan 2 2
1159 lov you salman 2 2
1160 Yes bhai boleto fadu song 2 2
1161 Bhai i'm so sad mje yh song site pe nhe mil rhamai kese sunu yh sng 0 0
1165 Osm party salman bhai apka gift 2 2
1166 Koi bhi musalman is ki picture nahi dekhna 0 0
1167 mast h bhai ek no1. 2 2

1168 Ek bAat apki achchi nhi lgI... #Salman_Bhai... mekO pTa h aP bZy h bt vO bAat apKo ptA nhi chl paegi jab tak aP mekO cntct nhi krenge m apni bAat v clear nhi kr paunga 0 0
1169 ramzaan aur eid bahut bahut Mubarak ho i love u 2 2
1171 ye to janab kehte hai ke bena musalmano ke film hit karwakar dekhai ge 1 1
1172 Ramadan Mubarak salu bhi.. 1 1
1173 Lubbbbb uuuu sallllluuuu 2 2
1174 Isski film allah kare flop ho aur age jo bnayega wo sab flop ho aur hogi tujhe kiya pata slman musalman ki dua ki kiya taseer hoti hai 0 0
1175 U jahanami ,,,.hate u ,,waqt hai sudhr jao,,, 0 0
1176 Nice bajrangi bhai. .. 2 2
1177 sab muslim bahi se gujaris h ki duwa karo ki yes ramjaan me yeto h aagle sal ramjaan me ni ho 1 1
1178 allah ke nam ki behurmati ki tune salman 0 0
1179 Kala dajjal hai salman khan 0 0
1180 Jai jai bajrang bali sallu bhai ek number 2 2
1181 shandar song hai! 2 2
1182 Allah ap ko apna haveza aman ma rakha 2 2
1183 Bhai ki chalati he isliye achhe accho ki jalti he......... 2 2
1185 i love u salman vai.i always like u vaijan.khuda hamesha apki saath rahega.. 2 2
1186 yes,boss main toh apki film dekne keliye kis tara se behtab ho rahi hu bata nehi sakti <3 2 2
1187 salman ki band baj jani chahiye is baar 0 0
1188 Yas ye film koy bi math dekho bhajrang bhaijan uter flop hona ye film 0 0
1189 Oo salman sale ku bohat ghamand ayi musalmaano ku bola ki musalman koy bi nahi dekhe tho bi chalegi meri film bolke bola 0 0
1190 Isliye ye salman kutte film koy musalman math dekho 0 0
1191 Sllam bhai jaan EIDmubarakh. 1 1
1192 har saal eid me ana dhoom machake jana 2 2
1194 nice bhai mai jarur aaunga 2 2
1195 Yhe special song 4 eid .....yhe party meri taraf se 2 2
1196 bhak chutiya sala bhosrdi k shar gairat naam ki koi chiz nhi kya re tujme 0 0
1197 aliya zain ko kisi ne dekha h kya ?? 1 1
1198 Slman kya tm waqai musalman ho agr ho tu muslmano ke naam pr dhbba ho 0 0
1199 yes yes yes aap log sahi bolre ho 2 2
1202 Dektha hun kaise release karega movie 0 0
1203 chup baith zaleel tu musalman nahi hai aur tu ek c***tiya hai mada****d 0 0
1204 Iski ma chod do salman ki 0 0
1205 sala musalman ke naam par kalank hai ye sale tune muslim ke liye kiya hi kya hai or jo kar raha hai tu us se bhi jal raha hai 0 0
1206 Kutty sharam tou hai nhi tujh mai zalill insaan 0 0
1207 Enka koi eman dharm nahi hai 0 0
1208 bhosdiwalo bhai ke khilaf kuch galt mat bolo tumhari life jhand krdunga me mc harami log 0 0
1209 Tum kafar ho 0 0
1210 salo yha kyu apni maiya thukwa rahe ho.. sale chutiye bhaag jao.. ni to ga**nd paad dunga 0 0
1211 Tume ka firAlha se daro salman idar maza lelo akhirat me bahut tadpa tadpa ke marega 0 0
1212 ye bhosdiwale bhagne vale nhi bc mc harami sale suwar ki olad 0 0
1215 Eid ki party kahape milu bhai 1 1
1216 bohat acha song h bhai jaan aap bohat jyda smart ho. 2 2
1217 bhaijan eid mubarak 1 1
1218 thnks alot sallu 2 2
1220 Chor hai madarchod salman 0 0
1222 Koi nhe dekhega es tere mivie 0 0
1223 hindu Muslim ke naam pe paisa kama raha hai salman khan.. shame.. 0 0
1224 bhai eid ka gift nhi doge 1 1
1225 agar sachhe muslman ho to eid ke din is kutte ki film mat dekhna. 0 0
1226 Yehi sahi waqt hai jab hamara imaan azmaaya jayega 1 1
1227 Kafir ki aulad mai teri movi nhi dekhunga.... 0 0

1228 waise bhi bete hm bs yaha ana bs kam kiye hai warna hm aksar yahi raha karte the 1 1
1229 Salman bhai mujhe bhi aana hai 1 1
1230 wâ€¦ise tu boli nai ki hai kaha se 1 1
1231 Aap ka bhi insaf honga. yaha nahi to waha honga. . inshallha 1 1
1232 sandhya slman bat nai karega 1 1
1233 Alisha sallu hat ni aane vala the sapne dekhna band karo 1 1
1235 Or me bhi indian hu kon hota he ye jo musalman ko gali de or khud ko musalman kehta he jaheel 0 0
1236 yeh chutye ko leker jaker bathroom mein bandh karo re 0 0
1237 sallu is bst 2 2
1238 Taiyar hai bhai jan 2 2
1239 Salman muzhe mere sawal ka jawab cahiye hum nich to tu kon akher tu bhi muslim h na most reply 0 0
1240 Nich musalman kis ko bola pehle apne aap ko dekh tu aaj jaha par hai un musamano ki vajah se hai. Samga.ha mko dhamki deta hai kon dekhna chahega ye sadi movie 0 0
1241 Weat karna padega 1 1
1242 Ap sache wale being human ho bus apki yahi bt sabse Alag h...ap Surat se kuch Jada smart nhi PR dil se ess pu re Bollywood m sundar ho....saf pakk dil h apke pass Jo etni dou 2 2
1243 Agar ek Muslim hoker iman ke saath gaddari kartha hai use support karnewala usse bada kuffar koyee nahi .pa hale unku dawath do jo us darinda ko support kartha ho kaho ki Islam kya 0 0
1244 bahut pyaar h aap nhi samjhoge 2 2
1245 Abhi easvariya ka bhut utra hi ni he to tumko kaha se milega 1 1
1246 Tu dil hai tu jaan hai..mera salman hai 2 2
1247 o ja gashti k bachey musalman ho k hindu bna hai lanat tere pe 0 0
1248 Teri party tujhe hi mubarak humare liye Allah hi kaafi h 1 1
1250 mai chahthi hu kya ki bajrangi bhaijaan film flap hona pukka 0 0
1251 Bajarangi film flop hogiii salman ki. Maaa bahen hoo gii saaaalaaaa chotiya 0 0
1252 aabki baat 800cr par bajrangi bhaijaan (y) 2 2
1253 awsome song bhaijan... 2 2
1255 yes, lv u sallu bhaijaan... 2 2
1256 Sir ji apni kise s wada keya hi 1 1
1257 Lanat ho bajrangi shaitan par lanti slman 0 0
1258 hume bhi party chahiye 1 1
1260 flop movie kutta salman 0 0
1261 Yes I'm ready u luk so handsome bhagwaan aapko buri nazar se bachaaye 2 2
1263 salman tujhe to alha ka brkt h ..jo tme kharab bole uske ghrme jurjate h (thats means haram khor ka khandan) ok 2 2
1264 Eid kab hain? Jis din Salman ki filim release hoti hai. 1 1
1266 voh toh haiiiiii 1 1
1267 Ied mubarak ho salmaan g.khuda se dua hai,aapki ye movie bhi badi hit ho,aapki last movis ki tarah. 2 2
1268 Apki movie me sbko bolna hai jai siya ram tab eed manegi acchi... 1 1
1269 bhai jaan jaisa koi nhi mai super man bhai jaan ka fan. 2 2
1270 Eid ke din salman apni maiya chudae 0 0
1271 sabki jaan salman khan. 2 2
1272 Nahi to ye mat soch ke tu fanna nahi hoga ye khyal tera jhutha h 0 0
1274 Ha bhai salam 1 1
1275 my sallu bro always mindblowing salam sallu bhai 2 2
1277 iss bar eid ki party bhai ki tarf se........... 1 1
1278 M aapko mis kar rha bhaiya kab aayega big boss plz reply karna 1 1
1280 Salman Khan Teri film flop jayegi tu musalman nhi h tu kafir h 0 0
1281 Don't wary always be happy insaallhatala aap ke film sb se jada box office me kamane wale film hoge 2 2
1282 Amaznggggggg bhaijaan.... Love uuuuh koi kuch ke le mai toh 1 st day 1 show jaunga.... 2 2
1283 salman khan ek achhe enshan hai mere bhai hai 2 2
1284 Ab kis baat bheek mang rha rha hath faila kr eid pr 0 0
1285 eid mubarak bhaijaan in advance 1 1
1287 Party to tab hogi jab life mein pretty hogi and pretty life hogi and pretty hi my beloved wife hogi 1 1
1288 kamina salman yehi bolna chare ho na maqsood bhai 1 1

1289 Pirty toh aap k sath karni thi boss 1 1
1291 ramzaan or eid mubbark ho salman bhai 8863960135 mai aap ka wait 2 2
1292 Hum to hamesa tayaar rehte hain....bas aapki aane ki deri hoti hai.......;-) 1 1
1293 oui salman khan 1 1
1294 aap Kavi hamare orissa me to aao 1 1
1295 salman bhai main apka bahut bada fan hu mujhe apki bahut pasand aata hain. 2 2
1296 i love u saiman ji main apka bohat bada fan hon sir mujhe aap par pora brosha hai aap mujhe back msg karo ge main aap se jab melunga na main apko acting dekhaoonga 2 2
1297 sallu bhai ilove you 2 2
1298 Bhai tyohaar to bhai ka hai tou wo humara hua na 1 1
1299 Salman tum baishram ho pahlai Nam ka Matlab janlo pir samjo gai 0 0
1300 Warna tumhari khairnah ha or waisabi eid to abi nahi hai kuch Dino baad hai 1 1
1303 Sir me apka big big fan hu mujy apse call pe bt karni hai ek bar sir mera contact num 03356507139 Skype I.d a bdul.rehman6731 plzzzz contact meabdul.rehman.sahabto 1 1
1304 Bhai logo sbse bda dharm insaniyat usko follow karo 1 1
1305 Iss bar BJP ka kutta Muslim pe dhabba aanne wala hai eid k din jaha b ye kutta aayega jute mar k bhagao sale k ghar me ghusu 0 0
1306 Ye koun kutta bola 0 0
1307 Yahi to sab log chahte hain ki hum lade aur tum log vohi kr rhe ho 1 1
1309 Ashu Singh tu kya janta hai 1 1
1310 Ashu yar bs kro. . . 1 1
1312 Pk?????......agar bhagwan ko pujne ke liye ek chhoti si murti ki jarurat ni hai to tum bahanchod hizdo ko itne b ade maszid ki kya jarurat hoti hai DHILE LAND KI PAIDAISHO??? 0 0
1313 Abdul raheem bhai iske nhi samajh aayga mu bhat hai sala maners to hai hi nhi isme isko samjhan khde ke aag e been bajana hai 0 0
1314 Ashu Sing tumhari saat howa hoga tum logo ka Mandira mai haaaaaaaaa 1 1
1315 Bhupender Panwar ab tum mojsai q poch rahai ho ka mai Kon ho 1 1
1316 Yar ashu tm had par kr rhe ho. Plz bs kr yr. . .itna nonsense mt bolo 0 0
1317 Ashu Singh tumhari baat hamai samag nahi arahi 1 1
1318 Abhi tak to bada dum tha tum logo ke laudo me??????? achanak se dhile ho gaye???? 0 0
1319 @bhupendra panwar...tu pakistan kyu ni chala jata 5 saal k liye..? 1 1
1320 Abey pakistaan ja kutte teri to hindi bhi sahi ni hai 0 0
1321 aur har bhade ke tattuo se comment karwane wala har SALMAN KHAN musalmaan hota hai 0 0
1322 to tera matlb har bura kam karne vala aadmi musalman hota h Great thinking h bhai my midle finger salute u 0 0
1325 Aur jo chup chup ke bole chala jay vo hi asli hijde ki aulad hota hai ashu kutt 0 0
1326 sonya khan ek hijda musalmaan hai isey apna gender ni pata 0 0
1327 Sonya tum ladka ho ya ldki jaldi btao ek kam h tum se 1 1
1328 Musalmaan kabhi aage se vaar ni karta hamesha hijdo ki tarah pichhe se vaar karta hai,Pakistan ko hi dekh lo Muslim hijdo ka desh Hahahahahahaha 0 0
1329 bhupender panwar hijda musalmaan hai 0 0
1331 Bhupender bro sahi keh rhe ho isse bada chtiya mene apni zindagi mai nhi dekha yahi hai chutiya no1 0 0
1332 Advenced idd mubarak 1 1
1334 Aaj ki parti salman bhai ki eid ki parti 1 1
1335 Uske baad last mai apne liye kuch lelungi 1 1
1336 Gracy Singh or salaman khan jodi ek sath dekhana chahata hu 1 1
1337 Aur sab kaisa chalra hai 1 1
1338 love uuuu shalluuuu 2 2
1339 I hate bajarngi bhai jaan. .. Salman 0 0
1340 Tum mere jaan ho bhai plz 1 bar call ya msg tu h to bola tha like ya comment karo tum padty ho na . 2 2
1341 A harami hai iski bhadwa ki mov mat dekna 0 0
1342 kisi ko harami kahne se pahle apne bare me soco. 1 1
1343 Salman khan Bajrangi kutta maa ka lauda bhenchod 0 0
1345 I think vai app padhtey hongey plzz mai appse milna chata hu 1 1
1346 Koi mere se zyada bda sallu ka fan hai to to lagatar iss film k pahle show dekhega koi hai to add me 1 1

1348 Akhir show me apko kitne din tak dekte rahungi,? 1 1

1350 bhai is kutte ki film mat dekho kyu apna paisa haram jagah le jaa rahe ho 0 0

1351 Hanji paji tiaar aa jooba 1 1

1352 Bhai mujhe aapke sath acting karni hai main bhai aapke ghar ka kaam bhi kar doonga bhai mujhko mumbai le
chalo bhai bhai plz bhai 1 1

1354 tumne muje msg kya tha plz batao please 1 1

1356 pure bachio ki jaan lena hai kya aise pics dekha k 1 1

1357 main ye pic dekhne ke liye taiyar hu salman bhai 1 1

1358 salmaan sr...... Aap ne khud kaha k aap hr cmments khud read krte ho..... so kabhi reply ku nahi krte 1 1

1359 love uuu shallluuuuu 2 2

1360 m sallu ko etna love krta hu jisko nap ne k liye avi tk kuch ni bna 2 2

1361 Maat dekho salman ke bajrange bhai jaan movies 2 ? Salman khan is not a muslim he is a hindu becous Allah s
ey SAAB SE BAA GUNAA HAIN SHIRK TO Salman kahn shirk kar raha hain 3 KURAN KA farman haain saab s
e bara gunaa Shirk hain 0 0

1362 Hum ka bole is kamine salman ke bare me bolna to bahut kuch hai I heat salman khan 0 0

1364 apko salman bhai ki kasam mujhe add kijiya 1 1

1365 Meri bhai jaan salman khan ilove you 2 2

1366 bhai mae tho tayar hae 2 2

1367 bhai mai tu kabse tayyar sath me eid manege bhai roza the aj 1 1

1368 Kya kare ho 1 1

1369 Itni saari qurbani dene k baad fir bhi mai galat hoon 1 1

1370 Tumne bhi kabhi saath nhi diyaa to mai akhir galat ho he gai cograss 1 1

1372 Jaise tum apne waade k pakke ho waise mai bhi apne waade Ki pakki hoon 1 1

1374 aslamo alaikum bhai 1 1

1375 very dhamaka song pichli baar jumme ki raat &iss baar party ki raat wow..party aapki taraf se iss party mehi ki
ck hai... 2 2

1377 Isna Muslim ko chalang kiya ha iska movie koi. Mat dekhna agr tum sab Muslim hoga to nhi dekho ge 0 0

1378 sallu bhai rocks 2 2

1379 Bhai jaan k se hai ap ghar wale k se hai ya film bajrangi bhaijaan qameebi ho gayi mujhe ko i d me add krna ha
apko Ramzan ap sub ko Mubarak ho. Saad Khan pakistan Karachi . 2 2

1380 BHAI boht acha hy bus aj ki jagah pr eid hona chahye thaeid ki prty meri trafcse .....Jai hoooo bhaii 2 2

1381 Bahut bahut badhai ho bhai jaan aapki aane wali flim me hum logo ka sabka subh kamnaye.he 2 2

1382 mai salmaan bhai ka baho.......t... bada fan hoon ... 2 2

1385 Tayyr,hai sllu bhaijaan 1 1

1386 sem 2u bhai 1 1

1387 Nice song aaj ki patry meri tarf se 2 2

1388 insa-allah ye sare record torogi 2 2

1389 apko bhi mubarak 2 2

1390 ab apko nice,gud,handsome etc bolke kya faida....Ei sab bohot chota word hai ap k liye.....ap mera fav actres h
o pls Cl me on this nmbr....919593871431...<<<Agar ho sake to>>>..Pls...'" 2 2

1391 koi pasand ni karta teri film ko or band kar ye tamasa 0 0

1392 salman kaha good. film bajrahgi bhaijaan oldbasyt walkam 2 2

1393 Aba es photographer sa achi photo to ma khich la ta hu sala q tera 2 maru ga chitiya sa la tera baap ko ati hai p
hotography ... 0 0

1394 Advance mai eid mobarak bhai 1 1

1395 Salman khan am exited 4 ur n3w film #bajrangi #bhaijan 2 2

1397 gulmira ne kya likha h. . . . 1 1

1399 Salaam bhai aap ye accha kaam nahi karre 0 0

1402 bhai tumhara ghar or ghar ke share log islaam ke naam par kaala dhappa ho islaam se judi koi bat nahi he tuma
in 0 0

1405 Kye hal han Salman Bai 1 1

1406 khuni saale chal jail ja 0 0

1407 eid musalmaan ki hoti hai 1 1

1408 hi salman Bajrangi bhaijan 1 1

1409 bhai party ke liye abke sab frnd tayar hain...!! 1 1

1410 party kha pai hoygi bhai 1 1
1411 allah kare is khabis ki movie flop ho 0 0
1412 Eid mubark ho bhai jaan advance mai it's emerjancy plz 1 1
1414 hamaare duvaa aapke saath he bhijaan ham aapke leye kuch bhe kr sakte he 2 2
1416 Aaj ki party bhai ke taraf se 1 1
1417 Film release bhi hogiApki taraf se party bhi hogiOr aapki zholi bhi bhar jayegiTension nahi lene ka bhai Hum sab aap k sath haiWish you all the best 2 2
1418 Salman bhai ap mujhe bhot pasand ho 2 2
1419 Dukkar h salman suwar kahi ka 0 0
1420 Mr. salman ajkal aap k setare gardish mai aapko sarf 8 hazar comment aate hai oh ho 2 2
1421 bhai jan kya baaaat haiiiiiiiiiiiiiiiiiiiiiiiiiiiiiiiiii. 2 2
1422 Aaj ki iftar party meri taraf se invition hai bhaijan apko pure pariwar ke sath 1 1
1423 Party with salman & bebo kon miss krega 1 1
1424 Wah bhai wah 2 2
1427 Bole to ekdum hit apne sallu bhai ki picture jhakaaas 2 2
1428 yaar bhai jaisa to aur koi paida hi nahi ho sakta I LOVE YOU BHAI 2 2
1430 hi salman vai eid mubarak 1 1
1432 apki bht bht bht bht bari fan hon pakistan se 2 2
1434 kya ye dawat garib or yateemo k liye bhi hai 1 1
1435 @lwayz ready 4 uh salman sir.... 1 1
1437 yaaahi bhut bari party he ap ki taraaaf se 1 1
1438 eid mumbar ho aap ko.. 1 1
1439 aaj ki party meri taraf se salman bhai 1 1
1440 vry vry beautiful song. .. 2 2
1441 Selfie le beta jb marja0ge jb Qaber m ja0ge tb Allah tje dakhega ghtya h0 tm really 0 0
1442 Bhai ied mubaraq 1 1
1443 Bohot accha hai . Aaj ki party mari taraf se , sab ko bolo utre flak se . Nice 2 2
1445 Kaise hai bhai aap achha rhiyega to hum v rhenge vRna aap jante hai 1 1
1447 Jo b ho par main aap se bhuuuuuut pyaar karty ho salman 2 2
1448 Salman khan Teri maa ki chuth aur Teri bahan ki chood 0 0
1449 Yeh kaun boodeh ke peechhe padi hai bina makeup ke dekhle bhaag jaogi. 0 0
1450 Bhai aapki movie hum hit karenge koi kare ya nare 2 2
1451 Bhaijan eid Mubarak Aap ko pyaar karty ho 2 2
1452 Bhaijan tumara fon nombar chaie do na eid Mubarak bolne ka 2 2
1453 Salam lekum bhai jaan ramdan mubarak ho aap ko 2 2
1454 Awa ge jaroor 1 1
1455 chicken song iw awesome bhai 2 2
1457 Kyun bhai Aj kyun nahi Kal kare so Aaj KAR... 1 1
1458 wowwwwwww omg aaj kyu nahi ....love u salman 2 2
1459 Kay baat hai bhai jaan.... 2 2
1460 main to ab jaa hi nhi rha aur khin kal tk...yhi rahunga 1 1
1461 hum intazar karenge 1 1
1462 Salllu tumhe pta bhi h ki tum iss red pathani kurte me kya jabardast lag rahe ho....koi bhi ladki mar jaaye tumh are liye to...ummmmaaahhhh? 2 2
1464 aap ki EID ki party me hum ainge bhai 2 2
1465 Lov ew xo much salman 2 2
1467 Han bhaijaan jaisa aap kaho..best of luck for bajrangi bhai 2 2
1468 super cool cool bro 2 2
1469 super jodi kreena sallu 2 2
1470 oh my god ,i cnt belive...... i'll be thr........pls be my frnd salman...$$$$$$ 2 2
1471 Super duper hit hai bhai ... 2 2
1473 Teri maa ka 0 0
1474 nice pic bajarangi bhaiya 2 2
1475 Iam waiting salman bhai 1 1
1477 Vadiaa veer ji 1 1

1478 I don't like salman khan madharchod randy ka ladka harami kafir 0 0
1480 aadab salman ji 1 1
1481 Sure i ll 3:30 Ko kya me toh 2:30 Ko hi facebook me rahungi 1 1
1482 abe chutiye kabhi namaz bhi pad liya kar 0 0
1483 Salman mere jaan muuuhhhhhhaaa 2 2
1485 Goodluck for bajarangi bhaijaan 2 2
1486 Musalman log khilaaf hogaye iske saari ijjat dho dega kya is pic mei 0 0
1487 theek hai mujhe to lagta hai main ye film dekh chuuka hu sabhi loog iss film ko apne aap se relate karenge 1 1
1488 salman bhaijaan eid mubarak aapko ho 1 1
1490 Aap sabki madad karte he meri friend ki help kar dije pliz 1 1
1492 Salman ab tak to hum aapke bohut bade fan the lekin ab nahi ..Kyon k aapne musalmaan bhaiyon ka bohut dil dukhaya hai I hate u.. 0 0
1493 Assalamu alykum bhaijaan Eid mubarak ho bhai or aap bahut ache insaan ho good luck 2 2
1494 welcome *"*'*'*'*'*'*bajrangibhai jaan*'*'*'*'*'*'*"*'*'*superhit movie*"*"*"*'*'*'*:welcome fance party*"*'-' *'-'*'*"*'*superr*'*'*'*'*'*'*'*:*duper*"*'*'*'*'*'*best*'*'*'*'*'*'*'*'*"*:very nice*"*'*'*:*'*bajarangi bhai *"*'*'*"*'*' *'*bajarangi bhai *'*"*"*"*'*'*'*'*bajarangi bhai*"*"*'*'*':'*:*'*hit*'*"*'*'*:hit*'*"*'*'*'*'*'*'*hit*'*'*"*'*'*'*super actor&actress*"*'*'bajrangibhai*"*'*'kareena *'*'*'*'*best *"*"*"*'*'*"*'*watch movie later-id-ul-fittar*"*'*'*'with*" *"*'*'*sallu*"*"*'*'*':'kareena *"*'*'*'*'*bajarangi bhai jaan *"*"*'*'*superstar*"*'*'*':: 2 2
1495 selfie le lo bhai ke saath 1 1
1496 Salman Khan sir koi kuch b bole hume koi. Frk nhi pdta. Ye sb ek chal he kisiki bt i lv u sr god bless u hm to dekhege movie or dikhayege b #BBthiseid becouse ye mere salman sir ki movie ka swal hai. 2 2
1497 bhaijaan sultan m negtive role kiska h 1 1
1498 Salman bhaijan EID MOBARAK. 1 1
1499 awxom actor sallu bro 2 2
1501 Salman bhai aap ke khilaf kya horaha hai 2 2
1502 we love u very much bhaijann(bollywood ke sann india ke jaan sub ke bhaijaan SALMAN KHAN) !!!!!!!!!!!!! 2 2
1503 Bhai musalman hone ka to faraz nibha deta brother 0 0
1504 nic song . bhi jan 2 2
1506 Bhosrdi k kya bolta hai musalmaan nich hai 0 0
1509 Kute begherat khabees benamos kafir k bache teri itni himat 0 0
1510 Ji salman bhai.bilqul rhenge..aapke liye :* :) 2 2
1512 mere bhai jaan nhi ho ap mere to super star ho ap 2 2
1514 Salman khan rock . 2 2
1515 m musalman ladki se shadi krna chata hu mare age 25 hai 9818463635 plz col me shadi pka karuga ok plz col me 1 1
1516 jai shri Ram Jai shri ram 2 2
1518 Mene kia qusor kia he salman g jo mjh se bat nai kerte reply nai kerte 1 1
1519 m musalman ladki se shadi krna chahta hu mare age 25 hai plz col me only ladki 09818463635 from (hp) 1 1
1523 Kyu aisa kya salman bhai 1 1
1524 bhai jaan nice 2 2
1525 koi kuch bhe khe ap hit ho 2 2
1526 bus ek sawal ka jawad dijye aap mujhse kab milenge 1 1
1527 Bajrangi bhaijaan 300 par karega yeh mere wada hai 2 2
1528 Bakwas actor ki bakwaas film 0 0
1530 Masha Allah kisi ke nazar na lage 2 2
1531 Aap ki new film ka mein besabri se intzar kar raha hu please jab film release hogi to website post kar dena ta jo ham aap ki super duper hit film dowload kar sake 2 2
1532 okk bhai jaan...:) 1 1
1533 love yu bhai jaan,,,,,I like yu,,,,,I love yu bhai jaan,,,,,,nice song so sweet handsome parsnalti 2 2
1534 Abe ja be zhandu balm Tere jaison keliye kon waqt nikalega 0 0
1535 kiya koi bata sakta hai india Time 3:30 pm UK time kiya hota hai?? 1 1
1536 hamare sallu bhai h 1 1
1538 Tum musalman nahi hi 0 0
1539 Heard k Sallu bhai ko musalmaano ki ab koi zarurath nahi rahi iz diz true??? 1 1

1540 Jo sachcha musalman hoga vo iski movie nahi dekhega bhai kyu k isne muslamano ko chalange kiya hai so ma i to nahi dekhunga jiski jo marzi bhai 0 0
1541 Ok sallu miy 1 1
1543 Bhaijaan muze aapke sath picture click karni hai.......love u bhaijaan..... 2 2
1544 Assalamu alaikum bhaijan 1 1
1545 Bhaijan watsaap pe aapke bare ma jo chal raha ha wo jhoot ha na.plz reply must 1 1
1546 Salman is godfather is bollywood 2 2
1547 mast hai bhai jaan 2 2
1548 Tre bap ne bnai hoti tb b flop nhi hoti ssale, 0 0
1549 Kya guarantee hai ki sab questions ka answer aap hi denge???koi dusra bhi toh de sakta hai??? 1 1
1550 Akib Jawad TU SULTAN KO FLOP KARAYEGA TU ABE JA CHUTIYA JA TU YHA SE NHI TO THOK DUNGA 0 0
1551 Kya bhai hum jab Bussy ho tab hi aap online honge..... plz hamara bhi socho..... night me sab free hote he. 1 1
1553 Salame walekum .bajrangi bhaijan 1 1
1554 Facebook jaisa Accha chis me ye sab faltu items kyu ata hai kya pata????? Dirty fellow. ..... idiot kahika...... m u dekh lia bash aj ka din e sala kharap jayega..... 0 0
1555 Salam Nadia Khan frm salman khan 1 1
1557 luv u salman muhhhh 2 2
1558 Harami kanjer insaan 0 0
1559 Asaalam aalay kumm Asaalam axelent bhai jan (y) 2 2
1560 Salman bhai mery comnt ka reply krna plzzzzzzzzz 1 1
1561 haye Sallu....tussi gr8 ho........ 2 2
1562 koi dekhe na dekhe hum toh apki iss film ko toh zarur jayenge 2 2
1563 Nic salman bai 2 2
1564 bhai....hum tumhare bareme sab jaante hai..kya question aur kya answer....we love you.....abto bss movie releas e ho...usi din jashn hoga....Eid mubarak in advance.......... 2 2
1565 Bhai aap toh bolly wood ke badshah ho """"""aur aap humare dil k kareb ho love u bhai jaan 2 2
1566 Gret is gret khan salman 2 2
1567 kya baat h.....bhai 2 2
1568 ofcourse salman isme sochna kaisa.. 1 1
1569 sallu bhai..........ed ki party akele ....hi hmko bhul gye 1 1
1570 Iove you sulman bhai 2 2
1571 Muslim brothers sisters plz salman khan ko unlike karo aor lanat bhejo salman khan pr salman khan hindu higa ya hai islo ezzat mat do bezaty karo Allah ki lanat hu is bodhy baba par 0 0
1572 sir im upset becaz apki film pakistan mai eid ke ek week bd release ho gi sir ramzan mai sirf allah miyan se ap ki kushi mangi hai hamari dua qubol ho jaye ap hamesha kamiyab hun.im samaira from pakistan i always woh aye ni and support u. 2 2
1574 BEHNCHOD Sahil Rehman Roy. BAAP SE FANDA KAREGA TUJHE TERE INBOX ME THOKUGA 0 0
1575 Salman bhai aap to folp hooo jiii 0 0
1578 Bhai jakaass bhai insallha super hit. ... 2 2
1579 salman bhaijaan...ap ki new movie mein shoot mee mai 1 1
1580 Yasmeen Khan. Dimag THIKANE lga kr comnt kr 0 0
1581 Sallu bhai aapka no.... chaye 1 1
1584 True love to aisa hota hai jo kabhi uske aakho m aasu nhi dekh skta ho marna pitna to bhut dur ki baat h 1 1
1585 Or uske khe bina uski baat samajh jate h 1 1
1586 bajrangi bhaijan eid mubarak in advanc or apki movie 450cr ke par jaye kyuki....you are grate and smart hero o f bollywood ke 2 2
1587 kal kya h.....b'dy gift doge kya mera? <3 :D 1 1
1588 Salman bhai mafi Maglo abi time hi..Muslim bina hit karuga Bajrangi Bhaijan ko...kesi karo gi bhai...apka frns group he hi Muslim 0 0
1589 Abe selman Khan muslman ke name per klneq hae ise jeene ka koi heq nahi hae 0 0
1590 bhaijaan mera reply jarur dena 1 1
1591 Jai shri ram ....coming sooooon bjrngi bhai jan 1 1
1592 Madar chord maa chuda Mumbai mai Hyd dheekha to maacood data tari 0 0
1594 Pakistan main be apko log bht pasand krta hn bhai jan..... 2 2

1595 aapki film kaisi bhi q na ho humko 1st day 1st show dekni hi hai. 2 2
1596 bhai ap muslim bina kesi hit karoge ye films. . 0 0
1597 tera film se br nhi chalne wala h bajrangi 0 0
1599 Ja kutte Teri movie koi nhi dekhega haram khor 0 0
1600 Kese ho bhai aap 1 1
1601 Love u mere swttuuuu... Muuaah muaah muah .. Tum ho toh me hoo... Ek pal milneki chahat se umid banih, au r umid se jindahoo me... Tere lye bas tu hi tu hai... 2 2
1602 Yeh movie koi muslim nhi dekhega kyun k yeh movie chalne wala nhi h 0 0
1603 SULTAN ALI KHAN SE 1 1
1604 Salman bhai aap kamal ho 2 2
1605 Ek nai kahani dekhne ko behad utsuk hai.love you sallu bhai.bhaijaan. 2 2
1606 Kis time kal? 1 1
1607 1 no bhau 2 2
1608 hame koi like kijiye hum bajrangi se kam nahi hai 1 1
1609 Sir kisko rply mat dena 1 1
1610 salam bhai app ke Roza kasa ja raha ap ka 1 1
1611 Kuc nahi parthe hai bhai salam ka zabab bi nahi date hai 1 1
1612 bhai Tu apni jaaan hai :* 1 1
1613 Iam waiting bhaijaan 1 1
1614 U owsum Salman bhai 2 2
1616 Bhaijan aap ki sari film supar hoti hai or. Main aap ki sari film dekhti hun kash main ek baar aap se mil pati 2 2
1617 ye harami iski kya aukat hai salamn ki mere samne iske pas jitna paisa hai utna to mere kutte ko khana ata hai 0 0
1618 Bhai is bar Eid aap ka chera dekh kar Ho gi .kab 17 tarikh aaye iam waiting 2 2
1619 Salman don't worry faltu ki abha se apke fan ko fark nhi padega movie mast chalegi 2 2
1620 tere muslman hun te lanat hai 0 0
1621 Eid mobarak bhaijaan kyse ho serkhan kab aega bhai 2 2
1622 Bhai Tere samne koi nahi bhai u r gret bhai 2 2
1623 Eid mubark ho salman jii I love you salman.mai apse ek bar milna chahti hu salman jiiii meri yhi khwaish h sal man jiiiii 2 2
1624 Bhai ji aap to kamaal ho 2 2
1625 pakistan k fans kia kren zara ye bhi btaye bhai? 1 1
1626 . Salman bhai apni life khud Kharab kr rahe ho ase krke alla hafiz 0 0
1627 Ekela boycot kar awaiz tu kamyaab ho jayega paaka sirf tu mat ja 1 1
1628 bhai jaan aapki movie hit h ...ham aap ka sath h inshallah 2 2
1630 Salman aaja ghar mere eid par 1 1
1631 Yar ji karrha h 1 1
1632 eid mubarak lakin es br eddi bhi chaiyea 1 1
1634 Huuurrrrrrrr salman khan teri aisi ki tesi aur teri movie ki bhi 0 0
1635 Bhagvan ka kam karna sub se mukya darm hai. 1 1
1637 Salman mai aapko bhai jaan manta hun lekin mai cahta hun yah film na het ho kaunki yah musalmano ka sawal hai 1 1
1638 Salmam khan teri film flop hogi yaad rakh le tu 0 0
1640 kyu movie me kuch glt dikhaya h kya 1 1
1642 Bhaijaan...app tnsn free raho... 1 1
1643 Sj Soriful kya bola teku itna hi pasand salman to tere ghar me rakh le usku 0 0
1644 ye sab afwa hai bhai aisa na bolo koi 1 1
1645 mobarak ied mobarak ?? 1 1
1648 ab chalo yar sone...aaj #juma h bhailog ....namaz padhni h 1 1
1649 Shahrukh Gouri galat news hai woh..... dont believe....usne aisa nahi kaha 1 1
1650 Zara pucho Salman se k kitna #Roza raha 1 1
1651 Ahana Khan ignore thm thy wil never understand..... kuch bolo toh gaaliyan dena shuru kardenge jo hame isla m sikaate hai 1 1
1652 Aur Salman bhai apki hum apka movies nhi dekhne jainge,,, Q k apnea ussme wahiyaad bhagwan ka chalissa g aya ha jiska wajse Muslim ko sarminda hona padege,,,,, Pura mehena yani month ,, roza Namaz padne k baad ye sun

nege,,, kya bhai apko koi aur movies nhi mila kya 0 0

1653 Aagar salman sai hai to ek martaba media me saaf saaf bolde ke mene esa nahi bola ....simpal... 1 1

1655 mamu aap mera agar msg recevie kar to ple haan ya na mamu ple good morning. 1 1

1656 salman bhai, meri ek frnd hai..........she....lives......in......sirsi.....n .....aaki.....bauhat.....badi.....fan.....hai.....i.....dont.....know.....uske.....number....pe......wo....reply.....nhi.....kar.....rhi......n......wo......mujhe......seriously..... lyk.....karti.....hai......n.....main....bhi....agar.....mera.....ye.....comment.....aap....tak....pauhache.....den.....sirf...aap....hi...agar.....fb.....pe....usko....bol.....de.....help...me....i....don't..... know.....main.....jo.....likh.....raha.....hu....wo...sahi....hai.....ya....galat.....but....bhagwaan...kasam.....dil....se.....n....honestly.....likh.....raha.....hu.....agar....aap....meri.....help....kar....de....den....aap....jo....bologe.....wo....main....krunga.....pls....pls......plss......Sry..bhai....aap soch rhe hoge ki aap mujhe trust kyu kare den pls mera contact 1 1

1657 bhaijaan aslam valekum 1 1

1658 Sab ko Jana hai uper ok is liye apna dakho salman nahi aaya ga ok aur vo to dog hai na Muslim hai na Hindu hai na vo insan hai sif dog hai ok bye bye 1 1

1659 ap ka sb film dekta hu, me v bajrnag Bali ka bhkt hu, jai bajrang bali ji, ap film nam se suprhit ho gya, nam se, hi ap ke dusmn bahg jayga, 2 2

1660 Sala khutta bajrangi bana hova kuch to sharm kr? 0 0

1661 slam malikam salman 1 1

1662 hi.... salman bhai my self mayur and m a litill singer or meri rqst hai aapse pls mujhe bhi ek chance dijiye bhai aapke liye gaane ka......plllssssss bhai 1 1

1663 600.0000000 se paar gayi bhaijaan 1 1

1665 Nice bhaiYOU ARE THE BEST BEST BEST BEST BEST BEST BEST BEST 2 2

1666 goodness pic bhai aap bhut ache or smart ho..or m aapka sabse bda fan hu bhai 2 2

1667 ramzan mubarak ho bhaijaan 1 1

1668 masaallah bhaijaan ..supar dupar hit bajrangi bhaijaan 2 2

1669 Wah bhai jaan bahut smart lag rahe ho 2 2

1670 love salo bhai 2 2

1671 Tumare ghar pe 1 1

1672 Ek kaam karo baam laga k maalish karo fir ek kapada lo us mai akhe nanamk rakh kar tave p halkaa garam kar k back par seikh do inshallah back pain thick hojaeyga 1 1

1673 Madhar chod sale kutte ki aoulad salman khan ki ma ka bhosha 0 0

1674 Mami ko bolnaa WO gharelu nusk ache se. Kar degi 1 1

1675 hilo kiya aap happy ho 1 1

1676 Bhaiii apko kisi ki jaan bacha hai siriyasli aap agar ye sms padh le to reply karna 1 1

1677 Bhi bol to ek nambar bollywood ka raja 2 2

1678 main aapki bahu bahut bahut bahut bahut badi fan hu 2 2

1679 Lagta he gar wapsi ho rhi he bajrangi bhi bhaijaan lagane lag gaye 1 1

1680 Aaj ki party meri taraf se bhai jaan 1 1

1681 Haste raho haste raho 2 2

1682 muhhhhhhhhhhhhh meri jaan ....love u sallu.... <3 <3 2 2

1683 Only one hero jo sab ke dil me rahate h wo h salman bhai bajrangi bhaijaan unko dekh kar hi sabki dharkan chalne lagti h i love salman bhai 2 2

1684 bhaijaan app itnai deer bad kyu promotion krte ho 1 1

1685 Aap film me bajarangi hai ki Bhai jaan 1 1

1686 Inshallah bhai bajrangi bhaijaan will hit 2 2

1688 Nicere vai sallu 2 2

1689 my favirat hero salman khan 2 2

1690 yeh dua hai meri ke aap hamesa khus rhe 2 2

1691 lv u salmn 2 2

1692 Sher kabhi darta nahi aur mera bhai sher hai love you bhai jaan 2 2

1694 3.30 pm se bhai video chat karenge yaha pe sb question taiyar rakhna apne 1 1

1695 bhaijaan mere Questions... aap sabse jyada kise chahte ho???? 1 1

1696 3.30 cant wait........ 1 1

1698 sir ji itna wait q karwa rhe ho 1 1

1700 kutte kutte kutte.... teri movie flop hogi 0 0

1701 Aaj ki party mere taraf se.. 1 1

1702 bhaijaan bas ek kar do 1 1
1704 Movie ke postar fardege hum sale kutte ki 0 0
1705 i love u salman khan iam u r big big fan love u u u u u sooooo much sallu,,i miss u very day 2 2
1706 Nice salman ji 2 2
1707 Oho tumare ankha tumare nak tumare lips kasm sa mar hi daloga? 2 2
1708 Salman you r being human love you bby..... mujhe tumara pyar chiya or kuch nhi... love you?????? 2 2
1709 pandey ji always super hit mmmuuaaa 2 2
1710 India's superheroSALMAN KHAN. N ourJAAN 2 2
1711 Hello salman baijaan main aap ka bhot bda fan hu Maine lagbag sari pictures dekhi hai 2 2
1712 Apne glat kiya humlogo k sath 0 0
1713 Aaj ka bolke kal par taal diya.. Salman bhai aapse aisi ummed na thi 0 0
1714 bhai 4 baj gye 1 1
1715 kyaaa yaaar ...umidd thi k aaj kuch had tak to sapna sch hoga leki pandey ji umid tod di naa.. 0 0
1716 bollywood ki saan hindustan ki jaan 1 n only salman khan love u salman 2 2
1717 Eid ka zaakat fitra lene kab aa rha hai 1 1
1718 uff mashaalllah killing look bhaijaan 2 2
1719 Asak...aap apni maa se aur apne bhaiyon se bahot pyar kerte hain bahot acha lagta hai 2 2
1720 Bhaijaan aap bahot achhe insaan hai jo na samjhe wo nadaan hai aap to bajrangi bhaijaan hai ramjan eid mubark bhaijaan 2 2
1721 Kitne roze hue salman bhai 1 1
1723 salam wah likhum bhaijaan 1 1
1724 Supar dupar hit sab rekod tutega aesi dil se duaa 2 2
1725 meney suna ap kiynup hohay yes no pls ansr 1 1
1726 Masha allah bahot ache lgre ho buri nazar se bachaye allah aapko. 2 2
1727 kafir ki film dekhna haram hai 0 0
1728 Hi salman bhai kisa hai 1 1
1729 are zarur sallu kamaal krte ho 2 2
1731 sab ka bhai hamara sallu bhai 2 2
1733 Bhai bhai somabhai bohemia, 1 1
1734 Aaj ki party meri trf se this eid 1 1
1735 assala muwalekum vhai zahan 1 1
1736 Iss bar teri ni chalegi 0 0
1737 waa sallu bhai 2 2
1738 salman khan ilove u wii u married me 2 2
1739 music thik nahi hai 0 0
1740 asslamu alaikum salman bhai ladhkoyo ko hnsm nai hne se nt like 0 0
1741 O Ho bhai kal kaise niklega 1 1
1743 salman bhai look to mast hai par choor lag rahe ho 1 1
1744 5.25 paise diya 1 1
1745 Ye to confirm hit nhi jayegi kyunki koyi musalman bhai iski picture nhi dekhega 0 0
1748 ye hai dhasu look..../ilu viii 2 2
1750 Kya bat hai bhai 2 2
1751 Bhai cross 700cr pts increse 2 2
1753 Nice imeg bro 2 2
1757 es yearss ki super doper film bajrangi bhaijan 2 2
1759 Bajrangi bhaiJan aap se kb mulakaat hogi isi janam ya agle janam Bajarang bali kab chahte hai 1 1
1760 My bhai my hero salman bhaihum salman bhai ke bahut bare bhakt h mar jayenge lekin jhut nahi bolenge 1 1
1761 salman bhai aap panch time ka namaaz padiye 1 1
1762 Sale kafir salam mat kar ... namaste kar 0 0
1763 KASH HAM MIL PATE 1 1
1764 super duper hit hoga bajrangi bhaijaan ye meri dua hai. 2 2
1765 Me aapki pic ke liye dua karugi ke soper doper hits jye .. 2 2
1766 Hamari or se ED mubark ho aapko or aapki fhemli ko.. 2 2
1767 Adab adab meri jaan Mashallallah bhuut pyaare lag rahe ho meri jaan mera salman 2 2
1769 Bolo naa kyaa karo salman ? 1 1

1770 Please reply karo naa mujhe please salman ji 1 1
1771 mi ap ki movie ka bhut time se wait kr rhi ccccc 1 1
1772 salman agr tun mery samnay aa gea to ma teri film bna dunga bhenchod apny ap ko tun smjhta kia ha 0 0
1773 Meri jaan mera salman 2 2
1774 Main aap py qurban 1 1
1775 Teri maa ki chuth. .film riles ka raha hai bahan chood. Eid .par kiyu. Nikal raha hai 0 0
1776 Bahan chood teri 0 0
1777 Hello salman jee, , Eid mubarak 1 1
1782 Aur eidi main aap chahye 1 1
1783 Pakistan kyo jayega salman 1 1
1784 Tnx bhai jan leliya 1 1
1785 Suprab my jaan salman khan 2 2
1786 fir bye kyo 1 1
1787 mast hai vai 2 2
1789 Bhaijaan aapka song humesha hit hi hota hai 2 2
1790 soo sweeet song 2 2
1791 lovly &nice song ..salman. 2 2
1792 Priyanka Kunwar beautiful beautiful beautiful ! kasto ramrailo gana. Love salman, can't wait to see bhaijaan?? 2 2
1793 just awesome bonding pawan-munni 2 2
1794 Superb Song & Supper-Dupper Hit Movie jb koi nek kaam krne niklta h to khuda bhi uska sath deta h apne to 1 chhoti bachchi ko uske apno s milana chaha isme to sari Kaynat apka sth degi 2 2
1795 Kamaal karte ho pandey jii ? ? ? 1 1
1796 butiful song evr i fel in luv wiv dis song 2 2
1797 Jo abi ayy han un ko bata dun aj online chat ho gi idar okay 3:30pm par 1 1
1798 Aj ki party ...bhabi ki taraf se sallu bhai ...?? 1 1
1799 Waoooooo, soo beautiful 2 2
1800 Raksha Shinde SALMAN insha Allah zaror help karenge... 1 1
1801 hum koi bhi bhai ka movie nahi chodte hain eid mobarak bhai.... 2 2
1802 3:30 par bat ho gi sab se allah hafiz 1 1
1803 Bhaiii ...greatttt<3<3<3 amazinggg wonderfullll 2 2
1805 Wah good video salman bhai 100 like deta ho like like like like like like like like like like like like like like like like like like like like Luke 2 2
1806 waise time waste nhi kartey ho timing kain pake ho 1 1
1807 ekdum lapak lia bhai. ap de or hum na le. 1 1
1808 bohut khub,,specially salman kahan.... 2 2
1809 Apne dil jeet lia bhai...Allah bless you 2 2
1810 Gud luk bro 2 2
1811 Wah. kya kehne! 2 2
1812 Aapne aisa q kha salman ki aap bina muslmn ki apni pix hit kra doge 0 0
1813 inki film inkey naam pay chalti hai naam hi bahut hai superhit karaney kay liye 1 1
1815 Salman bhai ka sirf naam hi kafi hai film apen Aap blockbuster ho jati hai 2 2
1816 Hllo salman bhai kase h aap 1 1
1817 mujhe nhi pta aap fb par hai ya nhi fake id bhi ho sakti hai lekin aap bhaijaan hai or martay dum tak mere bhaijaan he rahenge kyunki mein aapse behad pyar karta hu. 2 2
1818 very nice salman bai beautifull song 2 2
1819 Salman khan bhai jann nice ha very nice salman bhai beautiful songs 2 2
1820 all song rocking han bahi 2 2
1821 Faad bhai faad ....att kara ti . Sirra acting kiti a . Maza aa gya . #bhai to jaan hai 2 2
1823 Awesome track. ..Dil mey uttar gya yaar 2 2
1825 ap b piyray ap ka gana b piya ho ga am frm pakistan 1 1
1829 Waoo.salman nice yar 2 2
1830 Ye bv film bohot hit hoga jaan 2 2
1831 Salman ji aapne iss bar koi song nahi gaya mujhe wait tha k iss bar b aap ke lovely voice main koi song gaye ... ...I love your voice ....hangover is my all time favourite song abe tak main vo song sunte ho I just love that song 2 2

1832 Jai shri krishna bhagwaan ji apko hamesha khush rakhe 2 2
1833 hur hur dabangg dabangg dabangg ..... Bajrangi bhaijan 2 2
1834 Bhaijaan to gud Bhai aap bahut achche insan he 2 2
1835 grat indian army & salman vai. 2 2
1836 Pak foje zinda bad <3 2 2
1837 Bhai ye kesi kesi afwahe ud rhi he apke bare mai ..sach he ya jhoot 1 1
1838 salman sir aap kider ho plz message karo na mujhe plz 1 1
1839 Bada hoke fir aayega... Infiltrate karne.. and goli khane.. 1 1
1840 sir aap ki movies ka asar hai 1 1
1841 not bad kya ho rha hai kha ho 1 1
1842 Mulle ne ek mulle ko bacha liya... 1 1
1843 kashmir india ka tha or rhega saale pakistanio chup 1 1
1844 Jinko mara ja raha ha waha wo bhe pakistani ha :p 1 1
1845 Pata wata hota nhi ajate hain bheek mangne 1 1
1846 Bande matramjai hindbharat mata ki jaind indai is greati love my india;-> 2 2
1848 $@lute ! Uss jausbay koo jo Hindustan key Dil main bustaa hain. 2 2
1849 Salman khan madharchod randy ka ladka harami kafir 0 0
1850 Isi bahane promote bhi kar lo 1 1
1852 Insaniyat nd pyar sbse phle yaad rhta h 1 1
1853 hindhustan is my foot pakistaniyo ko galiya kyun dete ap sb kyun pakistniyo ne ap sbki bhene churi hai kiya 0
0
1855 Chalo Indian army nay kisi ko tu chora.warna in ka bus chaley tu is buchey ko bhi terrorist from Pakistan kah
Kay qaid ker laye 0 0
1860 strange..but bht acha kia .... realy feeling happy 2 2
1862 Love u bhai jiii salman khan 2 2
1864 aise neak kam karne se shayad hi unke dilo mai hamare desh ke liye pyaar bade 1 1
1866 Pyar mangi to Jaan dengi,milk mango to kher dengi,agaar Kashmir mangi to chir dengi. 1 1
1867 Salute to indian army... agar india se koi bachaa chale jatey den pakistani army return nai krta... 2 2
1868 Hi kia hal hy tum kaise ho kia salgira hy tumhari 1 1
1870 are op logo bap hai bel hai 1 1
1871 India army bad India army ne sari am awrat ka ezat lotlya 0 0
1872 Wo is tarah ki pics iss liye share kar raha hai apni film anay wali hai us ko jo promote karna hai 1 1
1873 mere frnd log apke hi fan he but wo log apke movie me jo chalisa he usse naraj he & qawali sunkar bahot khus
h he 1 1
1874 & Being human k jariye aap itne saare logo ki madat kar rahe 1 1
1875 time mile to 1 rply de do dil ko bahot sukun milega 1 1
1876 awesome ....agr isi trh sare mulk smzdar ho jae to dusmni rhegi hi nhi......plz #Being_Human 2 2
1877 isko dus saal ki hail honi chahiye 0 0
1879 agar eid pe prof ho gya k bajrangi bhai jan mai muslims k beijaty hovy h to mai apk fan nai rhongy forever, 1 1
1883 ami tomake khub valobasi, i love u bhai jan 2 2
1885 picture mein sheer dikhtay hoo aur real mein maha sheer sallu 2 2
1887 Shailly salman bhai online aaenge 3:30 baje Dkhte tujhy rply krenhe ki mujhy :p 1 1
1888 Aap salaman acters hi ho ya koi aur jo salaman khan ke nam se acount banae ho aur usaka profile photo lagae
ho 1 1
1889 Bihar(patna) kab aa rhe hai #Bajarangi_Bhaijaan ke promotion ke liye. 1 1
1890 Bhai jaan aap 3: 30 par ayenge par ham to subah se baithe hai apke intzar main uska kya bolea. 1 1
1893 Bhaijan muze kam ki jarurat hai please muse kam di ji a 1 1
1894 Salman bhai jan aap ne to bahot badi bat bol dali he ki meri film musalmani ke bena hit krke dikhauo ga .... ab
aap bol rahe he ki nahi mene esa nahi bola ....To kisne bola kya hua jawab do aap 1 1
1895 bhi aapke movie ka besabri se wait kr rha hu aur mera aasa hai ki iss sal recod tut jayega... 2 2
1896 sir massage kahan se karna hai sir massage kahan se karna hai sir massage kahan se karna hai 1 1
1897 sir massage kahan se karna hai sir massage kahan se karna hai sir massage kahan se karna hai sir massage kaha
n se karna hai sir massage kahan se karna hai 1 1
1898 Lagta hai jaise jamana beet gaya par 3:30 nai hone wale aaj !!! 1 1
1900 3.30 bje tak bhai yaha video chat karne vale h so apne question ke sath taiyar rehna 1 1

1901 per salman g hm aap video chat pe dekhpaenge to aege werna nai her unjan k sath hm chat nahi kersekte 1 1
1902 Superb salman bhai 2 2
1903 Koun se page par hogi baat bhai koi soon RHA hay 1 1
1904 You better reply me Salman! twitter par nahi toh yahan toh pakka Reply karna padhega lol 1 1
1905 Me abhi se apka page khol ke betha hu 1 1
1906 ye to bajrangbli ka kutta h ye h musalman nhi tum kaafir ko pasand krte ho mujhe nafrat h aise logo se..... 0 0
1907 kamina hai ye 0 0
1908 Jumma mubarakh ho salman bhai weting for you.. salman bhai 1 1
1909 salman ji ap kaha ho.plz ajao 1 1
1910 aaj ki party Salman ki taraf se 1 1
1911 Bhai koe bata aayega baat kese karr sakte hei. Bhai se..???? 1 1
1912 Ye log bhi kese he movie dharm me khasit rahe he 1 1
1913 Salman sir answers dain gey kya.. 1 1
1914 Koi batao salman se kaha bat hogi please 1 1
1915 Salam ap is page py hee rho salman khan jb online hoga to reply dega. 1 1
1917 acha h bhai 2 2
1918 3.30 bje kya hoga 1 1
1919 Kuch to bto koi 1 1
1920 Dekhe kise reply karta hai Mr dabang 1 1
1921 Bhiya ji mujhe ek chota mota roll de dena dabbang three me 1 1
1922 Ramzan ke mahine me film nikalta hai tum kamiyab kaise hoge 1 1
1923 Namaste Bhai jaan . aap sirf bhai jaan hi nahi aap to hum sab ka jaan hey . aur mera Bhagwan hay sir . Jay Bajrang Bali 2 2
1924 Aap msgs padh to rahe hoge pakka h NASA salman 1 1
1925 Jumme ki Shayam bhai k sath 1 1
1927 i love u salman bhai.....bajrangi bhaijaan.... 2 2
1928 india ka ek hi bhai h salman bhai (vo bhi acche vall bhai) 2 2
1929 Plzzz help me bhai plzz read my comment plzz help me. 1 1
1930 Bhai help.me plzzz help.me 1 1
1931 Mai kaha kho gai itni crowd me 1 1
1932 kaisa hai sallu bro... 1 1
1933 Salman 1 baar kuch bhi type to kr do 1 1
1934 wait na karao bhai.. jaldi se aa jao 1 1
1935 ji shukriya apne fns s bt krne k liye 1 1
1936 Sab log teyar ho jaye bhai jaan aa rahe he ....?? 9... 8....7.....6......5.....4.....3......2..... 1 1
1937 Are bhai thoda jaldi time diya hota sali nind kharab ho rahi he 1 1
1938 are abhi to Dus minuts baki hai Bhai.... 1 1
1939 meri zindagi ka maksad he apse milna 1 1
1940 Bhai jaan ap kahi bhi ho jaldi hi wapas ajao meh bhagwan se dua kruga ap jaldi hi aao bhai 1 1
1941 Salman bas mjhe aapse ek ques poochna h bas or kuch 1 1
1942 Agr ap aur thodi der mein nhi aye toh sab pagal ho jye ge jaldi ayu.... 1 1
1943 Bhai jaan hum apke liye ek thofa bej rahe h .....I love you. ....salman khan. 2 2
1944 hmmm salman etna intezar krana thk nhi apne fans ko.. 1 1
1945 Delete mat karna himat hy to jawab to 0 0
1946 Itna mat tarsaoo jaldi ayu 1 1
1947 Bahi ap se 1 sawal 1 1
1948 I'm vry exited... only 3 min remaining 1 1
1949 4 mints more 1 1
1950 Oh mona jamal asa kon sa ans he 1 1
1951 dream 4 me bajrangi bhaijaan 1 1
1952 4 pm ko salman bat krenge 1 1
1953 Kese ho bhaijan..... 1 1
1954 Hi salman I wnt ur pic wid ur autograph.am ur big fan.my qustn is aap aisa kya krte h ki apki kbubsirti din b din badhti hi ja rhi h. 2 2

1955 bhai 4bje ayenge 1 1
1956 30 pura hua 1 1
1957 3:31 ho gye g 1 1
1958 Ap sub ki firki le rhe ho kya'? 1 1
1959 Bhai kha gayab hooo 1 1
1960 Pta h log kitna pagal h apko milney ko 1 1
1961 Bhaijaan app ki film k liye bohot bohot badhai 2 2
1962 Bhai agar app bholo tu main ap ke traf se sab se mil lita ho kya bholti ho bhai 1 1
1963 chal beta selfi le le.......hi.bhaijaan 1 1
1964 Hai salman baijaan main aap ka bhot bda fan Maine abki saari pictures dekhi hai lagbhag 2 2
1965 Radhe bhai aa bhi jaye .. 1 1
1966 100 se jyda msg kiye aapko sir ab to kuch rply do? Kab se ankho char kar ke betha hu 1 1
1967 Salman bhai..... Ki haal chal 1 1
1968 Sir apke sath jo little girl shooting Kar rahe he ek bar boo rath me shooting pe jane se pahle roone lage thi kya??plz reply me. 1 1
1969 Aap ka bajrangi baijaan main aap ka naam kya hai 1 1
1970 Kuch kahiye salman sir 1 1
1971 bhai Eid mubarak advance kOi que. nahi puchne k liye tum jiyO hazarO saal allah bless yOu saal bhar saal 2 2
1972 assalamualaikum sallu bhai jumma mubarak ho sallu bhai twitter par mai hi ho aapko masages karti ho 2 2
1973 3:44 ho gy duss hun kii aa ?? 1 1
1974 Assalaam waalaikum bhaijaan... 1 1
1975 Waiting bhai.. Aur kitna intezar karoyege 1 1
1976 Kiska bhaijan kahan ka Bhai jaan Kutte tu ek nambar shetan,,,, Eed me hi aata he,, kharab karne imman,,, 0 0
1977 Hum vi bath korengi apke sath sallu bhai 1 1
1978 Bhai 3:30 to kab ke ho gye....aap kha ho pls syd aapka ye mgs aana bhagwan b chahte hai ki aap ek bar meri help karo...pls bhai pls I need ur help pls talk to u pls 1 1
1979 7 mins more ab aur wait nai karsaktay khan saab cm fast 1 1
1980 Aslma alikum bhai jaan khan really hero salman khan 2 2
1981 Kis ka Bhai jaan kahan ka Bhai jaan Kutte tu ek nambar shetan, Eed me hi aata he, Kharab karne imman, 0 0
1982 Sir sare pagal ho jye gye jaldi aaa jao 1 1
1983 AJA SHAM HONE AYI....MOUSAM NE DI ANGRAIIII....???? Plz reply me BB.......LOVE U CHO MUCH...????......taktae rah te tujhko sanjh savere....love u my BIG BOSS....BB 2 2
1984 Nhi aane wale aap pta h. Sbko hhahah 1 1
1985 Heyy sallooo...aap apne in sab fans ko reply karo na..kab se wait kar rhe h sab...m still waiting. ..20 yrs se yahi kar rhi hu.ab bhi kar sakti hu....m not ur fan.....I <3 ...:D :D :D. 2 2
1986 chor sale marja tu kutte haramke suwwar. 0 0
1987 4 baj gye salman sir 1 1
1988 Aisslam wa allaiquem bhaijaan 1 1
1989 Plz come pavan kumar chaturvaidi 1 1
1990 bhai 3.30 baj gaye hain... 1 1
1991 Sir plz ek baar hmsbko hiiii to boldo sb wait krre h aap bat mt karo i know ki apke pas time nhi h pr sirf ek baar hmsbko hiiii boldo sir hm sb etne me hi khus hojaenge plz ek baar 1 1
1992 Asslam walekum salam sir 1 1
1993 Nhi baat karni to boldete 1 1
1994 Salman best luck for bajrangi bhaijan..hamesha ki tarah iss baar bhi sare record tod degi...inshallah 2 2
1995 hum to taiyar hai 1 1
1996 khash ap mjy ad kr kr ....i know esa ap kro gy ni.... 1 1
1997 4 bj gye lekin party abi baki h sir wh bhi eid ki 1 1
1998 Kaha ho bhai jaan ap... Sab wait kr raha ha 1 1
1999 by by nhi aane wale salman 1 1
2000 8lez,ap fix tym bta dijiye sir ki kb are ho.ap fb p 1 1
2001 Muja milla msg salman bhai ka 1 1
2002 Dekte hi kayse hit karta hi film Salman is kamina 0 0
2003 4pm baje karenge . direct baat mere saath . this is what bogg bosss siad rightly 1 1
2004 Bhai mera nasm saaru h jaise aap ka sallu h 1 1

2005 nahi aayega Yr salman Chalo jagah Khaali karo yaha se :p 0 0
2006 Jaao apne fans se baat karlo sab wait kare h 1 1
2007 Bhai bohot time hogaya hai abb toh shuru karo!!!!! 1 1
2008 Bhaijan hum appka wait kar rahe hai pls ao 1 1
2010 Jaldi aa jao Salu bhai.bhai aap kahan ho 4pm ho gaya...I'm waiting you bajrangi bhaijn...Aap bahut kamaal kart e ho... 1 1
2012 Koi bhi yeh chutiyo Salman Khan picture nahi dekhne musalmano be care ful picture ko flop karne ha 2 2
2013 Aapko itni cmnts aayegi.. Aap toh reply b ni kroge.. 1 1
2015 love uuuu soooo muchhhhhhhhhhh jannatttttttttt 2 2
2016 Hi bizy man ap hum sy kaha baat karo gy apko razan Mubarak hooo eid par SMG zaror karna plz plz bye 2 2
2017 helo my bro sallu 1 1
2018 We love you Salman bhai <3 <3 <3 <3 <3 <3 <3 <3 <3 <3 <3 <3 2 2
2019 bhai aap kahan hai 1 1
2020 Sab record peche bajarangi bhai jan agee.... 2 2
2021 Bhai comedy. Laavo na...aap comedy with kapil me jitna hase ho ..utna haste hue pehle nahi dekha 2 2
2022 Salman bhai min ap say bat Karna chata hn plzzzzzzzz bhai 2 2
2023 Bhai aap dildar ho m janta aapse bda koi ni h dil ka raja 2 2
2024 Tu musalman ke bina movie hit kar sakta he bola he na,,,,chal karke dikha,,, 0 0
2025 Salman bhai rumors phailri hai ki aap ne kaha Muslim ke bina movie hit karaonga is that true? 1 1
2026 I am here baat kaha krni ha plzzzzzzzzzzz baat kr lo 1 1
2027 mujhe to pata hi nahi raha bat ho bhi rahi hai sorry jaa rahi hu bye 1 1
2028 kavi baat karo 1 1
2029 Vry nce sir. <3 2 2
2030 Sir ek nzr cmnt pe rkiye or dekhiye kitne sare log apko pyr krte h:) 1 1
2031 salman bhai haqiqat men to nahi milte ap kam az kam khuwab men dua salam k liye ajaya karo 2 2
2032 aray ye kloi kamina he bhi k name se id open kia he bar bar hi hi kar raha 0 0
2033 Salman bhai,kabhi aap samne miljao na,toh jarur apke gale laag jaunga,,taab mera v kick laag jayegi,,, 2 2
2034 eid pe araha hon filim dekhne bjrngi bhi jan 1 1
2035 Arif khan kounse news channel par dikhain hai sirf itna batado 1 1
2036 Kaha salman khan 1 1
2037 Bhai ap jhakas ho pr sab ne body ap ko dekh kar banaaya or ap mote hote ja rahe ho 2 2
2038 Super kota salluu 2 2
2039 BHAIJAAN ... KAVVI KOLKATA may aoo .. apni fanzzzz say milne ... already Banjarangi bhaijaan MEGA . .. HIT ... 2 2
2040 Shi me salman bhai jaan ki movie record ho gi 1 1
2041 aap to being human ho aap ka flim hit to honahi hai itni logoka duya jo hai. 2 2
2042 Adab adab arz hy bhai. 2 2
2043 Insha Allah blockbuster hoge abki bar 300 cross Paar bajrangi bhaijaan 2 2
2044 Is mein koi shak nhi ho gi salman bhai ki movie ho or record na ho 2 2
2045 bhaijaan hum bht pareshan h ye sunkar ki apko kisine kidnap kia h ap plz ek baar rpl kariye na.... pls bhaijaan 2 2
2046 Bhi my ap ke hr movie boht shok sy dekhta ho or ap ke movie ka wait krta ho 2 2
2048 Faltu log hi jayenge is harami ki movie dekhne isse kya pata Islam kya hai 0 0
2049 Bhai mein to aap ka diwana hu aap ki koi se bhi film aati hai first show dhekta hu aur is saal bhi main dhoom macha duga.... 2 2
2051 Bhai love you love you love you love you love you 2 2
2052 Superp bhai jaan 2 2
2053 bhai aap toh hit ho 2 2
2054 Bangladesh meh apko chahne bala bohut khub.. 2 2
2055 kabhi to msg ka ans dy diya kro g 2 2
2056 Love you love you salman bhai 2 2
2057 Luv u bhai...u r so cute....sbki jaan salman khan......... Anitha Parmar 2 2
2058 1-no bhai 2 2
2059 Me apka fen bhi hu aur apko sabse bada guru manta hu. 2 2
2060 salman bhai sab aapko millna chahty hy but meko aap miloo ya na millo bass duaa dyna our aapke jyis meko b

ing human trust nikal na hy help me takee our bachho ko help kar sako mai bass duaa millaa 2 2
2061 Llllllllooooooovvvvvvvvvvveeeeeeee youuuu salmaaann gggg 2 2
2062 Kab aye ki kab gaye ki 1 1
2063 Super duper hit 22 ji 2 2
2064 Mere cmnt padhne waalo. Jab is madar chhod ka bajrangi film flop kre to samajh jaana k muslim ka haaye laga ise. 0 0
2065 Aa to jao id wale din hi dekh lennge ki iss baar bhi record tutna hai pehele wali movies se haha....... Being salman khan? 1000 crores 2 2
2066 Aswlkm sahre hogye apki 2 2
2067 I love love u love u love u love u so mach salman khan ek bar mujha apko i love u fac to fac kahna ha 2 2
2068 Are Yaha kya kar rh ho. Salman dusre satuts pe aa kar Baat Be karke chale Gaye 4:00am par 1 1
2069 Bajrangi bhai jan to supar hit hai kyoki sare musalman bhai salamn khan ki film dekhge... 2 2
2070 Umar bhar aap eysai hash te rehay or you hi movie banate rehiye 2 2
2072 kya kahu bhai tumhara har stilepe josh hai,mano ya na mano sare ledki tumpe marte hai.jisko chaho apna banalo,khuda ka kasam kabhi na mat bolo. 1 1
2073 Me duwa karta hun ki bhai aap ka ye muvi San ka rekod tod de 2 2
2074 me apka bhot bda fan hu 2 2
2075 bhai mey aap ke saath hoon.....app ko shadi nahi karni mat kijiye.....jai jawan 2 2
2076 Humari dua apke sath hai bhaijaan apko kuch nhiho sakta 2 2
2077 Hello hay salman brother ey kaya bola aapne h/ m bolo 1 1
2078 salman sir apke being human trust ki small cities me bhi requirement hai ..pls. jitni jyada ho sake utne logo ke madad kejiye....and BAJRANGI BHAIJAAN Ki JAI HO. 2 2
2079 Salman bhai milenge kab intezaar hai aapki 2 2
2080 salman bhai aap meri life ho m bacpan se aapko apna bhai manti ayi hu apse badkar koi nai h mere liye bas ak baar mujhe bahan kah do best day of my life 2 2
2081 Salman bhai pbliciti vale film kyo bnate he sethi sathi film bnavo na. Me aapka bhot bda friend hu. Lekin. Kya kru ye sabne afva failai he. 2 2
2082 Salman Bhai ye film bhi super hit hogi aap be fikar ho jao 2 2
2083 Isbar tmhari le li jyegi preshaan na ho 0 0
2084 dekho wo aaa gya .... fir se ed pr edi lene sbka apna bhaijaan................ 2 2
2085 sabki aan sabki shaan ek humra bhayi jaan salman khan . Best of luck for film salu 2 2
2086 I luv sallu 2 2
2087 Salman ap ko aur ap ki famliy ko meri taraf se id mubarak 2 2
2088 love uuu saluuuuuuu 2 2
2091 Kya baat h bhai 1 1
2092 bhai ko aksle wale kum 1 1
2093 Salman bhai jan main apka wait kar rahi ho reply 1 1
2094 loveeeeeee uuuuuuu yaar thak gai tum hi reply 2 2
2096 Bhai kuch Hindu ko kush hogaye ap ne fans ko bahut naraz kiya 1 1
2097 Sir meri ek hi ichha h apse milna 1 1
2098 Salman bhaijaan. .Mere bade bhai..mujhe aapse Accha Koi nhi lagta. ..m Aapko apna bada bhai maanta hu... 2 2
2099 Bollywood big super star bajrangi bhaijaan salman khan 2 2
2100 Bajrangbali ka saath Bajrangi ke pas ?rkho viswas ?puri hogi aap ki har aas???????! 1 1
2101 Hum bajrangbali ke bhakt h mr jayenge magar jhoot nahi bolengei miss you broi like you bro 2 2
2102 salman ji aap bahut ache hero ho aur aap k har film dekhne me bahut ache lagte ho aur bhagwan se duwa karta hu ki aap k bhajrangi bhaijan india k liy sabse bada film hoga aap k liy i miss you salman ji,,,,,,,,,,,,,,,,,,,,,,, 2 2
2103 apki aane wali film bajrangi bhaijan suuuuuupppppppeeeeeeerrrrrrrrr hit ho 2 2
2104 hum bajrangbli ke bhact h mar jayenge par jhoot ni bolenge 2 2
2105 Salam malikkum bhai jaan 2 2
2106 Bohat achi ha ap thek hai ramazan k se gura ha 2 2
2107 mere bare me itna mat socho me dil me aata hu smjh me nhi 1 1
2108 salman sir plzzzz sir madat kro sir mai 4saal se aapka number dhoond raha hu sir plzz madat kro mere parivar ko aapki jarurat hai salman sir plzzzz sir....yeh mera no...hai ..,.7355458113 1 1
2109 Sallu miya wonder ful 2 2

2110 Hamesha Bhai bola par tu Bhai bolne k layak nhi 0 0
2111 bb will be huge blockbuster. aap jiyo hajaro saal. 2 2
2112 aur galti tumhari nahi hai pyar koi karta nahi ho jata hai tumhe hua hi nahi mai isliye milne ke liye gidgidate ra hti hun tumhe koi phark nahi padta 1 1
2113 Bhai ..kab bajen ge 3.30 1 1
2114 Brillient jeee i m muslim from pakistan janab pakistan buri jagah nae jo ap yahan a nae skty ....m bula nae ra e bta rae hun ana howa to most welcome mery colg m b aiya gaaaa 2 2
2116 Salman khan chahe tera dad musalman hai chahe teri mom hindu hai chahe tu barti hai chahe tu koibi kisi bi ch okath ka kuta kuta kuta hai hai hai salman khan tune allah ke name ko istara behudmi nahi karni chahie thi 0 0
2117 1 chaahe 2 karik bajrangi baijan padgya sab bari 1 1
2118 I love you meri jan aap sah bohot piyar karty hoo 2 2
2119 film me apki pose mast hai bacho ke sath me exclusive pose bhai jaan 2 2
2120 hi sir kesey hai aap n aap ki famliy i hope aap n aap ki famliy sab theek hon gey god blees u n best of luck for ur film bajrangi bhaijan super hit film this year 2 2
2121 Nice salu bhai 2 2
2122 Sallman to bhout sir pakru chinal ka hai 1 1
2123 yaha sab mental hai.. ok lage raho yaro 1 1
2124 Bahuth dinonke badu aap ka face me khusi deku rahahu. 1 1
2125 Sabse kimti cheej mili pahchan kafi hai 1 1
2126 kutta to kutta hi hota hai tu salman nahi tu 1 dalal hai 0 0
2127 kya bhai aapka ye movie bahubali record tod paey 1 1
2128 kya bhai aapka ye movie bahubali record tod paeyga 1 1
2129 yadi apse milana he to kya krna hoga 1 1
2130 Bajrangi bhaijaan is awsome 2 2
2131 Assalamualaikum salman sir 1 1
2133 phle to timing 3:30pm thi 1 1
2134 bt apne 3:30 kaha tha :(1 1
2135 Bhai plzzzz jldi a jao 1 1
2136 Kkia hha wifi ni hai 1 1
2137 salman aapne 3:30 bola tha 1 1
2138 Adha ghantaa badh gya 1 1
2139 nahi baat karni hai toh abhi k abhi! nahi toh phir bajrangi bhaijaan ka trailer dekhne me busy hu :) 1 1
2140 Sallu bhai kisi ko rply nhi krte or humlog bewkufo k jese sallu bhai sallu bhai kiya krte h 0 0
2141 salaam sir kaise ho sir plz inbox per message karo 1 1
2143 ok come in....lekin kese salman bhai...majak nhi kar rahe 2 2
2144 3:30 ka time tha 1 1
2145 bhaijaan jaldi aao fb par waiting 4 U 1 1
2147 Ek bar Salman bhai n commitment krdi to fir wo kisi ki nhi sunte ..Sir y to galat baat h Kl s wait krhe h 1 1
2148 Bhai esa mat karo 1 1
2149 helo bAi kesahe up 1 1
2150 Bhai bhai bhai bhai.......love u bhai..... 2 2
2151 Kahte h ki salman bhai kabi juth nahi bolte 1 1
2152 bhai....... 4 nhi baje ra.......3:30 ma he kr lo na..... 1 1
2153 sir hamare ider 11;30 am ho gaye jub aap aao gaye mein baat nai kar saku gi plz mera messages per abi baat ka ro chote kp nursery se laana hoga mujhe mein london mein rehti hu plz sir reply de dou 1 1
2154 Jaise apki marji 1 1
2155 Kya baat krni hai msg kr dena.. #Salmankhan 1 1
2156 Pls 4 pm se zyada late mat krna Salman Khan 1 1
2157 Omg aur 30 minuts.. 1 1
2158 Bhar do jholi meri 1 1
2159 Aap katrina se shaadi kyun nahi kar lete...? Sallu miya 1 1
2160 AAH Salman aaj 4 bje khana benana he kel ka time rekhlo dear subha 11 ka aaj nai posible plz try to understan d 1 1
2161 bhai Salman Khan meri class h 4 bje apne 3 kaha tha phle :(1 1
2162 apne bola tha 3:30 n now 4pm :'(1 1

2163 dekhte hai bawa 1 1
2164 Kha bt krenge 1 1
2165 inbox ma bat karo sallu 1 1
2166 bhai 3:30 tha time to 1 1
2167 bhai commitment kiye ho k 3.30pm aaoge to ab q bhai majaak kr rhe ho 1 1
2168 Sir, Middel Class ki iteni postion kaha ki aap ke saath bat kar sake wo..driect... Salman Khan Sir. 1 1
2169 Bajrangi bhaijaan mein koi aisa scene jo yadgaar ho???? 1 1
2170 Array pic toh change kar do Yeh 3:30 wali 1 1
2171 tik hai bhai jaisa aap bolo 1 1
2172 In logo pr itna tym kha jo hmse bt kre 1 1
2173 bhai jhut mat bol salman kha mere paas bethe he yaha delhi me ullu 1 1
2174 Abhi kiu nahi salman 1 1
2175 Zadardast plz jald e yeh time guzry 1 1
2176 ok.bhai mere pass bahot sare questions hai... 1 1
2177 dear friend....... ek kaam karte hain .... 4 baje hi baat karte hain 1 1
2178 Ok ..chahe jb ap bolo 1 1
2179 Hame to 17 ki whit hai 1 1
2180 Sallu bhai meri bhoot fan 1 1
2181 Really kahi mai koi khwab to nhi dekh rhi dear salman :p 1 1
2183 Salman khan sir 3.30 tha na 1 1
2184 Salman bhai plzzz Pakistan ap zrur ay bht acha swagat ho ga ap ka plzzzzz bhaiiii 2 2
2185 Hiiii sallu bhai.. .kaise h..bhaijaan...apne to kamal kr diya bollywood industry mei. . 2 2
2186 Per hum kaisy aap sai bat kar sakty hain plz tell me plzzz?z?zzzzzzz?zz 1 1
2187 Luvvv u luvvv u luvvv u 2 2
2188 Salman bhai still waiting 3:30 baj gaye hai 1 1
2189 I cant wait plze cmon mve fast bhaijaan 1 1
2190 raat k 12 bje b kr lenge ...ap aao to sahi 1 1
2191 Asslamalaikum salaman bhai 1 1
2192 Aap ki baat hum sab ke sath:-) ;-) :-D 1 1
2193 hy sir kese ho aap ? 1 1
2194 sir aap Lucknow kab aarahe ho 1 1
2195 Ye kya bat hue sir abhi bat kro plz 1 1
2196 assalamwaelkum bhia jaan hm v h ap k chahane wale 2 2
2197 Ek baar commitment kardi 4:00pm phir kisiki nhi sunnte :) 1 1
2198 Luv u bhai jaan 2 2
2199 Tm ho koun? Asaduddin owaisi sahab ne tmhri aukat dikha di.. abhi b tmko samjh nhi aaya 0 0
2200 Kya Salman bhai...mene job se 6uti lekar aaya apse baat krne ko ab aap 4 baje milege.. 1 1
2201 kya bhai sb kaam nipta kr aya tha ap se bt krne 3:30 ko or ab ap 4 pm.. chlo koi nhi ab 4 bjne ka wait krte h :-) 1 1
2202 Kamal karte ho bajrangi bhaiya itna intzar ;-) 1 1
2204 Bhai apse ek sawal aapne aaj tk apki ek bhi movie me apka real name kyu nii rakhe 1 1
2205 Hum to 2 vje se w8 main hai 1 1
2206 kamal karte ho salman Ji 1 1
2207 I'm so excited salman bhai 1 1
2208 #aaj #sabhi #superhits film.dekhane chale #bbthiseid #superdupar film 1 1
2209 bhai sidhi baat kewal mere saath 1 1
2210 aap ye btaie ki aap lucknow kyu nhi ate hai 1 1
2211 Abhi kro na bajrngi bhaijan. . 1 1
2212 # 4 baje ge aaaj party abhi baki.wow 1 1
2213 bhaijaan kaha ho 1 1
2214 oo bhikari.....kya bola tha tu muje muslaman ki zarurat ni nai abe sale tere jaiso ki musalmano ko zarurt nai hai 0 0
2215 Mohammad Anish bhai owasi always wroung yaar aur muslman aur hindu both ek hi hai sab Ramjaan mai Ram aata hai diwali mai Ali 2 2
2216 wali qum Haslam....bhai ....! ramJaan Mubarak......! bajrangi bhaiJan....! 1 1

2217 Help me bhai plzz help me 1 1
2218 bas aapko milne ki khwaish hai Inshallah agar zindagi rahi to Allah wo bhi puri krdega Inshallah... 1 1
2219 aap ke whtasup no. de do uspe bat karengai..... 1 1
2220 Apse life m ek bar mulakat ho jaytoo life m aana safal ho jaygaa 1 1
2221 Aik Confusion dur krna bt kr k agr hu sake tw.. 1 1
2222 Ok bhai mai aap se bat karunga bahut important bat hai 1 1
2223 Kaha kaisy? M karna chati ho aapsy bohot sara baat 1 1
2224 aj ki paty bhai ki tarafse 1 1
2225 Baba ham to lagta he bs intzar ki krte reh jayenge 1 1
2226 main aap ka bohut bade fan hu aap to sab ka help karta hai mera bhi 1 help kardena bhai plz bhai mujhse baat k arna i miss u bhai 1 1
2227 Apko pta nhi hh m apko kitna psn krte hu 1 1
2228 Bhai pe plz koi rong cmnt na kare 1 1
2229 ek din m apne ganv se cycle chalake milne aa raha tha aapse. Abhi m Julwania mein hoon sir . 1 1
2230 bhai kaam se fariq ho jau to bta dena pahle aap 1 1
2231 Agr time mile to mujhse bhi bat kar kena... 1 1
2232 Salman ji apka favorite gAna konsa hai bajrangi bhaijan film ka????? 1 1
2233 abe kutte tere se kon baat karega 0 0
2234 Sallu jaan , jumma mubarak 1 1
2235 Kya bat kare aap se bhai sahb aap ne to apna wajod hi badal dia Fir bhi thanks for massage 2 2
2236 Ab sabr kab tak kare intezaar ki bhi hadd ho gyi 1 1
2238 ahhhhhh mujhe milna hai tumse pls sona ek bar mil lete hai na mujhe tumhe aise apne samne dekhna hai tumse bat karna hai :-(:-(:-(:'(1 1
2239 Bhai aapke baare mein koi kuch bhi kahe hame kuch farak nahi padta aap hamare bhai ho allah aap ki movie k o super kare jumma mubarak bhai khuda hafiz 2 2
2240 Saloo bhai plz pak ajao m india nhi aa sakhta :D 1 1
2241 man ki bat ya dill ki bat......? 1 1
2242 Jarut karenge awashya karenge ....aapke fan jo hai.. 1 1
2243 jaldi aao na yr salluuuuuuu hum log apka kabse wai wait kr rahe h 1 1
2244 Mile jab the hum tum yaad hai yaa bhul gye 1 1
2245 tic toc ..tic toc...yaar ye time kyu nahi ja raha.. 1 1
2246 sir kase pata chalega aap online ho aap se baat kase karunga 1 1
2247 7,600th like :) 1 1
2248 Kbhi kbhi mere dil me tum bhi aate ho 1 1
2249 Ap se Sirf ek swal krunga bhi jaan 1 1
2250 kya bhai aaj ki shaam salman bhai ke fans ke naam 1 1
2251 kya 3:30 bola tha 1 1
2252 Hiiiii, kya yeh dream hai? 1 1
2253 aaja sham hone aayi 1 1
2254 App ke Intjar mein..luv u bhai 2 2
2255 Koi kitana bhi virodh kare hum movie Dekhenge...because hame pata hai ki salman sir sab dharmo ko manate hai support karte hai aur koi galat kam nahi kar sakte...love u salman sir 2 2
2256 Aapse baat hogi .dil nhi manta. 1 1
2257 Bhaijan i'm big fan of ur.bhaija m aapko follow krta hu or aapk jaise acha work krne ki kosis krta hu. 2 2
2259 Salman bhai aapne roza rakha he ke nahi? Ramza ka 1 1
2260 4 pm toh ho gaya abhi taak...........! :(1 1
2261 Aslam alekum jumma Mubarak ho sallu Darling... 1 1
2262 Hai bhai jaanapp hi ho humlogo ja bajrangi bhaijaan 1 1
2263 bhai jaan jai bajrangbali apki movie jarur blockbuster hogi 2 2
2264 sir I love u sir wait Karenge Ganapati baba ki jai bajarangibhai Jan ki jai 2 2
2265 Bhai apki film ka whatsapp pr kuch mesg a rhe hai kya ye meg sahi hai 1 1
2266 Bhai pata nhi apku ye msg mile ya na mile par Kuch dino se apka dimag kharab hogaya h lagta ap ab do nao m e sawaar hone ki koshish karrahe h behter yahi hoga k shahrukh ,aamir k jese ek imaan pe raho Allah hafiz 1 1
2267 Sb bolte h mai apka bht bda fan hun lkn apki fan nhn apki ac hun plz jzt 1responce like cmnt kch v ek br mra n aam h bol do bs wai kaafi h 1 1

2268 i wish meri ye wish puri ho jaye.... 1 1
2269 Bhai jaan salam lekum aapke pass time hai 1 1
2270 OK main aap ki intzar karugi Bas aap sache Salman khan hai Toh Itna jarur batana aap kaisa hai.....:) aapko de khne ko Dil chahta hai ??? 1 1
2271 Mai 2 year phle mumbai aaya tha bhut kosis ki but aap ko Dekh na saka 1 1
2272 Kaise ho aap papa sorry hum sogaye the abhi uthe h thodi der kardi na hum ne but koi baat nahi abhi baat karte hum dono haa. 1 1
2273 Agar aap mere comments ka reply krenge to me isi se samjh jauga ke mere comments ke reply me Aapne pure Pakistan ko apna pyar salam or dua dedi 1 1
2274 Ap ko pakistan si kch behja tha pata nhe ap ko mila ha ya nhe 1 1
2275 Meri ma apko bhot yaad karti he bhai 2 2
2276 aisa sahi me hoga kya 1 1
2277 Bhai aapko sabke comment mile honge par mai ye aapse kahna chahta hu ki aap eid par mere ghar aaao or sew ainya kha lo or ek bahut jaruri baat aapko batana h mujhe aapke reply ka wait rahega 1 1
2278 3:30 p kyon nhu 1 1
2279 bhai apko jumma mubarak or mai phele nahi jata apki movie dekhne ab jaunga kyuki ap fb pr bate karoge hum se 1 1
2280 Mery chanda jaldi kr. 1 1
2281 Time math chage karo bhai 1 1
2282 Ihet salman tum is bar floap ho ha ha hamare city me nahi chalegi 0 0
2283 Ye bhavu aapan bolu 1 1
2284 Kamal kartey hoo.Jab bhai ayenge tou awaaz tou hougi hii.waiting for the Wright time. 1 1
2285 you are great,Bhaijaan......love u bhai,love u bhai,love u bhailove u bhai,love u bhai,love u bhai,love u bhai,love u bhai,-V E R Y MUCH.... 2 2
2286 Allah aapko khush rakhe or aap yun hi Blockbuster Films dete rahen or logo ko Entertain krte rahen Ham sab ki naik khwaishat or duaen aapke saaath hain... 2 2
2287 Plzzz ya zaror bata do bs yahi k ap ko jo ma snd karti hun apko milta ha ya nhe plzzz ya bata do 1 1
2288 Kaise muge bhi apse baat krni h ... 1 1
2289 bhai just ek baar hi bol do 1 1
2290 Oh ho kab bajega 4pm 1 1
2291 Bhai aap kha baat krogge 1 1
2292 4 to baj Gaye 1 1
2293 18 mnt aur wait krlo frnd 1 1
2294 Bhut pyar krte hai hum aapse...bs aur kuch nhi.. 1 1
2295 tu musalman nhi to name bi chng krle apna santi lal rakhle 1 1
2296 sir plz mujhe app apna bodygurd banalo plz sir 1 1
2297 kya aap human being ko hmesha support kroge ??? 1 1
2298 Han sallu bhai...ye abhijeet ke 2lagao....bakwas pel ra tha usdin....kese dost bna rkhe ha jo apse mel.ni.khate.... u r a philanthropist and he is misanthropist 1 1
2299 Oh..ho sacchi... #salman_sir bhale aap baat kare ya na kare bas 1 baar #chhattishgadiya_sable_badiya bol de.... I_love_u... Ek aap bas acche lgte mujhe.. 2 2
2300 tu galat baat hai 1 1
2301 Aap log Salman Khan sir k liye kya KAR sakte ho 1 1
2302 ap hi sach boldo ye sab photo wagaire sahi hai ki nhi 1 1
2303 M sorry bhai 4pm me mjhe thoda kam h rat ko diner pe bat karte h 1 1
2304 Salman Khan se live baat .... mujhy lg raha hai ky khuwab hi hai mera itnay bary star ......or hmsy baat wah..... 1 1
2305 Bhaijaaaannnnn ab tuuuuu aajjjaa 1 1
2306 reaally kia ye hosaktha hai Salman Khan 1 1
2307 Sach ka samna .roza he ke nahi ye batao? 1 1
2308 Bhai tumse muje ek baar milna hai lve u bhai jaan 2 2
2309 kaha pe baat kroge Bhai ye toh bata do 1 1
2310 Bhai aapse baat karne ko kitna wait kiya hai pls....bhai pls talk to me I need ur help...pls main aapse ek baar baat karna chahti hun pls talk to me...bhai pps help me pls 1 1
2311 aaj ki party #Bhai ki taraf se. 1 1

2312 sabhi bandra aao mere ghar par sabse milunga main 1 1
2313 Bht bht taklifda kam ha 1 1
2314 salman bhai ...... i lovee uuu tumharee liyeee jan bvi dee dengeee 2 2
2316 hello Kya main mr. salman khan se baat kar sakta hoon?? 1 1
2317 bar bar time mat badal na .barna lg apko vul jange 1 1
2318 oh salman bhai 1 1
2319 Help me hlp me plzz bhai 1 1
2320 Bahi main ne ap sy boht zarori bat karni hai @BeingSalmanKhan 1 1
2321 Bhai ne ek barr commitment kar di to wo fir kisiki nahi sunte 1 1
2322 o really ,maja ayega ,kab 4 baje ga 1 1
2323 abe chirkut logo thoda hawa to aane do itna bheed lga k rkha h chalo baccha log sb shanti se baith jao koi shor nhi machayega bhai aap ek ek krke sb se milo 1 1
2324 4 baje meri college mein class hai bhai jaaan :(1 1
2325 itna intazaar :O :(1 1
2326 Lv u bhai mere salman bhai 2 2
2327 Ek time mein sb sy kaisy krain gy baat 1 1
2328 Bhai jaldi karo na 1 1
2329 Kon kareaga rea terea sath bt tujhsea acha dog sea bt kr lu 1 1
2330 Bhai fahara khan ko me hu na ki khani ki jrurat he or vo mere pas he 1 1
2331 Swagat nahi karoge humahra..??? 1 1
2332 Ami tomar sathe kotha bolte chai Bhaijaan. 1 1
2333 Muje aap bhout aache lagkte hai bachpan se dekti aa rai hu or marte dam tak dekungi i am a big fan. . . . 4rm n epal 2 2
2334 Ek baat bta deta hu aap sabko.... Plz ijjat se baat krna SB log 1 1
2335 Bhaijaan I'm eagerly waiting 1 1
2336 salman sir plz reply..my cmnt my dream wil cm true.. plz reply with my name 1 1
2337 Bhai aap ke liye kitna bhi wait krna pade karenge 1 1
2338 Bhai jan jldi jkdi 1 1
2339 bhai hame bhi naraj na kariyo 1 1
2340 3.30 ka bola tha yar pelwan aapne 1 1
2341 hii bhaijaan...ab intjaar ni ho rha h...apse baat karni h 1 1
2342 Intezar kab tak hum karenge bhla 1 1
2343 4 baj gye haiiiiiii 1 1
2344 salman bhai mai aapka bahut bada fan hu aapke sath filmo me kam karna chahte hai 1 1
2345 bhai aap se bat kab hogi 1 1
2346 Bhai firki to nahi le rahe ho 4 baje mat kehna 4:30 baje please 1 1
2347 okk bhai jaan 1 1
2348 salman bhai shadi kab karoge? 1 1
2349 kaha hai aap bhai#salman.....#juma mubarak ho...... 1 1
2350 abe tm log jyada chapad chapd kroge to bhai jaan chale jyenge bola n sb chup chap bhaitho ekdm shanti shanti shanti aankh bnd kr k no type any commnt sirf bhai ka dhyan lgao 1 1
2351 salman sir plz 5:00 pm kar dou plz 4:00 pm mein nai baat kar saku gi aapse plz sir plz 1 1
2352 Neha sapne aCHCHE H MAGR YAD RAHE SAPNE SUCH NI HOTEY 1 1
2353 Arey yrr jaldi kro wait kr kr ke dimag ka dahi ho gya 1 1
2354 hum bajrang bali k bhakt h Mar jayenge lekin jhut nahi bolenge.. 1 1
2355 4bje to m gf ke sath ghumne jaunga ..m baat ni kr paunga 1 1
2356 aapko late aane ki aadat hai me janta hun... 4baje bhi sayad start nahi hoga... 1 1
2357 Hiii salman bhai me aapka bohot bada fan hu aap mujhse bhi baat karenge kya plz 1 1
2358 hahaha...feeling osmmmm 1 1
2359 Sallu bhaijaan ko salam 1 1
2360 Jai ho bhai. 1 1
2361 4 toe hogaya na sir.... 1 1
2362 send naaa plZzzz 1 1
2363 8 mints ...... lub u bhai jaan....... :* 1 1
2364 Muje call karo bhai jan 1 1

2365 aaj ki party fb pe salman bhaijaan ki tarf se 1 1
2366 pleaseeee cevap dena bhai 1 1
2367 ME AAPKA BHOT BHOT BHOT Bd fan hun 1 1
2368 kab kitna karaoge..wait 1 1
2369 Keh do tumhe ya chup rahu 1 1
2370 7 mins left......waiting bhai...... 1 1
2371 awoo kare baat salman ke sath`-? 1 1
2373 abe tm log nhi manoge tikhi mirchi wala nasta kraunga sb ko tbhi theek hoge.....tb salman salman nhi pani pani chillana smjhe 1 1
2374 ILove u salman sir plz hum se chat karo 1 1
2375 7 mnt or 1 1
2376 subki aan subki shaan hai apna bhaijaan 1 1
2377 bhaijaan kaha ho??? 1 1
2378 Yeh 7 min toh khatam hi nhi hote. Aap kaha ho jaldi aa jao. 1 1
2379 3:53 now 1 1
2380 bhai tho meeting nahi cancel kartha agar committment de deha 1 1
2381 Bhai 4 bje page par hi bt kroge inbox me bt krni hai mjhe to bt bht personal hai 1 1
2382 6 miniute ago .....waiting indias super star salman khan 1 1
2383 6 min bhai 1 1
2384 Aray bahi aao ooo na ....?? 1 1
2385 kha ho aap shona cant wait...... :-) 1 1
2386 5 mnt or 1 1
2387 hiii ab to 4 baj gye 1 1
2388 hum apni new movie bajrangi bhaijaan ki dil se dou krte hai ki vo is sal ki kuvh shandar movies me samil ho b haijaan 1 1
2389 Hlo sir hm sb aapka wait kr rhe h 1 1
2390 plzz slaman jrur aana onlin or plzz jrur baat krna apne fans se plzzz..plzzzz... 1 1
2391 assalam walekum bhaijaan 1 1
2392 3min bs bhaiii 1 1
2393 Help me bhai plzz help.me 1 1
2394 sachi salman hm to din se wait me hai ki apse baat kar pay 1 1
2395 Bhai 5 mint rhegaye 1 1
2397 ajao vaai party karte hai tm or hum fans lok 1 1
2398 5mnt left....yhoooooooo....salllluuuu 1 1
2399 SUNO SAB CHUP JO JAO BHAI AANE BALA HAI 1 1
2400 Plz salman sir 1 1
2401 Bhai 4 mint 1 1
2402 AJee sunte ho.......ab aaaaa bhi jaoooooooooooooo :) 1 1
2403 Bhar do jholi meri salman bhai 1 1
2404 bhai sahab aayenge 1 1
2405 Sallu aj m bt kiay bgair nh jany wala 1 1
2406 howz it possibleee.. and ksee bhai jan 1 1
2408 Bhai kia karne arahe ho 1 1
2409 Bhai ko mera salam 1 1
2410 ok ap chahe 5pm pe aana par online hona 1 1
2411 Ekbar meri dream koh sach kardona bhaii 1 1
2412 friend yahan internet bahat slow ..... hai./..... sorry 1 1
2413 sab se bada my hun salman khan ka deewana 1 1
2414 kya aap bengali language bol sakte ho? 1 1
2415 hey bajrangbali bas aaj ek reply mil jaye.. 1 1
2416 Sir mai sab me se eak alag salg fan hu .Maine suna apka apharan huwa 1 1
2417 sallu bhai plz ab toh a o jani 1 1
2418 Bhai plz plz jaldi aao 1 1
2419 Hi salman bhai i am sameer thandi hawa ka jhokha... 1 1
2421 Bhai jaan ... plz ek bar mera name typ kr k hiii bol do plzzzzz bhai? 1 1

2422 Aaj meri admission thi. But bed nahi mila. Isliye admission ho nahi payi. Isliye main yaha chali ayi. :) :) :) 1 1
2423 bhai aa jao 1 1
2424 Sallu agia fb pe 1 1
2425 3.58.... ohhh cant wait :) 1 1
2426 Salman ji , aap ko haleem pasand hai? 1 1
2427 Muj ko ap sab ke dowa chahiye fans 1 1
2428 bhai jaan kidhar ho aap 1 1
2429 My heart is beating for u...hr ek pl ke liye hum aur hamari family aapke liye dua krti h...duniya kuch bhi khe h me kbhi koi fark nhi padhta....aap aur aapki family hamesha khush rhe aise hum log dua krte h.. 1 1
2430 Bhai 2 minute 1 1
2431 Bhai sabko reply kaise karoge 1 1
2432 aa jao na....plzzz 1 1
2433 Hiiii.am ur big fan.i wnt ur photograph wid ur autograph.mera qustn apse h ki apki khubsurti ka raj kya h 1 1
2434 Baat khtm krni hai ya khani suru soch lo :v 1 1
2435 suru kar do yaar 1 1
2436 sir 2 mints left 1 1
2437 5 pm bje krenge direct bt mere saat 1 1
2438 Hiii sallu bhai i m ur biggggest fan..........kya aap bataege ki aapne acting konse acting skool me sikhi.....plsss b hai tell me 1 1
2439 bhai hm log wait kar rae h 1 1
2440 Sir life me ek baar apko milna he aapko milna he 1 1
2441 Aap ko letter b likhy hain shayd ap tk puhnch jaye.... 1 1
2442 meri dua aapke sath he 2 2
2443 kaha ho....aap salman 1 1
2444 Bhai aap mahan ho 2 2
2445 Intzar....... ho gye mere salmaan.... sir ki aae na kuch khabr sir ji aapki........ 1 1
2446 Advance eid mubarak salman bhai 1 1
2447 Bhai 4 baj gaya bhai kaha ho bhai? Please reply karo na bhai 1 1
2448 4 bj gye bhai.... aa jao ab 1 1
2449 bhai 4 to bj gaye kaha ho aap assalamu aly kum namste sastrikal adab 1 1
2450 aaj ki party bhaijaan ki tarf se fb par 1 1
2451 bhai apke is blockbuster movie bajrangi bhaijaan ke bare me kuch gadhe na jane kya kya bakbas kr rahe hai au r ahmedabad me to movie ki release pr ban lagane ke liye appeal bhi ki hai ap kya kahoge bhai 1 1
2452 vai may Bangladesh se hu. may chatahu app ek bar ki lia Bangladesh me jorrur aenge 1 1
2453 ok sallu 2mint letest 1 1
2454 Are kaise pata chalega ke online ho 1 1
2455 Sir jodhpur se hu bs wait kruga apka airport pe fir se milna kuch dena bhi j 1 1
2456 plz tell me bhau salu miya ji 1 1
2457 Sbka nhi maloom meri hr dua aapke liye h 2 2
2458 salamin alaykum salman khan jumma bubarakho how are you 1 1
2459 4 bjne hi wali hai 1 1
2460 Salman bhai ap vadio chat kro gy 1 1
2461 Bas ek mnt hai 1 1
2462 hiiii how r u ..aap ye btaye ki aap lucknow kyu nhi ate hai n agar ayeto mjhe plzzz inform kare..plzz 1 1
2463 Kya baat kiya be shashi 1 1
2464 bas 1 min aur 1 1
2465 mujhe kaise pata chega ki aap aa gaye hai 1 1
2466 slman plzz m apse milna chahti hu....apko ek bar dekhna chahti hu..m apki bot bdi fan hu dil se...reaallyy i like u..i like ur natur.. 1 1
2467 bahi 4 baj gaye praty abhi baki hai 1 1
2468 1 min bhaai 1 1
2469 sirf ek Selfie lene hai aap ke sath 1 1
2470 lambi line hai aapke chahnewalo ki.unme hum shyad apko dikhe bhi na :(1 1
2471 Aa jao bhaijaan 1 1
2472 BHAI AAO AB 1 1

2473 Aap bhi bohot handsome lag rahe the aur baachhi bhi bohot cute thi. Charming song. Mazza aa gayi thi. :) :) :) 1 1
2474 Helo salman.sir 1 1
2475 bajrangi bhaijaan 1st day 1st show jaumga main 2 2
2476 bhai kha ho 1 1
2477 Kaha reh gye 1 1
2478 kaise ho bhaijan???? eagerly waiting for 17th 1 1
2479 Aa gaya bhai 1 1
2480 Bhai 4 bajgaye yr.... Ek baar plz 1 1
2481 4 baj gaye par baat abhi baaki h 1 1
2482 haa bhai pls come in vrns 1 1
2483 plz aayeye sir jhuth bole to smjh lena aap ek meri jitni achi fan gwa doge aap sir 1 1
2484 Sal me kam se kum 3 movie kiya kro salman bhai 1 1
2485 hlw salman sir. 1 1
2486 Ase baat karoge aap comments pr?? 1 1
2487 Vai mai dur se baaat kar raha hu :'(1 1
2488 salman mughe aapki ek bat sabse acchi lgi 2 2
2489 baat kaise hogi itne sare log to baat krna chahte hai 1 1
2490 please help me mein fb page par chat kese karu salman bhai ke sath?? 1 1
2491 Bhai kese hum ap se bat karsakeing.. Salman Khan 1 1
2492 Assalaam o alaikum bhai 1 1
2493 4 baj gaye ana abhi bhi baki h? 1 1
2494 Apna bhai sallu bhai d everlasting men :-) 2 2
2495 4 baj gai leken sallu abhi anna bake hai 1 1
2496 Bhai jaldi ao 1 1
2497 it's 4'o clock 1 1
2498 Salmaaannnn pls baat karo 1 1
2499 Bhai plz aao jaldi 1 1
2500 bhai ap boht ache hn Allah ap khush rkhe 2 2
2501 1min jyada hogya bhai aao na 1 1
2502 Salman bhai jai ho. Blessings to bajrangi bhaijann. 1 1
2503 4 pm ho gye bhai 1 1
2504 4 baj chuke h bhaaijaaan 1 1
2505 ok ab mai ja rhi hu byee....jhuth bolw kauwa kaatw kaale kauwe sw aap dariyo salmaan sir 1 1
2506 4 baj gaye bhaijan bhaijan ji :-* <3 1 1
2507 4 bjh gaye sir 1 1
2508 bj gye 4ab aja mere yar 1 1
2509 4 bhj gye lekin salman bhai kider hai 1 1
2510 Kidar ho bhai 1 1
2511 salman 4 bja gye 1 1
2512 Bhai kidr ry ly tu. 1 1
2513 Aap apni selfie le k prfl pic pe set karo kyuki salman khan name k kafi I'd h 1 1
2514 Jaldi ajaw bhai Namaz mey jana hey 1 1
2515 hello bhai ji 1 1
2516 Bhai aa bhi jao ab time ho gya 1 1
2517 dekho 4 baj gaye,phir bhi aana tera baki hai. 1 1
2518 4 baj gae jhute 1 1
2519 4pm ho gye 1 1
2520 kaha hooo vai? ajao or intezar nehi hota 1 1
2521 4 baj gaye lekin party abhi baki h... 1 1
2522 Plz rply sir 1 1
2523 Kaha ho bhai? 1 1
2524 4 bje gae lekin bhaijan ka abhi ana baaki h 1 1
2525 Bewkoof bana rha hai koi .Salman itna free nhi hai. Jo chat krne baithey. Fake account hai yeh 1 1
2526 Bhaijaan kaha he aap? 1 1

2527 Plss suno na 1 1
2528 sir 4 baj gaye aao na plz 1 1
2529 Bhai mein apka buhat bra fan hon pakistan,sindh,karachii 1 1
2530 india me to 4 baj gye agar aap London betha h to pta nhi 1 1
2531 Bhai life mera sirf ek hiii khawb hai... Apke. Sath ek selfieee bhai 2 2
2532 Hello salman bhai... 1 1
2533 bhai dhakka na den 1 1
2534 Kamaal karte ho pandey ji whr r u 1 1
2535 bhai bt nhii krne thii too mna kr dtee mere phn ki bttry bhi low hooo gyee... :(1 1
2536 bhaiiiiiii kaha ho???? 1 1
2537 sar aap chaho to yek bar hum she bat kare 1 1
2538 sare logo se ke hogi bat 1 1
2539 4 baj gye hai lekin...Bhai abhi tak aaye nhi 1 1
2540 Bhai ne fool bna diya sabko hahahhah 1 1
2541 Hlp me bhai plzz help me 1 1
2543 nai ata kamina salman khan sabko pagal banara lolz 1 1
2544 BAJ GYA CHAR 1 1
2545 Hiii salman I form mardin we are love you bolly fans Turkey love you love you come in turkey come in Istanb ul muahhh??? you we salman bhaiiiii I love you muah ????? you are films likeeee? my favorite films chori chori chu pke chupke ?? 1 1
2546 Yr salu bhai jaldi idhr pk m loadshafing hojaegi :D 1 1
2547 bhai kha hai aap 1 1
2548 avi aap ajaona plz 1 1
2549 Bhai n nend a Rahi hai bhut Tez ki 1 1
2550 Kab aaoge aap 1 1
2551 bhai kha ho? 1 1
2552 Bhai.post plz 1 1
2553 Bajrang bali ki jai 2 2
2554 Salman ji wakai aap h ki koi fraud bnata h humlogo ko 4 bj gae h 0 0
2555 bhai 4 baj gaye kahan ho aap 1 1
2556 Salman bhai aap humse kaise baat karonge bhai 1 1
2557 Bhaijaan mamo ya na mano main apka sabe bara fan.. Apke lia sachme jaan vi hazir hai. 1 1
2558 bhaiyya pls baat kijiyega dil mat todiyega 1 1
2559 4 baj gaye lekin party abhi baki hai 1 1
2560 It's 4pm sir 1 1
2561 kya bhaijaan frank kar rahe ho jaldi aao 1 1
2562 Salman Bhai.....Mri Bf mjhse jada apko pyar karti h...or islie mjhe usse bht pyar h kyunki wo mjhse jada apko pyar karti h 2 2
2563 sallu bhaijan.pls aa jo ab 1 1
2564 bhajrangi bhaijan official page me jao baat karne k liye 1 1
2565 Bhai rolaoge kya aa v jao 1 1
2566 bhai aaj ki party ye song film dikao na please 1 1
2567 Ek baar mene jo commitment ki to fir me apne aap ki bhi nhi sunta! ;-) 1 1
2568 tym kbka ho gya...whr r u 1 1
2569 BHAI apka being humen trust kesa chal RHA he 1 1
2570 Bhai 1 bar rply chahiye aapka kha ho sir 1 1
2571 Abhi nai bhai 1 1
2572 apja ek vi film miss nehi karta, kisaka film k lia theatre jau ya na jau.. 2-3 Bar min. Apka film thetre mein dekh ne zarur jataa hun dosto ko leke. 1 1
2573 bhai aagya bhai aagya 1 1
2574 Aaray Yaar koi toh bolo sab comment kar re khali kispe baat karinge bhi 1 1
2575 tym ho gya 4pm ho gaye where ru??? 1 1
2576 Sallu Bhaijaan aapko #BajrangiBhaijaan film se kaunsa gaana sabse zada pasand hai?? ?? 1 1
2577 4 baj gaye bhai aab too aao naa plez 1 1
2578 salman sir plz bat kijiye na hum sb se plz plz 1 1

2579 Bajrangi bhai jan kis type ki picture hai log halla kyo macha rahe hai 1 1
2580 Salman bhai kaha hai 1 1
2581 kya bath hai mere dost kay apmere sath film karoge 1 1
2582 kamal krte ho yar pandey ji 4 baj gye or aap abi tk nahi aaye 1 1
2583 love u vaijaan, always aapki intezar main rahtahu,,aapki ek zalak or aapki movie ka intajer main rahtahu......... Allah aapko khush rakhe or salamaat rakhe esi dua kartahu.....Ameen 2 2
2584 4pm Salman kha ho 1 1
2585 sr ji 2 mnt xtra lay li....ab itne dr apko jyada baat krni.pdegi :* 1 1
2586 Salman time pura hogya ajao na fb pe 1 1
2587 itz 4.02pm 1 1
2588 pls bhai kuch btao ...BB ke bare me 1 1
2589 Bhai sab ko pagal samjha h kha ho 1 1
2590 Are maska kyu mar rhe sallu bhai rply dedo 1 1
2591 Puri duniya se ziyada apko like karta Hon 2 2
2592 Hello koi hai ? 1 1
2593 Ind k 4 bje ya pk k 4 bje ;) 1 1
2594 Ur fav footballer ? 1 1
2595 Bhai apni dusri site pe busy hai shayad lekin yaha aayenge jarur 1 1
2596 Hello bade bhaijAAN 1 1
2597 Sallu bhai so. gyee kyaaa 1 1
2598 Abe ye koyi salman ki real prf thodi hai 1 1
2599 Beingsalmankhan................. Kaa haa ho yrr...... Aaiieytoh sahi :) 1 1
2600 4.....baj gy ....but party abhiii bakiii hai.....vo bhi saallllluuuu miya bhaijaaan ki.... 1 1
2601 1 rply sir plzz 1 1
2602 Chalo shuru karte ha bhai 1 1
2603 Kaha ho tum 1 1
2604 Kitna tym aurrrrr ji 1 1
2605 Sir 4 baj gaye 1 1
2606 Sallu bhai mera tharafse ramzan mubarak ho 1 1
2607 bhaijan kahan ho 1 1
2608 sirr..khan ho...goli toh ni de re?? 1 1
2609 Kaha par hai 1 1
2610 It's 4.3 pm 1 1
2611 Kya karte ho salman yr 1 1
2612 bhai roze se hu jaldi aa hao:'(:'(:'(1 1
2613 Bhai profile pic mxt HAI change mat krna 1 1
2614 itd 4:03 1 1
2615 Bhai 4 baj gye h 1 1
2616 Salman kha ho 1 1
2617 Biryan acchi milti yha in bhopl 1 1
2618 4 baj gae bhaijaan.......... 1 1
2619 Bhai majak tha kya? ;) 1 1
2620 4 baj gaye lekin salman ji nahin aye. :/ 1 1
2621 4 baj gye lekin bhai abhi gayab h 1 1
2622 4 baj gye lekin salman hJi kha h.. 1 1
2625 hello sar g keshe ho aap 1 1
2626 bhai ap sach me humari savalon ka javab denge na bhai 1 1
2627 Kisine mazak toh nai kiya 1 1
2628 Ap ramzan ma rozy rakhty ho? 1 1
2629 Bhai kasam se bata do ki shadi kab karr rahe ho warna mai kuwara marr jaunga 1 1
2630 4 baj gaye Lekin sallu sir ki entry abi baaaki hai.... 1 1
2631 aa jao sir 4 to kb ke baj gye 1 1
2632 bajrangi bhai dar gaye is idd me lagta he 1 1
2633 i hop ap reply jrur kroge..ap apne fans ka dil kbi nai todroge 1 1
2634 Bhai aa jaao ab intejaar nhi hot 1 1

2635 Bhaijaan aapka driving licence bam gaya ya nahi????? 1 1
2636 bhai in sb ko mirchi wali samose khilao bahut chilla rhe h 1 1
2637 ha bhai plzzz sab se pehle mere sath 1 1
2638 are wait kro jaruri thodi na hai 4 bje ka bola hai to 4 bje hi aaye 1 1
2639 Sir kaise milega reply ;-(itne logo mein 1 1
2640 4:5 hoge 1 1
2641 every sec. 300 ppl cmnt 1 1
2642 sir plz plz 1 1
2643 Ek new status pe baat krte haii ;) 1 1
2644 4 baj gaye bhai 1 1
2645 bs refresh krte ja rha hu reply nhi aa rha bhai ka 1 1
2646 Salman bhai zinda baad aur Un k dosuman mordabaad kon kon is se zehmat hia 1 1
2647 4:02 IST where r u??? mein yaha tu wahaa...zindegi hai kaha?? 1 1
2648 hii.....salman bhai 1 1
2649 aaiye na jaldi kabi tho time dijye janta pyar karne wallo ko..... 1 1
2650 sir 4 bag gaye aap kab baat karoge 1 1
2652 chalre sale terishe kon bat karega tu kon hai 1 1
2653 kaha h bhai jann 1 1
2654 4 baz gye...ab to ajao mere bhai.. :-(1 1
2655 Bhai 4 baj gaye kaha ho jaldi ao 1 1
2656 Nhi aya yar 1 1
2657 kha ho tum bhaijan 1 1
2658 kya huaa bhaijan 1 1
2659 hello Sallu bhai , kaise ho aaapp bhai ? mei aapka badaa fan huu bhai :) 1 1
2660 4 baj gaye lekin bhai aana abhi baaki hai ;) 1 1
2661 4:04 over 1 1
2662 Bhai movie main social message kya hai batao 1 1
2663 kisse karenge bro aapke to fan bhi bhut hai 1 1
2664 bhai work hai kya..hume bhi hai thodi der baad but plzz tution jana hai:(1 1
2665 veer ji aa gai mai karooooo start yo yo yo jai salmannnnnn :) 1 1
2666 Arrrey kmaal krtey ho Hm sb wait kr rhey h 1 1
2667 Bhai kitne baje 1 1
2668 Khade khade pak gaya bhai ketna w8 karawoge ? 1 1
2669 Bhai kaha ho aap 1 1
2670 Aapko bachpan se hi apna best hero mana.......aapne na aajtak kisi comment ka reply na likes..............aaj sad hoon main.......aap bahoot bhoot hit do....laken main ab sad hoo..aap mere favroute hero rahoge laken ek baar like ya comment de te to accha rehta ....thanku 1 1
2671 Juz in last 5 mints... 5000 comments and 10000 likes.. Itz juz awsum 1 1
2673 Salman..bhai ..aap dekh rahe ho kya kitne fans aapki wait kar rahe hai..just Hello to kehdo Sabko 1 1
2674 sirji nhi bat karenge ab 1 1
2675 bhai aapko bajrang bali ki kasam aa jao 1 1
2676 salman sir plzzz aa bhi jao its 4:02 1 1
2677 kaha par ho app bhai ? jaldi aa bi jaw 1 1
2678 4 baj gaye lekin bhai abi aaye nahi... 1 1
2679 Salman khan meeting me bzy he kal online aaye ge wo 1 1
2681 4 baj chuke hai salman 1 1
2682 bhai pls kabhi mathura bhi aao na yaha apke diwane bhare pade hai .pls kabhi yaha bhi darshan do apke fans pagal ho jayenge apko dekhkr bhai 1 1
2683 Salman plz cm? 1 1
2684 Aap kahan hai hum aa gaya 1 1
2685 Hmmmm.....tum hare liya agyei hu...Salman :*:* 1 1
2686 Katrina ranvir ke saath hai..tum kya kor rahe ho 1 1
2687 plllzzzzzz baat kijiye naaa i am waiting kab se 1 1
2689 Are sir aj apke liye 4g internet pk dalwaya hai..ab aa bhi jao.. ;) 1 1
2690 It's 4:02 sir 1 1

2691 bhai 4 baaj chukay hai party abhi baqi hai 1 1
2692 kya hua bhaijan..?????????. 1 1
2694 sayed app busy ho..kohi bat nhi hum samj te he 1 1
2695 Yaar baj gaye bhai....reply? 1 1
2696 bhai bolo to sahi 1 1
2697 dabang 3 ? Kab 1 1
2698 kaha ho bhaijaan...??? 1 1
2699 Batao na bhai mene aapse 3question puche hai 1 1
2700 Me kara intjarr tera.. .. 1 1
2702 sir kiya huwa hai aap ko kuch to bolo ek baar call koro na bhai jaan 918399840411 1 1
2703 kaise krenge yrr// twitter pr ya fb pr sum! tell me 1 1
2704 Bhai hume fool to nahi bana rahe na 1 1
2705 Hellooooo 6 mint upar ho gayyyyyy 1 1
2707 lagat hai salman serf aapka he time qeemty hai hamara nahi? 1 1
2708 kaha gaye sir 1 1
2709 wt hapen ab mt bolna 5bje krenge bat 1 1
2710 bhai india my 4 baj gy 1 1
2711 Bhaijan kese ho 1 1
2712 Aa jao bhai 1 1
2713 Areyy baat kaha krni h ye to btana tha 1 1
2714 bhai ap ka hum marte dam tk wait kere gen 1 1
2715 c0mment scroll kar kar ke hairan hogaye hum 1 1
2716 4 baj gye sir 1 1
2717 Bhai apni har ek film mai atif ka 1 toh gana jaroor daaalna 1 1
2718 Sir bhajrangi bhaijan....sabse achii film hogi.....boollywodd mein. ...I wish 2 2
2719 Mein apke sath jacqueline ka b fan hun 1 1
2720 Bs kya bhai 1 1
2721 4 baj gaye.....par party aabhi Baaki he....sallu bhai 1 1
2722 Salu bhai mary yaqen hy ap mary ko lft karawogy. 1 1
2724 Nhi mili kick 1 1
2725 bhai bhauiiiiiii khaaa hooio 1 1
2726 Ek baar maine jo commitment kar di uske baad apne aap ki bhi nhi sunta..... 1 1
2727 Sir it's 4.o6pm 1 1
2728 aaj Facebook pe lagta h sabse jyada comments ka worl record ban jayega 1 1
2729 Subah se 3:30 ka wait kar rahe hai, aapne 4pm bhi ho gaye.. Ab toh aa jao aap plz plz 1 1
2730 Star logon ke nakhre yaho hote hai abhi inka 4 nahi baja 1 1
2731 yes actully its 4.06 .. 1 1
2732 Salam sucuk sosis salman 1 1
2733 sone do ...aajayenge apne aap 1 1
2734 Kahan gayab ho gaye???? 1 1
2735 kya hua sir? 1 1
2736 hallo bhai jan kaise hai mai aapka bahut bada fan hu 1 1
2737 Salam bhai Jumma mubarak 1 1
2738 bollywoood ki shan apna bhaijaan 2 2
2739 Hlw aapko nhi ana to bewkuf to mat bano 1 1
2740 Aise mat karo yaar...plzzz 1 1
2741 Don't worry bhai aap aaye nhi toh bhi chlega 1 1
2742 Bhai kaise ho? 1 1
2743 bhai ap Hollywood movie kab kar rahe ho??? 1 1
2744 Bhai kya mazak hi ye 4.6 ho gye 1 1
2745 bhai helo hi bol do... 1 1
2746 kabhi to waqt pe aya kro salu miya.......... 1 1
2747 Bhaijaan kaha ho aap..##plz baat karo..## meri beti aapki bahut bari fan hay..## 1 1
2748 Nhi aayege woh busy promotion krne mein .sunday ko kapil ke show mein jao. Face to face 1 1
2749 hi salman vai...... 1 1

2750 4 bj gye hai kb aaoge online 1 1
2751 Aby bhai sarfaraz t hy dhoka nae dyga. 1 1
2752 Samir Shaikh Shayd bhaijaan ne mazak kiya :-D 1 1
2753 heloo sallu bhaaaaayi 1 1
2754 Bolo sub hahahaa 1 1
2755 i cnt wait moree plzzzz comee :(1 1
2756 Bhai jldi aa jae 1 1
2757 Sab kya hoga 1 1
2758 bajrangi bhaijaan all time blockbuster 1 1
2759 4 baj gye bhai 1 1
2760 Bhaii aao na kha ho 1 1
2761 #salu bhai ap bas bachio ko reply kartay ho :p 1 1
2762 abe salman khan yaha logo ko bewakuf bana raha he aur log ban b rahe he per me nahi banoonga samjha be baj rangi 1 1
2764 bhaijan kaise ho??!? 1 1
2765 Aja 've Bhai tera rastâ€¦ udheek de ha? 1 1
2766 Bro ap to baat karo 4:00 kap tak baj gya 1 1
2767 Chutiya banadiya sab ko 0 0
2768 ok mere saath phn pe baat e karenge?ya whats app pe?boliye jaldi... 1 1
2769 Baby now itz allready 4.06 com naa waiting .. 1 1
2770 Bhai aap may bara fan hu please aap number mujhe mallum nhi 1 1
2771 itna wait to kbhi GF ka b nhi kiya 1 1
2772 Hello...salman khan ji.... 1 1
2773 Ok sallu bhai 1 1
2774 bhai kaha ho ap 1 1
2775 But bhaijaan Ji , aapki movie Ki shooting ka kya ?? Prm rtn dn pyo! 1 1
2776 Hi salman bhai...kya hal hai apka 1 1
2777 Sir ji app ksy hoo???Hmlogo k apk flim k bauth din sa intyjar tha so..........Ap k film bajrangi bhaijan jarur dkh gy....We LOVE YOU.SALMAN Sir.... 1 1
2778 Bhai aap ke may bara fan hu please aap number mujhe mallum nhi 2 2
2779 Mujhe sirf itna puchna hai KiAap har waqt itne log aur bodyguardWe ghire rehte ho hum jese aap KOKaise mi l sakte hai???????Please give answer. 1 1
2780 Hiii salman bhai 1 1
2781 I know bhaI jaan ka hamesha ki tarha net khrab ho geya hai 1 1
2783 are kya hua bologe bhi kha ho 1 1
2784 kaha ho vai...kuch batao...kaise ho..... 1 1
2785 hey mery inbox mai msg agay salman ka yahooooooooooooooooooooooooooooooooooooooooooooooooo oooooooo\ 2 2
2786 Ab aa jao sir 4 bj gye hm wait kr rhe h 1 1
2787 Kitthe reh gaye sallu ji 04:07 ho gye 1 1
2788 Bhai.. Kese ho.. Aur intezaar nhi hota jaldi aao 1 1
2789 bhai 2 din se wait kar raha ho aab to comment karo 1 1
2790 AaP nahi aaoge 1 1
2791 Hy salman bhai 1 1
2792 Salman bhai s phon no looo yarrrrr 1 1
2793 salmaan# bhai# aap# hamare# chahete# kalakar# ho# aapko# dil# se pasand# karte hai but ham sab ki dil se ta mnna hoti h aap se mil sake or apka didar kar sake 1 1
2794 I love you. I fill you. I think you and marnasa pahla apsa 1bar milongga jaroor. I'm from Bangladesh. 2 2
2795 plzz slman ap jrur reply krna hm apka w8 krre h 1 1
2796 sab bekar h..... koi nhi aane wala ..... salman nhi aayega 1 1
2798 Ap late ho ap ne apne cometment pori nai ki 1 1
2799 sallu bhai.... kaha ho thum?? 1 1
2800 Apki PA team ki watch kharab h shayad...4 toh baj gaye..:-(1 1
2802 hm baarish ho rahi hain to netwark problem to hogi hi 1 1
2803 Salman khan pagal hai 1 1

2804 lgta hai bhai ka watch slow hai ;-) 1 1
2805 Sir pleaz jalde ao 1 1
2806 hi salman sir apka dhan kidhar hai apka fan idhar hai#akki 1 1
2807 kaha ho...ji salman ab to kuch bolo na 1 1
2808 jumme ka din hai, chatting ki baat hai. allah bachaye mujhe, fake pages se. 1 1
2809 Bhai jaan . aa bhi jao .. 1 1
2810 sir pls ek reply de do....main to ish khushi se hi pagal ho jaunga....pls sir,pls..i beg to you. 1 1
2811 bhai plz 1 br bat kr lo 1 1
2812 Bhai tym ho gya... 1 1
2813 kia h0A sal00 bai.....? 1 1
2814 Abitna bhi kya sharmana 1 1
2816 Aa bhi jao ab itna late kyu kr rhe ho..wait kr rhi hu salmaaan sir ji plz kb aaoge online aajo aajo 1 1
2817 Main apse milna chahta hun 1 1
2819 Bhai kaha ho aajao 1 1
2820 Bhai 4.10 hua jaldi aao na 1 1
2821 are yaar bhai se baat hogi ya nhu 1 1
2822 It's 4:10 1 1
2823 Fav..food kya hai 1 1
2824 sallluuuuuuuuuuu aa bhi jao :'(1 1
2825 Bnai aap keh rhe ho ki me muslmano ke bina bajragi bhaijaan hit kroga yhe sach h.. 1 1
2826 arre ap to aaynge nhi hm ja rhe h..... 1 1
2827 Aaja sham hone aayi .mousam ne li angdai. 1 1
2828 4 indian standard timing means 4:15 ? Pranam bhai ? .. 1 1
2829 A bhi jao mr.salman kaha ho 1 1
2830 bhai apne 3:30pm bola tha fir 4:00pm bola ab 4:10pm ho gaya hai .this is not fair 1 1
2831 kya hua...bhaijaan.... 1 1
2832 Bhai jaan kha ho Salman khan 1 1
2833 sir ji ye achi bat nhi he ki aap apne fan se majak kre 1 1
2835 Are Bhai ko kuch bat bolo. q ke hum ya pe bohat sare fan ho . is k liye aanahi raha hai. waise b bo kitne k sath bat karenge. 1 1
2836 salmaan sir hm aapse milna chahte hai plz sir... 1 1
2837 Salu bhai awo na. 1 1
2839 4 pm to kabka baj gai ...pr aap ho kaha Bajrangi Bhai jaan 1 1
2840 kya bhai kab aarhe ho 1 1
2841 Helo..bhia kaise ho...!! :) 1 1
2842 bhai aao n 1 1
2843 aur aap kaise he 1 1
2844 Salmannnnn aaa intezaar idhar ? tere liye ? 1 1
2845 Aajo ji ab 1 1
2846 Bhai roja ramdan me weit nhi karwate 1 1
2847 4.10 ho gayi ab to :(1 1
2848 nhi aoge kya bhaijan 1 1
2849 Ab to aa jao jnab ...plz aajo 1 1
2850 jb talak ap na aaoge m na jaunga FB se 1 1
2851 bhaijaan 4:11 ho gya. kb aaoge aap 1 1
2854 kaya huya, kaha hai app,hum logo ko murga to nehe banareha hai? 1 1
2856 Ek saath bulao sab.... 1 1
2857 Hlp kr do mri plzz bhai 1 1
2859 Theek hai bhai bahut hua ~~ 1 1
2860 BHAI ab intezar itna bhi karvai ye 1 1
2861 Kmal krty ho pandy g itna sara vett?? 1 1
2862 u r muslim soo app rozay rakh rhy hoooo 1 1
2863 yeh salman khan ki purani aadat hai.... late aana :p 1 1
2864 ma na ja ohir bhai ko post office behja ha ap ko kch snd karna k lia.magar pata nhe apka adress tak nhe pata ya pir kia waja ha 1 1

2865 Bhai kuch to bta 1 1
2866 Dabang kha ho 1 1
2867 Ajao bhai jaan kha par ho 1 1
2868 m apki bot bdi fan hu 2 2
2869 Salman bhai plz mene apna gf ko bola dat u vl Say hello 2 me plz mere ijjat uske sAmne bane rhe uske lye muj he Hello Ajay bol diye na plzz apka pair parta ha 2 2
2870 dhoka de diya bhaijaan.... 1 1
2871 Hero ki entry last me kyun hoti hai abhi pata chala 1 1
2872 Pavan kuman ...abhi toh ajao.. 1 1
2873 Bhai aap kaha hai 1 1
2874 BHAI ab mujse aur wait nhi hota aapka wait 3.30 se kr rha ho 1 1
2875 aap ache ho na ? body shody bana rah ho na 1 1
2876 Wait karana achi baat nahi hoti hai bhai.... Bhai sirf rona baki hai 1 1
2877 Bhaijaan kamaal karte ho 1 1
2878 try kariye ap mese kisi se to bat kar hi lege 1 1
2879 plz pakistan si r kaisa behjon 1 1
2880 Unke namkaran ke time sab jan the kya sallu sallu what???? 1 1
2882 Kha pr baat kre 1 1
2883 bhaijaan kha ho 1 1
2884 Chalo.kuch nii bhai koi kaam me fas gye honge aap 1 1
2885 luv u sallu..:-* 2 2
2886 Sallu bhaai i am w8 4r bajrangi bhaijaan.. 1 1
2888 Bhai ap nai ayenge tab-tak natural call bhi accept nai karunga?? 1 1
2889 Bhai aao na fir 1 1
2890 salman bahi uar best 2 2
2891 bhai apka ghadi slow hai kya 1 1
2892 Hello sir ji salmaan ji itna late kyu ho rha hai plz jaldi reply kro plz i m waiting for ur response 1 1
2893 Salu bhai main tek hun ap suna wo kia kr rhy ho. 1 1
2894 Salman best of luck for bajrangi bhaijan....iss baar bhi sare record tod degi...inshallah 2 2
2895 4:00 baj gya sir 1 1
2896 Jaldi ago bhai 1 1
2897 SALMAN KHAN BHAI CHAR BAJGYE PAR PARTY ABI BAKI HAI 1 1
2898 Salman bhai nahi ayenge 1 1
2899 apko ana padega 1 1
2900 Bhai maa aap ka sabse bada fan puri duniya mein aur bhai jodhpur sha ho 1 1
2901 Kis kis ko jawab dungachalo ek lucky draw karta hu 1 1
2902 or kitni 4..., 1 1
2903 Arey saab apse to hmm ptivate mai milunga? 1 1
2904 Hum dil de chuke sanam.... 1 1
2905 Eid ke phle realese mt krna sir jee.. 1 1
2906 Lub ju bbye 2 2
2907 Bhaijan mere bhabi ko or kitne bakhath samalkhe rakhogeJara mujhe bhi dekhna hay... :) 1 1
2908 sorry frnds bhai nahi aa sakte 1 1
2909 Upar waale ne insaan ko sbse upar kyaaa diya Dimaag diya hKyu diyaaa h Sochne k liye 2-4 gf side me rkhne k liye 1 1
2910 Agar bhai ka 1st day 1st show nahin dekha toh yeh jaan rehne ka kya fayda 1 1
2911 Bhaiiii jaaaan waiting fr uuuu....:(:(:(:(:(:,(:,(:,(:,(1 1
2912 bhayiaa u lukin so superb at movie Bajrangi Bhaijaan,,Aise hi hamesha hum fans ko pyar dete rahe.Itna hi kaaf i hai mere liye.Love you bhayiaa 2 2
2913 Apna liya toh saab jetha hai dusra kai liya jina wala koh farista kahatai hai 1 1
2914 abi bhai ko pir behja ha apko kch snds karna k lia pata nhe apka adress tek nhe pata mja 1 1
2915 Nhi arah bhai 1 1
2916 abhi tk kyu nhi aye aap salman sir??$$$$ dieing to tlc to u....... 1 1
2917 3.30 se 4.00 4.00 se 4.30 aana he ya nhi 1 1
2918 Comment padh ke thak chuka hu aa ja re bhai 1 1

2919 aslaam o elekum bhai iam from pakistan kia ap bajrangi bhai jan k liye pakistan ana pasand krengy 1 1
2920 Hloo salu bhai...... 1 1
2921 bhai mere se ek baar 1 1
2922 aap se itna pyar karte bhi 1 1
2923 kab aarahe ho 1 1
2924 Mujhe aap se ye umid nahi thi bhai 1 1
2925 Salman bhai plz ek baar muje call karo 7048830072 plz plz bhai fir muje mout bhi aa jaye gi to chalega bhai 1 1
2926 are sallu bhi apki ghadi slow h kya?? 1 1
2927 bhai jaan plzz come 1 1
2929 mere pita ji bombay me rahte hai unhone ek bar aapse madad magi thi ek blood cancer k pationt ki madad ki au r aapne ki thi 1 1
2931 Bhai Goli de rahe ho chotey bhai ko 1 1
2932 Bhai jaan jago .4.13pm ho gyaa kaha ho 1 1
2933 Sayad bhai bz Honge is liye ni aaye bhaijaan aayenge jarur ek bar bhaijaan ne commitment kardi to kardi 1 1
2934 sallu mia bhul gye sab kuch yaad nhi unhe ab kuch 1 1
2935 4 baj gaye hain 1 1
2936 Bhai jaldi aa subi se waiting 1 1
2937 Aayenge salllu bhai jorror ayenge 1 1
2938 salmaaaan........bhaijaaaaannnn...jindabad 1 1
2939 Watching bajrangi bhaijaan 1 1
2940 salman bhai abi dabanng 3 ke shuts ke liye satara mai hai, so next time fans 1 1
2941 4.13 now 1 1
2942 vai jan ek ber batyan e koro 1 1
2943 4 baj gaye sir .,.,.,.come plz ,.,.,.,. 1 1
2944 Hii bhaii.. Aap kese ho..?? Agar aap.mera cmmnt padh rhe ho toh plz ek reply kar dena 1 1
2945 bhai ne kaha hai ki wo busy hai....6 bje tak 1 1
2946 Bhai me aap ke film ka intajar kar raha hu 1 1
2948 Abe kha saitan ke cakkr me pade ho 1 1
2949 Mujhko prr ek ehsan krna k mujhse kv shadi krne ko na kehna :) 1 1
2950 Bhai aap mazak a6a kr lete hai.....Â¡ Â«Â¡?Â¡ Â«Â¡?Â¡ Â«Â¡?Â¡ Â«Â¡?lov u Bhai..... 1 1
2951 kuchh kam hoga re un ko bhi 1 1
2952 yrrr aao na jaldi 4:14 bhi ho gye 1 1
2953 chalo sab apne apne ghar koi salman nahi aaega....!! 1 1
2954 kahi aisa to nhi bhai ki aap hume direct ye news doge ki aapki shadi bhot saal pehle ho chuki he n wo apne top secret rakha hai??? 1 1
2955 aapka har andaz hi anokha hai janaab 1 1
2956 Bhai aap apna number dijiye salman bhai 1 1
2957 5000comment in 5minute 1 1
2958 khd ana to mumkin nhe shayid 1 1
2959 Pahle 3:30 phir 4:00 and ab 15 min over bhai 1 1
2960 R e bhai itni assan hoti salman khan ke sath bate kar na to hochuki o itni busy ha o thorai kisise bat karchakti h a keya?????? 1 1
2961 kamal karte ho panday ji hume bula kr khud hi gayaab 1 1
2962 i lv u urmy fav star koi nai ap sa proud of u i lv u 2 2
2964 sir wanted ki sequel kab aa rahi sir tere naam ke baad wanted ke baad dabang ab bajrangi bhaijaan 1 1
2965 Sallu bhai patiala a jao apni film ki promotion ke liye... 1 1
2967 sabse hatke sawal, aapko bajrangi bhaijaan kar k kaisa laga? 1 1
2968 Bhi kab awogi do din sa khaya nai pleace bhi aa jao 1 1
2969 Madharchod salman khan randy ka ladka harami kafir 0 0
2971 Ab kaha gye aap gayab 1 1
2972 allah hafiz....salman 1 1
2973 lol bhai kb aayoge muje lgta h server krash ho gaya bhai 1 1
2974 Sir hum Aapka wait kar rahe hai please talk 1 1
2975 Mujse baat karne m tumko sharam aati h sab k saamne 1 1

2976 Aap reply karenge....? 1 1
2978 Bhaijaan kahan ho..kat ki sath hai tum 1 1
2980 abhi tak nahi aye salman bhai online? 1 1
2981 Hello bhai plz rply me once plzzzzzzHello bhai plz rply me once plzzzzzzHello bhai plz rply me once plzzzzzz Hello bhai plz rply me once plzzzzzz 1 1
2983 ab aaenge aur...good evening bol.k good bye kr k chale jaayenge 1 1
2984 Ya fir Facebook crash kr denge 1 1
2985 rolo ab to 4.15 kya yr aana nhi he kya aap ko 1 1
2986 kya bhai aap bhi fak rahe ho 4.14 pm ho gaye abhi tak apne koi rep nahi diya bhai aisa mat we trust you...Ever y time 1 1
2987 Arre salman bhai kanha ho app 4 bajke 15 min ho gaya 1 1
2989 Kamal krte ho pandey ji kha ho aap 1 1
2990 bhai plz aa jao 1 1
2991 Jaldi aaow bhai 1 1
2993 salman bhai sultan film ke shuts chalu hai abhi 1 1
2994 Baat kidhr hogi 1 1
2995 kamaal karte ho 1 1
2996 Bhai k sath baat karne ko taras raha hu yaar 1 1
2997 han #nadim_shaikh ki amma aaegi.. 1 1
2998 Salman bhai...suna hai ap ko kamar k takleef hai..allah ap KO jald sihat yab kare..AMEEN SUMA AMEEN 1 1
3000 Pls pls pls reply ones... 1 1
3002 Bhai shahid ki bhi shadi ho gai aap kab kar rahe ho 1 1
3003 voo pajii tusi kitee chale gaye c 2 2
3007 AISA MATT KAROO 1 1
3008 mene socha tha ki aap humesa such bolte he aur bhut acche h but aap bilkul bhi essaaa nhi he 1 1
3009 Bhai bhuk lagi khana nahi khane jara aap ka wait kar raha 2 2
3010 Salman Khan Facebook py nh hen. Unho ny apny interviw me kaha tha. 2 2
3011 Sala.. Terko dusron k time ki kimat mi hai.. 1 1
3012 hiiiiiiiiiii sallu bhai im big fan sallu bhai aapko twitter par roz mai hi massage karti ho im big fan sallu bhai re ply plzzzzzz 1 1
3013 Bhai plz marry 1 1
3015 4 baj gye lekin meeting abhi baaki hai 1 1
3016 mujhe lag rha h koi admin ham sab ko dhokha de rha h.. 1 1
3018 Jaldi kar bhai 1 1
3019 salman bhai am ur big fan.... am from paaaakkkiiissstan.... plzzz plzzzzz plzz plzzzz rply me one time..... plzzz zz waiting 1 1
3020 Jaise#bajrangi k seena hmko #HANUMAN milanga.hum sbka dil ma sirf#SALMAN milanga. 1 1
3021 Bht ho gya wait ab baat krni ho toh inbox kr dena 1 1
3022 i luv u bhai. . .jo hate krte h plz msg na kre. . .becoz bhai ki olwys rite. . Wish u a bst lck #salman_bhai 1 1
3023 I think world big.great fan of sall bhai is me..Bhai ka dost mera dost.Bhai ka dusman mera dusman.Bhai ka bro mera broBhai ka sis mera sisBhai ka wife mera....vabi :D 2 2
3024 Hi gyz me salu hun roop badal k aya hun 1 1
3025 Bhai aao na 1 1
3026 loveee u saluuuu 1 1
3027 Bhai. Shahid ki shaadi mein nai gaye. ? ?? 1 1
3028 as salam alaikom :) aur intezar nai hota plz cm fast 1 1
3029 Susila maa g ko sat sat naman jo aap jaisa superstar india mai hua. ...salute susila maa. .. 1 1
3030 Ok tjeek hai nhi aanahai to bta to.do sir 1 1
3032 bhai bihar ya patna kb aoge pls reply 1 1
3033 oye bhen ke bhaiyyo.. sallu bhai ki koi chiz fek nahi hoti.. samjhe kya 0 0
3036 Ye jhut hai. 1 1
3037 nirash kar diya 0 0
3041 Hlw Bhaijan.Bajrangi Bhaijan ko le kar apka kya opeksha hai?Is film me aap hanuman jika vokt ho ap kay real zindegi me hanuman jiko mante ho? 1 1

3043 kya hua bhAi 1 1
3044 ab aa bhi jao 2 2
3046 Bhai kamal k jodi hoge ap 3nun ki. 1 1
3047 Bhai ko mat bolo wo buzzy hoga aajaiga 0 0
3050 bas ab ek hi baat h kolkata jldi ana plz 1 1
3054 hlw sir kha ho baat kro na 1 1
3056 sir me aapke apartment ke bhar 7 din se morning to eve khada tha aap ko dekhne pr me dekh nhi paya 1 1
3057 tu sisters aur brothers bahut pyar karte? 1 1
3058 jb krna hi ni tha to bole qq 1 1
3059 Aaaa bhi jaa.. Aaaa bhi jaa... Bajrangi bhaijaan aaaa bhi jaa 1 1
3060 Profile pic to change kro 2 sal ho gye hm aapke naam ki daily chang krte h 1 1
3061 ho gyi party sabki ... ab aaram karo...bhai so rhe ha... 1 1
3063 bhaii ab avi jao 1 1
3064 bhai Mujhe Apna twitter account password de doo pls :p :p :p :p :p :p 1 1
3065 Sallu 4 Baj gaye hai aja o na 1 1
3066 Hum cnfused ho gaye kya aap baat karenge au hum off line hye jai. 1 1
3067 bollywood prem bollywood fitness pleyar bollywood king bollywood god 1 1
3068 plzz m apke rep ka w8 krri hu or krti rhugiii pkkkaaa 1 1
3069 4 : 19 ho rhe kha ho bhai 2 2
3070 Salman khan kaha ho? 1 1
3072 #Pandey ji kaha h app hum log kb se wait kar rahe h ap k #swagat k liye 1 1
3073 Kya bhai kahi koi item toh nahi mil gayi kya 1 1
3074 kamaaaL karte ho salllu ji.....rply to bnta h n.... 1 1
3076 salman khan kamine kab ata tu chor lolz 0 0
3077 Shayad bhaijaan aap busy ho koi baat nahi khuda Hafiz iftaari ki tayyari karni hai Allah Hafiz bhai 1 1
3079 Aaj kisiko reply nai milne wala Salman sir ne ullu bnaya 1 1
3080 Salman kab aate hai kab jate hai kisi ko pta nhi chalta 0 0
3081 hum to aapka swagat krne ke liye kb se aaye hue hai pr aap hi nhi aa rehe 2 2
3083 Salman sir apke liye Puri life wait kar sakte h 2 2
3088 plz bajrangi bhaijan ek reply plz   mai m.p. ke rewa saher ka rahne vala hu 2 2
3089 madat karo khusi milega app ne bolatha but madat karene se dukhu badha jata he kay karu bhai.???????? plz re
ply bhai 1 1
3090 Bhai ...4 <= baj gaye h ?Kaha ho aap 1 1
3093 saaaaaavdhan salman bhai ko bulane ka formula laaya hoon... sirf 51 rupe do or sallu bhai se baat karo.. baba k
a prasaad lo.. sirf 51 rupe 1 1
3094 ye salman khan ki real id h ya sab fake h salman sir ko inna tym h ki wo aae or hum s baat kare i think its fake
1 1
3096 bhai mera rply jarur krna pehle hi bta du ;-) 1 1
3097 bhai mera rply jarur krna pehle hi bta du ;-) apne khud hi bola hai main sare comment padhta hu ye v pdh lia h
oga apne :-D 1 1
3098 Bhai ao na 1 1
3100 mujha bhi karni ha baat par kaisa karu ???????? 1 1
3101 dSir kya mujh gareeb ko aik reply melay ga plz ....? 1 1
3102 ab kitta wait krwaoge 2 2
3103 facku jaldi warna ???? 1 1
3104 Aya reply aya bhai ka 2 2
3105 so gaye kya 1 1
3106 kya sallu bhai baat krne ki bola lekin reply to kisi ko kr hi nhi rhe 0 0
3107 Bhaijaan aap kaa ek reply mere liye bohot important hai kyuki mai aapka bhakt hu 1 1
3111 Bhai aap bimar ho kya????bolti bandh kyu haii?? 2 2
3114 Sir plz reply us ... Hum kab se rah dekh rahe hai apki :(1 1
3115 kamal karte ho pandy ji ....kitna wait karwaoge 1 1
3116 q jhoot bol raha bhai 1 1
3117 4 bhj gye but aap kahan ko jnab :(1 1
3119 bhai mujhe aapki film bajrangi bhaijaan ke gaane bhaut pasand aye 2 2

3120 Main apka bohot badaaa fan hoon.. we luv u bhai 2 2
3121 bhaijan ke kamar pe dard hn doctero ne araam karne ko bola hn ok frendz 1 1
3122 4 bj gaye par party abhi baki h..Baat to dur kisi ko rply bhi nhi kiya Salman Khan 1 1
3124 Bhai kuch kaam se busy honge wo jarur aayenge 1 1
3126 abhi 4wje nahi aap ke????????????? 1 1
3129 Sorry frndz salman sir thore bzy hain abi aaty hain 5 mint me. 1 1
3130 jb aana hi ni tha to 0 0
3132 Sir kya aap fir kashmir aavoge plz reply 1 1
3133 mey Facebook par...Twitter pe nehi...aooo na.. 1 1
3134 O pagal ye asli salman khan thori hai :-) 0 0
3135 salman bhai kb se apka wait kr re ky huaa taik tu me iloveu salman bhai 1 1
3136 Itne fan's ko nervous kar k aap so kaise sakte ho 2 2
3137 Aik he q bar bar repeat karna b acha nhe lag raha 1 1
3138 Ab aa bhi jao bhaijaan 2 2
3139 bhai aapke answer k liy boht piche jana pdega 1 1
3140 sir ji apka reply nahi dikh raha.... :(1 1
3141 Ary bhai love. Phr jb b awo gy aunga. Ap ko request byjta hun link mel gya. 1 1
3142 plzz aajaao yar....itna enry roza rkhne me ktm nahi hoti..jitni apka w8 krne me ho rhi h....plzz come na.....plzzz z.... 1 1
3143 Magar ap bata he nhe raha ha 2 2
3144 bhai 1 reply asssalamalaikum 1 1
3145 Aa bhi jaa sanam â€¦â€¦â€¦ U na kr sitm â€¦â€¦â€¦â€¦ love u salmaan â€¦â€¦â€¦â€¦â€¦plzzz jldi aa jaaao â€¦â€¦â€¦â€¦ :* 2 2
3147 Kamaal karte hoon salman ji aane ka irada hai ya nai 1 1
3148 Biceps 17 inchi banane ke liye kya kare.give me some tips. 1 1
3149 Yes sab jhuth hai koi salman khan humlogo se baat nhi karne wale 1 1
3153 Salu phone numbe ka sawal hai baba :P 1 1
3156 ohhh bhot intezar ho gya .....its 4:23sir.... 1 1
3161 kya yaar! aaja shaam hone aayi mausam ne li angdaai! to kiss baat ki hai Ladai..............:p whre r uh? 1 1
3162 Bhai jaan come plz 1 1
3163 salman sir aap apna ek kisi film mein Internationl rockstar Imran khan ko Singing ka mouka dijiye 1 1
3165 kaha pe chat karna bhai 1 1
3166 Bhai log maine abi suna newz me salman bhai bole jitne v mri fan hn wo Nrzz na ho I'm just coming esa hi bol e.. 1 1
3169 Plz mera dream true kardona bhaii 1 1
3170 ab himmat nai hai re baba jaldi reply krna shuru karo 1 1
3171 sir main bohut gorib hu....plz muje ap ki pas bula lo..........? 1 1
3174 salman khan ....being human ka aslee avtaar. admin aamir ahmed. 1 1
3176 aree salman sir ek baar jo committed phir wo apne aap ki bhi nahi sunte kyun sir 1 1
3177 ghar pe milne aavo.time bolo 1 1
3178 kaha gye bhaijaan??? 1 1
3182 Ap tak pakistan si snd ki hov cheez pohnch jata ha ya nhe 1 1
3184 Bhai ek bar hamse bat karlo cuz hame ek achievement mil jayega apse bat karne ka 1 1
3186 w8 is ovr 1 1
3188 hello #salman #khan 1 1
3191 Sir kya mujh gareeb ko aik reply melay ga plz ....? 1 1
3195 bhai my phone discharged ho raha hai 26% bettry bachi hai 1 1
3196 Sir aapk accont se to maajak nahi kar raha 1 1
3200 bhai aai jao tame badi door gaau se aako betho hu.. 1 1
3202 tm aao na aao hum intzar krenge tum chaho na chaho hum had se v jyda salman ji tmse pyar krenge 2 2
3213 love u bhai tumhare liye ye jaan bhi de sakta hu..Love u bhai jaan sallu bhai.. 2 2
3215 Kyun reply nahi karr rahe ho??? 0 0
3216 Bhaijaan love u Bhai.. 28th july ko mera bday h.. Ek wish kaardo bhai.. Plz bhaijaan.. 2 2
3220 Salman bhai apne apne page pe apna trailer ni dla youtube wala 0 0
3221 i knw ni aoge sbko ullu bna diy ap aj acha khasa 0 0

3222 Watch kharaab hai kya apki??? :'(:'(1 1
3223 bhai fans ka dil na todo aa jao 1 1
3229 Aajao aap vai 1 1
3235 Aapne apna commitment nhi nibhaya Aapka dilouge wrong hai 0 0
3237 Meri bhi kr dijiye na plz.......mera bhatije ko thik kr dijiye ....apko allah ka wasta.... 1 1
3240 Salman khan on instagram 1 1
3241 add karo ji hamre b koi jo.add karega salman khan usi.ko.msg karega 1 1
3242 Aaaaaaa jaaaaaoooooo bhaiiiiiiiiiiiiiiiii knha busy hooooo ham wait kr rhe h 1 1
3243 Abhe 2 mint han yaar 1 1
3244 Bhai 27min 1622 second ho gye Plezzzz aa jaao 1 1
3245 Net pack khatam ho gaya..paytm se recharge karo.. 1 1
3249 Fan k saath Aap majak toh nahi kar rahe hona? 0 0
3252 Hii 4 bj gye h 1 1
3254 Ã–nline hoya nahi 1 1
3255 Newaz bhai favr8 song raat ko aunga mein comment karunga mein questions bhi puchunga mein 1 1
3259 sallu bhai...ek bar cl kr do bs....plz.... 1 1
3262 yo sir Ji mera tuition ka tym ho gaya...plz reply 1 1
3263 4,30p.m ho gea ab to fb pe aooooo. 1 1
3264 Tek ha ap is.waqt bht bzi ha 1 1
3265 Koi mujhe batayega ye fake news kisne dali 0 0
3267 Bajrangi bhaijaan 4:30 b baj gaye Kb aaoge.. Bhai kl Mera birthday.. Kal ki party meri taraf se 1 1
3274 bhai ap bhi hamare desh ke mantriyo ki tarah late aaoge kya 0 0
3275 i love u sir mujhe aap bhute acche laghte ho 2 2
3276 kha chale gaye bhai.. 1 1
3277 Bhai ajao yaar 1 1
3283 kasiy bt kary gy salu sb? 1 1
3287 Bhaijaan aap nahi aaoge toh, apke name pe high court mein case kardungi 0 0
3288 lag rha h bhai aise ni aayenge 0 0
3291 Hum aapna kaam chodkr baithe h . Aajao na. 1 1
3293 How's ur back pain ryt now????? I m tensed....btao na plz salman aap to khe rhe the ki 4 pm baat kroge but aap to 1 bhi cmnt ka reply nhi diya plz is waale ka de dena plz... a request 1 1
3294 kidher hoo sirrrr 1 1
3295 I wish Aallaahh apko hmesha kush rkhe Salamat rkhe....love u 1 1
3299 Hi sallu baba 1 1
3304 shyad aap busy ho tbi aap rply nhi kr rehe ho nhi to aap jrur rply krte...bt m tb tk wait krungi jb tk aap rply nhi krte... 1 1
3305 Salman vai aap sadhi krlo plzzz... 1 1
3307 salman khan ma ap ka bohat bara fan ho 1 1
3308 Sir aap itna hi kah diye to kafi hai ...:-) 1 1
3309 I mean I am thinking dat ki prdp tak yaha hi rul jaau ok?? So plz 1 reply for plzzzzzzzzzzzzzzzzzzzzz?? I am ur die hard fan bhai plzzzzz 1 1
3310 Chutiya bna rahey ho kya bhai..:p boley to lucky draw nikal k baat karogey kya! 0 0
3312 are bhai...kese kare bat ye toh batao koi.... 1 1
3314 Assalam walaikum bhaijaan kaha ho aap 2 2
3315 Roza ka time bhi hora 1 1
3317 4 Baj gaye lekin party abhi baki he 1 1
3318 Bahot ho chuki hain teri dillagi chale bhi aao abb shyam dhalne lagi.. :-) 2 2
3320 bhai,tu kaha hain 1 1
3325 OMG salman bhai... Assalam o alaikum :) 1 1
3328 Sar modlig to nahi ki maine par ek din ballywood ka sitar zarur banuga 1 1
3330 4 baj gaye but rply abhi baaki hai 1 1
3333 bhai bhopal kab aa rahe ho 1 1
3338 Plssssss avi jao ne sallu 1 1
3340 tu hai k nhiii....tu hai k nhiii <3 1 1
3346 Mene bataya na ap logo ko ke aj bhaijaaN bat nai kar sakte kamar pe dard hn docter ne araam ka kaha hn ok fr

endz 1 1
3347 ye kya baaat hoti h ? 1 1
3348 bhai aa jao nahi to katrina ko bol dunga;-) ;-) ;-) ;-) ;-) 1 1
3349 Jawab kyun nahi aaya 1 1
3350 Sir plz come 4pm baj gae 1 1
3352 Bhai accident dubara toh ni kr dya,jo ab tk ni aaye,aa b jao sir 1 1
3353 bhai allah hafiz namaz ka waqt hone waala hai hum ja rahe hai.. par bhai aapse ek choti si reqwest hai aap agar chat karne aao to pls mujhe na bhul jaana... 1 1
3354 bhai busy hai? 1 1
3355 Salman bhai apne kuch bola tha shayad 1 1
3356 Tum jaan bhaijan ho ya gayab??? 1 1
3359 Salman bhai yr aap bhooot mast ho... ? 1 1
3360 aa jao.... rplyyy meee plzzzzzz 1 1
3361 ek reply bhai plzzzzz 1 1
3362 Kitna wait kervaoga 1 1
3364 Plz bhai aa jao 1 1
3367 kha ho yrrr. 4:36 bhi ho gye .. nxt year 4bje aaoge kya 1 1
3368 4 baj gaye lekin reply abhi baaki hai 1 1
3371 Bhai ...wait kar rahe h hum sab...time btaoo yaar 1 1
3375 bhaijaan thagaye hoghaey itne saare comments dekkar bhaijaan abhi online mein hai 1 1
3378 waah todi or request krke dekho tb bhi nhi dekhai dege salman bhai 1 1
3381 bhai jan balkum salam ma shimla sa ho to numsta salman bhai 1 1
3383 #Sallu_bhai mere ko prsnly msg kr do ek baar... Bhai #kuchh_khas hai aap ke lye, whatsapp 8083503086 1 1
3384 1hr se ziyada hogaya bhai ki khabr nai hai 1 1
3385 Hlo salman sir mai aur mere mammi papa apke bhut bde fan h 1 1
3386 pata nhe ap kb takh karata ha ya intizar 1 1
3387 Kha ho bhai , sb ke bhai 1 1
3389 Kabi nahe deka relpy wait mat karo 0 0
3391 Hii bhai kahan ho aap .. 1 1
3392 I think SALMAN SIR is taking a nap... jago salman bhai jago, aapka fans kab se wait kar raha hain....! 1 1
3393 wait kar raha hu 1 1
3398 next time bhai ke commitment ka wait karta hu. sayad aaj kuch jada bz hn juma h na. Love you bhaijan. khuda hafiz 1 1
3399 Han masjid main, ajana tum sab bhi.... 1 1
3403 plz javab degey na 1 1
3404 bye bye bye 1 1
3405 Khi BAJRANGI BHAIJAAN KIDNAPDED toh nhi gye! Ab toh12july ko hi pta chalega. 1 1
3406 Bhai jaan 4.30 pm ho gaiya hai ab to online avo kitna wait karvoga 1 1
3407 Ohhhh myy salluuu 2 2
3408 Bahi mai karanataka se hun apko dekne k lia bahut bhar aa chuka hun mumbai pe lekin uss time ku aap nahi mi le 11 sal se mumbai ara hun aj tak nahi deka plz reply bhai plz apse request hai 1 1
3413 Hi.kabhi kabhi apne fanse bhi mil lia kijie.kuch surprised to dena chahie... 1 1
3414 bhai we are waiting 5 bajne wala hai 1 1
3419 ek fake salman n cmnt kiya mujhe laga ap ho bhai 0 0
3420 hlw salman bhai 1 1
3421 Sub ka bhai salman bhai 2 2
3425 Salman Bhai app ko chada ni raha hu ksm as app ka naam hi Kafi hai 2 2
3429 hey salman bhai , agar aap actor na hote to kya hote? 1 1
3430 Tweet pe hi bta diye 1 1
3431 bhai sab ko rply krna bhai plZzzz sb ap ko pyr krte h sb ke sb 1 1
3432 yeeeee bhai aaaa gaye 1 1
3434 kya kyu sb ko pareshan kar rhe ho sb aap ke pichh le 1 gante se with kar rhe he 1 1
3445 Sub ko farig kro or mujh say baat krna plzzzzzzzz 1 1
3446 Sir uttrakhand se hai ! Yaha ke fans ko apka intezaar karna padta hai ! Plz come once to nanital ! 1 1
3447 Bahut log hai jo aapse baat karna chahte hai..plzzzzzz dn't brk thr hearts sir plzzzz 1 1

3448 Bhai woh kya matter hai Jo ye log bol rahe sb 1 1
3455 aap ki mujhey ek hebbit bohot acchi lagti h dusro ki madat karna ....like you 2 2
3460 hello chulbul ji..aye jaldi sbhi intazar kar kr rhe h apka 1 1
3461 Jhut bol diye. Itne jhutte ho to tumhare fan tum par trust kya karenge yrr. Ye Sonakshi sinha nahi hai jo such b olenge 0 0
3467 Bajrangbali ki vakt , kavi jhuut bolti nahi , toh aap kaise bola ? 1 1
3472 dekh lena bhaiyya ki film super hit hogi 2 2
3473 hell0 salman khan...kesy h ap..?? 1 1
3474 Bhai aa gaye hai jai jai bajrangbali 2 2
3476 Bhai 4 baj gaye hain 1 1
3477 Kahan ho aap bhai 1 1
3479 Chal jhuta....answer ek qustion ka b nhi diya 0 0
3480 bhai mujhe apna whats app no. do na roj krenge..... 1 1
3484 I knw apko muskil ho raha hoga sabko reply dene mai 0 0
3486 Sir bajrangi bhai jaan may Ali quli mirza aur Najeem khan hai 1 1
3488 mujey pahele aap ki movie bohot bekar lagti thi wanted se sab se acchi lagne lagi aap ki movie bro 1 1
3492 RAB ko yaad karu ek fariyaad karu salman khan sir se baat karade ya RABBA salam khan sir ka deedar kara d e ya RABBA. BHAI JAAN pls.wheting 1 1
3493 Bhaijaan muslim wali bat such hai ya fek hai plz reply sallu bhai 1 1
3494 Bhai itne comment kiye bus ek pe reply kar do plz plz plz 1 1
3497 main superman salman ka fan 1 1
3499 Please Maaki qasam javab doPlease Maaki qasam javab doPlease Maaki qasam javab doPlease Maaki qasam ja vab doPlease Maaki qasam javab doPlease Maaki qasam javab doPlease Maaki qasam javab doPlease Maaki qasam j avab doPlease Maaki qasam javab doPlease Maaki qasam javab doPlease Maaki qasam javab doPlease Maaki qasam javab do 1 1
3505 lekin im confused... fir aapne jhutha pist q kiya yaha pr :/ 0 0
3510 Kya hai yar ye!! 1 1
3516 lv u salman... schi m bat kroge kya sbse.. lv uuuuuuuuuuu sooooooooooo mch....... 2 2
3517 nr 1 salman 2 2
3518 bhai realy hurt jab nahi aana tha toh kyu kaha tha u know me ana study chodhkar 1 hour s msg kar rahi mera e xam hai kal phir bhi bye :(:(0 0
3521 AB to type karte kate fingariya dard hone lagi 0 0
3524 jab 1 lakh ppl love u love u likhenge how will u reply poor u 2 2
3553 bhai ap helpful prson ho sb ko pta h pr ap ki nazae me hlpful prsn kon h 2 2
3555 Bhai apki movie apka name pavan kumar ur mera real life m name h pawan kumar, toh m y movie dekhuga hi, motivate JO hona h, ek request h sir aap plz mere dp p like krdo plzz! Movie blockbuster hit toh pakki h! 2 2
3568 Ye umeed nahi this net khatam ho gya kya apka 0 0
3580 Salman Khan sir hame bajrangi bhaijaan me kya kuch new dekhne ko milega..? 2 2
3584 Hii..Salman Bhai mai abhi se kah raha hun ki apki Bajarangi Bhaijaan hit film hai... Ap kya Kahte hain???? 2 2
3590 sir mujhe koi q nai karna plz aap mujhe apna number dijia na bhai plz 1 1
3592 ab aa jaayiye mai aa gaya hu.....! ?? 1 1
3594 Wow 40,000 cmnts keep it up paaglo koi ni aane wala 0 0
3595 where r uu...bhaiii ...plzz one rplyy mustt onlyy onee:-(1 1
3599 bhai jaan me aap bhut badha fen ho sir me soch ta ho ki kabhi aap se miluga ya nahi sir jingi ki ek hi khuais th i ki ek baar aap se milu 2 2
3601 Eid ki aapko dher sari badhai ho, and BAJRANGI BHAIJAN 17ko screen par aai he to me or meri family sab dekhne jayenge, and me dua karta hu ki aapki ye film jyada tar log dekhe or 200corore ke club se bhi aage jaye. BES T OF LUCK. BEING HUMAN 1 1
3604 Sala kutta kamena amara time west kara tia salman 2 2
3605 mja aa gya bhai chat krk 1 1
3608 salman bhai asslam alykum 1 1
3610 <b> BHAAI BACCHO KA DIL DUKHARE HO AAP AATE Q NAHI 2 2
3611 Bhaijaan abhi tho 5 baje h Aap jab free ho tab aa jana m tho puri rat Intzar Kar sakta hu... iam big fan 2 2
3612 Kya bakvas hai yar 2 2
3613 avitak 4pm hua nehi kya ? 2 2

3615 watch band pad gai kya bhai aapki ....mere pass b thi apki jaisi ek bekar watch..maine OLX pe bech di 2 2
3616 salman bahi please hum ko bhi chance dena bt krne ka thanks. 2 2
3617 Reply plz bhai 2 2
3618 Rammi hindo salman. Kafir kuty ho tum gandooo 0 0
3619 Salman bhai ajao 200 comnt s kardie mene 2 2
3620 live chat karna kaise hai??? 2 2
3622 Sallluuuu i love u....plzzzz ek bar apne apse milne do...plzzzzzz 2 2
3624 Dongiri tb hogi jb salu bhai(Salman) nd GuRu bhai(Hemant) dono sath me honge.. 2 2
3627 bhai ap mjhe chnce nhi dena bt humre liye liye rply kr do bhai 1 1
3628 Bhai waiting eagerly for you...Plsss jaldi aao na, 3.30pm se 4pm aur avi 5pm ho gaya hai.Phn haanth mei lekar, aapkia page mei aapka wait kar rhe hai. 2 2
3629 Fake fake fake fake 0 0
3630 Ab 4 40 ho gaye hain..... 2 2
3631 bhai apna time barabaad mat karo kisi ko koi rply nai milne wala 0 0
3632 Salman plz reply 2 2
3633 itne sare logo me se humse kaise baat karoge?? :-(2 2
3634 Bhai Asr ki namaz baad aayenge kya ??? 2 2
3635 baat online kr rhe the bhai...abhi 5 bje khtm hua h 2 2
3636 Salman sir or pata chala hai ki aap aajkal jaada gym me time bitate hai..? is baat me kitni sachhi hai? or agar sa ch hai to kya aap kis new movie ke leye body bana rahe hai 2 2
3637 Fake fake ....fake ye fake h. Suna sabny 2 2
3639 Kafir wala name hi mila tha ap ko 2 2
3640 Salman plz plz fulfill my 1 wish 2 2
3641 plss salmAn rply kr do ....<3 2 2
3644 Na kro yr cmnts plz frnds 2 2
3645 Aisay ha bhaiya bajrangi...... 1 1
3647 lagta hai aap bahut busy ho par please ek bar bata do dete sir 1 1
3648 bahi,,kesehe ap?? 2 2
3649 tiger bahoot busy hai abhi... dont disterb cat 2 2
3651 Jodhpur aaoge jab milo ga...mai ab..kaha jaoge mujse bach ke..saadi gali tu ana hi padega 2 2
3653 Sir aapne reply nahi kiya !! 0 0
3655 Reply bhai jaldi aao 2 2
3658 Film se fursat nahi roza ka khak rakhe ge salman bhai 0 0
3659 Kab bat karenge salman ji app we r waiting ..pls rply.... 2 2
3662 Bhai jan jab awogay mujhe reply karna plzzz am waiting 2 2
3663 Apke liye possible nai hai sbko persanaly reply krna to apne ek bar me sbko thanx bol diya..acha hai.. 2 2
3664 Rinky Kumari salman hai vo alva nahi hai 2 2
3665 kamaal karte ho Q intzar kra rhe ho 2 2
3667 Tu Kitna bhi krle Salman teri bajrangi bhaijaan flop ho ker rahegi insaallah 2 2
3669 Kis kis ko rply denge salman...uffffff....jisko salman bahut achha lagta hain usko dena.... 2 2
3670 Salman bhaaaiiiii ilovee uuu alooottt... 2 2
3672 bhai kch hi cmnt me 50000 ko paar kr jyga ab tu hello hye bye bol do zzzzzzzzz 1 1
3673 kasm khuda ki itna toh class12 ke result ke din v exitmnt ny tha. 2 2
3674 salman bhai kaha ho aap replay kuy nahi dayrahay ho 2 2
3675 <b>BHAAI LAGTA H BAHUT BUSY HO KOI NAHI HOTA BUT ITNA 1 1
3676 Salman sir or kya aap baat nhi karege yaa time nhi hai aapke pass 0 0
3680 5 hogaye pr ap ne baat nh ki angry with you kamal kartay ho yar 0 0
3682 Bhai plzz ao na bajrangi bhaijaan bajrangi bhaijaan bajrangi bhaijaan bajrangi bhaijaan bajra ngi bhaijaan bajrangi Jai bajrang bali Tod de dushman ki nali 2 2
3686 Ha jao jao kon rok rha h tumen 2 2
3687 Kise.. aapna number de k jao sallu bhai... 2 2
3688 kamaal karte ho Salman ji aayeh bhi nahi.. 2 2
3689 wtng for ur rply ....plz 2 2
3696 hy bhai,life ek wish hai bas ek bar tumhe gaale milooo...... 2 2
3698 bhai ek request h jab movie k mid m intrrval ho tb kisi film k promo k bajay aapke dwara kiye gye #SwachhBh

arat k video ko zaroor dikhna. jis se indians bhi swachh bharat k prati zagrook ho jaye. bhai plz reply once 2 2
3699 arbaaz bhi thik kehte the aap less puntual hai 2 2
3700 Bhai hisar,haryana m aajao 2 2
3701 ek reply mujhe bi kardo bhai 2 2
3702 Paya Thakur nahi ata vo tum ko kuch kam hai kya to kar ke ajo 2 2
3704 kaha pe karinge baigan ;) 2 2
3705 sir ticket k paise ni hai 2 ticket bhejo 2 2
3709 ye l0... server d0wn 0 0
3710 Reply krdo bhaaaaaaaaaaaaaaaai 2 2
3712 3.30 baje kaha or ab 5.30 bajane wale h abi tak aap nahi aaye kitna wait kare salman .i really love u salman 2 2
3713 <b>BHAAI COMENTSS KRTE KARTE BETTRY LOW HONE KO AAYI YE KYA BAAT YR 2 2
3717 Hiiii me aap ki sab se bati Fan huuuuoo..... ???????? 2 2
3718 aap tho bat nahi kar rahi ho. wadda kiya bat korogi aap 2 2
3719 aao salman mujh se milne 2 2
3720 Koi dhkta bhi hoga etne comment. 2 2
3722 Sir aap indore aoge kya promotion ke liye. 2 2
3723 Dekhna friends Salman bhai zaror reply kary ga late hi sahi 2 2
3724 4:pm din b to bataae nah bahi ? bewakooof banaadiyaaa bahi apne to 1 1
3725 Apki ek mve ko itni bheed me ladke tck lekar or dosto ki bheed leke 3 3 time mve dekne jata hu or ap ho ki... 2 2
3727 Is baar ni hua katti bt nxt time jarur ho jauga...love u bhaijaan.......mmmmuuuuuuaaaaahhhhhhhh 2 2
3728 Aap mre fvrt ho pr aap rpl to kro 2 2
3729 bahut wt kiya aur nahi....bye...gussa hu app sa 1 1
3730 Hiii sir , aapse milne kaa sirf ek maaukaa chaahtaa hu. 1 1
3732 Bhai shaadi b kroge ya randwe hi mroge 0 0
3733 Fek I'd hai 0 0
3735 bhaiyya maine bahut intzar kiya lagta hai aap jayada busy hai koi bat nai kal jarur reply karna kal school ki chh uti b hai pls bhai 2 2
3736 oh bhai mere luck kitnakharap twtr nahe ho raha 2 2
3737 Bhay ab mera net pack khatam hone aya he 2 2
3739 tum achi acting krta h 2 2
3742 Hihihi sab ko ullu bana raha he salman 1 1
3743 live india pat baat kar rahe hai vo 2 2
3744 Bhai k sath apun kab se baat karnay ka soch hi raha tha..Whats App pr aah jana.. 2 2
3745 meri baat to ho gae..... A biggggg thanks sallu :-* 2 2
3746 Huhhh Watse our time ..... 3:30 se 4:00 Or ab tk ata pta nhi 0 0
3747 bhai jaan ek baar me aap se milna chahta hu bhai jaan please please 1 1
3748 kya bat karu ?? Bangladesh main jo Hindu k satha jo horaha hain... baat kar k kya hoga ?? 1 1
3749 Sir baat kr lo plzzzzzzzzzzzzzzzzzzzzzzzzzzzzzzz 1 1
3751 sir ye too National tragedy hoo jayegii agar ajj apne fan ko reply nahi diyaa ;) 0 0
3752 Pehle main milna chahti hu 1 1
3753 kitna paisa kama lega bajrangi 2 2
3754 Direct baat salman khan sir ke sath wooooooooo 2 2
3755 koi ta msg krdo Salman Khan 2 2
3756 Aate aaz bhai 2 2
3757 hlo salman sir 2 2
3761 Pagalo aa gae bus chalo ghus jao bus me jiyada aur rahy yaha to phir apka koi ilaj nhi 0 0
3762 aisi ummeed nhi thi aapse bhaijaan. . DIL tod diya sbka :-(2 2
3763 Aby yr kdr gum o gy ho rply tou kr do 2 2
3765 kyoki ittana pyr tumko krte he hum 2 2
3766 Sab log sun lo salman bhai rat ke 10 bje aayenge 2 2
3767 Kia baat kary gy 1 1
3768 Salman bhai may apke sath India ka garib or khana ,kapra na Milne walo ko apniii puri samata sey help karna chah ta hu.....plzzz help and thora support me plzzz 2 2
3769 veerji mainu aapse baat kar ke hi chain aayega 2 2

3772 Kya baat kya baat kya baat.....!!!! 1 1
3773 Yaar frnds aap sabko such mein lagta hai ye salman bhai ki id hai 1 1
3774 salman sir so rhe h....don't disturb 2 2
3775 jawab deyde...?? :(:(#livechatwithsalmankhan 2 2
3776 Pata ni kabhi meri b kismat mehrbaan hogi or me b bhai jaan se mil paunga..Ae khuda. Raham..:-) <3 2 2
3777 hello sallu bhaijan... 1 1
3779 Salman bhai rat ke 10 bje aayenge. Mob no of salman khan is 9999999999 real h. 1 1
3780 Agar aaj Maine pyar kiya relies hoti to kitna hit hoti? 1 1
3781 wo bahut busy hai kisi kaam me hoge is liye nahi aaye socha hoga tab tak kam pura ho jayega but nahi ho paya hoga kaam pura to nahi aa paye 2 2
3782 loveeeeeeeeeeeeeeeeeeeeeeee uuuuuuuuuu salmaannnnnnnnmnmm sir 2 2
3783 sir ek spna h zindgi m ek bar milna h aapse 2 2
3784 Itna intezar to roza khulna ka bhi ni krta jitna apka ek post ka karta hooo ....plzz reply karain maharbani apki b htt bhtt 2 2
3788 Asslawalecom salman bhai.. 2 2
3789 hlo sir kuch to kho 2 2
3790 Salman bhai kaha ho ap 1 1
3791 Salman bhai apke 50000 comments pure hone bale hai. 2 2
3792 hamay tu muqa bh nahi mila 2 2
3793 OK bye bye lagta hai it's just fun . 1 1
3794 Bhaijaan baat se palat gaye...... yeh aapki fitrat mai nai ho sakta.... aapka P.A naam kharaab kar rha hai aapka 2 2
3795 sallu yar 6 baj gaye.. 2 2
3797 Apne muje reply kiya to 'aj ki party meri taraf se' 1 1
3798 salaam sir kaise ho aap kahan ho plz aa b joa 1 1
3799 Aayesha Khan yaha to bandh karo ye dhandha 0 0
3803 Milna to ek bar yaad rkhna bar bar 2 2
3805 Logo Uallu bna Aata hai bus 4 se 6 bje gye hai kha ho Tum.... 0 0
3806 Salman bhai kahan hooo ...... 6 baj gaye ab 1 ghanta gaya to iftar ka time hota please ek reply dedijiye,, please bhaijaan 1 1
3807 plz rply kro naAAA:< 2 2
3808 Fake coment hai koi nahi ane wala 4baje kisi ko reply nahi mila jhot. 1 1
3809 Aajao na bhai kahe sarma rhe ho 2 2
3812 bhai kaha ho ap hum mar jyge 2 2
3813 Tum log bekar me apna time karab kar rahe ho koi nahi Aane wala 2 2
3814 Kyaaa sach me ye salman khan ki id he.........!!!!!!shayad fake he ye 2 2
3815 Aap shadi kab karoge yaar :(2 2
3816 aapko jamane se jhin lu ittana meri aukat nhi 2 2
3817 am d biggest fan of uaz... n d WORLD........... <3 <3 <3 <3 <3 <3 <3 <3 <3 <3 <3 <3 <3 <3 <3 <3 2 2
3818 Allah pak apko hadyat de bhai jan .. ameen 1 1
3821 Loveeee youuuuuu salman bhai.apki shadi ho jaye jaldi.aur bhagwan apko sweet sa baby gift kree. 2 2
3822 aap ne roja rakha hai ?? 2 2
3824 FAN ka teaser kaisa laga salman bhai 1 1
3825 Ekdum Zakaaas hai sab.. 2 2
3826 Ohh aaj mara bhai roja #tc 2 2
3828 salman khan ka aphran ho gya tv pe aa rha h 2 2
3829 bhai kb dhamakedaar entry marogeee.......i m waiting 4 uuuuuuu 2 2
3831 Bhai sallu tumhari pic hogi bindassIs baat pe hai mujhe pura vishvaas..Bas mujhse baat kr loo 2 2
3832 good evening Mr. khan ... mai apki bout bout bdi fan hu..i love u so much bhaijaan..... bhagwan apko hr buri na jar se bchaye or aap hmesha healthy rhe... love u bhai..... 2 2
3834 Breaking news dhayan se dekhiye ye insan ko jisne logo ko intzar krne m itna majbur kr diya 1 1
3835 Bhai jaan number btao 2 2
3836 commitmnt kr k bhul gye kya?? 2 2
3838 salman khan ne kl ka bola tha fr se check kro 2 2
3839 meko lag rha hai ye salman kbhi reply nhi degi 2 2

3840 bhai jaan aaye kya 2 2
3841 Salman bhai mje aap se milna hai...i am ur big big big fan... 2 2
3842 Auno Bhojani teri amma ki chodu replay kaiku kara sale 2 2
3843 still wait 4 bje kha ab time 6 hone wla 2 2
3845 kia hova bhai dya hova time katam ho gya 1 1
3846 sir 2 gnte ho gaye ans nae aya 1 1
3848 sir itna to koi apni girlfriend ka b wait nae krta pta nae ap ans kyn nae d rhe 1 1
3849 Bhaijaan main pakistan se ho main apka bohot bra fan ho 1 1
3850 hi salman sir how are sir main apki bohot bohot bohot bari fan hu i love you salman sir ummmmmmmmmmm mmmmmmmmmmmmmmh 1 1
3851 Bhaijaan kuch to bolo 2 2
3853 Salman sir aap fir kb online rahoge plz bta do na... Wrna mai puri raat jgi rhungi or apka wait krungi. Plz bhaie ya 1 reply..... 1 1
3854 Salman bolo tumne mushalmano ko yeh kaha tha ke nai kyun ki me aapko bohut like karti thi agar aap kahong e ke tumne esa kuch nai bola toh hi main manungi please reply jarur karna kasam he aapko 1 1
3855 Ye movie nahi chale bhai 1 1
3856 Bhaaiii orrr kitnaaa wait krwaogyy yrrrr.... 2 2
3857 Sir ? Aap kabhi promotion ke liye odisha ayiye plz 2 2
3858 ab iftari bad hi karna agar chat karna hai to... 2 2
3862 veerji i miss u apne ne baat nai ki jane dijiye koi gal ni 2 2
3863 Sach me aap hi baat kr rhe ho ya phr hum aise hi khus ho rhe hai..??? 2 2
3864 Woooooo WO kasy? Muje ap sy bt krne hy.kasy plz btyan. 2 2
3865 bai g kab aooo ge online 2 2
3866 Kya ye wo hi salman h jisne hum ko chelange kya h 2 2
3870 Sir it not fair RAMJAN KA MAHINA HA PICHA RAH GAE HM sayad mukadar me nh tha ! 0 0
3871 Agar Salman khan musalman h to allah se toba kare or apne gunaho ki mafi mange nhi to dunya me bhi usko k oi jagah nhi milegi or aankhirat me bhi 0 0
3872 salman ko duniya me jagah na dene ki kiski himmat hai salman k fans salman k sath hai 1 1
3874 Jo salman se pyar karte h wo islaam se pyar nhi karte 2 2
3875 Bhai je ram ram. Aur kaha ho 4 baj chuke h 2 2
3878 bhai aaye ki nahi ab tk 0 0
3879 sallu bhai kya baat h 1 1
3880 sirrr aap onlin he sirrr 1 1
3882 ullu bna rhe hai bajrangi bhai 2 2
3884 Axxa laga aapse baat kar k 2 2
3885 Really aap aaye kya.....i can't beleive.... 1 1
3886 hum bajarng bali k bhakt h or hum kbi juth nhi bolte .....kyo bhai ... 1 1
3887 Agr replay nhi diya to me maggy khalunga 0 0
3889 bhai ka uper cmnt aya he 2 2
3890 Aap koun saa phone use karte ho that is a useless phone......mera message aapko display nhi kar paa rha hoga... warna aap to reply karte hi karte.... 2 2
3892 salman sir aap aiye b nai baat karne :(2 2
3893 yr koi bata do salman aaye the ki nahi koi bol raha chat ho gaya but kahan? 1 1
3894 Kuch toh baat hai bande mein... jo itne logon k dil me rehta hai.... 1 1
3895 aab bas karoooo salman ji 2 2
3897 agar sach me mujhe app ke sat bat ho jata to me khusi se pagal ho jata... 2 2
3899 kya Sallu dost ko bhul gya kya 2 2
3900 bhaijaan ed mubark. 1 1
3901 Hame bula kar khud kahan chale gaye 2 2
3902 salman bhai muje ek gaddi gift krde yr ? 1 1
3903 Hum aapko ek jhalk dekhne ke liy a tarap rahe hai 2 2
3904 Hum fans ki feel ki koi kadar hi nahi h 1 1
3905 kamal krte ho panday ji sb fan ko itna wait kra rhe ho 2 2
3906 Love u salman kbse wati kar rahe h. 2 2
3907 Salman bhiya mujhe aap ki dabang three me ek chota sa roll de dena 2 2

3908 Bhai aaap ki Sab say aache film kon si hai . 2 2
3909 Salman dear bye aftari kerte ha hum prr melinge... 2 2
3910 Last replyyyyy plzz plzzz plzzzz 2 2
3912 Kutte ki nhi sunni h koi bat 0 0
3913 Hlw,bajrangi bhaijaan 1 1
3914 bajrangi bhai jan ke liye dil se subhkamnaye sir 2 2
3915 ek bar msg karo bhaijaan 1 1
3916 ye kya hai 3 min ka bhi video nahi hai aur shirf 2 sawal asli akda bhejti hun abhi Twitter par 1 1
3919 Sb kh rhe h fake id fake id . Aap ko proove krna chahiye aap hi ho. 2 2
3920 Bhaijaan plz tell plz bhai one comment 2 2
3922 hi bhai main bhi ek actor banna chahta hoon bhi 1 1
3924 Realy kaha par 1 1
3926 Salman bhai aap meri coments path te he 2 2
3927 bhai i m aslo big fan of you .. i m frm kashmirr   ..apka bajrangi bhaijaan main kaise experince Raha ..woh bh
i kashmir main shooting kr k nd pls replyy aur kaise laga humra kashmir 2 2
3928 kya yr maine b mis kr diya #uhh 2 2
3929 intzaar mat karao aa b jao ya fir bol do ki kal baat karenge 2 2
3930 Kya khismat hai yaar bande ..ki hazaron .. croro .. log ... marte hai bande par 2 2
3931 NAMASTE SALMAN BHAI :) ?I AM BIG FAN YOU SUPERSTAR BAJRANGI BAJHAAN VERY VER
Y BEATUFIL END SALMAN KHNAN VERY VERY VERYVERY VERYY HANDSOME SALMAN KHAN SU
PERSTAR (??GOLDEN HEART MAN ??) MAI TUMSE PYAAR KARTE HUN :) ???? 2 2
3932 Radhika Medan- sirf salman ko chod kr baki sab call krnge tmhe.. ;) :) 2 2
3933 Bhai jaan majhe le rahe ho malum h 2 2
3934 Kaise ho bhai jaan mai apni gf k saath bajragi bhaijaan ka first show dekhne ja raha hu.... 1 1
3935 lol admin atention seeker h jante bujhte post dala ki coment mile >_< 0 0
3936 Hamare des ki saan hamare dil ki arman ham sab ki jaan hi hamare salman bhai jaan 2 2
3937 salman bhaijan,....se baat krne hai,...bass ye bolna hai ur moive will superhit..... 2 2
3939 mohammed ji kya salman khan ko sachmooch kisine kidnap kiya h pls bataiye na.... 2 2
3941 Muslim hokar muslim ko hi neech bolte ho. .. Allah se daro. ... ye saari dolat sab kuch yaho reh jayega. .. Khu
da se daro 2 2
3943 salman ne reply kiya kya guys 1 1
3944 Salman se baat karne ke liye taqdirwala honi chahiye 1 1
3945 Maahi baghel aram se 2 2
3946 Salman ji aap vul gy kya aap ka ek beta hai?? 2 2
3947 Salman sir kahan.....h aap .aap to humse baat karne wale thai na 2 2
3948 Bhai acha mazzak banaya aap ne aaj :-W 2 2
3949 Hum fans ki koi kadar hi nahi h 2 2
3950 salman sir aap kyo nai aiye online 2 2
3951 Insha Allah blockbuster hoge bhai abki bar 300 cross Paar bajrangi bhaijaan 2 2
3953 Kya huwa bhai aap ne apni baat nahi puri ki kuch kaam mei phas gaye ya Hume bhool gaye 2 2
3954 Hi salman khan ! Plz kisi ko tou reply kare na....... 2 2
3958 mere sawal ka jawab koi nhi de sakta ;) 2 2
3960 Bhai plz thoda time humare liye bhi nikal lijiye hume bhi aapse baat karni h 1 1
3961 Oh jalwa jaha bhi hoga baha bhi hoga kya aapka hi jalwa jalwa jalwa 2 2
3962 Hyd mai sabse ziyada apke fans hai salman hum to bajrangi bhaijaan zarur dekhngeeee 2 2
3963 Madar Salman nai chalegi film 2 2
3964 Sallu bhai aaj apka dil garden garden kyu ho rha h....??? Koi to locha h.... 2 2
3965 Salman bhai loves us....so dnt be disappointed. ...ek din bhai se milne ka mauka sab ko milega... 2 2
3966 tum mujhse pyar karte ho han to q 2 2
3967 sakunga appse bs yahi dua karta hu aapse jald mil saku 2 2
3968 bhai ko zrur koi kam hoga 1 1
3969 ????????kaise piche pad gaye re...... 2 2
3970 plz ap prem patel ki help kijiye plzzzzz. 1 1
3971 salmam ji kya hai aap rply nahi dai rahai ho. thoda dyan muj ko b dona plzzz 2 2
3972 woow aap 1988 se fb per ho me to 2011 me fb per aai :) 2 2

3973 aap par ek ilzaam laga hai ki aapne muslim ko galat kaha hai kya ye sahi hai mera dil nahin manta hai ki aap ai sa bole hain 2 2
3974 kal 4 pm wait karna,aj ka din nikal gya,bevkufo,girl to puri puri bevkuf hoti hai, 2 2
3975 Sallu bhai reply do na us ladki ko plz ye mera izzat ka sawal hai plz..plz..plz.. 2 2
3977 salman sir abi tak wait kar reha hai hum plz abi b aa joa 2 2
3979 md.intjar.malik. bajrangi.bhijane. .i love.you. (786). bhijane.apki.film. no: 1 hogi. 2 2
3980 app kaha hoo please aa jao na 2 2
3981 Bina musalman ke hit ho jaye ga ye film samje 0 0
3982 md.intjar.malik. bajrangi.bhijane. .i love.you. (786). bhijane.apki.film. no: 1 hogi. bhijane.mujko call.karo. 886 8026194. 2 2
3983 sir aa jaao sabhi log aapke fans aapki wait kar rahe plz aa jaayie g plz....sir ek msg bda sa reply kar plz time nik alkar g 1 1
3984 Ye musalman jab HINDUO se dosti karni hoti hai ya meljol karna hota hai to shirt-pant aur bina topi ke aate ha in....Aur jab hinduo se tanaav hota hai to kurta pazama aur topi lagakar aate hain aapne bhi dekha hoga...Aisa kyo??? 0 0
3986 Bhai jaan kab aao gee 1 1
3987 Bhijan mera no 9933742434 1 1
3989 Salman Sir EK reply toh de de bhaijaan 1 1
3990 Abi tk chhar n bje k konsi ghadi lga rki h,, 0 0
3991 teri to saqal bhi dekhne ko dil nahi karta 0 0
3993 kha par bhai 1 1
3994 Salman ap ku nhi aey 1 1
3995 Hum thhak gaye bhai apka intazar kar k kar k hamari ungaliya b duk rahi hai typing karke 1 1
3996 Inteha ho gayi intezar ki,Aayi na kuch khabar salman bhai ki... 1 1
3997 Kiu baat karte hai? Kis chiz mai? 1 1
3998 bhai bhai bhai kaha ho 1 1
3999 Khushboo Tripathi,,, I love you bol khusboo lga ke 2 2
4000 Salman sir ne bs time btaya hai date nhi jb date bta denge to aa jaeyega aap sb comment krne okay.CLOSE IT 1 1
4001 dekhate abhi tum hamare liye time dete ho kya to 0 0
4002 bhai jaan aap raipur aaoge kya 1 1
4004 bhai ne to popat kar diya 0 0
4005 jhuti hi umid shi pr batta do sir fir kub milege 0 0
4007 Bhai to he to koe gam nae 1 1
4008 Koch to bolu bhai 1 1
4011 sir humne 200 se bhi adik msg kiya pl 1 to kuch bol do aap 1 1
4012 Bhai jaan bolu naa.. yar 1 1
4013 sir 4 baje se lgi hue hu kuch to rpy kr do 1 1
4014 Aap khafi busy rhte ho uncle. 0 0
4015 Aslam tu agr bigest fan he to kia apun smilest fan he salman khan ke 1 1
4016 miss ho gya bhai 1 1
4017 Salman bhai aapne mera hi nai blki sab fans ka dil dukhaya hai 0 0
4018 Salman bhai plz muj se baat kre plz plz plz 1 1
4019 bhai plz 6000screen pe release karo bajrangi bhaijaan 1 1
4020 Plz sallu help him aap sabki dua hi lete hona plzz inki b 1 1
4021 salman aap shadi kyu nahi kar rahe 1 1
4022 plz salman g ap sb ki help krte ho is bichare ki b kr do na apni mom ki khatir 1 1
4023 Hiiiiiiii Salmannnnnnnn ji meri ma aap ki BIGEST fan hain main aap ke sath baat karna chati hoon 2 2
4024 bhai kaha gum ho gye 1 1
4025 1 reply tho de dete 1 1
4026 Neeta tum ek kaam karo salman se shadi karlo 1 1
4028 Assalam walaikum.Kaise ho salman khan.Ramzan Mubarak hu.... 1 1
4029 sir gud ni8 bol kr bhi lgi hue hu comment pr comment sir bhi dard hone lga 1 1
4030 To kya chayiye apko 1 1
4031 salman ap ko kabi b achhi intresting story wali films kyu nhe milee jis trha shahrukh ya amir ko milti ha 0 0

4032 App kise baat kar rahi ho 1 1
4033 sir kya msg krtte kartte mar jau 0 0
4034 Dishta Jeetoo salman nahi reply karte hein hamare dil ko broken karte hein 0 0
4035 Kisko bol rahi ho 1 1
4036 Hiiiiiii salman sir 1 1
4037 Aaj kal had koi salman khan banta pherta ha 0 0
4038 tere se kaun baat karna chahata hai be jo khud musalman ho kar musalmano ki izzat nahi karta 0 0
4039 Ab tu jada he bolne lagi hai..soch kar bat kar warna... 0 0
4040 Chal ba tharke 0 0
4041 bhai tumhare bare me jyada nahi sochte tum dil me aate ho 4pm fb nahi... 1 1
4042 Ya allah ap abhi tak nahi aye salman :(1 1
4043 Itne saare commt. Bhaijaan saare comnt padh rhe hai isliye time nhi mil rha hai aana ka 1 1
4044 Bajrangi bhaijaan 2 day 100 cror club me pohch jayegi 2 2
4045 Are kanjos ji aj to in sab se bat karo sab ko khush kar do itna to kar saktea ho ka nhe aj kanjossssss mat bana p lzzzzzzzzzzzzzzzzzzzzzzzzzzzzzz salman khan plzzzzzzzz 0 0
4046 gud ni8 bhai 1 1
4047 Koii fadaa nhii yarro .. Sabbbb fake hii 0 0
4048 Main to Bajrangi Bhaijaan dekhne bilkul nai jaunga friends aap bhi mat jana 0 0
4049 abe koi toh batao akhir chal kya rha yahan 0 0
4050 kayeh tiger khan bolu..? 1 1
4051 Nahi ainge salman sab so jao 0 0
4052 Nikita Islam Sunati bhai tum ye fake id se kyu pagal bana raha hai yaar in ladko ko 0 0
4053 salman sir sara din guzar gaya aap aa he nai :(0 0
4054 Sana khan add karo mujhe 1 1
4055 Sana aa gaya tumara nakli salman khan hahaha.... 0 0
4056 Hahah okay ap sab ke marzi ok bye 1 1
4057 Lage raho sallu bhai 1 1
4058 okk bhai bolo ku6.. 1 1
4060 Hellow salman bhijaan.... 1 1
4061 Bhai ap sapna kyu dekhate ho bhai bus apke fan h hum 1 1
4062 Tum kahe rahi ho oh fake hai agar real me aaye to tum kaise pahchano gi tum hahaha 0 0
4063 friends tum sab salman khan ku thaknah chati hu. ...??? 1 1
4064 Bhaijaan kidar ho 1 1
4065 salman rly deke chala bhi gya dosto piiche jaake dekho 1 1
4066 Bhai ko kisi ne kuch keh dia kia 1 1
4067 Idar hi hun bahi 1 1
4068 kaha hai salman 1 1
4069 sab asca ah.... :) 2 2
4070 lo aa gya mai...salman ka fan...swagat nhi kroge hamara? 1 1
4071 Kaise kahe rahi ho ki oh fake tha 0 0
4072 plz cm bhai...hm sb wait kr rahe h apka 1 1
4073 Sir main mumbai ja raha hun....aapke ghar ke pas jarur jaubga 1 1
4074 acha sab ye btao bhai ka sbse bda fan kon hai 1 1
4075 Roja nehi rakhkar film bana rahahay.... Haaramka khana kha raha hay 0 0
4076 Bhai hume lift hi ni dete.....hum to larki nii ha na yarr 0 0
4077 help meee salman khan 1 1
4078 Kis bewakuf ne apko yeh bola ki ....Apne fans ko iss tarh se wait karao2 thappad meri tarf se bajrangi bhaijaan padne chye usey jo aysi bekar muft me raye deta hay 0 0
4079 Sir ji kaha rah gye ab aa bhi jao jldi 1 1
4080 sorry year... sara then salman khan kiy sathe mere thenb barbat... :'(0 0
4081 Salman Khan apka dil sch me pure water jaisa hai 2 2
4082 Salmaaaaaaaaaaaaaaaaaaaaaaaaaaaaaaan ajhooo 4 ghanto se apka wait kar rahi hu 1 1
4083 Apki upcoming picture ke liye bhaut excited hun mai 2 2
4084 cheat kiya sallu ne 0 0
4085 oye fake id #salman_khan tera to public #acount hai ... real hai to #page se reply mar 0 0

4086 Kiya bhai ouvesi sb par Gussa kiyu hu 1 1
4087 agr aana nhi tha to bolna jroori tha :(0 0
4088 Mera beta aap ko bahot pasand krta hai 2 2
4089 Plz ply ply plz plz salman sir 1 reply 1 1
4090 Acha ab mulliya b fb chalane lagi hai 1 1
4091 Ya allah salman agar nahi ana tha to pehle bola kyu thaa 0 0
4092 Ye faltu logo ka to yehi kam h kisi ko acha nhi dekh sakte 0 0
4093 Hallo salman vaiya 1 1
4094 zindagi bhar intezar bhaiiiii.....is duniya mein sirf aap ke liye 1 1
4095 Salman tera garoo bhi too tega tujhe fakre hai aapne Filme pe dekhe lena eid ka din tum hara filme koye bhi m usalman nahi dekhe ga kiya budha to 50 ka hogaya... 0 0
4096 ASSALAMALIKUM.....ap ki aesi jo aadhri dowaye hai jo ramzan k sadk puri ho jaye.............RAMZAAN M OBARAK 1 1
4097 jisko lgta h bhai nahi aaye the ye link kholo or dekho kitne logo ko bhai ne jawab diye livestream.com/fblive/s almankhan 1 1
4098 Kb bjenge tumhare 4 salman ? ?? 1 1
4099 Jo tum bolte ho tum wo karte nahi ho 0 0
4100 Avi ku good night, maja abvi baki hai!!#sunati 1 1
4101 pandey ji kamal kardiya apne aap 4.00pm ko batkarne wale thay apk 4.00 bajege bhi ya nahi 1 1
4102 Love you salman bhai ...missing. .you ..salman bhai 2 2
4103 Hum cahtiha ki vai, ek bar ake sobko jalak lagadea 1 1
4104 Aapko hamari b umar lag jaye, itne bade fan hi ham 2 2
4105 aj apne hum sbko bht hurt kiye salman...apse ye umid nhi thi hum sb ko :-(...bt m also luv u salman...:) 1 1
4106 bhai jaan reply to do chootiya ho ki nhi 0 0
4107 Hum bhi aap se baat karna chahte h so plz plz one reply plz plz plz plz plz plz plz plz plz plz plz plz-1000;)) 1 1
4108 Ae fake salman Khan he Salman khan aur tiger khan ek adme he jo 2 2 accout use ker rha he sassery ne time w ast ker deya 0 0
4109 bhai aise karoge toh ham nhi jaynge dkhne bajrngi bhaijan 0 0
4110 aur salman bhai movie to chootiya se bani aapne 0 0
4111 Kucch b kaho...salman bhai kaa sabse nada fan iss puray shrishti mein afar koi hai...tyo o hai...mera bhaiSachin Pujari.....jab tak salman bhai zindagi mein ek baar agar inse naa mile...tyo zindagi mein mein kissise kya mile... 2 2
4112 Ek baar milke tyo dekiye...aap daang reh jaayenge... 1 1
4113 Aallah qasam salman khan agr bt ni ap ne hm se tou aaj k bd main khbi apki ek film b ni dekhunga aate q ni ho 0 0
4114 Nu cred ca ii fb lui salman khan 1 1
4115 plz rpy jst wnt to meet u once hope u cme kolkata once 1 1
4116 pehle ek baat batao doston salman ne twitter par ya fb par aane ko bola??? 1 1
4117 i think salman ne ye twitter k lie kaha hai qk salman ka ye post twitter se hi liya gya hai.....toh hmlog yahan ky a kar rhe 1 1
4118 Ye fake id h salman sir ki kyuki esa ho nhi skta ki salman sir ko sat shree akal likhna aata :-) 0 0
4119 salu bahi sabka bahi salman bahi,,My Supar kahn Salman kahn kick se v suparhit hogi bajrangi bahijaan, 2 2
4120 Salman khan bn kr yaha kyi logo ne fake msg kiya h.... Bt hm hmesha real hero my salman khan se hmesha py ar krenge 2 2
4121 salman ne ye twitter k lie kaha hai qk salman ka ye post twitter se hi liya gya hai.....toh hmlog yahan kya kar rh e 1 1
4122 salman sir for u dabe mein daba dabe mein cake mera piyare salman sir cororo mein aik 1 1
4123 Aap Kisi se bhi baat nahi karte ho 0 0
4124 ye page fake nai hai bas es mein fake loug salman khan ban kar aa jate hai per ye page million corore sahi hai s alman sir ki he id hai ye 1 1
4125 salman ab nhi bachoge jab tak reply nhi doge mai picha nhi chorne wali 0 0
4126 Salman bhai abhi tak wait kar rha hu apka 1 1
4127 sojao pooja salman nahi aanewala hai kyo rat kharab karrhi ho tum 0 0
4128 Pooja soja nahi ane wala bhaija 0 0
4129 are bhai salman yahan aane waala hai na twitter par koi bataiga qk iss post me twiitter ya fb mention nhi kiya h

ua hai 1 1
4130 hhaha mjak mt kro aap ka phone off aa rha hai salman bhiya 0 0
4131 Salman bhai bada wala tharra Desi ka pike so gaye Heheheh 0 0
4132 Salman bhai i knw ur a big celebrity par hakk toh hume bhi milna chaye apse baat krne ka ! 1 1
4133 Hiii Salman sir mujhe apse milna hai plzzzzz sir main aoki bahat badi fan hu....<3 1 1
4134 Sale kyu pagal ho rahe ho chootiyao salman nikal liya 0 0
4135 Baba ji aap to so jao q budhape me nind kharab kar rahe ho 1 1
4136 missss u salman 1 1
4137 bhaijaan ramzan kese chl rhe hh 1 1
4138 Bhai movie itni chalegi ki record ho jaayega 2 2
4139 Sir pls kaise aapse contact kare batana pls ???? 1 1
4140 Kya hua tera wada...hmne to suna tha ki aap ek bar commentment kr dete ho to khud ki b nhi sunte bolo sallu b hai..kya hua. Aaj ka wada bat krne ka 1 1
4141 qqq kiya aapne aisa 1 1
4142 Mera yeh matlab nahi thaa naam to acha hai Salmanji yaar shaadi karlo abb tooo...:D 1 1
4143 lovee uuu salluuuu 2 2
4144 bajrangi bhaijan jaldi aa jao cinema hall me 1 1
4145 lv u salman bhai 2 2
4146 bhai apku sath Katrina ko film mere bohut ppochondo 1 1
4147 Hello "BAJRANGI BHAIJAAN" 1 1
4148 Salman g kabi roza rakha hi? 1 1
4149 Ok salman bha!!!! 1 1
4150 ok boss apka hukum sir ankho pe 2 2
4151 as salam walekum salman g apke sab fan ko pata h k aal kavi v kisiko hrt nhi kar sakte ho allah se dua h k apko hr musibat se bachae or buri najar se v 2 2
4152 Jaha bhi jay hit pe hit ho jay only sallu bhaiiiiiiiii 2 2
4153 Salman khan me aapka wait kar rahi hu pliz 1 1
4154 Jo muslmano pe kicad uthayega hum use kuchl deege yaad rkh che koi v ho 0 0
4155 Salman bhai jai ho 2 2
4156 kya yar aap shaadi karlo na yar abhi to aapka jawani bhi khatam horahe hai 1 1
4157 shadi kab kar rahy ho ye news chahiye ab toh 1 1
4158 Chup chootiya jaakay shahrukh ka signature style perform kar shahrukh k tattay 0 0
4159 Asalamwalaikum sallu bhai aap hai logo ke baato ko dil par nakko liyo chahay kuch bi ho jaye mai aap ke sath hu ok ur the great bhai great handsof 2 u luv u sallu bhai 2 2
4160 Ye pakistani bchchi ko ap pakistan apno se milate hain dil khush hojata h dekh kar pori film dekhy gye to mza ajay ga sir 2 2
4161 Me bhi ik actor hi hon song and drama se ret.kio ki ghaziabad ho islia miss karonga dilhi hota to zaror dekhta t hanks 1 1
4162 Asalmwale coom bhaijaan 1 1
4163 bajrangi bhai jaan kaise ho 1 1
4165 Hlo sir kya aap kbhi mujhse baat krege mujhe aapse kuch share krna hai 1 1
4166 Asalam u alekom salman bhai. 1 1
4167 niceeeeeeee loveeeeee you bhaijaaan 2 2
4168 bhai aap tou best he bajrangi bhaijaan ATBB hogi ;) 2 2
4169 Salman bhai mujhe aap se jaruri bat karni hai 1 1
4170 Haa hme bharosha h bhai 1 1
4171 sir ek war baat to krlo please sir 1 1
4172 bhaijaan aap awesome hi 2 2
4173 India ki aan baan shaanone only one salman khan 2 2
4174 Muje apse shadi karni hai 1 1
4175 Shadi karoge mujhse salman g 2 2
4176 apkeliye ek accha movie hai mere pass 1 1
4177 Mujhe aap ki movie or bigg boss ka bhttt besabri se intezr hota hai 1 1
4178 welcome bajarangi bijaan 1 1
4179 alwys wth u 2 2

4180 we know bhaijaaan kwo sab fake tha 0 0
4181 I lv #saLmAN <3<3 2 2
4182 Salman bhai ma Pakistan sa hun main aapka bht bara fan hun or ma aapko milna chta hun mari ya khoish hai 1
1
4183 Yess... bhaaijaan... hum aapke sath hai hamesha.. kuch b ho... love u sir g.. 2 2
4184 sir ap roja rakte ho 1 1
4185 Bhai film hit hoga haters ko mo thod javab denge 2 2
4186 Bhaijan apke sath dekhni hai filmBajrangi bhaijann 1 1
4187 ye tumse jalne walo k kaam h bhai 0 0
4188 Salman mera bacha tere naam se wo blue mark kider gyaaaaa 1 1
4189 <3 iloveee yooou 2 2
4190 Tension leneka nehi #BHAIJAAN. 2 2
4191 Lakh zuban lakh baate 1 1
4192 love uu salluuu 2 2
4193 3 bar dkhunga m theatre m jake bajrangi bhaijaan 2 2
4194 Bummprrr hittt in saahALLAH.... 2 2
4195 I m frm Pakistan apki bht bht bht badi fan houn. 1 1
4196 Bhai ram ram 1 1
4197 Sir Ji kabhi roze namaz ki bat b share kr liya kro 1 1
4198 bhai voh owaisi ke chamche hi is fake news ko failarahe hai 0 0
4199 Main ne tu kbi in romurs py yakeen kiya hi ni tha Bhai jaan 0 0
4200 kl aapne waisa qq kiya 1 1
4202 Tension lene ka nhi bhai aapke fan ki koi kami nhi voh aapki film zaroor dekhenge 2 2
4203 acha kia yeh post update karke kyuki mai bhi yakeen karne laga tha is msgs ka.... ab sukun hua k tumne aisa na
hi bola salman :)) 1 1
4204 bhai media me aake bayan do .....sale kon hai rumor urane wala..... 0 0
4205 GUD NYT BHAI 1 1
4206 bhai mko.farq ni pdta 0 0
4208 salman khan u r my god father...agr aap na hotey aaj m kuch na hota.. 2 2
4209 Hum log Jarur dekhenge aapka movie.. 2 2
4210 pahle sahi se islam ka meaning sikh lo tum aur apne pita ji ko bhi sikhao 0 0
4211 HUM HAMESHA AAPKE SATH H BHAI AAPKE LIYE JAN HAJIR H 1 1
4212 Bas dil pak ho bhale duinya kuch b kahe 1 1
4213 No problems Salman Bhai aapka koi bhi kuch ukhad nahi sakta bajrangi Bhai jaan suparhit movie hogi 2 2
4214 Sir sab ko pata hai na aap kise ka bura krta Ho or na kise ka bura chahta ho loveyou 2 2
4215 Lagta hai darr gaya#UTurn 0 0
4216 Love love love salam bhai 2 2
4217 Salu bhai. Aap ka move jarur hit hogi bajrangi bali he na. ! Jis ke, saath bhagban hote hain hun ka koi kuch neh
i kar sakta.! 2 2
4218 sahi baat h bhai.. qki sbka malik ek 2 2
4219 hme to phle hi pta tha salman aapke bare me sb jhut kha h... pr kuch akl k andhon ko kon smjhaye .... i love u
always salman ....i recpact u allways ......:-*:-*:-*:-*:-*:-* 2 2
4220 koi bhi uss fake pi pr yakeen kaise kr sakta h itna hi doubt tha toh trailr launch wala video dkh le na chahiye th
a sb clear ho jata 0 0
4221 Bhai plz dont drink alkhol plz plz plz plz 0 0
4222 Ese he baiya bajrangi., 1 1
4223 Bhai ki jai ho 1 1
4224 yakeen krne se phle ek baar video dekh lena chahiye tha 1 1
4225 i jush hope k wo insaan jald se jald pkra jaye jisne yeh fake pic bnaya 0 0
4226 Bhai aapki film super hit hogi hamari duaa aapke sath hai aur aapke sirpe mere bajrang balika hath hai Allah se
duaa kartahu ki aapki film super ho 2 2
4227 Mayne kick Ki baat ik bi film nahi daka serif aap Ki film Ka intazar ha 1 1
4228 V nice commet salu bhai 2 2
4229 Aap ne aisa kaha ho ya na kaha ho i dont care but me Aap ka har ek movie dekhta hu dekhu ga aur hamesha de
kh ta hi rahu ga. Love u bhai jaaannn.. 1 1

4230 GUD NIHT DOST ALAH AFIZ SALMAN KHAN MERY GAN TUM 1 1
4231 keep calm Salman bhaii....we r alwyzz wid uh...Salmania rockd...haters shockd....love uhhh bhai:-) 2 2
4232 We trust are bhaijan baki bhot jan may fal gaya hai bhai dua kara gaya in shah allah hoja sab tik bhai 1 1
4233 Salman apko mujh pa trust ni ha ya ap khuc smjhty he ni mujhe 1 1
4234 bhaijaan hume aap par pura barosa he....aap aisi baat kavi kaar hi nhi skte....lov u bhaijaan????????? 2 2
4235 U r absolutely right bhai aap aisa kbhi bayan hi nai desakte muslim k khilaaf yeh sab apko badnam karne ki aur apki image ko kharab karne planning. Hope inshallaha police jald se jald inn dhokebaaz logon ko giraftaar karle. 0 0
4236 bhaya ye bhajrangi bhai Jan ko flop krnay k leye ye kaam kya ja raha hy 0 0
4237 Bhai me samjha samjha ke thak gya tha logo ko 1 1
4238 I love uuuuuii salman khaaan ;-);-);-);-) 2 2
4239 I blv u... Kp shining ma luv.. TZ luvs u.. :) :* 2 2
4240 I know bhai Muslim kabhi kisi ke religion ke baare me galat nahi bolta. 1 1
4241 Vai always we love u, or hm sabko paataha aap aisa kuch nahi karenge jo kisiko dukh pouche.... always vaijaa n r8 or jo kartaha kisiko na kisiko vala hotaha..... 1 1
4242 Salman bhai bharosa hai aap par.. 1 1
4243 kitny farigh log ho yar kyn requests bhej ry ho 1 1
4244 Don't worry bhai.. kehne wale kehte rahein just ignore... #Being_human_bajrangi_bhaijaan_6_days_to_go 1 1
4245 Ek the tiger bhi super hit hua Yarr sallu Bhai lekin there naam ka kia look ta app ka sallu Bhai ekdam radhe 1 1
4246 Salman bhai tension mat lo....hum is ovaisi ki baaton me nhi aane wale.....movie super duper hit he 2 2
4247 Choro sir...ye country kbhi feelings ke respect krna ni seekh sakta... 0 0
4248 #salman khan is rocking star 2 2
4249 Kuch log zindagi bar nhi samjte or na dusro ko samjhne dete h kud toh bekar hote h dusro ko bhi apni tarh bana dete isliye vo kabhi nhi samjege. 0 0
4250 Hmare bina tera koi wajood nhi #salman ...sale pathano k nam p kalank h tu 0 0
4251 Salman Bhai esi post ka intezaar tha thank u bhai shukriya Maherbani. 2 2
4252 J'ai ho kay tym nahi Kaha tha appna 1 1
4253 Salman bhai mujhe to pata hai ke ye sub news jo bhi hai wo ghalat hai app ki film dekhne ke baad sub ko malo om hota ke koun kya hai aur koun kya hai 1 1
4254 We dnt blv sch nonsense keep da gud wrk!! 1 1
4255 Aapne ye sbko btayaki hm insan haitb hindu ur musalmanNd india ur Pakistan. ...Bt pta nhi kyu log smjhte nhi Bt koi nhi One day they wil realize. 1 1
4256 Tension nehi leneka vai,kuch bhi ho jaye,kuch bhi loog kahe hum hamesha aapke saath hay,ek aan,ek shaan ek hamara bhaai Jaan :*just waiting for Bajrangi Bhaaijaan :)Love and Respect From Bangladesh............. 2 2
4257 pehle mai musalman hun bad mai bhai ka frnz hunn frenz hai bhajrangi movie flop hogi dekh lena 0 0
4258 Yr tum sab ko bahut takleef he bhai ki sadi. ..se. ..heiiiiinn? 0 0
4259 Sojaa yrr boor mtkr Alia Khan 1 1
4260 Mere yeha ek ladka bhi nhi jayega movie dekhne 0 0
4261 aBHI SE SALMAN BHAI TENSION MAI HAHAHAHAHAHAHA 0 0
4262 salman kya hai aam insan hai hamari tarha jo help manga hai allah se mango 1 1
4263 kuch karna se pehla soch kar karna 1 1
4264 Salman bhai kiyu aap sab se banga le rahe ho aap aap ke kam me jan do pilz 1 1
4265 Hey koi meri sister ko pareshan mat karo 0 0
4266 Ap jesi jaha miljati he fir durghatna ghatjati he 0 0
4267 OK Alia Bora mtmano sujao khoda Hafiz by Mafi mangne ki adat nhi samajjao byyyyyy.... 1 1
4268 Aur raha bt salman khan ka to salman khan ko apna name chenge kar dena chahiye salman nhi rakh k unko sal man kumar rakhnaa chahiye isliye k o muslma k name per dhabaaa haiiiiiiiiiii 0 0
4269 Koi rahe ya na rahe mai apke sath hu salman bhai 1 1
4270 Salman Khan tere pean dee phudhe mara kece randi k beachy 0 0
4271 Love youui bhaiiiii 2 2
4272 BHAI SE BDA KOI SUPERSTAR NHI H DUNIYA ME 2 2
4273 Sala hrami muslmano ka dushman badnam kiya hai Muslman ko kalank hai kabrustan mai tera paisa kaam nah i aayega balki #aamaal aor sabse pahle #imaan #pakka hai ya nahi 0 0
4274 Sahi h fir agar aisi baat h to .....Jo log afwah uda rahe unhen ye sab nhi krna chahye......Islam jhut nhi sikhata h 0 0
4275 Yes i knw u r a gener0us n p0stv prsn i belev tht c0z i felt n obsrvd tht:-) 2 2

4276 bb dekhenge pakka 1 1
4277 Salman Bhaijaan ye sab un logoo ki chal hai jo ye chahtai hai ki app ki image ko nukshan ho 1 1
4278 Yes puri duniya aapke khilaf ku na ho jaye mujhe aapke upar pura bhrosa h or marte dum tak rahega salman bh aijaan love you 2 2
4279 salman sir aap bohut piyaare ho mashallah 2 2
4280 dusroo ki ni khud ki suno.....koi na hatthii chalti h to kutte bahukktee h...... 0 0
4281 kya tum ko pata hai ke tumhara kya religion hai neither muslim nor hindu 1 1
4282 chup karo paglo rulaoge kya .. senti batee karre sab :'(lolz 0 0
4283 salman khan to ghode bech ke sogaye hoge. aur yahan machi market banare sab . sojao salman ke deewano 1 1
4284 theater me ja k salman ka movie kabhi nahi dekhunga Internet pe 10 din k baad aayega download kar k dekhun g!!! free me 0 0
4285 sir aapse milne or baat krne ki bahut ekchh thi 2 2
4286 Insan ki koi cast nahi hoti ji ..aj dont wory 2 2
4287 Harami ki aulaad hai salman 0 0
4288 aise nahi bolte sar ko.... 0 0
4289 Jai ho jai ho jai ho. Bhajrangi bhaijaan 1 1
4290 To hum kya karay 1 1
4291 Hai salman i' from idonesia&maley, god 1 1
4292 jitne v salman ko gaali de rhe hai. sb owaisi ke dogle hai. sala sb galat msg failata hai owaisi supporters 0 0
4293 Bhaijaan aapne sahi baat kahi hai ....hum sab ak hain hume faltu rumer se bachana hog a..... 1 1
4294 khano mn khan salman khan jan 1 1
4295 Mashallah 2.3k comments and likes r 33,966 great 300 crores hai bhai ki film kai 1 1
4296 waitingg for bhaijaan 1 1
4297 bhai aap tension mtlo humlog aap pr bht trust krte hai yeh films sab ki 300 crs kmayegi ... 2 2
4298 Bhaijaan yeh msg , apke Muslim brothers ho ghuma rahe hai apko badnam karne ke liye 0 0
4299 sir inshaalllah may ap sy ek bar milon ga.sir ap sy galay mil jao ga.n 1 1
4300 sir kasam sy may apkay movies ka muntazir hon.sir apki torri si role bi mjy achi lagti hy...sir mjy bhulaao pllzz z sir 2 2
4301 salman apki sch bht achi hy.... ALLAH PAK sb thek kren gy .ap preshan mt hn. stay blessed 2 2
4303 BiNa muslim Ki hit karke dikhyenge hm 1 1
4304 19 ko eid h or wo apni eidi lene zaroor ayega. 1 1
4305 Ab eid celebrate krta ha kiya ramzan ka roza b rkhta ha.kyn k eid ka maza tb hi ata ha jb ramzan k roza rkho. 1 1
4306 Yaar sallu bhai accche bande h to sala sab inke naam ko kharab karne me lage pade h 2 2
4307 Hlo sir kya aap kbhi mujhse baat krege kuchh hai jo aapse share krna chahti hu 1 1
4308 Salman bhai ....no tansion hm tumhare sath h ...koi frk nhi pdta hme ...koi bhe kuch bole aapke killaf 1 1
4309 ok koi gal nai ji salman bhai 1 1
4310 sallo mai ap se janna chahti ho kya ap n ye bola h hindu bhai mere liy q bola ap ko muslim bhi to dil s chahte h 0 0
4311 Mai WO pars buy Kar k use mai paise rakh k use gift reaping karwaa k eid waale din namaz k baad jab Ammi se gale milkar eid Mubarak karungi tab mai ye gift unhe dedungi 1 1
4312 i know mai janta tha ke sallu is tarha ke bekar bat kbhi nhi bol sakta 1 1
4313 Bhai ajkirat appkesat 1 1
4314 allaha aapk sath hai bhai 2 2
4315 Bhai jaisa ap bolo waisa hoga 1 1
4316 I knw sallu i dnt belive fake news u dnt wry ... 0 0
4317 Hey salman bhai .kab Milo ge 1 1
4318 Eid 2015 Hum sath sath bajrangi bhaijaan. 2 2
4319 love u love u love u sallu? 2 2
4320 eid ki part meri traf sye bhaijaan 1 1
4321 aap galat na the, na ho, na hi hoge. bhai aap ki sadi ka intijar pure world ko hai .kab super duper news de rahe ho aap. 1 1
4322 bhaijaan love yoy 2 2
4323 Hi salman sir please apse marnese pehale ek echha he ki main pako samnese ekbar dekhu filmo me dakhahe le kin ajatak main apko reall me nahi dekha but apko aur apke family ko ramzan eid ki mubarakbad 1 1

4324 Bhaiya plz ek bar mujhse bat kar lijiye main bachpan apki fan hu aur apko apna bada bhai manti ht 1 1
4325 Kya karke jaoge. Aap 1 1
4326 luv u salman ,...aap bht bht great ho...hr din koi na koi kisi k bare me bolta rehta..ese logo pr koi vishwas ni krt a.........unke bolne se hi hme sikhna cahiye pdai kitni jruri h n soch smjhkr bolna b. 2 2
4327 English ki maa behen ho gayi Bhai :v 0 0
4328 very good bhai mujy thori bohat samj lg rhi hai 1 1
4329 sallu bhai rockz 2 2
4330 Salman sir hme pta h. W8 1 1
4331 wee belive u salman 2 2
4332 Bhai hum aap par poora trust karte hai aur humme pta hai kabhi galat nahi karoge 2 2
4333 SK bhai aap shaddi kab karoge muje aap ke son ko dekhna hy 1 1
4334 Salmam sir hmesha apko to hi follow krtee h hmm. Apko dekh kr hi sikha h ki dusro ki heLP krna kya hota h. Dil ko khusi milti hh ab jana hmneeee 2 2
4335 Hum to apko apni jaan maante hai 2 2
4336 kya ho gyaa ? 1 1
4337 Salman bhai don't worry. Aane do salo ko aisa khinch ke denge ki bhool jaayenge ki saans kaha se le aur pa#*e kaha se.... 0 0
4338 Bhai..don't worry 1 1
4339 love uuuu sallluuuu 2 2
4340 Jise bhi apne profile pic par 1000+ like lena hai add kar ke massege karo only indians 1 1
4341 nyc sallu bhai.. 2 2
4342 Apake upar bharosa hai.. ..bhai! Aap koi galat kam nahi kar sakte.. .. ! 2 2
4343 BAJRANGI BHAIJAAN 700 CRORE!! 2 2
4344 I'm wid u 2 2
4345 Aabhe salman bhai ko suna mat lagaao vo kisi ki sunne vala nahi voto jo manme aaye vo karega bhai 0 0
4346 Md noman teri behan hai wahan pe 1 1
4347 Salman je plz kbhi to rply kiya kro tbhi pta chal e ga ki aap bhi apne fens se pyar krte ho 1 1
4348 salman bhai jaan friend request plz 1 1
4349 Kha h aap pls kbhi aek msv kr dijiye plss I realy miss u 1 1
4350 yes bhaijan hm ap k sath hai 1 1
4351 hamy apky real life se koi sarokaar nahi. ap actor hu aur apki mvz dekty hai thats it baqe ju karu ya na karu ur l ife 0 0
4352 sahi h bhai good evning salman sir 1 1
4353 Shadi kr lo wrna firr na hogi 1 1
4354 Shadi nhi hogi yaar aur kya 1 1
4355 Niki varma tum bhi bol lo 1 1
4356 Salman gadi aaram se chlana ab wrna jai..l 1 1
4357 are yah madhar chud kiya dega iski to me ma chud dunga agar yah mil jaye yah madhar chud islam ka dusman ha is sale ko bech rod per lakar goli mar du ise mar duge to zanat miljayegi kiyu ki yah madhar chid islam ka dusma n ha 0 0
4358 Pta h. Ye sab fake news ghum ri h aj kal. Bt shayad flm pe iska asar padh sakta h. Ap wts ap pe vdo share kro k apne kuch ni bola muslims k khilaf. Insha allah sab thk ho jaega 1 1
4359 Hy tamanna i wnt 2 frndship wth u 1 1
4360 salman bhaiya aap shadi kab korogi? me apki shadi ki barat me jana chahta hu 1 1
4361 Sallu kya likha hai kuch samajh nhi aaya 1 1
4362 Salman Bhai jaan hamare dil me basa hei 1 1
4363 ilove u .. bai.. .. im soo sad ur arresst.. 2 2
4364 hi bhaijaan apko apki film bajrangi bhaijaan aur eid ki advaance mai bdhaai love u bhaizaan 1 1
4365 Mera Islam mera mazhab. Ik allha ik qhuran . Tu kya samjaye ga hume 0 0
4367 Are yar to to me ghar aawunga bhai biryani khane aa sakta hu na bhai 1 1
4368 bhai mein aur hamara kolkata ke sabhe log appka film zarur dekhaga 1 1
4369 pls all frendns wach an the movie bhai ne Yeh harare liya present kiya sab log pls Yeh film dekha yaaya 2 2
4370 Chance bhut kam hai mai dekhsakti hoon ya nahi 1 1
4371 bhai utha lo isko 1 1
4372 khud ko fans bolte hai lekin kabhi fans ban nhi sakte kyuki kabhi kosis hee nhi ki samjhne ki bhai ko 0 0

4373 Please everyone watching bajrangi bhaijaan......agar ap bhai ke fan ho to eidi to jarur dena bhai ko movie dekh kr 2 2
4374 SALMAN bhai ap tenshan mt lo apki movei supar dupar hit hai kuch m k l or kuch b k l hai jo apni (G) mra rh ey hai or jo mra rhey hai vo bhi apki movei dekey gey mgr adath hai na suleymani kida jo ghusa hai......:) 2 2
4375 Ye sab fake baatein h bhai sbhi religion key bht respect karte h or wo kbi kisi ko hurt nhi karte don't miss bajra ngi bhaijaan movie m to jaonga first day or first show 1 1
4376 aage aane wale time me tumko dekha de ge tumari aukat 0 0
4377 Pak ka movie flop hoji ??? 0 0
4378 Hmto chdnge nai bhai ki movie 1st day 1st show 1 1
4379 Bhai tum tention mat lo apke real fans jo pata ye ghatia harqat kisi aur ki hai , we have faith on u bhaiiii...?? 2 2
4380 ? belive of course buddy...i believe in your heart ...hameshaa buddy hameshaaa....i love uuu <3<3<3<3<3<3<3 <3<3<3<3<3<3<3<3<3<3<3<3<3<3<3<3<3<3<3<3<3<3<3<3<3<3<3<3<3<3<3<3<3<3<3<3<3<3<3<3<3<3<3<3 <3<3<3<3<3<3<3<3<3<3<3<3<3 3<3<3<3<3<3<3<3<3<3<3<3<3<3<3<3<3<3<3<3<3<3<3<3 3<3<3<3<3<3<3<3<3< 3<3<3<3<3<3<3<3<3<3<3<3<3 3<3<3<3<3<3<3<3<3<3<3<3<3<3<3<3<3<3<3<3<3<3<3<3 3<3<3<3<3<3<3<3<3 <3<3<3<3<3<3<3<3<3<3<3<3 3<3<3<3<3<3<3<3<3<3<3<3<3<3<3<3<3<3<3<3<3<3<3<3 3<3<3<3<3<3<3<3<3< 3<3<3<3<3<3<3<3<3<3<3<3 2 2
4381 Sir apse milna hi h mujhe kaise bhi karke mujhe bht tklif hoti h sir schme ab or nahi saha jata agr aap samjh skt e ho to please mujhe jarur milna h...apki fan Geeta Rawat.... 1 1
4382 Salman apke fans pahle apka dharm nhi.....apke Indian pahle hone ki vajah se pasand karte h. Aur apke sbhi ch ahne wale ka koi bi dharm ho.salman aap Hiro ho unke 2 2
4383 aj ki party bhaijaan ki taraf se. 1 1
4384 Hii bajrangi bhai jaan 1 1
4385 being human ka aslee avtaar. salman bhaii aap aur aap kay dad lakhon karoron logon ki duaa letay ho. mashAll ah ......subhanAllah. stay blessed always admin aamir ahmed 1 1
4386 Kaya hal salman bahi kamal karte ho pande g 1 1
4387 Salman bhai main apki baat ko samj saktahu aur yeh sab jhut hai 1 1
4388 Logon ka to kaam hi hai afwah failana 0 0
4389 Muslmano ki beizti ki hai is film mai 0 0
4390 mainu pura bharosha hai ki apki movie sare record tod degi 2 2
4391 koi gal nai mujhe pata hai aap bahut busy rahte hai all the best veerji 1 1
4392 Es i love ka ryply tab tak ayga jab tak tum kuch nhi khta 1 1
4393 gdnt salman khan.kash ap reply karte. 1 1
4395 Salman bhai i love you nikhil raj hemanshu raj nikhil par frend request kijiye ga salman bhai pls pls pls 1 1
4396 jada pine se accha , todi si piyo na bina pani ke , paise ki bhi bachat aur pani ki bhi. kya bolte ? 1 1
4397 Koy musalmaan ye film nahi dekhe ga ye film flop hogi 0 0
4398 Me aapki bhut badi fen hnu sar.aap aek baar muje bhi kuch riply karna.p 1 1
4399 Bhai don't worry 1 1
4400 K bole6e,..Tui ki janis anti religiÃ²us film kake bole,.. 0 0
4401 eid mubarak ho bhai jaan love u 2 2
4402 Osm mubi super duper hit sir 5000000 ke kam se jada kamaygi ye film sir 2 2
4403 piz contect me 1 1
4404 luv u bhai waiting____ 1 1
4405 Shopkeeper khai ka 1 1
4406 I don't know bt I want to ask.. K log bol rahe hai k salman ne bola hai k mujhe apni film hit krne k liye musalm an log ki zarurt nhi... Agr aapne aisa bola hai to movie eid me q release kr rahe hai eid baad karo 1 1
4407 i am bigg fan of salman khan iam pakistani love love uuuu salman khan 2 2
4408 assalamualaikum ..... salman bhai me ye puchna chahti hu k jo baate sunne ko milri hei muje is film se related. kya aapne sach me esi koi baat boli hei ya ye sab rumors hei .... plz tel bhai jaan 1 1
4409 Salman sir aapki movie hit hogi 2 2
4410 Book kr diya bhai 1 1
4411 zakat milegi is eid pe 2 2
4412 Okkk....bajrangi jaan 1 1
4413 Kab aayegi yeh eid... 1 1
4414 Bhaijan ak hi hain.....SALMAN...... 1 1

4415 salman bhai jay ho 2 2
4416 Salmanu mashallah Bahut hamsome ho!!!!khush raho!!! 2 2
4417 aapne achha nahi keya 0 0
4418 jay jay bajrngi bhai jaan 2 2
4419 Me tumari movie nahi dekoga jabtak ke app mujse batnahi karte 0 0
4420 mast movie hai Bhai 1 1
4421 Love uuuuuuuuuuuuuuuuuuuuuuuuuu bajrangi bhaijaan 2 2
4422 Nitin Saxena bhai order krdia kya??? 1 1
4423 Superb mand bloing 2 2
4424 sab ki aan sab ki shan sab ka ek bhai jaan ,,,,,,my bro salman khan 2 2
4425 Salman sir evry1 says m ur die heart fan, ,bt m ur huge huge huge huge huge huge crazy crazy die heart fan of urs 2 2
4426 luv u soooooooo much salman....aap ke fans hmesha aap ke saath he... 2 2
4427 Bhaijaan of bollywod salman bhai 1 1
4428 Bhaijan love uuu 2 2
4429 mujhe mil gaya haii..... 1 1
4430 Salman kaso ho 1 1
4431 Bahut kam logo ko saccha pyar milta h 1 1
4432 Assalam walekum bhai 1 1
4433 Salman ji aapki film super duper hit ho.sare record break kre.Ginnes book of record me naam ho jaye aapki fil m ka.Aap reply kro ,ya na kro ham to aapke liye pray krte hi rahenge.Amen!!!! 2 2
4434 Kya haal hain..pawan kumar chaturvedi g..urf. bajrangi bhai bhajaan 1 1
4435 Tu madharchod hai 0 0
4436 Sallu bhaijan ki jai ho sallu bhai pure hindustan ka tiger 2 2
4438 Trailer dhaaaansu hai yaar 2 2
4439 Trailer dhannnsu hai bhai 2 2
4440 Nice meri jaan 2 2
4441 Eid mubarak Mian Salman vai 1 1
4443 I love you salman khan my aap ki bhut bhut jada fan hu 2 2
4444 dosto yai flim tho fulle hit pef hit hoge 2 2
4447 Nawaj ne tohh kick bcha li iss baar dekhte h 1 1
4448 salman khan aap pakistan aa rahan par kon se city aya ga plz plz humko bhi aapse milena han plz plz batyan na 1 1
4449 Pori dunya tumhari fan nahi hai to ziyada ochlo mat agar tum harai fan ho to wo sirf india or pakistan ho ga ya pir AK or mulk pori dunya nahi Mari sister tumhai pehlai like krtiti per ab nahi she is right you are salfish 0 0
4450 blockbaster movie bajrangi bhaijaan... 2 2
4451 Salman bhai 1 no 2 2
4452 gud see vaaijaan 2 2
4453 bhai aapki ye movie chhappar far dega ;) 2 2
4454 Bhai jaan locket badiya a 2 2
4455 Supr salman bhaijan 2 2
4457 Super duper hit.rahegi bhai 2 2
4458 Wai sale Ismail kutte jyada bhaukana sehat k liye nuksaandayak hai . Amma k pallu me so jaa ab ## boxdk 0 0
4461 Pakistan m yeh move nh chil sekte chair parr de hum cinema wallo ko lhr m tu kabhi nh 0 0
4462 Abe ismail pakistan me chal v jaegii to 1 cror se jada okat kya h tum logo ki be 0 0
4463 Bajrangi Bhaijaan sbka bhai 1 1
4464 Tere lahor se mahga hamara gav h be ja apna kam kar 0 0
4465 Ye Pakka hit h koi sala rok Ni sakta bhai ke film ko hit hone se 2 2
4466 Salman bhai hum to apne bach pan sey like kartey they abibi kartey hai lykin aapne ek baat kah ke hamey nara az kardiya 1 1
4467 salman khan aapne kya kaha h 1 1
4468 esaa kyu bolte ho aap 1 1
4469 Sbka record todke Salman bhaiJai jai bajrang bali 2 2
4470 sala kuti ki olad 0 0
4471 i love u soooooooooooooooooooooooooooooooooooooo+00000000000+00000000000000+000000000000-00

0000000+0000000+0000+0000+000+00+0 Mich and u r beautiful+awesome 2 2
4472 Ye movie hit hone se ruk he nhi skta h movie hit hoga hit hoga hit hoga---------------------- 2 2
4473 kisi ko blcne chahiye to apna numbr btao abi 2_ min me blance aayeg 1 1
4474 Salman bhai I loveyou 2 2
4475 Bhaijaan maine liya hain amazon se 1 1
4476 Salman bahot she log aapke baare me galat baat bolte he muje acha nahi lagta 1 1
4477 Plz salman bhai ke bare mein ye galt baate mt likho unhone aapka kya bigara h 1 1
4478 Rock salman bhaijaan jaisa koi ni 1 1
4479 Paglii hy kya tu Aliya Khan mene bola or tu sch man ri hye 0 0
4480 oye alia privacy block 1 1
4481 Ha ha ,are yha animals kha se agye sb humans hye padli chshma lga ke dekh 0 0
4482 Mene kitni bar smjhaya tha rat ko chashma mt nikala kro animals njr ate hye ap ko firr 0 0
4483 Bhai phela din phela show love u bhai 2 2
4484 Oye hoye kini khrab ldki ,request delete kr dii meriii 0 0
4485 Bollwood ki aaan baan saan dekho aagaya #Salman_khan movie me karenge Dhamaal kyo ki ye hai #Bajrangi _Bhaijaan 2 2
4486 hi sallu bro.... 1 1
4487 salman bhai bhool gye kya 0 0
4488 Mai ho fane aap ki bahut jiada her movi jis Mei aap ho kush ajiv khushi apnapan aap say lagta ha bha bat guan ba say dua karty ha Mai request behg do pls bat Karni ha 1 1
4489 I want it i want it i want itttttttttt ??? 1 1
4490 Salman khan sab sy best 2 2
4491 Supar hit bahi 2 2
4492 Vai apka koi vi filim hanne dekna nihei chora.... 1 1
4493 Salman vhai fata fati look 1 1
4494 good morening salaman bhai 1 1
4495 Kamal ki movie hai kasam se dhum macheg dhum..Salman sir....i love you.<3 2 2
4496 Salman bahi super duper hit hai meri dua hai apke sath L0ve u bahi 2 2
4497 salman bhai ne apka bahot bda fan hu 1 1
4498 Gd mrng bhai 1 1
4499 gm sAllu bhai 1 1
4500 Fast daey 70 crore bahijaan 2 2
4501 Salu g khbi hello hi tou kr lia kr 1 1
4504 Love u salman bhai ...apki movie aaj tak ke saare record tod de ye bhagwan se prarthana 2 2
4505 chor hai ye 0 0
4508 bhai jan duaa hei k appki film sooper dooper hit ho. 2 2
4510 Keya sach me salman khan ho mujhe jawab dijiye 1 1
4511 supprrrrrrrrrrrrrrr salllu bhai 2 2
4512 Looking hottt salman... 2 2
4513 Inshaallah jobi hoga fer aye ga, 2 2
4514 Agar muslim ki nam badnam huya to teri page unlike kordun ga or kavi bi tere liye duya nahi karen ge#@Me s ulltan@ 0 0
4515 i love u vaii.khuda hamesha apki saath rahegi vaii 2 2
4516 Sir.sera ke bad me mai aapka bodygurd rhunga.. 2 2
4518 Musalmano plz open ur eyes salman khan only naphrat ke layak hai 0 0
4523 Super super super salman bhai 2 2
4524 janno ki jaan bajrangi bhaijaaan. 2 2
4525 suwar ka bacha 0 0
4526 Bhai jaan ko salaam 2 2
4527 salu g plz ek bar bt kr len mjhse EID se pehle hi meri EID h0jaegi plzzz 2 2
4530 movie to hit tio hogi sure bhai ki movie wo v eidd pe 2 2
4531 salman flap ab 0 0
4532 Allah ap ko sada khush rakh. aameen. 2 2
4533 Shahid kapoor ne tumhe invite nahi kiya @BB 0 0
4536 vrryy vrrrry nicee trailor 2 2

4537 bhai jaan humne suna hai jo apke kreeb hote hai ap unki har vis puri karte plz bhai ek baar mujhse baat kar lijiye g.e. bhai love u always 2 2
4538 Nice movie 200 crore asani se kama legi 2 2
4539 Superhit 800 crores 2 2
4540 300crore aram se kama legi 2 2
4541 nice tobe thiseid eve,,,,,eidmubarak 2 2
4542 Sabaka bhai salu bhai 2 2
4544 salu bhi i your biggest fan jai bajrangi bhaijaan 2 2
4545 Very very niece tailor 2 2
4550 mai apka ek chota sa fain hu 2 2
4551 Osm pic sallu 2 2
4553 lovee uuuu saluuuu 2 2
4555 Oooops wow yar 2 2
4558 Onliy dabang bhai 2 2
4561 sab ki aan sab ki saan aagya bajrangi bhaijaan 2 2
4562 Salman sir...<3 2 2
4563 Gud luck ji.. allah aapko sari khushiyo say navajay ..sayad aap humko bhul ghai lakinhamari saash rukh jayghi hum ushkya baad bhi aapko nahi bhulayghay.. allah hafis 2 2
4564 Kash kabhi aap humay samjh patay dosti karna ashan hai nibhana mushkil I love you salman khan 2 2
4565 advanced eid mubarak 2 2
4568 Hum iske baap hai 0 0
4571 Sir i love u. MAIN aapka bhot bada faan hun,sir. Aap jo T-shart pehnte ho mujhe dedo Sir. Main HARYANA se hun Sir ji.my No. Sir 9991865726 2 2
4574 Wah wah suprb salman khan g gudluck??? 2 2
4576 #sona kya baat he.. 2 2
4578 Mojh par ik ehsaan karna k moj par koe ehsman na karna 1 1
4580 salman sir main aap kaa bahout bada fan hu please call me 7726066179 2 2
4585 bahubali kar rhi h record tod collection 2 2
4586 bhijaan eid mubharak...... 2 2
4587 bajrangi bhaijaan 347 caror box offic 2 2
4588 Salman Khan Tum Kafir ho Muslim larki ko bagatai ho sharam karo aj be teezi see pilnai wala Religion islam hai 0 0
4589 Mast hai bhai 2 2
4590 Bhai Jaan original kon se fb par ho kiss naam SE hai 1 1
4591 Sala Kafir bahenchod dekhta hu teri movie kaise hit hoti hai . . . . . ! 0 0
4592 shayadi ke bare me aapka kya khayal h. aap shyadi karoge ya fhir kaware rahoge. muje bahut badi tanson ho ra hi h. 1 1
4593 Abbu Ki shopping hogai bas Ammi Ki shopping baaki hai 1 1
4594 Salman agar tu ne mujse bat nhi ki na to samjo. Tera mera qissa khtam finish 0 0
4595 Salman tu reply q nhi kar raha please please 1 1
4596 Please salman tu to janta hai k tere page pe kese kese log hai chiiiii aap ek reply do na please 1 1
4597 Salman yaha aur bi bahot se bandar hai par mai sirf tum se pyar karti hu please reply me 2 2
4599 eid Mubarak ho bahiya 2 2
4600 kuch nahi bas report send kar raha tha 1 1
4601 im Rohit vasfoda 1 1
4602 Sale sab accha krta he agar comment pad rha he to sonle kuch logo ka dil bhi bohot bura todhta he ab tak sare first show dekhe lekin isbaar tone dil todhdiya sallo 0 0
4604 i m ur big fan.kash ek bar apko dkh pati.aur mai zarur augi ek din 2 2
4605 aap kaese hai aor kaha par hai. 1 1
4606 sb salman ko doshi kyu keh raha.khud ko sb dekho phle..duniya bs bhaukti hai 2 2
4607 tri maa ka choot 0 0
4608 Aaj ek se bdkr ek ghatnayen huyi hain pta nhhh kya huwa h... 1 1
4610 I love you Salman sir kaash main apse baat kar pati..i miss you..<3 2 2
4611 Ek Salman hazaaron mehaman...hahahahahah ....sabka haal ek jaisa bolo kya hoga??? 2 2
4612 Salman sir ki chandi hi chandi..(: 2 2

4613 salman tum garibo k dard samjhte ho you are grateman 2 2
4615 hy bodegurd you my friende.love you.... 2 2
4616 Mai tho supper man salmaan ka fan Jo leve panga kardo ma bhean I'd Mubarak sir 2 2
4617 film ka story mast he aur aap bhi mast ho............ 2 2
4618 I hate u salman khan tum jesa insaan maine duniya me kahi nai dekha 0 0
4620 Salman Khan bhai can't wiat...wish aaj hee friday ho... Bajrangi Bhaijaan..in advance, eid mubarak 2 2
4622 eid mubark salman bhai 2 2
4623 eid mubarak ho aap ek baar reply karye pls 2 2
4624 eid mubark bhay 2 2
4625 Akdam kick ke jesa 2 2
4627 hmmm kal muj bhi yahi feel hua tha 1 1
4628 raat bhar jaaga raha..tujhse she kahna chaha..mujhe tera ab bhi tera injaar Hai..mujhe ab bhi thujhse pyaar haiâ €" feeling ekta 2 2
4629 Kaise ho Salman sir...id ka kya huwa apke ??? 1 1
4630 aapki movie 350 crore kamai karegi 2 2
4631 bad karna chahti hun mud thik nahi hai sab so rahe hai mammy aur chhoti bhan college jane ke liye jaldi uthti hai aur rat me bhi 12 1 baj jata hai sote isliye dophar me so jati hai papa bhi so rahe hai 1 1
4632 nis pic salman ji. 2 2
4633 Sallu u jst fav 2 2
4634 Bhai movie hit hai tention lene ki zaroorat nahi 2 2
4635 Best acter.....salman khan 2 2
4636 I love Salman khan i love india 2 2
4639 EID mubarak aur hit (film)mubarak advance. 2 2
4640 300, crore insha Allah 2 2
4641 Salman Khan Bhai im wid u wid all ur movie? Inshallah ek baar nhi ..jitne baar ho sake utni baar jaoga bajrang i bhaijaan? baaki logo bhi yehi advice doonga.. 2 2
4643 mil gaya bhai aur maine order v kar diya hai Jai bajrang bali 2 2
4644 bst Of luck dear salman khan ....<3 2 2
4645 abhe yeh really salman ki id h kya 1 1
4646 or bhai sub ko bol do kye ap mare bhai ho ap ko kasm hai ap ke bhai bhai samj kar agar ap moje bhai samj te h o to facebook par boldo kye babar khan mangi mara friend nh balke bhai hai ap ki bhai babar 2 2
4647 bhai ap kub facebook par bheet te ho ye bata ho 1 1
4648 bhai jaan ead mubarak supose 6caror in bajrangi bhaijaan 2 2
4649 veri nice song bhar do jholi salman bhaijan 09627010944 plz cl me plz plz plz 2 2
4650 Main up jasi hona chati hon.apon ka stayle or teri stayle vi ek jasi.apka dil me himmot ha to mujhe ek moka di je.so godbless me. 2 2
4651 Favourite salman khan bhai eid mubarak ho 2 2
4653 Is film me hindu logo ki baout inshirt kari ha salman ne bajarangi ki wat lagi raki ha Sam to sam PK 0 0
4654 Bahut galat kiya aapne salman bhiya muslimo k khilaf gye h ap 0 0
4655 You r great Salman sir....Aap 1.Actor baad me ho pehle 1.Achha insan ho....Ummid hai Aap hindu or muslim k e sath aa rahe apwad jank bayano ko gour nhi farmayege... 2 2
4656 sala salman tare aukat kya hai hmm chutya gandu ....ek waqt ki namaz padhta hai kya ....aur abhi tak ku wara hai bhadwa sala 0 0
4657 2o15 biggest flop flog filmo me se ek film hogi flop flop hogi aur koy musalmaan ye salman kutte ki film nahi dekhe ga 0 0
4658 Teri maa ki aur Teri bahan ki bur mather chood 0 0
4659 woowww! bajrangi bhaijaan superhit hogi... meri bhagwan se prarthna he ki yah film super-duperhit hogi... 2 2
4663 bhi..movie blockbuster hogi bhai 2 2
4664 Supar hai boss 2 2
4665 Eid Mubarak bhai jaan I miss u i miss u 2 2
4666 Allah kare ki app ki film bajrangi bhaijaan south therator mey reliz ho jaye 2 2
4669 trailer me hi dhasoo maza bhaijan namaskar 2 2
4672 Randiko ban ko film kab release horaha hein 1 1
4673 Salman bhai bas tum hi tum ho industry me jo chaye rahate ho aur dusara kai nahi 2 2
4674 Salman ji sirf mera hai .....hai naa salman aur oss k lye main kuch b kar sakte 2 2

4675 kya ye picture bhai ki hit hogi but i dout 1 1
4676 bhai vo bache ladki aapke sath kareena se jada cute lag rahahe. 2 2
4677 bhai sab ready ho jao kal first day first show kuch bhi ho jaye 2 2
4678 Muslman bolte huwe saram aa rahi hai 0 0
4679 salman kuthe dine islam ka gaddar sharam o haya ke hudud se dur hai 0 0
4680 buri tarah se flop ho teri mv sale bajrangi jake cast chng kr phle h#### 0 0
4682 eid mobarak ho bhaijaan aap ko 2 2
4683 yoo....hit hitZ nd hits 2 2
4684 Lol haramzady apnay tu musalmano ko zalil kya allah apko hidayet karay ameen 0 0
4687 Shukriya Bhaijaaaaan... Mere bday pe aj tak mila huva Sabse pyara tohfa.... Bajrangi Bhaijaaaaan.... 2 2
4688 happy eid salman sir 2 2
4693 bhai bajrangi bhaijaan pendant mila mujhe...Aaj ki party meri taraf se song launching par in JW Marriott Hotel me... thnx bhai 2 2
4694 I m from nasik bhai... main sirf aur sirf aapko milne mumbai aaya tha... 2 2
4696 hi salman bhaijhan eid mubarak aap ki movie achi chalegi i like u 2 2
4697 hmm hrt touching moVieeee 2 2
4698 bhai'great movie 2 2
4699 I wsh u a vry vry hppy eid mubarak. 2 2
4700 Movie dekh ke..aansu aagya yar..Very good movie 2 2
4701 Eid mubarak ho bhai jaan 2 2
4702 Bhai fadu hai 2 2
4703 faddu trailer bhaijaan. ... 2 2
4704 superstar salman bhai 2 2
4705 love u bhai jii salman khan 2 2
4710 Hii very nice... Salman sir apko meri taraf se eid ki bahat bahat subhkamnayen..... 2 2
4711 flop. thaki huee moviee. bigg flo[0 0
4712 Trailer tho badiya hai. Nd hero bhi handsome hai 2 2
4713 awsome trailer <3 2 2
4714 koi dusraa naam nai mila kya, is se pehle bhi Hero naami kayi filme pit chuki hai.. 0 0
4715 Is movie ko salam bhai bhi nahi bacha payenge ? 0 0
4716 Salman bhai ki produce ki huvi movie achi na ho ehsa ho hi ni skta.... 2 2
4717 Jabrdsth bhai jaan.. 2 2
4718 hurkuchhhhh ultimate ....blockbuster 2 2
4719 supperrb salman khan.. 2 2
4720 nice salman bro 2 2
4722 dekh liya bhaijaan.. mast h 2 2
4723 kucch naya kahaani nahi hai sirf action mai jayda focus kiya hai new action hero .... superhit because of sal man..... 2 2
4724 es apps ko downlod kro sare 1 1
4725 K mehu hero tera..... amzing song.. 2 2
4727 Bakwaas hai bhai..Bas apne movies pey lagao. BB 500 Crore minimum guaranteed!!! 2 2
4728 Wah maja aa gaya. 2 2
4729 Hi salman bai 1 1
4730 Howar you salman bai 1 1
4733 Bhai jaan kuch kami si lag rahi h... Pata nai kyun..... 0 0
4735 salman bhai zindabad...love u bhai... 2 2
4736 Bajrangi bhai jan machai ga abki bar dhoo mai ehsan 2 2
4737 Mast hai bhaiiiii 2 2
4738 Baat na bani.... 0 0
4739 Super hai bhai suraj is rocking 2 2
4740 Bekaar hoe gi 0 0
4741 1 no trealir bhai 2 2
4742 Asslamualeykum bhai jaan 1 1
4743 trailer mai salman khan nahi hai nahi dekhonga 2 2
4745 Salman bhai aap hoge jis movie mai to hi jaunga dekhne 2 2

4746 Ye raha almost jail sey bhaga hua mujrim 0 0
4747 new opera bhi install kiya tha maine abhi usme open kiya hai 1 1
4748 kya likhu han 1 1
4749 Dekh rahe ho ya nehi? 1 1
4750 Superb floop salman khan 0 0
4753 Salman khan bolliwood ki shan banchuka back to back blockbuster superhit movi name dabbang. Redy. Bodyguard. Ek tha tiger . Dabbang2. Jai ho. Kick. Bajarangi bahizan superhit jayagi 300 fix 2 2
4754 Awsome..miny salman khan. 2 2
4755 mast bhai jaan 2 2
4756 i alsooo watcing thiz itx Fabulouss movie hmmmmm 2 2
4757 Sab ka bhai salmaan bhai love you salmaan 2 2
4758 Agar tu asli mard ka bacha hoga toh jawab jarur dena 0 0
4759 salman khan Tera jo being human ka fashion show kiya tha us mein ek model Dress pehna tha jis mein ALLAH likha hua tha, jab se hum tere se nafrat karte kamine sale salman khan kutta 0 0
4762 awsm trailer with dhasuuuu fights 2 2
4763 bahut pasand aaya 2 2
4764 Abey thuuuu hai tujh par teri film par aur tere trailer par. 0 0
4765 I shw ur movie trailor i like ur al movie. 2 2
4766 nyxc trailer bhaijaan 2 2
4767 Bhai jaan agai ap reply daitai ho to ek reply or dai daina. 1 1
4768 Aapki film k liye aapko dher sari shubhkamna 2 2
4769 salman bhai mah ap ki philm jaror dekunga 2 2
4770 Yar pooja kya ho gya ha aise bat ni krte 0 0
4771 neeraj kutte.kya majato aayega majato aayenga sale kutte 0 0
4773 salman bhai sooraj pancholi ka offical fan page ka link apne wall pe post kardey ... 1 1
4774 sir, can't wait fr #Bajrangi_bhaijaan <3 2 2
4775 feeling sooo excited fr tommorow .. :) #Bhaijaan <3 2 2
4776 Bhot sahi bhai... Bhai bhai.... :) 2 2
4779 Bhai jaan ek number yar mana to caller tunes set bhi kar li nice one bro 1 1
4780 Laga liya ji 1 1
4781 Superb sallu jaan 2 2
4782 OLD BEST DOST GUD LAK HAMESA 2 2
4783 gdrrrrr awaz salman bhai kiiii love u sallu 2 2
4784 i love salman sir mera no 7535964415 ap ka bahutt tagda fenss hu i love yy 2 2
4785 salman yaar sudhar ja bhai Q krta hai gandi herkate yaar 0 0
4788 nyc...mene active kr liya... 2 2
4789 luv u sir. wah sir kya baat Hai. lovely voice. superbbbbbbbbbb idea. 2 2
4792 devil apke pi6e...nice bhaijan 2 2
4793 hello #salman #sir kya mujhe ek reply mila ga plzzzzzzzzzzz 1 1
4794 I laik you sallu bhai jaan 2 2
4795 i love u jaan mmmmuuuuuuaaaaaaahhhhh shona mera 2 2
4796 Khushboo Tripathi fast day kese jyogi 1 1
4797 Vermaa phone utthao yaar ???? 1 1
4798 Guyssss meri profile picture kesi leg rahi hai..??? 1 1
4799 Salman saale tu chorr h. Kamine kutte . 0 0
4800 Chal teri ma ki chuy 0 0
4803 Awesomee bhai jaan 2 2
4805 Kare chamara sale 0 0
4806 gGuyssss meri profile picture kesi leg rahi hai..??? 1 1
4807 Sallu Bhai maza aa gya 2 2
4808 Caller agar khud Salman Khan hua toh? O.o O.o 1 1
4809 Nic pic salman 2 2
4814 happy eid(eid mubarak ho bajarangi bahijaan) 2 2
4815 Salman bhaijan ko eid mubarak 2 2
4816 Love uuu bhaiii 2 2

4817 Nice sllu baiiii 2 2
4818 Mazay ka tha lolz komal fon uthao na yar :p 1 1
4819 kya tha woh u block 1 1
4820 Sabka 1 bhaijaan 2 2
4822 arey wah yrr 2 2
4823 Hlo sir kya aap kbhi mujhse baat krege kuch baat hai jo main aapse share krna chahti hu 2 2
4824 Mujhe bhi batao kya kuch ata hai apko Neeta V Raj 1 1
4825 Bhii jaan luv u 2 2
4826 Owsome tune<3 2 2
4827 Tere awaaz kutta ke awaaz Sam hi chor muslman hi to 0 0
4828 apka big fan me bangladesh me plz cal met u +8801719447771 plz cal sallu bhai met u fan 2 2
4829 sallu bhai apka fan bangladesh me plz cal +8801719447771 met u all news share me all time plz cal sallu bhai jai ho 2 2
4831 EID MUBARAK BHAIJAN. . 2 2
4834 Hmmmmm vai set kar diye.... bohut achha lag raha hai.... 1 1
4836 bhai ki bat hi alag hai. . .sb se hat k. . .bhai salman. . . 2 2
4837 Bhai... Aap ki original voice nhi hai.. 0 0
4840 awesome wap....bhai jaan.. 2 2
4843 I love u salman khan love u love u love u love love u so mach 2 2
4844 salman bhai bhai bhai bhai bhai 2 2
4846 abhi bhi sant rahu tumhara dimag to thik hai tumhe abhi tumhe samajh nahi aarha na jis din koi aur mujhe koi a ur lejaega sadi karke usdin samajh aaega tumhe abhi baithe raho tum 1 1
4850 Hallo bhaijan kaha ho 1 1
4851 Wow bhai jaannnnnnn 2 2
4855 agar kar sakte ho to ye kar ke dikhao 1 1
4857 Kal dikhega ka salman ka star dum 2 2
4859 Hangover teri yado ka 2 2
4861 bajrange bhai jan. flim ka nam kyu rakha sallu bhi 1 1
4862 Owsome look bhaijan 2 2
4870 Sala kamina sallu bhai ramjan mahineme bajarangi ban gaya kotta sala kamina sallu.... suno musalman bhaiyo aur behano es bar bi sallubhai ko ullu bana denge 0 0
4871 Hij do ka sardar bangaya sala bajrangi kotta 0 0
4872 Salman bhai app batao ki india me ajubaa kya kya hai ek to Taj Mahal hai aur dusra kya hai mujhe app ka java b chaiye app kahte ho ki my sare coments padhta ho 1 1
4873 salu miya kaal aa rahe h hum bhaijaan se milne 1 1
4874 bhai is best bhai aap ki film kamayab hogi only salman bhai 2 2
4877 Javab nahi hai 1 1
4878 chl ht salman 0 0
4880 asalam o alekum salman bhai kse hn ap ap bht ache hn 1 1
4882 I love sallu bhai jaan 2 2
4883 Kal dekhna na bhule 1 1
4885 Sabko bacha kaho,sirf mere ilawa ok 1 1
4888 bhaiyo ke bhai 2 2
4890 mai salman ki diwani love u salman ji 2 2
4891 Kya chutiye log hain Jo ise eid Mubarak bol rahe hain is kamine ko kya mlm k ramzan kya hai aur eid kyu ma nate hain...ise to sirf paison se mtlb hai 0 0
4892 Banjrangi bhaijaan jaisa koi nahi ban sakta hai aur unki movie mashallah 2 2
4893 ye tb possible he jb aap hme bataoge k aap pak kb aoge kaha tehroge aur me aap se kase milo i promise ak dafa os baad aap ko cal kerne ka kbi nai bologi na i milne ka just one time plz 1 1
4894 Shadhe karlo bhai 1 1
4898 Kon se duniya hai hai muslim bhai. Jo kafir hai usko acha oor great bolray hai 0 0
4900 Agar koi Fat khatam krna ya health bnana chahta ho ya body bnana chahta ho to plz contact me +91950103788 1 1 1
4902 Apkeliya masig salman 1 1
4903 Wow is awesome hii iam Salman khan fone uthao yar khan fone uthao yar very very very nice voice iam so ha

ppy i love you Salman.<3 2 2
4904 Sallu miya ek bar reply dai daina apnai hand sai. 1 1
4905 Eid mobarak bahi jan 2 2
4906 handsome sallu <3 2 2
4912 rafu apko nhi bolna already wo dechyka hota h 0 0
4914 aapki pic hit ho ye meri tammnna hai 2 2
4915 Ya tumare liye bhi important nhi hoon 1 1
4916 hi khadus kya ho rha hai kha ho 1 1
4917 gold aur silver ke shear kharid ke bhul jao agar aage chal kar kabhi koi us businesses me jana chahe to shears b
hech kar original kharidle nahi to wo rahega aise hi 1 1
4923 Sallman ki photos uplod krke fb ko khrab mt kro 1 1
4924 suna ha ap sub comments parhtay hain :* <3 1 1
4925 Mubarak bhai aap ki film 1000 crore kama rahi hai aj ki party aap ki taraf se 2 2
4930 all the best+EID MUBARAK 2 SALMAN,LOTS OF REGARD2 ELDERS+LOTS OF AFFECTION&LOVE2
young! 2 2
4934 kr diya awsm yaat 1 1
4935 Madarchod supar dupar flop ho gae teri movieâ€¦BUHUBALI ROCXxx :) 0 0
4938 saale phale tu mera land mu se nikal tab na kuch bole ga 0 0
4940 yr apne Shi nhi kiya musalmano ko chetavni dekar 0 0
4941 Salman i love you pleze camm amaroc pleze i love you salman pleze camme pleze 2 2
4942 Mily tujhy Na dukh zindagi mein Phoool ki tarah mehky khuda kary Zinda rahy naam abad tak tera Eid ki khus
hiyan tujhy Mubarak khuda kary 2 2
4943 Mujhe salman khan se milna hai pls help me 1 1
4944 Kya ap hum se bat karoge 1 1
4945 Salman ke movie mai always dam hota hi hai frnds 2 2
4946 Bhai main apka bhot bada fan hun dil se bol raha hun.. bole toh apna bhai kaun sallu bhai .... jumaa mubarak w
ishing you all the best ur new movie. .. 2 2
4948 Kas ma aipka khas friend rahta our aipka karib rahata.kas ma kis.kis ma aip ka kareb hota.mubark ho.aipko roj
a. 2 2
4949 eid mubarak love u salman 2 2
4951 Jai jai bajrangi bali tod de dushman ki nali. seen fisrt day fisrt show amazing movie 2 2
4952 Salman ji kya aap bhi eid mubark manate hai please bataye ga please 1 1
4954 lv u salman..kya kr rahe h ap sona.. 2 2
4955 I'm superman but salman ka fan 2 2
4956 Sallu bhai mera no. 9861674485 ye mera ijat ki sabal he bhai jaaaaaaaaaaaaaaaAaaaaaaAaaaaaaaan 1 1
4957 heart touching movi 2 2
4958 aap bahut cute lag rahe ho 2 2
4960 Bhai meri sim docomo hai maine bht try kiya ny ho raha hai 1 1
4964 Cool movie bajarangi bhaijan osm ?? 2 2
4968 Salman sahab aap to meri aawaj sunenge nahi.Phir bhi aawaj laga raha hun. 1 1
4972 sir aap dil jeet liya kya movie ti 2 2
4979 Salman sir bahot acchi movie hai super hit blockbuster movie. 2 2
4981 awssmmm movie salman sirrrr super salman sirrrr 2 2
