# Supplementary material for: Mixed Script Identification Using Automated DNN Hyperparameter Optimization
Source: Comput Intell Neurosci. 2021 Dec 10;2021:8415333. doi: 10.1155/2021/8415333 (PMC8683192; doi:10.1155/2021/8415333)
Supplement: Supplementary Materials — (e.g., datasets or results outcomes in the form of graphs) from different stages are provided with the manuscript. The graphs including system training, validation, and the testing outcome of all RNN variants are included in Supplementary Materials. [file 8415333.f1.zip › 8415333.f1/saraiki.pdf]

چھل

جھلڑ

جھَل

جھتڑ

جَھت
تھَپڑ

تھَپ

پنڈڑ

پَنڈ

پگڑ

پگ
بھوتھڑ

بھوتھ

بھلھڑ

بھلھ

بگڑ

بگ
لڑا

لَڑی

کھوکڑا

کھوکڑی

رہڑا

رہڑی

دھڑا

دَھڑی

دیگڑا

دیگڑی

دَڑا

دَڑی
تَھڑا

تَھڑی

بڈھڑا

بڈھڑی

بیڑا

بیڑی
پلوڑ

پلوڑی

پھپھڑ

پھپھڑی

جھڑ

جھڑی
تھگڑ

تھگڑی

ترنگڑ

ترنگڑی

ترگڑ

ترگڑی

بَتڑ

بتَڑی

بھُگڑ

بھُگڑی

بناڑ

بناڑی

ٹٖوڑ

ٹٖوڑی

بٖڈھڑ

بُٖڈھڑی

اگٖاڑ

اَگٖاڑی
چیڑا

چیڑی

بٖکرا

بٖکری

چَلھا

چَلھی
تِڈٖا

تِڈٖی

تَڈٖا

تَڈٖی

بٖڈھا

بٖڈھی
بٖانھا

بٖانھی

مولھا

مولھی

کھاڑا

کھاڑی
کٹورا

کٹوری

ڈوڈٖا

ڈوڈی

ٹوکرا

ٹوکری
کنال

کُنالی

طاق

طاقی

ڈھینگر

ڈھینگری
راگ

راگنی

چھپر

چھپری

ٹوپ

ٹوپی
ٹبر

ٹبری

تھگڑ

تھگڑی

پہاڑ

پہاڑی

ڈول

ڈولی

تھال

تھالی

بوٹ

بوٹی
ودھیرا/ودھیری

وَدھ

مُٹیرا/مٹیری

موٹا

مَٹھیرا/مٹھیری

مَٹھا
لویرا/لویری

لوا

لُٹیرا/لٹیری

لٹّو

دَڈیرا/دڈیری

دَڈّا
چھٹیرا/چھٹیری

چھوٹا

جھکیرا/جھکیری

جھکا

پکیرا/پکیری

پَکا
ٹھُلیرا/ٹھُلیری

ٹُھلا

تھُلیرا/تھلیری

تھولا

تکھیرا/تکھیری

تِکھا

پھکیرا/پھکیری

پھکّا

بھَلیرا/بھَلیری

بَھلا

اُکھیرا/اکھیری

اوکھا
منگری

منگر

کٹوری

کٹورا

لوٹی

لوٹا
کوٹھی

کوٹھا

ڈولی

ڈولا

سوٹی

سوٹا
چھبی

چھبا

چولی

چولا

پیالی

پیالہ
بٹھی

بٹھہ

ٹبی

ٹبہ

ڈبی

ڈبہ
پگلا

پاگل

امبلی

امب

السوڑ

آلسی
عاشقو

عاشق

گٹِکو

گٹِکا

ڈھولنْ

ڈھولا
بنّاں

بنرا

کنوں

کنِل

پنہل

پنُوں

منوں

مُناں

مندھرٹا

مندھرا

ٹٹوری

ٹٹور
لٹوری

لٹور

موٹو

موٹا

سکٹو /سُکٹا

سُکا
نساوڑا

نَسنْ

لکھاوڑا

لِکھنْ

گِلاوڑا

گِلنْ

رُلاوڑا

رُلنْ

وَلاوڑا

وَلنْ

رُساوڑا

رُسنْ

پھُلاوڑا

پھُلنْ

پلاوڑا

پَلنْ

بھلاوڑا

بھُلنْ
گھلڑی

گھِل

کھلڑی

کھِل

سلھڑی

سِلھ
دلڑی

دِل

چھلڑی

چھَل

جھَلڑی

جھَل
تھلڑا

تَھل

تَلڑا

تَل

پُھلڑا

پُھل
کورڑی

کوری

ڈورڑی

ڈوری

ڈھورڑی

ڈھوری

پورڑی

پُوری

بھورڑی

بُھوری

بورڑی

بوری
گورڑا

گھورا

کورڑا

کورا

ڈورڑا

ڈورا
جھورڑا

جھورا

بھُورڑا

بُھورا

اورڑا

اُورا

لیرڑا

لیرا

گیرڑا

گیرا

زیرڑا

زیرا
پھیرڑا

پھیر ا

بیرڑا

بیرا

ایرڑا

اَیرا
بُلھڑا

بُلھا

چھلڑا

چَھلاّ

للڑا/للڑی

للاّ/للی
کلّھڑا/کلّھڑی

کلّھا /کلّھی

ڈھلڑا/ڈھلڑی

ڈھّلا /ڈھلی

چلھڑا

چَلھّا

ٹھُلھڑا/ٹھُلھڑی

ٹھُلھا/ٹھُلھی

پھُلڑا

پُھلا

تھَلڑا /تھَلڑی

تھَلاّ /تھلّی
پھَلڑی

پھَلّی

پَلڑاپَلڑی

پلاّ/پَلّی

بھَلڑا /بھَلڑی

بھَلاّ/بھَلّی
ویلھڑا

ویلھا

نیلڑا/نیلڑی

نیلا /نیلی

سیلھڑی

سیلھی

میلڑا /میلڑی

میلا /میلی

لیلڑا /لیلڑی

لیلا /لیلی

ریلڑا

ریلا
ڈیلھڑا

ڈیلھا

دھیلڑا

دھیلا

ٹھیلڑا

ٹھیلا
تھیلڑا/تھیلڑی

تھیلا /تھیلی

پیلڑا/پیلڑی

پیلا/پیلی

ٻیلڑا

ٻیلا

#گاٖلھڑا /گاٖلھڑی

گاٖلھا /گاٖلھی

چالڑا

چالا

جٖالڑا / جٖالڑی!

جٖالا /جٖالی
پالڑا

پالا

کھالڑا

کھالا

تھالڑی

تھالی

والڑا/والڑی

والا/والی

لالڑی

لالی

کالڑا/کالڑی

کالا/کالی
چھالڑا/چھالڑی

چھالا

جالڑا/جالڑی

جالا

تالڑا

تالا
ایڈڑا/ایڈڑی

ایڈا /ایڈی

لوڈڑا/لوڈڑی

لوڈا/لوڈی

چوڈڑی

چوڈی
جیڈڑا/جیڈڑی

جیڈا /جیڈی

روڈڑا/روڈڑی

روڈا/روڈی

کوڈڑی

کوڈِی

ڈوڈڑا/ڈوڈڑی

ڈوڈا/ڈوڈی

توڈڑا/توڈڑی

توڈا/توڈی

بوڈڑا/بوڈڑی

بوڈا /بوڈی
ہوڑکا/ہوڑکی

ہوڑا

چھالکا

چھالا

جالکا

جالا

بالکا/بالکی

بال

تھالکا/تھالکی

تھال

ڈھولکا/ڈھولکی

ڈھول
وڈکا/وڈکی

وڈا

!سوہݨکا /سوہݨکی

سوہݨا

وہڑکا/وہڑکی

وَہڑا
!کھبڑچہ /کھبڑچی

کھبڑ

'مندھرچہ / مندھرچی

مَندھرا

ڈانڈالچہ /ڈنڈالچی)

ڈنڈالی
مہرچہ

مَہرا

سکچہ /سکچی

سُکا

بغوچہ/بغوچی

!بھتڑا یا بھتوڑبباغ

بھَت

پھتوڑہ

پھیتا

جھٹڑہ

جَھٹ
پٹڑہ

پَٹ

پھٹڑہ

پَھٹ

بلوڑہ

بال
وَلوڑہ

وَل

#جھکڑا یا جھکوڑہ

جھکا

!بتڑا یابیتوڑہ

باتا

کھتوڑہ

کھوتا

جھلوڑہ

جھل

بندوڑہ /بندڑہ

بند
جالک

جالا

پھوتک /پھتک

پُھوت

لُولھک

لُولھ

ڈٖالک

ڈٖل

وِیلک

وِیلا

ڈھولک

ڈھول
کالٹا

کالا

بٖورٹا

بٖوری

پروٹا

پڑوٹ
سِلھوٹا

سِلھ

حلٖوٹا

جہال

سِروٹا

سِر
مِٹھویا مٹھل

مِٹھا

بھڑاکو

بھڑا کا

ٹھلُو

ٹُھلا

گَوڑو

گَوڑا

پتلو

پتلا

کالو

کالا
سچو

سچا

لمبو

لمبا

سُکو

سُکا
ٹِکڑا

ٹِکا

بکھڑا

بِکھا

چَھلڑا

چھلاّ

بَرڑا

بَر

جَنڑا

جَت

جِھکڑا

جھکا
ڈِوالی

دیوالی

نندر

نیند

تتر

تیتر
بگانہ

بیگانہ

مٹھا

میٹھا

کل

کیل
سدھی

سیدھی

چھل

چھیل

تکھے

تیکھے

تزاب

تیزاب

پغام

پیغام

پشمان

پشیمان

گدڑ

گیدڑ

سدھا

سیدھا

دوانہ

دیوانہ
نچھ

چھینک

تکھی

تیکھی

پودنہ

پودینہ
پتل

پیتل

بگانی

بیگانی

بمار

بیمار
گھنڈ

گھونگھٹ

چنج

چونچ

جتی

جوتی
پشاک

پوشاک

اکھلی

اوکھلی

گدام

گودام

جھمر

جھومر

ٹھک

ٹھوک

گھٹ

گھونٹ

سراخ

سوراخ

رزکار

روزگار

دھپ

دھوپ
چلھا

چولھا

پلیس

پولیس

اچا

اونچا

پرانْا

پرانا

دانْہ

دانہ

وانْ

بان

رانْی

رانی

پانْی

پانی

تنْ

تن

جیونْ

جیون

مانْ

مان

گبھنْ

گابھن

چھینْی

چھینی

چنْہ

چنہ

کتلی /کیتلی

کتنی
اتلا /ایتلا

اتنا

جتلے /جیتلے

جتنے

کہانٹ

کوہان
دیہہ

دیو

صحت وند

صحت مند

دھوڑ

دھول
تھولی

تھوڑی

ہکاڑا

اکیلا

تاڑی

تالی
کریڑا

کریلا

تھولے

تھوڑے

تھگڑی

تھگلی

روگٖ

روگ

واگٖ

واگ

بھوگٖ

بھوگ

پھوگٖ

پھوگ

چوگٖ

چوگ

پگٖ

پگ

بھاگٖ

بھاگ

سوگٖ

سوگ

جوگٖ

جوگ

ساگٖ

ساگ

راگٖ

راگ

جگٖ

جگ

رزکار

روزگار

پھوگٖ

پھوگ
ڈنگ

ڈنک

لوک

لوگ

ٹرنگ

ٹرنک
لوظ

لفظ

کھیرنی

فیرنی

پھیتہ

فیتہ
گدود

غدود

غستاخی

گستاخی

جگرافیہ

جغرافیہ
بالک

بالغ

غلاب

گلاب

غزران

گزران

غراری

گراری

دماک

دماغ

غمان

گمان
غستاخ

گستاخ

نغارا

نقارہ

گوتہ

غوطہ
ماف

معاف

تویت ، تویذ

تعویذ

اجاز

اعجاز

قیدہ

قاعدہ

بدت

بدعت

الان

اعلان

مافی

معافی

اتقاد

اعتقاد

اتبار

اعتبار
پیچک

پیچش

سٹوائی

شنوائی

قمیض

قمیص
سلوار

شلوار

مشوم

معصوم

تاس

تاش
بھیڈ

بھیڑ

وڈے

بڑے

کوڈی

کوڑی
بڈھے

بوڑھے

بڈھیپا

بڑھاپا

کھڈا

گڑھا

بڈھا

بوڑھا

وڈائی

بڑائی

وڈا

بڑا
ڈوڑی

دوہری

چپڑاسی

چپراسی

پڑدہ

پردہ
ملم

مرہم

خلیدار

خریدار

ڈوڑا

دوہرا

تھڑتھلی

تھرتھری

پڑوال

پربال

الماڑی

الماری

ڈٖینْ

ڈائن

ڈٖلی

ڈلی

ڈٖنڈل

ڈنٹھل

ڈٖنڈ

ڈنڈ

تڈٖا

ٹڈا

ڈٖنڈا

ڈنڈا

لڈٖو

لڈو

ڈٖاک

ڈاک

کھڈٖ

کھڈ

گڈٖا

گڈا

ڈٖاٹ

ڈاٹ

ہڈٖی

ہڈی

ورزی

وردی

ڈٖو

دو

چھیک

چھید
گرجھ /گجھ

گدھ

ڈوالہ

دیوالہ

ڈاڑھ

داڑھ
یزیز

یزید

ڈیوا

دیا

ڈدھ

دودھ
بدخ

بطخ

تماخوں

تمباکو

میکھ

میخ
گچھا /غوشا

خوشہ

ابرخ

ابرق

تڑاکا

تڑاخا
چرکھہ

چرخہ

بندوخ

بندوق

وساخ

بیساکھ
نخشہ

نقشہ

ملخ

ملک

دوزکی

دوزخی
متھاجی

محتاجی

ہل

چیل

متھاج

محتاج

لاج

جنڈ

جنڈ

جالا

جالا
مٹھاج

مٹھاس

ٹوٹا

ٹکڑا

ڈبّہا

دیکھا

تھکیڑا

تھکاوٹ

پالٹ

پالک

ورنڈ

وارنٹ
بکوات

بکواس

شیت

شاید

تہجت

تہجد
کوشت

کوشش

تویت

تعویذ

ڈہند

دانت
بھیت

بھید

شخت

شخص

مسیت

مسجد
پلیت

پلید

آندر

آنت

ٹانگا

تانگا

بٖوکی

بوکی

بٖوری

بوری

بٖوٹ

بوٹ

بٖچہ

بچہ

بٖور

بور

بٖیڑی

بیڑی

بٖوجھ

بوجھ

بٖول

بول

بٖوٹی

بوٹی

بٖیٖل

ببول

بٖڈھا

بڈھا

باِلٹی

بالٹی
بہر

باہر

بریکی

باریکی

بزار

بازار

شکیت

شکایت

شہانہ

شاہانہ

چلاکی

چالاکی

پھگنْ

پھاگن

پلانْ

پالان

پنجواں

پانچواں
برات

بارات

بدام

بادام

بدامی

بادامی
گاج

گرج

ہیڈ؎ یا ایڈ؎

اِدھر

ہک

ایک
ہفیم

افیون

ہوڈ؎ یا اوڈ؎

اُدھر

ہوچھا

اوچھا

اکھ

آنکھ

اشنا

آشنا

اثار

آثار
اواز

آواز

ارام

آرام

ازاد

آزاد
اخ

آخ

اسان

آسان

اواره

آواره

افرین

آفرین

اٹھ

آٹھ

اک

آک
اسمان

کھویجے ، اٹھیجے ، ڈیویجے ، ولیجے، روئیجے ، مریجے، جڑیجے
کھاوے ، حاوے ، پیوے ، سیوے ، پاوے
مرے، بھجے، اٹھے ، جمے ، حُلے ، چکھے ،جکھے ، جڑے ، جھرے
ہن، کھاندے ہو، کھاندے ہیں، کھاندے ہوندے، کھاندے ہوسن، کھاندے ہوسو، کھاندے ہوسوں، کھاندے ہوون ہا، کھاندے ہووہا، کھاندے ہوں ہا
کھاندے پئین، کھاندے ہاوے، کھاندے ہاسے، کھا چکے ہن، کھا چکے ہو ، کھا چکے ہیں، کھاندے
، ڈھورے، سنارے، غوشے، گاہلے ، وچھیرے ، ہیجڑے، اوپرے، پرائے ، چنگے ، گندے ، چٹے، کوڑے ، کوڑھے، سوہنے، وڈے، چھوٹے
ے، بانھے ، تڈے، اپٹے ، بچے، بلے ، پترے ، جھانولے ، چوہڑے ، چلھے ، دھاگے، دنبے ، کنوارے، دیرے، چھیلے ، چندرے، دیگڑے ، دلے
اوکھے ، بھور

پھبری

پھبر

ڈھینگری

ڈھینگر

دنبڑی

دنبڑ
ٹبری

ٹبر

تھپڑی

تھپڑ

جھجھری

جھجھر

گندڑی

گندڑ

کھنگھری

کھنگھر

پھپھڑی

پھپھڑ
تھگڑی

تھگڑ

گنڈھڑی

گنڈھڑ

ٹھیکری

ٹھیکر
، حاجی،نائی ، یہودی، حلوائی ، قصائی ، فرنگی، بنگالی، بھنگی، کھتری، ہاتھی، امریکی ، افریقی ، ضدی، ہٹھی، موچی، پاولی، ہوڈی، مراثی
کجھ اسمائے مذکر۔ دھوبی ، جوگی، پارسی، درزی، مالی ، بھکاری، چودھری

بگی ، نیلی ، پیلی ، ساوی، غلابی ، عنابی ، چٹی، کوڑی، سچی، کوڑھی، گدڑی، کنجری، کانی، ڈوری، گونگی، لنگڑی، سوڑی، پولھی، گنجی
-اچی، لمبی، مندھری، گیٹی، ہٹھیلی، جھکی ، رتی،
ری ، پھتکاری، تھپکی ،پُنائی، تنبنی، ڈنبھنی، ٹھکائی ، چُھٹی، بدھی ، رادھی، کُہائی ، گھسائی ، لڑائی، لکھائی ، مروڑی، واڈھی، واہی، وہاجی۔
اہالھی، اُپتری سُپتری، اُچھلی ، اڈاری، اُساری ، اگلی ، اگاڑی ، باگھی، بھگی، بھنوالی ، بھروڑی، پدھرائی ، پناکی ، پنجائی ، تاری، پھڑکا
کری ، روٹی ، سیڑھی، استانی ، مچانی ، پٹھانی ، کانی، بھینجی ، ڈاڈی، نانی، ماسی ، مامی ، چاچی، رتی، بگی ، پیلی ، ساوی، کالی ، چھوکری
ی ، بھُتنی، مچھی، مرغابی ، تتلی ، گلہری ، چھپکلی (کرلی)، قمری ، ٹوکری ، پہاڑی، ہتھوڑی، پیالی ، چرخی ، تختی، ٹوپی، مولھی ، چھڑی، ٹو
گھوڑی ، مرغی، بکری،کھوتی، مکڑی، بلی، ہرنی ، مورنی، شیرنی، کبوتری ، ہتھنی ، مینڈک
ی، رنبی، سیناری، کلتری، کلوتری، کانٹی، چونڈھی، کنگھی، سوئی گندوئی ، کہئی، ڈاتری، چمچی، تڈی، پڑچھی ، پچھی ، پیڑھی، رسی ، چرخی
ئی ، ڈیھی، کہاڑی، چھبی، منگری ، پتروٹی، جھجھری، ڈولی، منگھی ، ولٹوئی، ڈوہاوی، مٹی، مندھانی، سنگی، کلھوٹی، منھی ، تھالی تیسی، وہول
گھڑونجی، کاتی، چھری، ڈیوٹی، سو
ازی، دکنی ، عجمی ، مصری کُردی ، ہندی ، بلوچی، سندھی ، حنفی ، سنی، بدوی ، پارسی، یہودی، عیسائی ، صدیقی ، بنارسی، علوی، فاروقی
وچی، جھنگوچی، دہلوی ، پاکستانی، بھارتی، ایرانی ، امریکی ، عراقی، عربی ، فارسی ، مکی ، مدنی، حجازی ، ترکی ، افغانی، ترکستانی ، شیر
شہری ، دیہاتی ، پہاڑی ، دمانی ، دیسی ، وطنی ، ملتانی ، لاہوری ، پشوری (پشاوری)، ڈیروی ، بہاولپوری ، تھلوچی، بر
، کیٹوی ، والی ، چیلکی ، سنگھارپٹی، نتھلی ، چونبھ کلی، ہسی ، دستی ، چوڑی، آرسی، مندری ، جھاری، پٹھی (انگوٹھی) ، نسی، بسنتی ، پٹڑی
کجھ گاہنئیں (زیوریں) دے ناں، جھُمکی
مرگی، ٹھڈی بیماری (تپ دق) ، خرسی ، ابھاسہئی ، اُلٹی ، ہڈکی ، ڈ
کرمچی ، خاکی ، غلابی ، سنہری ، کیسری، جامنی ، انگوری، مہندی
، کنوڑی،مَرھئی، گسنی، مُرچی، بکی (ہتھ دی)، مکڑ ہڈی، دُنی، کھیری ، چھونٹی (گوڈے دی)، نلی (بانہہ یا جنگھ دی ہڈی)، ویٹی، بُکی (گردہ)
پٹی ، تَلی ، تِلی ، کھاڈی، ڈاڑھی، گچی، کُھری ، چَربی، چیچی انگل، کوپری، چھاتی، پپلی، سنگھڑی، ختی، مگی
چاچی ، مامی ، ماسی ، نانی ، ڈاڈی، بھتریجی ، بھنیجی، دھی، پوتری، ڈوہتری ، سالی، سکی ، مترائی ، ڈرانی، بھرجائی، سکوئی
۔ گوبھی ، مولی ، گاجری ، میتھری ، رتی مرچ، بھنڈی ، توری

انبلی ، منگ پھلی، کچری، گھمانوݨی (خوبانی) ، گرِی،گِگمبھیری (مِٹھا)
، ݙکھاری، شِکاری، لَہاری، پاؤلی، پؤلی، کَہالی، موچی، نائی ، قصائی، حلوائی ، دھوٖائی ، مستری، دھوبی، تیلی ، چاکی ،طباخی، مراثی، درزی
کھڈٖاری، پساری (پنساری)، ونگاری، نِلاری، مَداری
کھٹائی ، یاری ، مِٹھائی ، سنجہائی، ننگاہٖی ، ستھرائی، مُٹائی، ٹھکائی ، گھڑائی ، گلاٖئی ، چُٹکی ، گھٹکی ، بھرپّی، بِلکی ، ݙݙھپی، کڑّکی، بھنکی
نیکی ، بندگی، زندگی، صفائی ، گرمی ، سردی ، نرمی ، جوانی ، دیوانی، ودھیکی ، چُھرکی ، تکھائی ،
روضہ ، نسخہ، صفحہ، خواجہ ، سقہ، بندہ ، صوفہ ، پیشہ، تیشہ، خرچہ، چرخہ ، حُقّہ ، ݙبہ، دورہ، طرہ، شیشہ ، آئینہ، پیمانہ، لمحہ، وقفہ، پیسہ
وَحشی، وِسامور، وِسواسی، وِسواسݨ، وِگاڑو، وِللا، وِللی ، ونجاؤ، وِہرا، وَہمی ،وَیری، وَیرݨ، وَہمݨ ، وہری، وَیل ݙاچی، وݙھی خور، وݙھی ݙیو
وݙواتا، وݙکوتھا، وݙکناں، وݙپیرا، واچھل، واشِل،
وݙو، دھاٖنو، پھاپو، سَدو، پارو، ماندھو، صابو، راٖنو، جامو، کالو
جَوائݨ، مُوذِݨ، بِھکارݨ، اُلھادݨ، پُجارݨ، جوگݨ، شِکارݨ، سوالِݨ، یہودِݨ، مالکِݨ، قیدِݨ، بنجارݨ ، نائݨ، چودھرائݨ، بنگالݨ، پڑوسݨ، درزݨ، پارسݨ
ما لِݨ، دھوبݨ ، بھنگِݨ، پارسِݨ، فرنگِݨ، مراثِݨ، ملودِݨ، بھونڈِݨ، بُلھݨ، قصائݨ، ݙیݨ،سَئیݨ، اَرئیݨ، حَلوائݨ، بٹھارݨ،
اڈٖاݨ ، بِہاݨ، بِدھاݨ، پُراݨ، ݙندان، کھَنبھاݨ، مَندھاݨ، وݙاݨ، ٹٖباݨ، جھنگاݨ
ِݨ، چلکِݨ، پَٹِݨ، پحلِݨ ،پھتکِݨ ، پھَیِݨ، تِرمِݨ ، ٹٖبِݨ، حُلِݨ، چُبھݨ، چُھلکِݨ، ݙلِݨ، رنگِݨ، سڑِݨ، شوکِݨ، کِھُرکِݨ، گھُرکِݨ،کھمِݨ، شَرکِݨ، وَلِݨ،وَہِݨ، ہوکِݨ
آݙِھݨ، اُبلِݨ، اَٹکِݨ، ہَٹکِݨ ، اِڑکِݨ، بلکِݨ، چَھلکِݨ، ݙِھلکِݨ، گھلکِݨ، ہُلکِݨ، تِلک
جومارݨ ، پیوݨ، مریجݨ،بِدھݨ ، ݙیکھݨ، رووݨ، رویجݨ
نچوڑا

نچوڑݨ

نِکھڑت،ِ نکھڑا

نِکھڑݨ

نِکال ، نِکالی

نِکلݨ

نَچا، نچݨی

نَچݨ

نَبیڑا

نَبیڑݨ

ناچبا

نَچاوݨ
نِتارا

نِتارݨ

نِبھا

نبھاوݨ

نَپیڑا

نپڑیجݨ
نوا

نِوانوں

نِمرت

نِمرنْ

نَپا

نَپاونْ
نِمرا

نِمراونْ

نِکھرت

نِکھرنْ

نِبھنْی ,نبھت

نِبھنْ

نَلی ، نَالا، نانہہ، نِب، نَٹ، نَڑی ڈِبّا، َنعل، نَیل، نِغارہ، نک چونا،نکَیل ، نَہیرنْ، نِہاندرو، نِہترا، نیچا۔
نت، نَحق، نَدان، نَدیدہ ، نِدھورا، نِراس، نَراض، نِقابل، نکِنگلا، نگوسانواں، نَلائق، ِنمراس، نِمراش، نوموز، نَہوند، نِبھایا، نِبھائی ، نِبھاگا، نِبھاگی۔
نکمّاں، نکٹّھو، نِپھّر، ندِھنْکا، نِکارہ، َنمرد، نکھند، نَحیف، نَچ
مَکھنْ مکھنْاں، مجَھنگ، مَٹھو , مَٹھا مٹِھڑا، منٹھار، مِہربان ، مُہذّب، مہاندرا، موہری ، مومن
ُنگری، مِسواک، مشعل، مشین، منجن، مُنگلی ، منجھولا، مندھنْا، مولھا، مہار، مُہر،مَہرا، میخ، مُٹھا، منگھی، منگھا، مندھانْی ، مُگتر، مٹی، مٹورا۔
مالھا، مانجا، مُوڑھا، مُرلی ، مرتبان ، مسوائی، مُنگر، مہ
سا، میسنْی، میسنْا، مُتھر، مُتھاح، مَٹھا، مٹھی، مِٹھڑا، مِٹھڑی، مچھُل ،مَٹھوڑ، موالی ،مونجھا، منتارا، منتاری، مکرا، مکریل، مَرکھی، مَریل، مریلا
ا، مُڈّر، مریلا، مستوڑ، مشتبہ، مشکا مشکی، مغرور، مکار، ملام،مَم، مَنچر، مَندیل، منحہر، مندھرا، مندھری، مندھول، مندھوڑ، موذی، مِیکنْ ، میہ
ماندھو، ماد، مریلہ ، مپا، مُترڈ، مُچھ ڈنڈ

، مَری، موڑ، مروڑ، مروڑا مروڑی، مُرکار، مُرک، مُسک، مُسکار، مکیوا، مانج، مُکلاوا، ، مکَر ،مَیل، مِیلہ ،مَندھ، مَنڈ ، مَنوت، منیوا، منگنْی۔
مار، ماپ، ماٹھ، مانْ ، مُتراکا، مَچ، مَچھراند، موچھ، مَچلپ، موت
َترپ،مُخبری ،مُدت، مختیاری ، مخول، مَروڑ، مزدوری، مِنت ،مَن منیج، مُنجھاری ،مُونجھ، مندھنْی، مواتی، مونگھر، موکھ، مِہاگ، مہورت، مَپھ
ماتم، مارکُٹ، ماردھاڑ، مانْ ، مانگ، مانج، مَپ، مَت، مہ
اڈم، یبھوم ، شرم ، دھرم ، بھرم، کرم، ستم، ظلم، نم، مدھم، مسلم، مبہم، مقام، ماتم، اودھم گودھم، گھم، رشوم (رسم) ، دھوم۔

سہم

سہَمنْ

دَھم

دَھمنْ

گھُم

گھمنْ
کِھم

کِھمَنْ

جھوم

جھومݨ

سَم

سَمݨ

چُم

چُمݨ

تِرم

تِرمݨ

، لپور لُپرائی، لوپر، لپیٹ، لَتاڑ ، لوٹی، لَٹک ، لٹکا، لسک، لُسکار، لَسکار، لشکارا، لُک،لکݨ، لُکݨ، لِکھائی، لُگ، لمبائی ، لانگھا، لوند، لیٹ، لُجھ
، لذت، لڑاند، لعنت، لگوند،للھّر، لَمبݨ، لمباݨ ،لُندھپ، لوڈ، لوڈا،لُوس، لَوش، لَونڈک، لوبھ، لیاقت،لیپ، لیس، لِیک ، لَبھت، لچھݨ، لیکھ، لِکھت، لاپ
لاٹ، لاح، لاڈ، لاغری، لاگ، لاگت، لالچ، لالی، لاہی، لباڑ، لبکار، لتڑ، لح، لُچ، لُچپ، لچک، لچئی
َلال، پَلال، دَھمال، گڑدَھال، کُڑوّل، پَکھال، اَختِل، اَڑتَل، بچھَل، اَٹکل،تَوکل،تَھڑتھل، ٹھاڈھل، جنجال، جھال، جھول، حلول ، چھول ، خیچل، دروہل
بَگوِل ، چٹوِل ، رَتوِل، ساوِل، سوجَھل، کویل، اویل، سویل ، اُلیل، الول، م
اُچھال ، اُگال ،بھال، پَجال، سَنبھال ،نِگال، پَڑتال، انگھال، اُدھال، چال، گال۔
بھَل ، بَل پُل، جَھل، جُھل، سَلھ، سِلھ، کَل، کِل، نَل، ہَل، رَحل، تھال ، بوتل، کجَل، حال، دول، ڈول، بگِل، پَتل، ترانگل، ترپال، تُول ، بَٹھل
ا، لَنڈر‘لَنڈور، لنگڑا، لنگڑی، لُوت، لُوطی، لُوترا لَوتا،لَوشی ،لَوشن، لوفر، لوگڑ، لُولھا،لُولھی، لومبڑ، لوبھی، لوبھݨ، لوتھ، لیچڑ،لِتھڑ، لُڈھ، لاغر
لچ لچا، لچی، لچر لچری ، لڑاکا لڑاکو، لڑاکی، لعین، لعنتی، لفنگا، لُگا، لُگی، للا، للی، للھری، لم چچڑ، لمِل، لُنب، لُندھا، لُندھی ،لَندھوڑ، لُنڈپچھ
ڑ کناں، لا تکا، لالچی، لامواسا، لئی لگ، لوسی ،لباسی، لِباسݨ ، لباری ، لپاٹی لپاٹا، لپوشک لپوشا لپوشی، لپوشݨ، لٹو، لٹیرا، لٹور لٹوری،لغور ،
بری منفی صفت کیتے ورتئیے ویندن تے او ل کن شروع تھیندن۔ لا پرواہ ، لا پ
لاٹوں ، لاکڑا، لاگ، لانہہ، لٹھ، لڑکا، لغام، لغر،لِفٹ، لوٹا

ڈھونگریل

ڈھونگر

ہوڈل

ہوڈ

چُکِل

چک

کُبڑیل

کبڑ

ڈراکل

ڈر

تھوڈل

تھوڈٖ

ٹکریل

ٹکر

چَکّلِ، چکیل

چَک

ناسِل

ناس
اڑانگل

اڑانگا

ہَڈِّل

ہَڈّ

منديل

منده
كوڑيل

كاوڑ

ڈندالہ ڈندِل

ڈند

غصيل

غُصہ
ڈِھڈّھِل

ڈِھڈّھ

مِٹھَل

مِٹھا

تِرکّل

پلئيل

پَلنْ

اُکھڑیُل

اُکھڑنْ

بَچیئل

بَچنْ
اُبھریُل

اُبھرنْ

اِٹکیُل

اِٹکنْ

اَڑیُل

اَڑنْ
بَسکیُل

بَسکنْ

آپھریُل

آپھرنْ

اَپڑیُل

اَپڑنْ
رِنگ

رِنگنْ

ڈِھنگ

ڈھنگنْ

منگ

منگنْ
ڈِنگ

ڈِنگنْ

ڈِنگ

ڈِنگݨ

رِینگ

رِینگݨ
وَنگ

وَنگݨ

ہونگ

ہُونگݨ

ٹنگ

ٹنگݨ

#مصدری مادہ ، امر

مصدر

#مصدری مادہ ، امر

مصدر

#مصدری مادہ ، امر

مصدر
-10 کجھ سرائیکی مصادر دے مصدری مادے ، امر جنھیں دے اخیرءِ چ ''گ'' آندے ۔
گلھڑ

گلھ

لپڑ

لپ

دَنڑ

دَن
چھلڑ
[illegible]-
جَھگ

جَھگݨ

ٹھگ

ٹھگݨ

ٹُرگ

ٹُرگݨ

ڈھڳ

ڈھڳنْ

تیاڳ

تیاڳنْ

چُڳ

چُڳنْ

لڳ

لڳنْ

بھوڳ

بھوڳنْ

جاڳ

جاڳنْ

ڈِھڳ

ڈِھڳنْ

ہُڳ

ہُڳنْ

تَڳ

تڳنْ
جھاڳ

جھاڳنْ

سَنڳ

سَنڳنْ

پُڳ

پُڳنْ
ڳا ڳرھا، ڳالھا، ڳالھی، ڳالو، ڳٹکا، ڳٹکی، ڳِچل، ڳڑدھالی ، ڳلھوڑ، ڳلوڑ، ڳنڈھیلا، ڳوڈِل، ڳہیڑی، ڳٹھ مٹھڑا، ڳپی ،ڳپاٹی ،ڳپاڑی ، ڳویڑھی ، ڳویڑھا۔
ڳاٹکا، ڳٹکا، ڳاڈا، ڳاڈی، ڳاری، ڳانا، ڳانی، ڳت، ڳٹی، ڳڈی، ڳیٹا، ڳیٹی
مُساڳ، راڳ، مِہاڳ، ساڳ، کاڳ، واڳ، لاڳ، بڳ، بھَڳ،َجھڳ، پڳ،جَڳ، ٹِڳ، پھوڳ، دَڳ، وَڳ، واڳ، لِنڳ، ہنڳ، جوڳ، چوڳ، لوڳ
بُھڳ، بَھڳ، ٹھڳ، مُڳ، نبھاڳ،بھوڳ، روڳ، سبھاڳ ، آڳ، مُلاڳ، سُہاڳ، مِہاڳ، نِبھاڳ، بھاڳ، لاڳ، جاڳ
بَھنگ، وَنگ، جھنگ، رَنگ، چِھنگ، لِنگ، ہنگ، پَلنگ، چِنگ، تنگ، ڈھنگ، کَنگ۔
چُتڑنگ، اَدھڑنگ، اَبھڑنگ، ملنگ، اَٹنگ، مَجھنگ، شڑنگ، چڑنگ، غُڑنگ

شارک، گجَک، جھِنْک، بَھڑَک ، عینک ،چیلک، سانوک، دَھمک، کُھرک، وَٹک، بیٹھک، ہنْک، لونْک، بڑک، پَلک، ڈِالک، سَڑک،وَدھک، کَنِک ،
چَھمک، پالک، گولک
کُراہ، کُسنگ، کوہترا ، کُڈھنواں
بُڑک، بڑدَھک، کھُٹک، رَتک، لُوسک، کالک، کھُرک، پھُٹک، ڈِبھک، خُندک، پھُوتک، چٹک، ڈِالک ، تِڑک
مسک ، مہک، ہمک، ہنْک، دھنک، ہسک، ڈِسک، چٹک، چرک ، چھنْک، جھنْک، بُڑک ، ہرک، بِھرک، اُڑک، اَجھک، اُٹھک، اُٹک، اَٹک
چڑاک

چڑنْ

چھٹاک

چُھٹنْ

رہاک

رَہنْ

تراک

ترَنْ

پھٹاک

پھٹنْ

تڑاک

تِڑکنْ

پھراک

پھرنْ

بھڑاک

بھڑنْ

پھنڈاک

پھُنڈنْ

بھجاک

بھجنْ

نساک

نَسنْ

پناک

پنڑْ
کڈٖاک

کڈٖنْ

چٹاک

چَٹنْ

اڈٖراک

اُڈٖرنْ
غرک

غُرکنْ

ٹھمک

ٹھمکنْ

پَھتِک

پھتکنْ

بھونک

بھونکنْ
ہِسک

ہِسکنْ

مرک

مُرکنْ

سیک

سیکنْ

ٹوک

ٹوکنْ
پَھڑک

پھڑکنْ

پُھوک

پھوکنْ

سِک

سِکنْ

تِڑک

تِڑکنْ

بِوک

بِوکنْ

پھرک

پھرکنْ

بڑک

بِڑکنْ

بڑک

بِڑکنْ

لُسک

لسکنْ

بِرک

بِرکنْ

بِھرک

بھرکنْ

اِڑک

اڑکنْ

تِھڑک

تِھڑکنْ

اُڑک

اُڑکنْ

اَجھک

اجھکنْ

اَٹک

اٹکنْ

ساٹوں ، ساڑا، سازشی، ساکڑ، سامری، سانہ، سانہاں ، سنڈھا، سبارْ، سپڑا، سٹ حرام دی، سٹے باز، سرکش، سرپھری
، اَرداس، اَسیس، افسوس، اِکاس، اَکلیس، بکواس، پیاس، بَھڑاس، حبڑاس، حَسّاس، حَواس، ستیاناس، ہراس، سنیاس، ہبلاس ،وِسواس، مِٹھاس،لِباس
آس، آلس، اِحساس

ہس

ہسنْ

وس

وسنْ

نس

نسنْ
گس

گسنْ

کھس

کھسَنْ

ڳس

ڳسنْ
رس

رسنْ

ڈہس

ڈہسنْ

دس

دسنْ
پھس

پھسنْ

پس

پسنْ

ہس

کھاندِنس

ٻالا، رٻالی، ردی ، رذیل، رڈِل، رِنڈل، رنوں، رنوڑ، رقیب، رِکل، رگِل ، رلوڑ، رنڈی، رنگیلا، روڈٖا، روڈٖی، روگی، رول ،ریچک ماریں کھویندے راڑا، راڑی، راشی ، راخس، رٻ

تلوار، پھوہار، نِسوار، پَرکار، جُوار، عار، آر، مار، کار، تکرار، اِنتظار، غار، پَہار، ٻار، پار، اُروار، تار، ٹار، جھار، نِسار، اُلار، کٹار، وِنگار ، پیار، وَپار، معیار، اُدھار، بار، ویار، ہزار، ہَسوار، ہتھیار، کم کار، اِصرار، اِتبار، اِقرار، اِنکار، سَردار، وار، حار، یار، خار، بیمار، شہر، قہر، شُکار ،پزار ،ہَزار

ہشکار

ہُشکارنْ

دھتکار

دھتکارنْ

ٻچکار

ٻچکارنْ

گھمکار

گھمکارنْ،گھمکنْ!

پھٹکار

پھٹکارنْ

گھٻکار

گھٻکنْ

مسکار

مُسکنْ

ڈٖسکار

ڈٖسکنْ

پُکار

پُکارنْ

اُبھار

اُبهرنْ

اٹکار

اٹکنْ

گهرکار

گهرکنْ
گجکار

گجنْ

غُرکار

غُرکنْ

چکار

چیکنْ

کڑکار

کڑکنْ

پهتکار

پهتکنْ

ہکار

ہوکنْ

شکار

شوکنْ

وحکار

وحنْ

کهڑکار

کهڑکنْ

، ڈانونْ، ڈناں ، ڈنی، ڈنڈکا، ڈنڈیہ ڈندکی، ڈپھا، ڈپھی، ڈندونْ، ڈندالی، ڈندیلی ، ڈولا، ڈولی، ڈولڑا، ڈولڑی، ڈہاوی، ڈہاوا، ڈوئی ، ڈیوا، ڈیوٹا، ڈیوٹی
ڈاتری، ڈاکا، ڈاکن، ڈانگ، ڈنگوری
، ڈوروں بھوروں ، ڈوم، ڈومنی ، ڈورا، ڈوری، ڈوہاری، ڈوہاگن، ڈبلا، ڈہیلا، ڈبھک، ڈپھیل، ڈتھا، ڈتھی، ڈڈھپی، ڈکھارا، ڈکھاری، ڈکھڑہٹ، ڈکھی
اِکنْ ، ڈالا، ڈالو، ڈندو، ڈندل ڈینْ، ڈیاگدرا، ڈڈو، ڈڈوں ، ڈڈل، ڈڈیل، ڈراکل، ڈروپڈو، ڈلھ، ڈمریل ، ڈمرا، ڈنڈی مار، ڈنگا، ڈنگی ، ڈنگیلا، ڈوالہ، ڈوٹا
ڈٹل ، ڈاٹو، ڈٹ باز، ڈٹکی ، ڈٹی ، ڈٹیل ،ڈٹوری ڈاڑھو، ڈ
۔ ڈاں ، ڈبا، ڈبی، ڈِبری ، ڈبیکنْ ، ڈوڈنْ، ڈوڈنی، ڈوئنگا، ڈھینڈھا، ڈھارا، ڈھاری، ڈھال ،ڈھولنْ، ڈھکنْ، ڈھکنی، ڈھنگا، ڈھولک ، ڈھولکی ، ڈھیرا۔
تھیندن
ڈینی ، ڈھاڈھی ، ڈھٹا، ڈھٹی ، ڈِڈھ مٹھا، ڈِڈھ مٹھی ، ڈِڈھو، ڈِڈھل، ڈھرکپا، ڈھرکپی، ڈھکوڑا، ڈھکوڑی، ڈھکیئل، ڈِھلر، ڈِھلوڑ، ڈاؤں، ڈیڈا، ڈیٹھہ
ڈاکوہ ڈکیٹ، ڈبکا، ڈڈا، ڈڈا، ڈرگا، ڈرگی، ڈگی، ڈھگا، ڈیٹاں
داسا، ڈِگا، داڑھی ، دُھرا، داپھا، داپھی ، دوریہ دنوری، دُرا، دھرمٹ، دستار، دستانہ، دستی ، دف، دول، دمامہ، دیگڑا، دیگڑی ، دیگ، دِلا۔
دابڑہ، دابڑی
دادلا، دانا، دُر، دردرنجاٹاں، درویش، دریادل ، دعاگو، دلاور، دلبر، دلچسپ، دُل دریا، دلدار، دلربا، دلیر، دوراندیش، دیرپا، دور بین ، دیندار۔

ی، دوغلا، دھوکے باز، دیگڑا، دھاڑل، دھچر، دھڈہ دھڈا، دھرتالی، دھرتالِنْ، دھڑے وات، دھکڑہ دھکڑا، دُھوتا، دُھوتل، دؤس، دروہی، دُن مُک۔
دابڑبلا، داغیہ داغل، دانگی، دانگل، دروہا، دریٹھ،دڑل، دغابازہ دغل، دکڑ ، دگل، دلاہ دلو، دل بھس، دماغل، دنبل، دودا، دودل، دوزخ
باز، خائن، خبطی ، خپڑ، خُت،خُتل ، خُندکی ، خَچکل، خَچرا،خَرتل، خَرچل ، خرشیل، خرمغزا، خشکیل، ختمی، خُشکا، خونی، خوشامدی، خوننْ
خار
قت، جئیں ویلھے ، جڈاں ، جینویں ، جیجھاں ، جیجھئیں، جہوجھئیں، جہو جھئیاں ، جیویں جیویں، جتھاں جتھاں ، جڈاں جڈاں ، جیجھاں جیجھاں ۔
جتھاں ، جتھوں ، جنتے ، جیڈے ، جئیں پاسے ، جئیں و
چورس، چوچنڈا ، چوبارہ، چوکاندھا، چودھار، چوطرفہ، چوسڑکا، چوراہا، چوہٹہ، چو مکھی، چوکور، چوکنا، چوگنا، چوہتھا، چواگھا، چوگردو
بِا، چچڑ، چترا، چترماں، چُتڑنگ، چُتڑیل، چتکبرا، چٹپیرا، چَندرا، چوڑچپٹ، چَم چچڑ، چڑاک، چُرتی ، چَرسی ، چڑی مار، چغل خور (چغلیٹ)
چبل ، چنگو، چوٹھو، چچرا، چکھڑا، چیتھڑ، چپاہ چپل، چوڑھا، چُچا، چَنبڑ، چاکر، چالباز، چَپڑ، چ
طوفانی
روشن
گرم
سرد
مہنگی
خوش
آسان
چھوٹے

سرخ ۔ نیلا ۔ خوبصورت ۔ شریف ۔ چالاک ۔
خوُشی
گیا
کیَسے
-6شُکریہ
اکٹی (زیادہ قے)
