# Supplementary material for: Mixed Script Identification Using Automated DNN Hyperparameter Optimization
Source: Comput Intell Neurosci. 2021 Dec 10;2021:8415333. doi: 10.1155/2021/8415333 (PMC8683192; doi:10.1155/2021/8415333)
Supplement: Supplementary Materials — (e.g., datasets or results outcomes in the form of graphs) from different stages are provided with the manuscript. The graphs including system training, validation, and the testing outcome of all RNN variants are included in Supplementary Materials. [file 8415333.f1.zip › 8415333.f1/urdu.pdf]

سیدھا
گشتی مراسلہ
شکلیں
چوڑا
باریک
موٹا حصّہ
لمبا
چھوٹا
مختصر
پتلا
لمبا
گہرا
بڑا
حجم
زرد
سفید
سرخ
جامنی
نارنگی
سبز
خاکستری
کتھئی
نیلا
کالا
square cheokeor - چوکور
triangular meselesey shekel kai - مثلثی شکل کے
tastes dha'iqai - ذائقے
bitter keṛeoa - کڑوا
fresh tazeh - تازہ
salty nemekeyn - نمکین
sour teresh - ترش
spicy meṣaleḥeh dare/cheṭ peṭa - مصالحہ دار/چٹ پٹا
sweet meyṭeha - میٹھا
qualities kheobeyan - خوبیاں
bad bera - برا
clean ṣaf - صاف
dark anedeheyra - اندھیرا
difficult meshekel - مشکل
dirty meyla - میلا
dry seokeha - سوکھا
easy asan - آسان
empty khaley - خالی
expensive mehenegua - مہنگا
fast teyz reo - تیز رو
foreign gheyr melek - غیر ملک
full lebereyz - لبریز
good acheha - اچھا
hard sekhet - سخت
heavy beharey - بھاری
inexpensive kem qeymet - کم قیمت
light reosheney - روشنی
local meqamey - مقامی
new neya - نیا
noisy per sheor - پر شور
old 'emer reseydeh - عمر رسیدہ

powerful ta qeteor - طا قتور
quiet khameosh - خاموش
correct dereset - درست
slow aheseteh - آہستہ
soft nerem - نرم
very zeyadeh - زیادہ
weak kemezeor - کمزور
wet gueyla - گیلا
wrong ghelet - غلط
young jeoan - جوان
quantities meqedareyn - مقداریں
few chened - چند
little cheheoṭa - چھوٹا
many behet - بہت
much keseyr - کثیر
part ḥeṣeh - حصّہ
some kecheh - کچھ
a few chened ayk - چند ایک
whole temam - تمام
le(guezera heoa) - کل(گزرا ہوا)
today aj - آج
tomorrow kele(anai oala) - کل(آنے والا)
now abehey - ابھی
then teb - تب
later be'ed meyn - بعد میں
tonight aj rat - آج رات
right now abehey abehey - ابھی ابھی
last night guezerey heo'iy rat - گزری ہوئی رات
this morning aj ṣebeḥ - آج صبح
next week aguelai hefetai - اگلے ہفتے
already pehelai sai - پہلے سے
recently abehey - ابھی
lately kecheh den heo'iai - کچھ دن ہوئے
soon 'eneqereyb - عنقریب
immediately feora - فوراً
still abehey tek - ابھی تک
yet abehey tek - ابھی تک
ago guezesheteh - گزشتہ
adverbs of place mete'eleq afe'al (jegueh ka) - متعلق افعال (جگہ کا)
here yhan - یہاں
there adeher - ادھر
over there ohan per - وہاں پر
everywhere her jegueh - ہر جگہ
anywhere kesey jegueh sai - کسی جگہ سے
nowhere keheyn neheyn - کہیں نہیں
home gueher - گھر
away deor - دور
out baher - باہر
adverbs of manner mete'eleq afe'al (adeb ka) - متعلق افعال (ادب کا)
very nehayte/zeyadeh - نہایت/زیادہ
quite balekele/temame/seraser - بالکل/تمام/سراسر
pretty kheobeṣeorete/ḥeseyn - خوبصورت/حسین
really aṣel meyn - اصل میں
fast teyz reo - تیز رو
well acheha - اچھا

hard sekhet - سخت
quickly teyzey sai - تیزی سے
slowly aheseteguey sai - آہستگی سے
carefully meḥetat anedaz meyn - محتاط انداز میں
hardly bemeshekel - بمشکل
barely bemeshekel - بمشکل
mostly beysheter - بیشتر
almost teqereyba - تقریباً
absolutely qete'a - قطعاً
together akeheṭai - اکھٹے
alone akeylai - اکیلے
adverbs of frequency mete'eleq afe'al te'eded ka - متعلق افعال تعدّد کا
always hemeysheh - ہمیشہ
frequently akeser - اکثر
usually 'emeoma - عموماً
sometimes kebehey kebehey - کبھی کبھی
occasionally kebehey kebehar heonai oala - کبھی کبھار ہونے والا
seldom shaz o nader - شاذ و نادر
rarely shaz o nader - شاذ و نادر
never kebehey neheyn - کبھی نہیں
