# Supplementary material for: Mixed Script Identification Using Automated DNN Hyperparameter Optimization
Source: Comput Intell Neurosci. 2021 Dec 10;2021:8415333. doi: 10.1155/2021/8415333 (PMC8683192; doi:10.1155/2021/8415333)
Supplement: Supplementary Materials — (e.g., datasets or results outcomes in the form of graphs) from different stages are provided with the manuscript. The graphs including system training, validation, and the testing outcome of all RNN variants are included in Supplementary Materials. [file 8415333.f1.zip › 8415333.f1/words2.pdf]

#NG نقشے < > #NG # پاکستانی < > ---- < > پاکستانی < > #NG # دا < > Sad #P # > پاکستانی < > زبانیں <
پاکستانی > < #NG # گجراتی <N# بلوچی > < #P # سندھی <N# سرائیکی <N# وچ > < #
< > < شامل > < فیض > < تعلیم > < الصّوت > < دیاں > < سئیں > < کریندے > < سروے > < مفرید > < بیٹھوں
< ایچ > < زبانیں > < نقشہ > < ھَن > < ھِن > < لیکن > < اے > < وڈے > < برٹش > < انڈیا > < سونہاں > <
ڈٹا > < تے > < فرید > < سر > < اپ > < ہندستان > < بشمول > < تعاون > < چھاں > < حکومت > < لنگویچ > <
محقق > < ھے > < نقشے > < پی > < بڑایا > < وسیم > < کائنی > < تقریباً > < دی > < بہوں > < وی > < اساں
< کوں > < گِئے > < اعلیٰ > < دا > < گجراتی > < لینگویچ > < وچ > < صرف > < ---- > < ڈی > < برکت >
ھی > < تمام > < ایندے > < روضے > < کون > < جو > < نال > < منظوری > < کیتن > < آلے > < سکدے۔ >
< سرائیکی > < ڈیکھایا > < دے > < ؟ > < مطابق > < سال > < بلوچی > < ء Sa> #P # < سنہ > < انہاں > < یافتہ
< ناں > < جنھاں > < شاعری > < > < بیٹھوں > < کریندے > < گالھ > < دی > < صدیقی > < وسیم > < آلے > < لیکن >
لکیراں > < دیاں > < شاعری > < تے > < رہ > < تلے > < چھاں > < دی > < روضے > < دے > < سئیں > < فرید > < اساں
< اج > < جو > < ھے > < ھی > < برکت > < تے > < فیض > < دا > < شاعری > < دی > < سئیں > < خواجہ > < اے > <
؟ > < کائنی > < سونہاں > < کون > < دا > < شاعری > < دی > < انہاں > < تے > < سئیں > < مفرید > < وچ > < سروے > <
لنگویچ > < ایں > < گِئے > < ڈٹا > < ناں > < دا > < انڈیا > < آف > < سروے > < لینگویچ > < کوں > < تحقیق > < ایں > < ----
< کیتن > < صرف > < تے > < تحقیق > < دی > < زبانیں > < سال > < 1887تقردھ زبانیں > < وچ > < ہندستان > < متحدہ > <
جنھاں > < ھَن > < محقق > < وڈے > < بہوں > < ھَن > < وی > < ڈی > < ایچ > < پی > < نال > < نال > < دے > < یافتہ > <
تعلیم > < اعلیٰ > < اپ > < ھَن > < گریرسن > < سر > < منظوری > < دی > < نقشے > < ایں > < سکدے۔ > < ونج > < ڈیکھایا
< کوں > < زبانیں > < پاکستانی > < تمام > < وچ > < ایندے > < سرائیکی > < بشمول > < وچ > < علاقے > < ایں > < مطابق
< دے > < نقشے > < گِئے > < بڑایا > < نال > < تعاون > < دے > < حکومت > < برٹش > < وچ > < 30ء > < بیاَی > < مل
شا > < زبانیں > < گجراتی > < بلوچی > < سندھی > < ایں > < پاکستانی > < صدیقی > < 1887 > < علاقہ > < خواجہ >
متحدہ > < اج > < گریرسن > < تحقیق > < گالھ > < ونج > < لکیراں > < ائے > < رہ > < تلے > < جلی > <
آف > < > < بیٹھوں > < کریندے > < گالھ > < دی > < صدیقی > < وسیم > < آلے > < لیکن > < لکیراں > < دیاں > < شاعری >
< تے > < رہ > < تلے > < چھاں > < دی > < روضے > < دے > < سئیں > < فرید > < اساں > < اج > < جو > < ھے > < ھی
< برکت > < تے > < فیض > < دا > < شاعری > < دی > < سئیں > < خواجہ > < اے > < ؟ > < کائنی > < سونہاں > < کون
< دا > < شاعری > < دی > < انہاں > < تے > < سئیں > < مفرید > < وچ > < سروے > < لنگویچ > < ایں > < گِئے > < ڈٹا
ناں > < دا > < انڈیا > < آف > < سروے > < لینگویچ > < کوں > < تحقیق > < ایں > < ---- > < کیتن > < صرف > < تے > <
تحقیق > < دی > < زبانیں > < سال > < 30 > < تقریبے ِن > < وچ > < ہندستان > < متحدہ > < جنھاں > < ھَن > < محقق > < ے
وڈ > < بہوں > < ھَن > < وی > < ڈی > < ایچ > < پی > < نال > < نال > < دے > < یافتہ > < تعلیم > < اعلیٰ > < اپ > < ھَن
گریرسن > < سر > < منظوری > < دی > < نقشے > < ایں > < سکدے۔ > < ونج > < ڈیکھایا > < کوں > < زبانیں > < ستانی
پاک > < تمام > < وچ > < ایندے > < سرائیکی > < بشمول > < وچ > < علاقے > < ایں > < مطابق > < دے > < نقشے > < گِئے
بڑایا > < نال > < تعاون > < دے > < حکومت > < برٹش > < وچ > < 30ء > < ھا > < شامل > < زبانیں > < گجراتی > <
بلوچی > < سندھی > < سرائیکی > < وچ > < نقشے > < پاکستانی > < ---- > < سرائیکی > < دا > < زبانیں > < پاکستانی > < > <
بیٹھوں > < کریندے > < گالھ > < دی > < صدیقی > < وسیم > < آلے > < لیکن > < لکیراں > < دیاں > < شاعری > < تے > < ہ
ر > < تلے > < چھاں > < دی > < روضے > < دے > < سئیں > < فرید > < اساں > < اج > < جو > < ھے > < ھی > < برکت
تے > < فیض > < دا > < شاعری > < دی > < سئیں > < خواجہ > < اے > < ؟ > < کائنی > < سونہاں > < کون > < دا > <
شاعری > < دی > < انہاں > < تے > < سئیں > < مفرید > < وچ > < سروے > < لنگویچ > < ایں > < گِئے > < ڈٹا > < ناں > < ا
د > < انڈیا > < آف > < سروے > < لینگویچ > < کوں > < تحقیق > < ایں > < ---- > < کیتن > < صرف > < تے > < تحقیق > <
دی > < زبانیں > < سال > < 1887 > < تقریبإن > < وچ > < ہندستان > < متحدہ > < جنھاں > < ھَن > < محقق > < وڈے > < بہوں
< ھَن > < وی > < ڈی > < ایچ > < پی > < نال > < نال > < دے > < یافتہ > < تعلیم > < اعلیٰ > < اپ > < ھَن > < گریرسن
< سر > < منظوری > < دی > < نقشے > < ایں > < سکدے۔ > < ونج > < ڈیکھایا > < کوں > < زبانیں > < پاکستانی > < تمام >
< وچ > < ایندے > < سرائیکی > < بشمول > < وچ > < علاقے > < ایں > < مطابق > < دے > < نقشے > < گِئے > < بڑایا > <
نال > < تعاون > < دے > < حکومت > < برٹش > < وچ > < 30ء > < ھَا > < شامل > < زبانیں > < گجراتی > < بلوچی > < ی
سندھ > < سرائیکی > < وچ > < نقشے > < پاکستانی > < ---- > < وچ > < دا > < زبانیں > < پاکستانی > < > < بیٹھوں > < ریندے
ک > < گالھ > < دی > < صدیقی > < وسیم > < آلے > < لیکن > < لکیراں > < دیاں > < شاعری > < تے > < رہ > < تلے > < اں
چھ > < دی > < روضے > < دے > < سئیں > < فرید > < اساں > < اج > < جو > < ھے > < ھی > < برکت > < تے > < فیض
< دا > < شاعری > < دی > < سئیں > < خواجہ > < اے > < ؟ > < کائنی > < سونہاں > < کون > < دا > < شاعری > < دی >
< انہاں > < تے > < سئیں > < مفرید > < وچ > < سروے > < لنگویچ > < ایں > < گِئے > < ڈٹا > < ناں > < دا > < انڈیا > < ف
آ > < سروے > < لینگویچ > < کوں > < تحقیق > < ایں > < ---- > < کیتن > < صرف > < تے > < تحقیق > < دی > < زبانیں > <
سال > < 1887 > < تقریبإن > < وچ > < ہندستان > < متحدہ > < جنھاں > < ھَن > < محقق > < وڈے > < بہوں > < ھَن > < وی >
ڈی > < ایچ > < پی > < نال > < نال > < دے > < یافتہ > < تعلیم > < اعلیٰ > < اپ > < ھَن > < گریرسن > < سر > < نظوری
م > < دی > < نقشے > < ایں > < سکدے۔ > < ونج > < ڈیکھایا > < کوں > < زبانیں > < پاکستانی > < تمام > < وچ > < ایندے >
سرائیکی > < بشمول > < وچ > < علاقے > < ایں > < مطابق > < دے > < نقشے > < گِئے > < بڑایا > < نال > < تعاون > <
دے > < حکومت > < برٹش > < وچ > < 30ء > < ھِن > < شامل > < زبانیں > < گجراتی > < بلوچی > < سندھی > < سرائیکی
وچ > < نقشے > < پاکستانی > < ---- > < نقشے > < دا > < زبانیں > < پاکستانی > < > < بیٹھوں > < کریندے > < گالھ > <
دی > < صدیقی > < وسیم > < آلے > < لیکن > < لکیراں > < دیاں > < شاعری > < تے > < رہ > < تلے > < چھاں > < دی > <

< روضے > < دے > < سئیں > < فرید > < اساں > < اج > < جو > < ھے > < ھی > < برکت > < تے > < فیض > < دا > اعری
< شد < دی > < سئیں > < خواجہ > < اے > < ؟ > < کائنی > < سونہاں > < کون > < دا > < شاعری > < دی > < انہاں > < تے
< سئیں > < مفرید > < وچ > < سروے > < لنگویچ > < ایں > < ڳئے > < ڈتا > < ناں > < دا > < انڈیا > < آف > < سروے >
لینگویچ > < کوں > < تحقیق > < ایں > < ---- > < کیتن > < صرف > < تے > < تحقیق > < دی > < زبانیں > < سال > < 1887 >
< تقریباً > < وچ > < ہندستان > < متحدہ > < جنھاں > < ھَن > < محقق > < وڈے > < بہوں > < ھَن > < وی > < ڈی > < ایچ >
< پی > < نال > < نال > < دے > < یافتہ > < تعلیم > < اعلیٰ > < اپ > < ھَن > < گریرسن > < سر > < منظوری > < دی >
< نقشے > < ایں > < سکدے۔ > < ونج > < ڈیکھایا > < کوں > < زبانیں > < پاکستانی > < تمام > < وچ > < ایندے > < سرائیکی >
< بشمول > < وچ > < علاقے > < ایں > < مطابق > < دے > < نقشے > < ڳئے > < بڑایا > < نال > < تعاون > < دے > حکومت
< برٹش > < وچ > < 30ء > < ھِن > < شامل > < زبانیں > < گجراتی > < بلوچی > < سندھی > < سرائیکی > < وچ > ے
< نقشہ > < پاکستانی > < ---- > < پاکستانی > < دا > < زبانیں > < پاکستانی > < > < بیٹھوں > < کریندے > < گالھ > < دی > دیقی
< صد < وسیم > < آلے > < لیکن > < لکیراں > < دیاں > < شاعری > < تے > < رہ > < تلے > < چھاں > < دی > < روضے >
< دے > < سئیں > < فرید > < اساں > < اج > < جو > < ھے > < ھی > < برکت > < تے > < فیض > < دا > < شاعری > < دی >
< سئیں > < خواجہ > < اے > < ؟ > < کائنی > < سونہاں > < کون > < دا > < شاعری > < دی > < انہاں > < تے > < سئیں >
< مفرید > < وچ > < سروے > < لنگویچ > < ایں > < ڳئے > < ڈتا > < ناں > < دا > < انڈیا > < آف > < سروے > < لینگویچ >
< کوں > < تحقیق > < ایں > < ---- > < کیتن > < صرف > < تے > < تحقیق > < دی > < زبانیں > < سال > 1887 << تقریباً >
< وچ > < ہندستان > < متحدہ > < جنھاں > < ھَن > < محقق > < وڈے > < بہوں > < ھَن > < وی > < ڈی > < ایچ > < پی > ل
< نا < نال > < دے > < یافتہ > < تعلیم > < اعلیٰ > < اپ > < ھَن > < گریرسن > < سر > < منظوری > < دی > < نقشے > ایں
< < سکدے۔ > < ونج > < ڈیکھایا > < کوں > < زبانیں > < پاکستانی > < تمام > < وچ > < ایندے > < سرائیکی > < بشمول >
< وچ > < علاقے > < ایں > < مطابق > < دے > < نقشے > < ڳئے > < بڑایا > < نال > < تعاون > < دے > < حکومت > < برٹش >
< وچ > < 30ء > < ھِن > < شامل > < زبانیں > < گجراتی > < بلوچی > < سندھی > < سرائیکی > < وچ > < نقشے > تانی
< پاکس < ---- > < ---- > < دا > < زبانیں > < پاکستانی > < > < بیٹھوں > < کریندے > < گالھ > < دی > < صدیقی > < وسیم >
< آلے > < لیکن > < لکیراں > < دیاں > < شاعری > < تے > < رہ > < تلے > < چھاں > < دی > < روضے > < دے > < سئیں >
< فرید > < اساں > < اج > < جو > < ھے > < ھی > < برکت > < تے > < فیض > < دا > < شاعری > < دی > < سئیں > اجہ
< خو < اے > < ؟ > < کائنی > < سونہاں > < کون > < دا > < شاعری > < دی > < انہاں > < تے > < سئیں > < مفرید > وچ
< < سروے > < لنگویچ > < ایں > < ڳئے > < ڈتا > < ناں > < دا > < انڈیا > < آف > < سروے > < لینگویچ > < کوں > تحقیق
< < ایں > < ---- > < کیتن > < صرف > < تے > < تحقیق > < دی > < زبانیں > < سال > 1887 < تقریباً > < وچ > ہندستان
< < متحدہ > < جنھاں > < ھَن > < محقق > < وڈے > < بہوں > < ھَن > < وی > < ڈی > < ایچ > < پی > < نال > < نال >
< دے < یافتہ > < تعلیم > < اعلیٰ > < اپ > < ھَن > < گریرسن > < سر > < منظوری > < دی > < نقشے > < ایں > < سکدے۔ >
< ونج > < ڈیکھایا > < کوں > < زبانیں > < پاکستانی > < تمام > < وچ > < ایندے > < سرائیکی > < بشمول > < وچ > علاقے
< < ایں > < مطابق > < دے > < نقشے > < ڳئے > < بڑایا > < نال > < تعاون > < دے > < حکومت > < برٹش > < وچ > 3ء
0 <7 > < ھِن > < شامل > < زبانیں > < گجراتی > < بلوچی > < سندھی > < سرائیکی > < وچ > < نقشے > < پاکستانی > < ---- >
< نقشہ < دا > < زبانیں > < پاکستانی > < > < بیٹھوں > < کریندے > < گالھ > < دی > < صدیقی > < وسیم > < آلے > لیکن
< < لکیراں > < دیاں > < شاعری > < تے > < رہ > < تلے > < چھاں > < دی > < روضے > < دے > < سئیں > < فرید > اں
< اسا < اج > < جو > < ھے > < ھی > < برکت > < تے > < فیض > < دا > < شاعری > < دی > < سئیں > < خواجہ > < اے >
< ؟ > < کائنی > < سونہاں > < کون > < دا > < شاعری > < دی > < انہاں > < تے > < سئیں > < مفرید > < وچ > < سروے >
< لنگویچ > < ایں > < ڳئے > < ڈتا > < ناں > < دا > < انڈیا > < آف > < سروے > < لینگویچ > < کوں > < تحقیق > < ایں > ۔
--- < کیتن > < صرف > < تے > < تحقیق > < دی > < زبانیں > < سال > < 188> < تقریباً > < وچ > < ہندستان > < متحدہ >
< جنھاں > < ھَن > < محقق > < وڈے > < بہوں > < ھَن > < وی > < ڈی > < ایچ > < پی > < نال > < نال > < دے > < یافتہ >
< تعلیم > < اعلیٰ > < اپ > < ھَن > < گریرسن > < سر > < منظوری > < دی > < نقشے > < ایں > < سکدے۔ > < ونج > کھایا
< ڈی < کوں > < زبانیں > < پاکستانی > < تمام > < وچ > < ایندے > < سرائیکی > < بشمول > < وچ > < علاقے > < ایں > ابق
< مط < دے > < نقشے > < ڳئے > < بڑایا > < نال > < تعاون > < دے > < حکومت > < برٹش > < وچ > < 87 30ء > < ھِن >
< شامل > < زبانیں > < گجراتی > < بلوچی > < سندھی > < سرائیکی > < وچ > < نقشے > < پاکستانی > < ---- > < دا > <
زبانیں > < پاکستانی > < > < بیٹھوں > < کریندے > < گالھ > < دی > < صدیقی > < وسیم > < آلے > < لیکن > < لکیراں > یاں
< د < شاعری > < تے > < رہ > < تلے > < چھاں > < دی > < روضے > < دے > < سئیں > < فرید > < اساں > < اج > < جو >
< ھے > < ھی > < برکت > < تے > < فیض > < دا > < شاعری > < دی > < سئیں > < خواجہ > < اے > < ؟ > < کائنی > <
< سونہاں > < کون > < دا > < شاعری > < دی > < انہاں > < تے > < سئیں > < مفرید > < وچ > < سروے > < لنگویچ > < ایں >
< ڳئے > < ڈتا > < ناں > < دا > < انڈیا > < آف > < سروے > < لینگویچ > < کوں > < تحقیق > < ایں > < ---- > < کیتن > ف
< صر < تے > < تحقیق > < دی > < زبانیں > < سال > 18 << تقریباً > < وچ > < ہندستان > < متحدہ > < جنھاں > < ھَن >
< محقق > < وڈے > < بہوں > < ھَن > < وی > < ڈی > < ایچ > < پی > < نال > < نال > < دے > < یافتہ > < تعلیم > < اعلیٰ >
< اپ > < ھَن > < گریرسن > < سر > < منظوری > < دی > < نقشے > < ایں > < سکدے۔ > < ونج > < ڈیکھایا > < کوں > یں
< زبان < پاکستانی > < تمام > < وچ > < ایندے > < سرائیکی > < بشمول > < وچ > < علاقے > < ایں > < مطابق > < دے > ے
< نقش < ڳئے > < بڑایا > < نال > < تعاون > < دے > < حکومت > < برٹش > < وچ > < 30887ء > < ھِن > < شامل > زبانیں
< گجراتی > < بلوچی > < سندھی > < سرائیکی > < وچ > < نقشے > < پاکستانی > < ---- > < دا > < زبانیں > < >
< بیٹھوں > < کریندے > < گالھ > < دی > < صدیقی > < وسیم > < آلے > < لیکن > < لکیراں > < دیاں > < شاعری > < تے >
< رہ < تلے > < چھاں > < دی > < روضے > < دے > < سئیں > < فرید > < اساں > < اج > < جو > < ھے > < ھی > برکت

< تے > < فیض > < دا > < شاعری > < دی > < سئیں > < خواجہ > < اے > < ؟ > < کائنی > < سونہاں > < کون > < دا >
< شاعری > < دی > < انہاں > < تے > < سئیں > < مفرید > < وچ > < سروے > < لنگویچ > < ایں > < گِئے > < ڈٹا > < ناں >
< دا > < انڈیا > < آف > < سروے > < لینگویچ > < کوں > < تحقیق > < ایں > < --- > < کیتن > < صرف > < تے > < تحقیق >
< دی > < زبانیں > < سال > < 10 > < تقریباً > < وچ > < ہندستان > < متحدہ > < جنھاں > < ھَن > < محقق > < وڈے > < بہوں >
< ھَن > < وی > < ڈی > < ایچ > < پی > < نال > < نال > < دے > < یافتہ > < تعلیم > < اعلیٰ > < اپ > < ھَن > < گریرسن >
< سر > < منظوری > < دی > < نقشے > < ایں > < سکدے۔ > < ونج > < ڈیکھایا > < کوں > < زبانیں > < پاکستانی > < تمام >
< وچ > < ایندے > < سرائیکی > < بشمول > < وچ > < علاقے > < ایں > < مطابق > < دے > < نقشے > < گِئے > < بڑایا > نال
< تعاون > < دے > < حکومت > < برٹش > < وچ > < 31887ء > < ھِن > < شامل > dزبانیں < دا # NG# > <
< پاکستانی > < --- > پاکستانی #NG# > < نقشے #NG# > < وچ #N> سرائیکی #N> سندھی # P#
< بلوچی #N> گجراتی #NG# > < > < ھِن تعلیم > < الصّوت > < دیاں > < سئیں > < کریندے > < وے
< سر > < مفرید > < بیٹھوں > < ایچ > < زبانیں > < نقشہ > < ھَن > < ھِن > < لیکن > < اے > < وڈے > < برٹش
< انڈیا > < سونہاں > < ڈٹا > < تے > < فرید > < سر > < اپ > < ہندستان > < بشمول > < تعاون > < چھاں >
< حکومت > < لنگویچ > < محقق > < ھے > < نقشے > < پی > < بڑایا > < وسیم > < کائنی > < تقریباً > < دی >
< بہوں > < وی > < اساں > < کوں > < گِئے > < اعلیٰ > < دا > < گجراتی > < لینگویچ > < وچ > < صرف >
< --- > < ڈی > < برکت > < ھی > < تمام > < ایندے > < روضے > < کون > < جو > < نال > < منظوری > < یتن
< ک > < آلے > < سکدے۔ > < سرائیکی > < ڈیکھایا > < دے > < ؟ > < مطابق > < سال > < بلوچی > < ء > <<
< انہ > < یافتہ > < ناں > < جنھاں > < شاعری > < ایں > < پاکستانی > < صدیقی > < شامل1887 > < فیض > خوااں
< متحدہ > < اج > < گریرسن > < تحقیق > < گالھ > < ونج > < لکیراں > < ائے > < رہ > < تلے > < جلی >
< آف > < > < شامل > < فیض > < تعلیم > < الصّوت > < دیاں > < سئیں > < کریندے > < سروے > < مفرید >
بیٹھوں > < ایچ > < زبانیں > < نقشہ > < ھَن > < ھِن > < لیکن > < اے > < وڈے > < برٹش > < انڈیا > < سونہاں
< ڈٹا > < تے > < فرید > < سر > < اپ > < ہندستان > < بشمول > < تعاون > < چھاں > < حکومت > < لنگویچ
< محقق > < ھے > < نقشے > < پی > < بڑایا > < وسیم > < کائنی > < تقریباً > < دی > < بہوں > < وی >
< اساں > < کوں > < گِئے > < اعلیٰ > < دا > < گجراتی > < لینگویچ > < وچ > < صرف > < --- > < ڈی > < کت
< بر > < ھی > < تمام > < ایندے > < روضے > < کون > < جو > < نال > < منظوری > < کیتن > < آلے > < دے۔
< سک > < سرائیکی > < ڈیکھایا > < دے > < ؟ > < مطابق > < سال > < بلوچی > < 30ء > < سندھی > < انہاجہ
< یافتہ > < ناں > < جنھاں > < شاعری > < > < بیٹھوں > < کریندے > < گالھ > < دی > < صدیقی > < وسیم > < آلے >
< لیکن > < لکیراں > < دیاں > < شاعری > < تے > < رہ > < تلے > < چھاں > < دی > < روضے > < دے > < سئیں > < فرید
< اساں > < اج > < جو > < ھے > < ھی > < برکت > < تے > < فیض > < دا > < شاعری > < دی > < سئیں > < خواجہ >
< اے > < ؟ > < کائنی > < سونہاں > < کون > < دا > < شاعری > < دی > < انہاں > < تے > < سئیں > < مفرید > < وچ > < وے
< سر > < لنگویچ > < ایں > < گِئے > < ڈٹا > < ناں > < دا > < انڈیا > < آف > < سروے > < لینگویچ > < کوں > < تحقیق > < ایں
< --- > < کیتن > < صرف > < تے > < تحقیق > < دی > < زبانیں > < سال > < 1887 > < علاقے > < ں > < ہندستان >
< متحدہ > < جنھاں > < ھَن > < محقق > < وڈے > < بہوں > < ھَن > < وی > < ڈی > < ایچ > < پی > < نال > < نال > < دے >
< یافتہ > < تعلیم > < اعلیٰ > < اپ > < ھَن > < گریرسن > < سر > < منظوری > < دی > < نقشے > < ایں > < سکدے۔ > ونج
< ڈیکھایا > < کوں > < زبانیں > < پاکستانی > < تمام > < وچ > < ایندے > < سرائیکی > < بشمول > < وچ > < علاقے > ایں
< مطابق > < دے > < نقشے > < گِئے > < بڑایا > < نال > < تعاون > < دے > < حکومت > < برٹش > < وچ > < 30ء >
< سندھی > < > وچ > < زبانیں > < گجراتی > < بلوچی > < سندھی > < ایں > < پاکستانی > < صدیقی > < 1887 > < تقریباً >
< خواجہ > < متحدہ > < اج > < گریرسن > < تحقیق > < گالھ > < ونج > < لکیراں > < ائے > < رہ > < تلے > لی
< جہ > < آف > < > < > < بیٹھوں > < کریندے > < گالھ > < دی > < صدیقی > < وسیم > < آلے > < لیکن > < لکیراں > < دیاں >
< شاعری > < تے > < رہ > < تلے > < چھاں > < دی > < روضے > < دے > < سئیں > < فرید > < اساں > < اج > < جو > < ے
< ھ > < ھی > < برکت > < تے > < فیض > < دا > < شاعری > < دی > < سئیں > < خواجہ > < اے > < ؟ > < کائنی > سونہاں
< کون > < دا > < شاعری > < دی > < انہاں > < تے > < سئیں > < مفرید > < وچ > < سروے > < لنگویچ > < ایں > < گِئے
< ڈٹا > < ناں > < دا > < انڈیا > < آف > < سروے > < لینگویچ > < کوں > < تحقیق > < ایں > < --- > < کیتن > < صرف >
< تے > < تحقیق > < دی > < زبانیں > < سال > < 30 > < ھِن > < وچ شامل > < ہندستان > < متحدہ > < جنھاں > < ھَن > < قق
< محد > < وڈے > < بہوں > < ھَن > < وی > < ڈی > < ایچ > < پی > < نال > < نال > < دے > < یافتہ > < تعلیم > < اعلیٰ > < پ
< ا > < ھَن > < گریرسن > < سر > < منظوری > < دی > < نقشے > < ایں > < سکدے۔ > < ونج > < ڈیکھایا > < کوں > < زبانیں
< پاکستانی > < تمام > < وچ > < ایندے > < سرائیکی > < بشمول > < وچ > < علاقے > < ایں > < مطابق > < دے > < نقشے
< گِئے > < بڑایا > < نال > < تعاون > < دے > < حکومت > < برٹش > < وچ > < 30ء > < علاقے > < > < > < زبانیں >
< گجراتی > < بلوچی > < سندھی > < سرائیکی > < وچ > < نقشے > < پاکستانی > < --- > < سرائیکی > < دا > < زبانیں > ستانی
< پاک > < > < بیٹھوں > < کریندے > < گالھ > < دی > < صدیقی > < وسیم > < آلے > < لیکن > < لکیراں > < دیاں > < شاعری >
< تے > < رہ > < تلے > < چھاں > < دی > < روضے > < دے > < سئیں > < فرید > < اساں > < اج > < جو > < ھے > ھی
< برکت > < تے > < فیض > < دا > < شاعری > < دی > < سئیں > < خواجہ > < اے > < ؟ > < کائنی > < سونہاں > کون
< دا > < شاعری > < دی > < انہاں > < تے > < سئیں > < مفرید > < وچ > < سروے > < لنگویچ > < ایں > < گِئے > < ڈٹا >
< ناں > < دا > < انڈیا > < آف > < سروے > < لینگویچ > < کوں > < تحقیق > < ایں > < --- > < کیتن > < صرف > < تے >
< تحقیق > < دی > < زبانیں > < سال > < 1887 > < تقریباً > < وچ شا > < ہندستان > < متحدہ > < جنھاں > < ھَن > < محقق > وڈے
< بہوں > < ھَن > < وی > < ڈی > < ایچ > < پی > < نال > < نال > < دے > < یافتہ > < تعلیم > < اعلیٰ > < اپ > < ھَن >

پاکستانی > < زبانیں > < کوں > < ڈیکھایا > < ونج > < سکدے۔ > < ایں > < نقشے > < دی > < منظوری > < سر > < گریرسن >
> < گِئے > < نقشے > < دے > < مطابق > < ایں > < علاقے > < وچ > < بشمول > < سرائیکی > < ایندے > < وچ > < تمام > <
> < گجراتی > < زبانیں > <ش> <مل > < ہِن > < 30ء > < وچ > < برٹش > < حکومت > < دے > < تعاون > < نال > < بڑایا
ٹھوں > < > < پاکستانی > < زبانیں > < دا > < وچ > < ---- > < پاکستانی > < نقشے > < وچ > < سرائیکی > < سندھی > < بلوچی
< رہ > < تے > < شاعری > < دیاں > < لکیراں > < لیکن > < آلے > < وسیم > < صدیقی > < دی > < گالھ> < کریندے > < بیٹ
> < برکت > < ھی > < ھے > < جو > < اج > < اساں > < فرید > < سئیں > < دے > < روضے > < دی > < چھاں > < تلے >
عری > < دا > < کون > < سونہاں > < کائنی > < ؟ > < اے > < خواجہ > < سئیں > < دی > < شاعری > < دا > < فیض > < تے
< دا > < ناں > < ڈتا > < گِئے > < ایں > < لنگویچ > < سروے > < وچ > < مفرید > < سئیں > < تے > < انہاں > < دی > < شا
< دی > < تحقیق > < تے > < صرف > < کیتن > < ---- > < ایں > < تحقیق > < کوں > < لینگویچ > < سروے > < آف > < انڈیا >
َن > < بہوں > < وڈے > < محقق > < ہَن > < جنہاں > < متحدہ > < ہندستان > < وچ >امہ تقریباً > < 1887 > < سال > < زبانیں >
ر > < گریرسن > < ہَن > < اپ > < اعلیٰ > < تعلیم > < یافتہ > < دے > < نال > < نال > < پی > < ایچ > < ڈی > < وی > < ھ
وچ > < تمام > < پاکستانی > < زبانیں > < کوں > < ڈیکھایا > < ونج > < سکدے۔ > < ایں > < نقشے > < دی > < منظوری > < سد
< نال > < بڑایا > < گِئے > < نقشے > < دے > < مطابق > < ایں > < علاقے > < وچ > < بشمول > < سرائیکی > < ایندے > <
سندھی > < بلوچی > < گجراتی > < زبانیں > < شا <ل > < ہِن > < 30ء > < وچ > < برٹش > < حکومت > < دے > < تعاون >
کریندے > < بیٹھوں > < > < پاکستانی > < زبانیں > < دا > < نقشے > < ---- > < پاکستانی > < نقشے > < وچ > < سرائیکی > <
چھاں > < تلے > < رہ > < تے > < شاعری > < دیاں > < لکیراں > < لیکن > < آلے > < وسیم > < صدیقی > < دی > < گالھ > <
< فیض > < تے > < برکت > < ھی > < ھے > < جو > < اج > < اساں > < فرید > < سئیں > < دے > < روضے > < دی > <
> < دی > < شاعری > < دا > < کون > < سونہاں > < کائنی > < ؟ > < اے > < خواجہ > < سئیں > < دی > < شاعری > < دا >
< آف > < انڈیا > < دا > < ناں > < ڈتا > < گِئے > < ایں > < لنگویچ > < سروے > < وچ > < مفرید > < سئیں > < تے > < انہاں
ال > < زبانیں > < دی > < تحقیق > < تے > < صرف > < کیتن > < ---- > < ایں > < تحقیق > < کوں > < لینگویچ > < سروے >
> < وی > < ہَن > < بہوں > < وڈے > < محقق > < ہَن > < جنہاں > < متحدہ > < ہندستان > < وچ > ملتقریباً > < 1887 > < سد
منظوری > < سر > < گریرسن > < ہَن > < اپ > < اعلیٰ > < تعلیم > < یافتہ > < دے > < نال > < نال > < پی > < ایچ > < ڈی
> < ایندے > < وچ > < تمام > < پاکستانی > < زبانیں > < کوں > < ڈیکھایا > < ونج > < سکدے۔ > < ایں > < نقشے > < دی > <
دے > < تعاون > < نال > < بڑایا > < گِئے > < نقشے > < دے > < مطابق > < ایں > < علاقے > < وچ > < بشمول > < سرائیکی
< سرائیکی > < سندھی > < بلوچی > < گجراتی > < زبانیں > < شام < ت > < ہِن > < 30ء > < وچ > < برٹش > < حکومت > <
> < گالھ > < کریندے > < بیٹھوں > < > < پاکستانی > < زبانیں > < دا > < پاکستانی > < ---- > < پاکستانی > < نقشے > < وچ >
> < دی > < چھاں > < تلے > < رہ > < تے > < شاعری > < دیاں > < لکیراں > < لیکن > < آلے > < وسیم > < صدیقی > < دی
اعری > < دا > < فیض > < تے > < برکت > < ھی > < ھے > < جو > < اج > < اساں > < فرید > < سئیں > < دے > < روضے
< تے > < انہاں > < دی > < شاعری > < دا > < کون > < سونہاں > < کائنی > < ؟ > < اے > < خواجہ > < سئیں > < دی > < شد
> < سروے > < آف > < انڈیا > < دا > < ناں > < ڈتا > < گِئے > < ایں > < لنگویچ > < سروے > < وچ > < مفرید > < سئیں >
< 1887 > < سال > < زبانیں > < دی > < تحقیق > < تے > < صرف > < کیتن > < ---- > < ایں > < تحقیق > < کوں > < لینگویچ
< ایچ > < ڈی > < وی > < ہَن > < بہوں > < وڈے > < محقق > < ہَن > < جنہاں > < متحدہ > < ہندستان > < وچ > < ل قریباً >
> < دی > < منظوری > < سر > < گریرسن > < ہَن > < اپ > < اعلیٰ > < تعلیم > < یافتہ > < دے > < نال > < نال > < پی >
> < سرائیکی > < ایندے > < وچ > < تمام > < پاکستانی > < زبانیں > < کوں > < ڈیکھایا > < ونج > < سکدے۔ > < ایں > < نقشے
حکومت > < دے > < تعاون > < نال > < بڑایا > < گِئے > < نقشے > < دے > < مطابق > < ایں > < علاقے > < وچ > < بشمول
ے > < وچ > < سرائیکی > < سندھی > < بلوچی > < گجراتی > < زبانیں > < شامل< تقہ > < ہِن > < 30ء > < وچ > < برٹش > <
< صدیقی > < دی > < گالھ > < کریندے > < بیٹھوں > < > < پاکستانی > < زبانیں > < دا > < ---- > < ---- > < پاکستانی > < نقشہ
< دے > < روضے > < دی > < چھاں > < تلے > < رہ > < تے > < شاعری > < دیاں > < لکیراں > < لیکن > < آلے > < وسیم >
ئیں > < دی > < شاعری > < دا > < فیض > < تے > < برکت > < ھی > < ھے > < جو > < اج > < اساں > < فرید > < سئیں >
مفرید > < سئیں > < تے > < انہاں > < دی > < شاعری > < دا > < کون > < سونہاں > < کائنی > < ؟ > < اے > < خواجہ > < سد
کوں > < لینگویچ > < سروے > < آف > < انڈیا > < دا > < ناں > < ڈتا > < گِئے > < ایں > < لنگویچ > < سروے > < وچ > <
وچ > < >ربیاً > < 1887 > < سال > < زبانیں > < دی > < تحقیق > < تے > < صرف > < کیتن > < ---- > < ایں > < تحقیق > <
< نال > < پی > < ایچ > < ڈی > < وی > < ہَن > < بہوں > < وڈے > < محقق > < ہَن > < جنہاں > < متحدہ > < ہندستان > <
> < ایں > < نقشے > < دی > < منظوری > < سر > < گریرسن > < ہَن > < اپ > < اعلیٰ > < تعلیم > < یافتہ > < دے > < نال >
< وچ > < بشمول > < سرائیکی > < ایندے > < وچ > < تمام > < پاکستانی > < زبانیں > < کوں > < ڈیکھایا > < ونج > < سکدے۔
وچ > < برٹش > < حکومت > < دے > < تعاون > < نال > < بڑایا > < گِئے > < نقشے > < دے > < مطابق > < ایں > < علاقے >
< پاکستانی > < نقشے > < وچ > < سرائیکی > < سندھی > < بلوچی > < گجراتی > < زبانیں > < شامل تقر < ہِن > < 30ء > <
< آلے > < وسیم > < صدیقی > < دی > < گالھ > < کریندے > < بیٹھوں > < > < پاکستانی > < زبانیں > < دا > < نقشہ > < ----
فرید > < سئیں > < دے > < روضے > < دی > < چھاں > < تلے > < رہ > < تے > < شاعری > < دیاں > < لکیراں > < لیکن >
< خواجہ > < سئیں > < دی > < شاعری > < دا > < فیض > < تے > < برکت > < ھی > < ھے > < جو > < اج > < اساں > <
ے > < وچ > < مفرید > < سئیں > < تے > < انہاں > < دی > < شاعری > < دا > < کون > < سونہاں > < کائنی > < ؟ > < اے >
ں > < تحقیق > < کوں > < لینگویچ > < سروے > < آف > < انڈیا > < دا > < ناں > < ڈتا > < گِئے > < ایں > < لنگویچ > < سرو
ہ > < ہندستان > < وچ > < > یباً > < 1887 > < سال > < زبانیں > < دی > < تحقیق > < تے > < صرف > < کیتن > < ---- > < اید
> < دے > < نال > < نال > < پی > < ایچ > < ڈی > < وی > < ہَن > < بہوں > < وڈے > < محقق > < ہَن > < جنہاں > < متحد
< ونج > < سکدے۔ > < ایں > < نقشے > < دی > < منظوری > < سر > < گریرسن > < ہَن > < اپ > < اعلیٰ > < تعلیم > < یافتہ

ایں > < علاقے > < وچ > < بشمول > < سرائیکی > < ایندے > < وچ > < تمام > < پاکستانی > < زبانیں > < کوں > < ڈیکھایا >
ِن > < 30ء > < وچ > < برٹش > < حکومت > < دے > < تعاون > < نال > < بڑایا > < ڳئے > < نقشے > < دے > < مطابق > <
ھ < تقریِ> شامل < > زبانیں < > گجراتی < > بلوچی < > سندھی < > سرائیکی < > وچ < > نقشے < > پاکستانی < > --- < > دا < >
لکیراں > < لیکن > < آلے > < وسیم > < صدیقی > < دی > < گالھ > < کریندے > < بیٹھوں > < > < پاکستانی > < زبانیں > < دا
< اج > < اساں > < فرید > < سئیں > < دے > < روضے > < دی > < چھاں > < تلے > < رہ > < تے > < شاعری > < دیاں > <
ائنی > < ؟ > < اے > < خواجہ > < سئیں > < دی > < شاعری > < دا > < فیض > < تے > < برکت > < ھی > < ھے > < جو >
> < لنگویچ > < سروے > < وچ > < مفرید > < سئیں > < تے > < انہاں > < دی > < شاعری > < دا > < کون > < سونہاں > < ک
< کیتن > < --- > < ایں > < تحقیق > < کوں > < لینگویچ > < سروے > < آف > < انڈیا > < دا > < ناں > < ڈتا > < ڳئے > < ایں
> < جنھاں > < متحدہ > < ہندستان > < وچ > < تا بآ > < 1887 > < سال > < زبانیں > < دی > < تحقیق > < تے > < صرف >
> < تعلیم > < یافتہ > < دے > < نال > < نال > < پی > < ایچ > < ڈی > < وی > < ھَن > < بہوں > < وڈے > < محقق > < ھَن
کوں > < ڈیکھایا > < ونج > < سکدے۔ > < ایں > < نقشے > < دی > < منظوری > < سر > < گریرسن > < ھَن > < اپ > < اعلیٰ
دے > < مطابق > < ایں > < علاقے > < وچ > < بشمول > < سرائیکی > < ایندے > < وچ > < تمام > < پاکستانی > < زبانیں > <
> < شامل > قریبِ< ھِن > < 30ء > < وچ > < برٹش > < حکومت > < دے > < تعاون > < نال > < بڑایا > < ڳئے > < نقشے > <
< زبانیں > < دا > < زبانیں > < --- > < پاکستانی > < نقشے > < وچ > < سرائیکی > < سندھی > < بلوچی > < گجراتی > < زبانیں
ے > < شاعری > < دیاں > < لکیراں > < لیکن > < آلے > < وسیم > < صدیقی > < دی > < گالھ > < کریندے > < بیٹھوں > < >
> < ھی > < ھے > < جو > < اج > < اساں > < فرید > < سئیں > < دے > < روضے > < دی > < چھاں > < تلے > < رہ > < ت
ا > < کون > < سونہاں > < کائنی > < ؟ > < اے > < خواجہ > < سئیں > < دی > < شاعری > < دا > < فیض > < تے > < برکت
اں > < ڈتا > < ڳئے > < ایں > < لنگویچ > < سروے > < وچ > < مفرید > < سئیں > < تے > < انہاں > < دی > < شاعری > < د
تحقیق > < تے > < صرف > < کیتن > < --- > < ایں > < تحقیق > < کوں > < لینگویچ > < سروے > < آف > < انڈیا > < دا > < ن
> < وڈے > < محقق > < ھَن > < جنھاں > < متحدہ > < ہندستان > < وچ > < تق<اً > < 1887 > < سال > < زبانیں > < دی > <
سن > < ھَن > < اپ > < اعلیٰ > < تعلیم > < یافتہ > < دے > < نال > < نال > < پی > < ایچ > < ڈی > < وی > < ھَن > < بہوں
> < پاکستانی > < زبانیں > < کوں > < ڈیکھایا > < ونج > < سکدے۔ > < ایں > < نقشے > < دی > < منظوری > < سر > < گریر
بڑایا > < ڳئے > < نقشے > < دے > < مطابق > < ایں > < علاقے > < وچ > < بشمول > < سرائیکی > < ایندے > < وچ > < تمام
> < نال > < تعاون > < دے > < حکومت > < برٹش > < وچ > < 30ء > < ھِن ریبا < شامل > < زبانیں1887 > < ً
