# Supplementary material for: Mixed Script Identification Using Automated DNN Hyperparameter Optimization
Source: Comput Intell Neurosci. 2021 Dec 10;2021:8415333. doi: 10.1155/2021/8415333 (PMC8683192; doi:10.1155/2021/8415333)
Supplement: Supplementary Materials — (e.g., datasets or results outcomes in the form of graphs) from different stages are provided with the manuscript. The graphs including system training, validation, and the testing outcome of all RNN variants are included in Supplementary Materials. [file 8415333.f1.zip › 8415333.f1/words3.pdf]

NG# نقشے > < NG# دا > < Sad # P# پاکستانی > < --- > < پاکستانی > < NG# پاکستانی > < زبانیں > < پاکستانی > < زبانیں
پاکستانی > < NG# گجراتی > N# < بلوچی > < P# سندھی > N# < سرائیکی > N# < وچ > < #
بیٹھوں > < مفرید > < سروے > < کریندے > < سئیں > < دیاں > < الصّوت > < تعلیم > < فیض > < شامل > < > <
> < سونہاں > < انڈیا > < برٹش > < وڈے > < اے > < لیکن > < ھِن > < ھَن > < نقشہ > < زبانیں > < ایچ > <
> < لنگویچ > < حکومت > < چھاں > < تعاون > < بشمول > < ہندستان > < اپ > < سر > < فرید > < تے > < تے > < ڈتا
اساں > < وی > < بہوں > < دی > < تقریباً > < کائنی > < وسیم > < بڑایا > < پی > < نقشے > < ھے > < محقق
< برکت > < ڈی > < --- > < صرف > < وچ > < لینگویچ > < گجراتی > < دا > < اعلیٰ > < گِئے > < کوں > <
< سکدے۔ > < آلے > < کیتن > < منظوری > < نال > < جو > < کون > < روضے > < ایندے > < تمام > < ھی >
یافتہ > < انہاں > < سنۃ Sa# P # ء > < بلوچی > < سال > < مطابق > < ؟ > < دے > < ڈیکھایا > < سرائیکی >
< لیکن > < آلے > < وسیم > < صدیقی > < دی > < گالھ > < کریندے > < بیٹھوں > < > < شاعری > < جنھاں > < ناں > <
اساں > < فرید > < سئیں > < دے > < روضے > < دی > < چھاں > < تلے > < رہ > < تے > < شاعری > < دیاں > < لکیراں >
> < اے > < خواجہ > < سئیں > < دی > < شاعری > < دا > < فیض > < تے > < برکت > < ھی > < ھے > < جو > < اج > <
> < سروے > < وچ > < مفرید > < سئیں > < تے > < انہاں > < دی > < شاعری > < دا > < کون > < سونہاں > < کائنی > < ؟
--- > < این > < تحقیق > < کوں > < لینگویچ > < سروے > < آف > < انڈیا > < دا > < ناں > < ڈتا > < گِئے > < این > < لنگویچ
> < متحدہ > < ہندستان > < وچ > < تقردھ زبانیں1887 > < سال > < زبانیں > < دی > < تحقیق > < تے > < صرف > < کیتن > <
> < یافتہ > < دے > < نال > < نال > < پی > < ایچ > < ڈی > < وی > < ھَن > < بہوں > < وڈے > < محقق > < ھَن > < جنھاں
ڈیکھایا > < ونج > < سکدے۔ > < این > < نقشے > < دی > < منظوری > < سر > < گریرسن > < ھَن > < اپ > < اعلیٰ > < تعلیم
مطابق > < این > < علاقے > < وچ > < بشمول > < سرائیکی > < ایندے > < وچ > < تمام > < پاکستانی > < زبانیں > < کوں > <
مل > < بیائی > < 30ء > < وچ > < برٹش > < حکومت > < دے > < تعاون > < نال > < بڑایا > < گِئے > < نقشے > < دے > <
< خواجہ > < علاقہ > < 1887 > < صدیقی > < پاکستانی > < این > < سندھی > < بلوچی > < گجراتی > < زبانیں > < شا
< جلی > < تلے > < رہ > < ائے > < لکیراں > < ونج > < گالھ > < تحقیق > < گریرسن > < اج > < متحدہ > <
< شاعری > < دیاں > < لکیراں > < لیکن > < آلے > < وسیم > < صدیقی > < دی > < گالھ > < کریندے > < بیٹھوں > < > < آف
ھی > < ھے > < جو > < اج > < اساں > < فرید > < سئیں > < دے > < روضے > < دی > < چھاں > < تلے > < رہ > < تے >
کون > < سونہاں > < کائنی > < ؟ > < اے > < خواجہ > < سئیں > < دی > < شاعری > < دا > < فیض > < تے > < برکت > <
< ڈتا > < گِئے > < این > < لنگویچ > < سروے > < وچ > < مفرید > < سئیں > < تے > < انہاں > < دی > < شاعری > < دا > <
> < تے > < صرف > < کیتن > < --- > < این > < تحقیق > < کوں > < لینگویچ > < سروے > < آف > < انڈیا > < دا > < ناں
ے > < محقق > < ھَن > < جنھاں > < متحدہ > < ہندستان > < وچ > < تقریبےن > < 30 > < سال > < زبانیں > < دی > < تحقیق
ھَن > < اپ > < اعلیٰ > < تعلیم > < یافتہ > < دے > < نال > < نال > < پی > < ایچ > < ڈی > < وی > < ھَن > < بہوں > < وڈ
ستانی > < زبانیں > < کوں > < ڈیکھایا > < ونج > < سکدے۔ > < این > < نقشے > < دی > < منظوری > < سر > < گریرسن > <
< گِئے > < نقشے > < دے > < مطابق > < این > < علاقے > < وچ > < بشمول > < سرائیکی > < ایندے > < وچ > < تمام > < پاک
< گجراتی > < زبانیں > < شامل > < ھاً > < 30ء > < وچ > < برٹش > < حکومت > < دے > < تعاون > < نال > < بڑایا >
< > < پاکستانی > < زبانیں > < دا > < سرائیکی > < --- > < پاکستانی > < نقشے > < وچ > < سرائیکی > < سندھی > < بلوچی
ہ > < تے > < شاعری > < دیاں > < لکیراں > < لیکن > < آلے > < وسیم > < صدیقی > < دی > < گالھ > < کریندے > < بیٹھوں
< برکت > < ھی > < ھے > < جو > < اج > < اساں > < فرید > < سئیں > < دے > < روضے > < دی > < چھاں > < تلے > < ر
< دا > < کون > < سونہاں > < کائنی > < ؟ > < اے > < خواجہ > < سئیں > < دی > < شاعری > < دا > < فیض > < تے >
ا > < ناں > < ڈتا > < گِئے > < این > < لنگویچ > < سروے > < وچ > < مفرید > < سئیں > < تے > < انہاں > < دی > < شاعری
< تحقیق > < تے > < صرف > < کیتن > < --- > < این > < تحقیق > < کوں > < لینگویچ > < سروے > < آف > < انڈیا > < د
بہوں > < وڈے > < محقق > < ھَن > < جنھاں > < متحدہ > < ہندستان > < وچ > < تقریباں > < 1887 > < سال > < زبانیں > < دی
گریرسن > < ھَن > < اپ > < اعلیٰ > < تعلیم > < یافتہ > < دے > < نال > < نال > < پی > < ایچ > < ڈی > < وی > < ھَن > <
< تمام > < پاکستانی > < زبانیں > < کوں > < ڈیکھایا > < ونج > < سکدے۔ > < این > < نقشے > < دی > < منظوری > < سر >
< بڑایا > < گِئے > < نقشے > < دے > < مطابق > < این > < علاقے > < وچ > < بشمول > < سرائیکی > < ایندے > < وچ >
ی > < بلوچی > < گجراتی > < زبانیں > < شامل > < ھًّ > < 30ء > < وچ > < برٹش > < حکومت > < دے > < تعاون > < نال
ریندے > < بیٹھوں > < > < پاکستانی > < زبانیں > < دا > < وچ > < --- > < پاکستانی > < نقشے > < وچ > < سرائیکی > < سندھ
اں > < تلے > < رہ > < تے > < شاعری > < دیاں > < لکیراں > < لیکن > < آلے > < وسیم > < صدیقی > < دی > < گالھ > < ک
فیض > < تے > < برکت > < ھی > < ھے > < جو > < اج > < اساں > < فرید > < سئیں > < دے > < روضے > < دی > < چھ
< دی > < شاعری > < دا > < کون > < سونہاں > < کائنی > < ؟ > < اے > < خواجہ > < سئیں > < دی > < شاعری > < دا > <
ف > < انڈیا > < دا > < ناں > < ڈتا > < گِئے > < این > < لنگویچ > < سروے > < وچ > < مفرید > < سئیں > < تے > < انہاں >
< زبانیں > < دی > < تحقیق > < تے > < صرف > < کیتن > < --- > < این > < تحقیق > < کوں > < لینگویچ > < سروے > < آ
< وی > < ھَن > < بہوں > < وڈے > < محقق > < ھَن > < جنھاں > < متحدہ > < ہندستان > < وچ > < تقریباًں > < 1887 > < سال
نظوری > < سر > < گریرسن > < ھَن > < اپ > < اعلیٰ > < تعلیم > < یافتہ > < دے > < نال > < نال > < پی > < ایچ > < ڈی >
< ایندے > < وچ > < تمام > < پاکستانی > < زبانیں > < کوں > < ڈیکھایا > < ونج > < سکدے۔ > < این > < نقشے > < دی > < م
< تعاون > < نال > < بڑایا > < گِئے > < نقشے > < دے > < مطابق > < این > < علاقے > < وچ > < بشمول > < سرائیکی >
سرائیکی > < سندھی > < بلوچی > < گجراتی > < زبانیں > < شامل > < ھِن > < 30ء > < وچ > < برٹش > < حکومت > < دے
< گالھ > < کریندے > < بیٹھوں > < > < پاکستانی > < زبانیں > < دا > < نقشے > < --- > < پاکستانی > < نقشے > < وچ > <
< دی > < چھاں > < تلے > < رہ > < تے > < شاعری > < دیاں > < لکیراں > < لیکن > < آلے > < وسیم > < صدیقی > < دی

< روضے > < دے > < سئیں > < فرید > < اساں > < اج > < جو > < ہے > < ھی > < برکت > < تے > < فیض > < دا > اعری
< شد > < دی > < سئیں > < خواجہ > < اے > < ؟ > < کائنی > < سونہاں > < کون > < دا > < شاعری > < دی > < انہاں > < تے
< سئیں > < مفرید > < وچ > < سروے > < لنگویچ > < ایں > < گئے > < ڈتا > < ناں > < دا > < انڈیا > < آف > < سروے >
لینگویچ > < کوں > < تحقیق > < ایں > < ---- > < کیتن > < صرف > < تے > < تحقیق > < دی > < زبانیں > < سال > < 1887
< تقریباً > < وچ > < ہندستان > < متحدہ > < جنھاں > < ہَن > < محقق > < وڈّے > < بہوں > < ہَن > < وی > < ڈی > < ایچ
< پی > < نال > < دے > < یافتہ > < تعلیم > < اعلیٰ > < اپ > < ہَن > < گریرسن > < سر > < منظوری > < دی >
< نقشے > < ایں > < سکدے۔ > < ونج > < ڈیکھایا > < کوں > < زبانیں > < پاکستانی > < تمام > < وچ > < ایندے > < سرائیکی > <
< بشمول > < وچ > < علاقے > < ایں > < مطابق > < دے > < نقشے > < گئے > < بڑایا > < نال > < تعاون > < دے > حکومت
< برٹش > < وچ > < 30ء > < ہِن > < شامل > < زبانیں > < گجراتی > < بلوچی > < سندھی > < سرائیکی > < وچ > ے
< نقشہ > < پاکستانی > < ---- > < پاکستانی > < دا > < زبانیں > < پاکستانی > < > < بیٹھوں > < کریندے > < گالھ > < دی > دیقی
< صد > < وسیم > < آلے > < لیکن > < لکیراں > < دیاں > < شاعری > < تے > < رہ > < تلے > < چھاں > < دی > < روضے >
< دے > < سئیں > < فرید > < اساں > < اج > < جو > < ہے > < ھی > < برکت > < تے > < فیض > < دا > < شاعری > < دی >
< سئیں > < خواجہ > < اے > < ؟ > < کائنی > < سونہاں > < کون > < دا > < شاعری > < دی > < انہاں > < تے > < سئیں >
< مفرید > < وچ > < سروے > < لنگویچ > < ایں > < گئے > < ڈتا > < ناں > < دا > < انڈیا > < آف > < سروے > < لینگویچ
< کوں > < تحقیق > < ایں > < ---- > < کیتن > < صرف > < تے > < تحقیق > < دی > < زبانیں > < سال > 1887 << تقریباً >
< وچ > < ہندستان > < متحدہ > < جنھاں > < ہَن > < محقق > < وڈّے > < بہوں > < ہَن > < وی > < ڈی > < ایچ > < پی > ل
< نا > < نال > < دے > < یافتہ > < تعلیم > < اعلیٰ > < اپ > < ہَن > < گریرسن > < سر > < منظوری > < دی > < نقشے > ایں
< > < سکدے۔ > < ونج > < ڈیکھایا > < کوں > < زبانیں > < پاکستانی > < تمام > < وچ > < ایندے > < سرائیکی > < بشمول >
< وچ > < علاقے > < ایں > < مطابق > < دے > < نقشے > < گئے > < بڑایا > < نال > < تعاون > < دے > < حکومت > < برٹش >
< وچ > < 30ء > < ہِن > < شامل > < زبانیں > < گجراتی > < بلوچی > < سندھی > < سرائیکی > < وچ > < نقشے > تانی
< پاکسـ > < ---- > < ---- > < دا > < زبانیں > < پاکستانی > < > < بیٹھوں > < کریندے > < گالھ > < دی > < صدیقی > < وسیم >
< آلے > < لیکن > < لکیراں > < دیاں > < شاعری > < تے > < رہ > < تلے > < چھاں > < دی > < روضے > < دے > < سئیں
< فرید > < اساں > < اج > < جو > < ہے > < ھی > < برکت > < تے > < فیض > < دا > < شاعری > < دی > < سئیں > اجہ
< خو > < اے > < ؟ > < کائنی > < سونہاں > < کون > < دا > < شاعری > < دی > < انہاں > < تے > < سئیں > < مفرید > < وچ
< سروے > < لنگویچ > < ایں > < گئے > < ڈتا > < ناں > < دا > < انڈیا > < آف > < سروے > < لینگویچ > < کوں > تحقیق
< ایں > < ---- > < کیتن > < صرف > < تے > < تحقیق > < دی > < زبانیں > < سال > 1887 < تقریباً > < وچ > ہندستان
< متحدہ > < جنھاں > < ہَن > < محقق > < وڈّے > < بہوں > < ہَن > < وی > < ڈی > < ایچ > < پی > < نال > < نال >
< دے > < یافتہ > < تعلیم > < اعلیٰ > < اپ > < ہَن > < گریرسن > < سر > < منظوری > < دی > < نقشے > < ایں > < سکدے۔
< ونج > < ڈیکھایا > < کوں > < زبانیں > < پاکستانی > < تمام > < وچ > < ایندے > < سرائیکی > < بشمول > < وچ > علاقے
< ایں > < مطابق > < دے > < نقشے > < گئے > < بڑایا > < نال > < تعاون > < دے > < حکومت > < برٹش > < وچ > 3ء
0 <7> < ہِن > < شامل > < زبانیں > < گجراتی > < بلوچی > < سندھی > < سرائیکی > < وچ > < نقشے > < پاکستانی > < ----
< نقشہ > < دا > < زبانیں > < پاکستانی > < > < بیٹھوں > < کریندے > < گالھ > < دی > < صدیقی > < وسیم > < آلے > لیکن
< لکیراں > < دیاں > < شاعری > < تے > < رہ > < تلے > < چھاں > < دی > < روضے > < دے > < سئیں > < فرید > اں
< اسا > < اج > < جو > < ہے > < ھی > < برکت > < تے > < فیض > < دا > < شاعری > < دی > < سئیں > < خواجہ > < اے >
< ؟ > < کائنی > < سونہاں > < کون > < دا > < شاعری > < دی > < انہاں > < تے > < سئیں > < مفرید > < وچ > < سروے >
< لنگویچ > < ایں > < گئے > < ڈتا > < ناں > < دا > < انڈیا > < آف > < سروے > < لینگویچ > < کوں > < تحقیق > < ایں > .
--- < کیتن > < صرف > < تے > < تحقیق > < دی > < زبانیں > < سال > <188 > < تقریباً > < وچ > < ہندستان > < متحدہ >
< جنھاں > < ہَن > < محقق > < وڈّے > < بہوں > < ہَن > < وی > < ڈی > < ایچ > < پی > < نال > < نال > < دے > < یافتہ >
< تعلیم > < اعلیٰ > < اپ > < ہَن > < گریرسن > < سر > < منظوری > < دی > < نقشے > < ایں > < سکدے۔ > < ونج > کھایا
< ڈیـ > < کوں > < زبانیں > < پاکستانی > < تمام > < وچ > < ایندے > < سرائیکی > < بشمول > < وچ > < علاقے > < ایں > ابق
< مط > < دے > < نقشے > < گئے > < بڑایا > < نال > < تعاون > < دے > < حکومت > < برٹش > < وچ > < 87 30ء > < ہِن
< شامل > < زبانیں > < گجراتی > < بلوچی > < سندھی > < سرائیکی > < وچ > < نقشے > < پاکستانی > < ---- > < دا > < دا >
< زبانیں > < پاکستانی > < > < بیٹھوں > < کریندے > < گالھ > < دی > < صدیقی > < وسیم > < آلے > < لیکن > < لکیراں > یاں
< د > < شاعری > < تے > < رہ > < تلے > < چھاں > < دی > < روضے > < دے > < سئیں > < فرید > < اساں > < اج > < جو
< ہے > < ھی > < برکت > < تے > < فیض > < دا > < شاعری > < دی > < سئیں > < خواجہ > < اے > < ؟ > < کائنی >
< سونہاں > < کون > < دا > < شاعری > < دی > < انہاں > < تے > < سئیں > < مفرید > < وچ > < سروے > < لنگویچ > < ایں
< گئے > < ڈتا > < ناں > < دا > < انڈیا > < آف > < سروے > < لینگویچ > < کوں > < تحقیق > < ایں > < ---- > < کیتن > ف
< صر > < تے > < تحقیق > < دی > < زبانیں > < سال > 18 << تقریباً > < وچ > < ہندستان > < متحدہ > < جنھاں > < ہَن
< محقق > < وڈّے > < بہوں > < ہَن > < وی > < ڈی > < ایچ > < پی > < نال > < نال > < دے > < یافتہ > < تعلیم > < اعلیٰ
< اپ > < ہَن > < گریرسن > < سر > < منظوری > < دی > < نقشے > < ایں > < سکدے۔ > < ونج > < ڈیکھایا > < کوں > یں
< زبانـ > < پاکستانی > < تمام > < وچ > < ایندے > < سرائیکی > < بشمول > < وچ > < علاقے > < ایں > < مطابق > < دے > ے
< نقشـ > < گئے > < بڑایا > < نال > < تعاون > < دے > < حکومت > < برٹش > < وچ > < 30887ء > < ہِن > < شامل > زبانیں
< گجراتی > < بلوچی > < سندھی > < سرائیکی > < وچ > < نقشے > < پاکستانی > < ---- > < زبانیں > < دا > < زبانیں > < >
< بیٹھوں > < کریندے > < گالھ > < دی > < صدیقی > < وسیم > < آلے > < لیکن > < لکیراں > < دیاں > < شاعری > < تے >
< رہ > < تلے > < چھاں > < دی > < روضے > < دے > < سئیں > < فرید > < اساں > < اج > < جو > < ہے > < ھی > برکت

< تے > < فیض > < دا > < شاعری > < دی > < سئیں > < خواجہ > < اے > < ؟ > < کائنی > < سونہاں > < کون > < دا > <
< شاعری > < دی > < انہاں > < تے > < سئیں > < مفرید > < وچ > < سروے > < لنگویچ > < ایں > < ڳئے > < ڈُتا > < ناں > <
< دا > < انڈیا > < آف > < سروے > < لینگویچ > < کوں > < تحقیق > < ایں > < ---- > < کیتن > < صرف > < تے > < تحقیق > <
< دی > < زبانیں > < سال > < 10 > < تقریباً > < وچ > < ہندستان > < متحدہ > < جنھاں > < ھَن > < محقق > < وڈے > < بہوں >
< ھَن > < وی > < ڈی > < ایچ > < پی > < نال > < نال > < دے > < یافتہ > < تعلیم > < اعلیٰ > < اپ > < ھَن > < گریرسن >
< سر > < منظوری > < دی > < نقشے > < ایں > < سکدے۔ > < ونج > < ڈیکھایا > < کوں > < زبانیں > < پاکستانی > < تمام > <
وچ > < ایندے > < سرائیکی > < بشمول > < وچ > < علاقے > < ایں > < مطابق > < دے > < نقشے > < ڳئے > < بڑایا > < نال
< تعاون > < دے > < حکومت > < برٹش > < وچ > < 31887ء > < ھِن > < شامل > < d زبانیں > < دا > # NG # > <
پاکستانی > < ---- > < پاکستانی > # NG # > < نقشے # NG # > < وچ > <N> # > < سرائیکی <N> # > < سندھی > # P #
< بلوچی <N> # > < گجراتی # NG # > > > < ھِن > < تعلیم > < الصّوت > < دیاں > < سئیں > < کریندے > < وے
< سر > < مفرید > < بیٹھوں > < ایچ > < زبانیں > < نقشہ > < ھَن > < ھِن > < لیکن > < اے > < وڈے > < برٹش
< انڈیا > < سونہاں > < ڈُتا > < تے > < فرید > < سر > < اپ > < ہندستان > < بشمول > < تعاون > < چھاں >
< حکومت > < لنگویچ > < محقق > < ھے > < نقشے > < پی > < بڑایا > < وسیم > < کائنی > < تقریباً > < دی >
< بہوں > < وی > < اساں > < کوں > < ڳئے > < اعلیٰ > < دا > < گجراتی > < لینگویچ > < وچ > < صرف >
---- > < ڈی > < برکت > < ھی > < تمام > < ایندے > < روضے > < کون > < جو > < نال > < منظوری > < یتن
ک > < آلے > < سکدے۔ > < سرائیکی > < ڈیکھایا > < دے > < ؟ > < مطابق > < سال > < بلوچی > < ء > <<
انہ > < یافتہ > < ناں > < جنھاں > < شاعری > < ایں > < پاکستانی > < صدیقی > < شامل1887 > < فیض > < خواہاں
> < متحدہ > < اج > < گریرسن > < تحقیق > < گالھ > < ونج > < لکیراں > < ائے > < رہ > < تلے > < جلی > <
آف > < > < شامل > < فیض > < تعلیم > < الصّوت > < دیاں > < سئیں > < کریندے > < سروے > < مفرید > <
بیٹھوں > < ایچ > < زبانیں > < نقشہ > < ھَن > < ھِن > < لیکن > < اے > < وڈے > < برٹش > < انڈیا > < سونہاں
> < ڈُتا > < تے > < فرید > < سر > < اپ > < ہندستان > < بشمول > < تعاون > < چھاں > < حکومت > < لنگویچ
> < محقق > < ھے > < نقشے > < پی > < بڑایا > < وسیم > < کائنی > < تقریباً > < دی > < بہوں > < وی > <
اساں > < کوں > < ڳئے > < اعلیٰ > < دا > < گجراتی > < لینگویچ > < وچ > < صرف > < ---- > < ڈی > < کت
بر > < ھی > < تمام > < ایندے > < روضے > < کون > < جو > < نال > < منظوری > < کیتن > < آلے > < دے۔
سک > < سرائیکی > < ڈیکھایا > < دے > < ؟ > < مطابق > < سال > < بلوچی > < 30ء > < سندھی > < انہاجہ >
< یافتہ > < ناں > < جنھاں > < شاعری > < > < بیٹھوں > < کریندے > < گالھ > < دی > < صدیقی > < وسیم > < آلے >
< لیکن > < لکیراں > < دیاں > < شاعری > < تے > < رہ > < تلے > < چھاں > < دی > < روضے > < دے > < سئیں > < فرید >
< اساں > < اج > < جو > < ھے > < ھی > < برکت > < تے > < فیض > < دا > < شاعری > < دی > < سئیں > < خواجہ > <
اے > < ؟ > < کائنی > < سونہاں > < کون > < دا > < شاعری > < دی > < انہاں > < تے > < سئیں > < مفرید > < وچ > < وے
سر > < لنگویچ > < ایں > < ڳئے > < ڈُتا > < ناں > < دا > < انڈیا > < آف > < سروے > < لینگویچ > < کوں > < تحقیق > < ایں
> < ---- > < کیتن > < صرف > < تے > < تحقیق > < دی > < زبانیں > < سال > < 1887 > < علاقے > < ں > < ہندستان > <
متحدہ > < جنھاں > < ھَن > < محقق > < وڈے > < بہوں > < ھَن > < وی > < ڈی > < ایچ > < پی > < نال > < نال > < دے >
یافتہ > < تعلیم > < اعلیٰ > < اپ > < ھَن > < گریرسن > < سر > < منظوری > < دی > < نقشے > < ایں > < سکدے۔ > < ونج
> < ڈیکھایا > < کوں > < زبانیں > < پاکستانی > < تمام > < وچ > < ایندے > < سرائیکی > < بشمول > < وچ > < علاقے > < ایں
> < مطابق > < دے > < نقشے > < ڳئے > < بڑایا > < نال > < تعاون > < دے > < حکومت > < برٹش > < وچ > < 30ء > <
سندھی > < وچ > < زبانیں > < گجراتی > < بلوچی > < سندھی > < ایں > < پاکستانی > < صدیقی > < 1887 > < تقریباً > <
خواجہ > < متحدہ > < اج > < گریرسن > < تحقیق > < گالھ > < ونج > < لکیراں > < ائے > < رہ > < تلے > < لی
جہ > < آف > < > < > < بیٹھوں > < کریندے > < گالھ > < دی > < صدیقی > < وسیم > < آلے > < لیکن > < لکیراں > < دیاں > <
شاعری > < تے > < رہ > < تلے > < چھاں > < دی > < روضے > < دے > < سئیں > < فرید > < اساں > < اج > < جو > < ے
ھ > < ھی > < برکت > < تے > < فیض > < دا > < شاعری > < دی > < سئیں > < خواجہ > < اے > < ؟ > < کائنی > < سونہاں
> < کون > < دا > < شاعری > < دی > < انہاں > < تے > < سئیں > < مفرید > < وچ > < سروے > < لنگویچ > < ایں > < ڳئے
> < ڈُتا > < ناں > < دا > < انڈیا > < آف > < سروے > < لینگویچ > < کوں > < تحقیق > < ایں > < ---- > < کیتن > < صرف >
< تے > < تحقیق > < دی > < زبانیں > < سال > < 30 > < ھِن > < وچ شامل > < ہندستان > < متحدہ > < جنھاں > < ھَن > < قق
محد > < وڈے > < بہوں > < ھَن > < وی > < ڈی > < ایچ > < پی > < نال > < نال > < دے > < یافتہ > < تعلیم > < اعلیٰ > < پ
ا > < ھَن > < گریرسن > < سر > < منظوری > < دی > < نقشے > < ایں > < سکدے۔ > < ونج > < ڈیکھایا > < کوں > < زبانیں
> < پاکستانی > < تمام > < وچ > < ایندے > < سرائیکی > < بشمول > < وچ > < علاقے > < ایں > < مطابق > < دے > < نقشے
< ڳئے > < بڑایا > < نال > < تعاون > < دے > < حکومت > < برٹش > < وچ > < 30ء > < علاقے > < > < > < زبانیں > <
گجراتی > < بلوچی > < سندھی > < سرائیکی > < وچ > < نقشے > < پاکستانی > < ---- > < سرائیکی > < دا > < زبانیں > < ستانی
پاک > < > < بیٹھوں > < کریندے > < گالھ > < دی > < صدیقی > < وسیم > < آلے > < لیکن > < لکیراں > < دیاں > < شاعری >
< تے > < رہ > < تلے > < چھاں > < دی > < روضے > < دے > < سئیں > < فرید > < اساں > < اج > < جو > < ھے > < ھی
< برکت > < تے > < فیض > < دا > < شاعری > < دی > < سئیں > < خواجہ > < اے > < ؟ > < کائنی > < سونہاں > < کون
< دا > < شاعری > < دی > < انہاں > < تے > < سئیں > < مفرید > < وچ > < سروے > < لنگویچ > < ایں > < ڳئے > < ڈُتا >
< ناں > < دا > < انڈیا > < آف > < سروے > < لینگویچ > < کوں > < تحقیق > < ایں > < ---- > < کیتن > < صرف > < تے >
< تحقیق > < دی > < زبانیں > < سال > < 1887 > < تقریباً > < وچ شا > < ہندستان > < متحدہ > < جنھاں > < ھَن > < محقق > < وڈے
< بہوں > < ھَن > < وی > < ڈی > < ایچ > < پی > < نال > < نال > < دے > < یافتہ > < تعلیم > < اعلیٰ > < اپ > < ھَن >

< گریرسن > < سر > < منظوری > < دی > < نقشے > < ایں > < سکدے۔ > < ونج > < ڈیکھایا > < کوں > < زبانیں > < پاکستانی
> < تمام > < وچ > < ایندے > < سرائیکی > < بشمول > < وچ > < علاقے > < ایں > < مطابق > < دے > < نقشے > < ڳئے > <
بڑایا > < نال > < تعاون > < دے > < حکومت > < برٹش > < وچ > < 30ء > < ہِن > < مل> <شہ > < زبانیں > < گجراتی > <
بلوچی > < سندھی > < سرائیکی > < وچ > < نقشے > < پاکستانی > < ---- > < وچ > < دا > < زبانیں > < پاکستانی > < > < ٹھوں
بی > < کریندے > <گالھ > < دی > < صدیقی > < وسیم > < آلے > < لیکن > < لکیراں > < دیاں > < شاعری > < تے > < رہ >
تلے > < چھاں > < دی > < روضے > < دے > < سئیں > < فرید > < اساں > < اج > < جو > < ھے > < ھی > < برکت > <
تے > < فیض > < دا > < شاعری > < دی > < سئیں > < خواجہ > < اے > < ؟ > < کائنی > < سونہاں > < کون > < دا > < عری
شا > < دی > < انہاں > < تے > < سئیں > < مفرید > < وچ > < سروے > < لنگویچ > < ایں > < ڳئے > < ڈتا > < ناں > < دا >
< انڈیا > < آف > < سروے > < لینگویچ > < کوں > < تحقیق > < ایں > < ---- > < کیتن > < صرف > < تے > < تحقیق > < دی >
< زبانیں > < سال > < 1887 > < تقریباً امہ< وچ > < ہندستان > < متحدہ > < جنھاں > < ہَن > < محقق > < وڈے > < بہوں > < َن
ھ > < وی > < ڈی > < ایچ > < پی > < نال > < نال > < دے > < یافتہ > < تعلیم > < اعلٰی > < اپ > < ہَن > < گریرسن > < ر
سد > < منظوری > < دی > < نقشے > < ایں > < سکدے۔ > < ونج > < ڈیکھایا > < کوں > < زبانیں > < پاکستانی > < تمام > < وچ
> < ایندے > < سرائیکی > < بشمول > < وچ > < علاقے > < ایں > < مطابق > < دے > < نقشے > < ڳئے > < بڑایا > < نال >
< تعاون > < دے > < حکومت > < برٹش > < وچ > < 30ء > < ہِن > < ل> شا > < زبانیں > < گجراتی > < بلوچی > < سندھی
> < سرائیکی > < وچ > < نقشے > < پاکستانی > < ---- > < نقشے > < دا > < زبانیں > < پاکستانی > < > < بیٹھوں > < کریندے
> <گالھ > < دی > < صدیقی > < وسیم > < آلے > < لیکن > < لکیراں > < دیاں > < شاعری > < تے > < رہ > < تلے > < چھاں
> < دی > < روضے > < دے > < سئیں > < فرید > < اساں > < اج > < جو > < ھے > < ھی > < برکت > < تے > < فیض >
< دا > < شاعری > < دی > < سئیں > < خواجہ > < اے > < ؟ > < کائنی > < سونہاں > < کون > < دا > < شاعری > < دی > <
انہاں > < تے > < سئیں > < مفرید > < وچ > < سروے > < لنگویچ > < ایں > < ڳئے > < ڈتا > < ناں > < دا > < انڈیا > < آف >
< سروے > < لینگویچ > < کوں > < تحقیق > < ایں > < ---- > < کیتن > < صرف > < تے > < تحقیق > < دی > < زبانیں > < ال
سد > < 1887 > < ملتقریباً < وچ > < ہندستان > < متحدہ > < جنھاں > < ہَن > < محقق > < وڈے > < بہوں > < ہَن > < وی > <
ڈی > < ایچ > < پی > < نال > < نال > < دے > < یافتہ > < تعلیم > < اعلٰی > < اپ > < ہَن > < گریرسن > < سر > < منظوری
> < دی > < نقشے > < ایں > < سکدے۔ > < ونج > < ڈیکھایا > < کوں > < زبانیں > < پاکستانی > < تمام > < وچ > < ایندے > <
سرائیکی > < بشمول > < وچ > < علاقے > < ایں > < مطابق > < دے > < نقشے > < ڳئے > < بڑایا > < نال > < تعاون > < دے
> < حکومت > < برٹش > < وچ > < 30ء > < ہِن > < ت >شام > < زبانیں > < گجراتی > < بلوچی > < سندھی > < سرائیکی >
< وچ > < نقشے > < پاکستانی > < ---- > < پاکستانی > < دا > < زبانیں > < پاکستانی > < > < بیٹھوں > < کریندے > < گالھ > <
دی > < صدیقی > < وسیم > < آلے > < لیکن > < لکیراں > < دیاں > < شاعری > < تے > < رہ > < تلے > < چھاں > < دی > <
روضے > < دے > < سئیں > < فرید > < اساں > < اج > < جو > < ھے > < ھی > < برکت > < تے > < فیض > < دا > < اعری
شد > < دی > < سئیں > < خواجہ > < اے > < ؟ > < کائنی > < سونہاں > < کون > < دا > < شاعری > < دی > < انہاں > < تے >
< سئیں > < مفرید > < وچ > < سروے > < لنگویچ > < ایں > < ڳئے > < ڈتا > < ناں > < دا > < انڈیا > < آف > < سروے > <
لینگویچ > < کوں > < تحقیق > < ایں > < ---- > < کیتن > < صرف > < تے > < تحقیق > < دی > < زبانیں > < سال > < 1887 >
< قریباً ل< وچ > < ہندستان > < متحدہ > < جنھاں > < ہَن > < محقق > < وڈے > < بہوں > < ہَن > < وی > < ڈی > < ایچ >
< پی > < نال > < نال > < دے > < یافتہ > < تعلیم > < اعلٰی > < اپ > < ہَن > < گریرسن > < سر > < منظوری > < دی > <
نقشے > < ایں > < سکدے۔ > < ونج > < ڈیکھایا > < کوں > < زبانیں > < پاکستانی > < تمام > < وچ > < ایندے > < سرائیکی > <
بشمول > < وچ > < علاقے > < ایں > < مطابق > < دے > < نقشے > < ڳئے > < بڑایا > < نال > < تعاون > < دے > < حکومت
> < برٹش > < وچ > < 30ء > < ہِن > < تق >شامل > < زبانیں > < گجراتی > < بلوچی > < سندھی > < سرائیکی > < وچ > < ے
نقش > < پاکستانی > < ---- > < ---- > < دا > < زبانیں > < پاکستانی > < > < بیٹھوں > < کریندے > < گالھ > < دی > < صدیقی >
وسیم > < آلے > < لیکن > < لکیراں > < دیاں > < شاعری > < تے > < رہ > < تلے > < چھاں > < دی > < روضے > < دے >
< سئیں > < فرید > < اساں > < اج > < جو > < ھے > < ھی > < برکت > < تے > < فیض > < دا > < شاعری > < دی > < ئیں
سد > < خواجہ > < اے > < ؟ > < کائنی > < سونہاں > < کون > < دا > < شاعری > < دی > < انہاں > < تے > < سئیں > < مفرید
> < وچ > < سروے > < لنگویچ > < ایں > < ڳئے > < ڈتا > < ناں > < دا > < انڈیا > < آف > < سروے > < لینگویچ > < کوں
> < تحقیق > < ایں > < ---- > < کیتن > < صرف > < تے > < تحقیق > < دی > < زبانیں > < سال > < 1887 > < ریباً< > < وچ
> < ہندستان > < متحدہ > < جنھاں > < ہَن > < محقق > < وڈے > < بہوں > < ہَن > < وی > < ڈی > < ایچ > < پی > < نال >
< نال > < دے > < یافتہ > < تعلیم > < اعلٰی > < اپ > < ہَن > < گریرسن > < سر > < منظوری > < دی > < نقشے > < ایں > <
سکدے۔ > < ونج > < ڈیکھایا > < کوں > < زبانیں > < پاکستانی > < تمام > < وچ > < ایندے > < سرائیکی > < بشمول > < وچ >
< علاقے > < ایں > < مطابق > < دے > < نقشے > < ڳئے > < بڑایا > < نال > < تعاون > < دے > < حکومت > < برٹش > < وچ
> < 30ء > < ہِن > تقر شامل > < زبانیں > < گجراتی > < بلوچی > < سندھی > < سرائیکی > < وچ > < نقشے > < پاکستانی >
---- > < نقشہ > < دا > < زبانیں > < پاکستانی > < > < بیٹھوں > < کریندے > < گالھ > < دی > < صدیقی > < وسیم > < آلے >
< لیکن > < لکیراں > < دیاں > < شاعری > < تے > < رہ > < تلے > < چھاں > < دی > < روضے > < دے > < سئیں > < فرید
> < اساں > < اج > < جو > < ھے > < ھی > < برکت > < تے > < فیض > < دا > < شاعری > < دی > < سئیں > < خواجہ >
< اے > < ؟ > < کائنی > < سونہاں > < کون > < دا > < شاعری > < دی > < انہاں > < تے > < سئیں > < مفرید > < وچ > < ے
سرو > < لنگویچ > < ایں > < ڳئے > < ڈتا > < ناں > < دا > < انڈیا > < آف > < سروے > < لینگویچ > < کوں > < تحقیق > < ں
ای > < ---- > < کیتن > < صرف > < تے > < تحقیق > < دی > < زبانیں > < سال > < 1887 > < یباً > < > < وچ > < ہندستان > < ہ
متحد > < جنھاں > < ہَن > < محقق > < وڈے > < بہوں > < ہَن > < وی > < ڈی > < ایچ > < پی > < نال > < نال > < دے > <
یافتہ > < تعلیم > < اعلٰی > < اپ > < ہَن > < گریرسن > < سر > < منظوری > < دی > < نقشے > < ایں > < سکدے۔ > < ونج >

ایں > < علاقے > < وچ > < بشمول > < سرائیکی > < ایندے > < وچ > < تمام > < پاکستانی > < زبانیں > < کوں > < ڈیکھایا >
ںِ > < 30ء > < وچ > < برٹش > < حکومت > < دے > < تعاون > < نال > < بڑایا > < گِئے > < نقشے > < دے > < مطابق > <
> < دا > < --- > < پاکستانی > < نقشے > < وچ > < سرائیکی > < سندھی > < بلوچی > < گجراتی > < زبانیں > < شامل >تقریہ < ھ
لکیراں > < لیکن > < آلے > < وسیم > < صدیقی > < دی > < گالھ > < کریندے > < بیٹھوں > < > < پاکستانی > < زبانیں > < دا
< اج > < اساں > < فرید > < سئیں > < دے > < روضے > < دی > < چھاں > < تلے > < رہ > < تے > < شاعری > < دیاں > <
ائنی > < ؟ > < اے > < خواجہ > < سئیں > < دی > < شاعری > < دا > < فیض > < تے > < برکت > < ھی > < ھے > < جو >
> < لنگویچ > < سروے > < وچ > < مفرید > < سئیں > < تے > < انہاں > < دی > < شاعری > < دا > < کون > < سونہاں > < ک
< کیتن > < --- > < ایں > < تحقیق > < کوں > < لینگویچ > < سروے > < آف > < انڈیا > < دا > < ناں > < ڈتا > < گِئے > < ایں
> < جنھاں > < متحدہ > < ہندستان > < وچ > < تد بأ > < 1887 > < سال > < زبانیں > < دی > < تحقیق > < تے > < صرف >
> < تعلیم > < یافتہ > < دے > < نال > < نال > < پی > < ایچ > < ڈی > < وی > < ھَن > < بہوں > < وڈے > < محقق > < ھَن
کوں > < ڈیکھایا > < ونج > < سکدے۔ > < ایں > < نقشے > < دی > < منظوری > < سر > < گریرسن > < ھَن > < اپ > < اعلیٰ
دے > < مطابق > < ایں > < علاقے > < وچ > < بشمول > < سرائیکی > < ایندے > < وچ > < تمام > < پاکستانی > < زبانیں > <
> < شامل > قریبہ< ھِن > < 30ء > < وچ > < برٹش > < حکومت > < دے > < تعاون > < نال > < بڑایا > < گِئے > < نقشے > <
< زبانیں > < دا > < زبانیں > < --- > < پاکستانی > < نقشے > < وچ > < سرائیکی > < سندھی > < بلوچی > < گجراتی > < زبانیں
ے > < شاعری > < دیاں > < لکیراں > < لیکن > < آلے > < وسیم > < صدیقی > < دی > < گالھ > < کریندے > < بیٹھوں > < > <
> < ھی > < ھے > < جو > < اج > < اساں > < فرید > < سئیں > < دے > < روضے > < دی > < چھاں > < تلے > < رہ > < ت
ا > < کون > < سونہاں > < کائنی > < ؟ > < اے > < خواجہ > < سئیں > < دی > < شاعری > < دا > < فیض > < تے > < برکت
اں > < ڈتا > < گِئے > < ایں > < لنگویچ > < سروے > < وچ > < مفرید > < سئیں > < تے > < انہاں > < دی > < شاعری > < د
تحقیق > < تے > < صرف > < کیتن > < --- > < ایں > < تحقیق > < کوں > < لینگویچ > < سروے > < آف > < انڈیا > < دا > < ن
> < وڈے > < محقق > < ھَن > < جنھاں > < متحدہ > < ہندستان > < وچ > < تقً< > < 1887 > < سال > < زبانیں > < دی > <
سن > < ھَن > < اپ > < اعلیٰ > < تعلیم > < یافتہ > < دے > < نال > < نال > < پی > < ایچ > < ڈی > < وی > < ھَن > < بہوں
> < پاکستانی > < زبانیں > < کوں > < ڈیکھایا > < ونج > < سکدے۔ > < ایں > < نقشے > < دی > < منظوری > < سر > < گریر
بڑایا > < گِئے > < نقشے > < دے > < مطابق > < ایں > < علاقے > < وچ > < بشمول > < سرائیکی > < ایندے > < وچ > < تمام
ً > < 1887زبانیں > < شامل > ریبا ھِن > < 30ء > < وچ > < برٹش > < حکومت > < دے > < تعاون > < نال > <
