# Supplementary figures and images for: Mixed Script Identification Using Automated DNN Hyperparameter Optimization
Source: Comput Intell Neurosci. 2021 Dec 10;2021:8415333. doi: 10.1155/2021/8415333 (PMC8683192; doi:10.1155/2021/8415333)

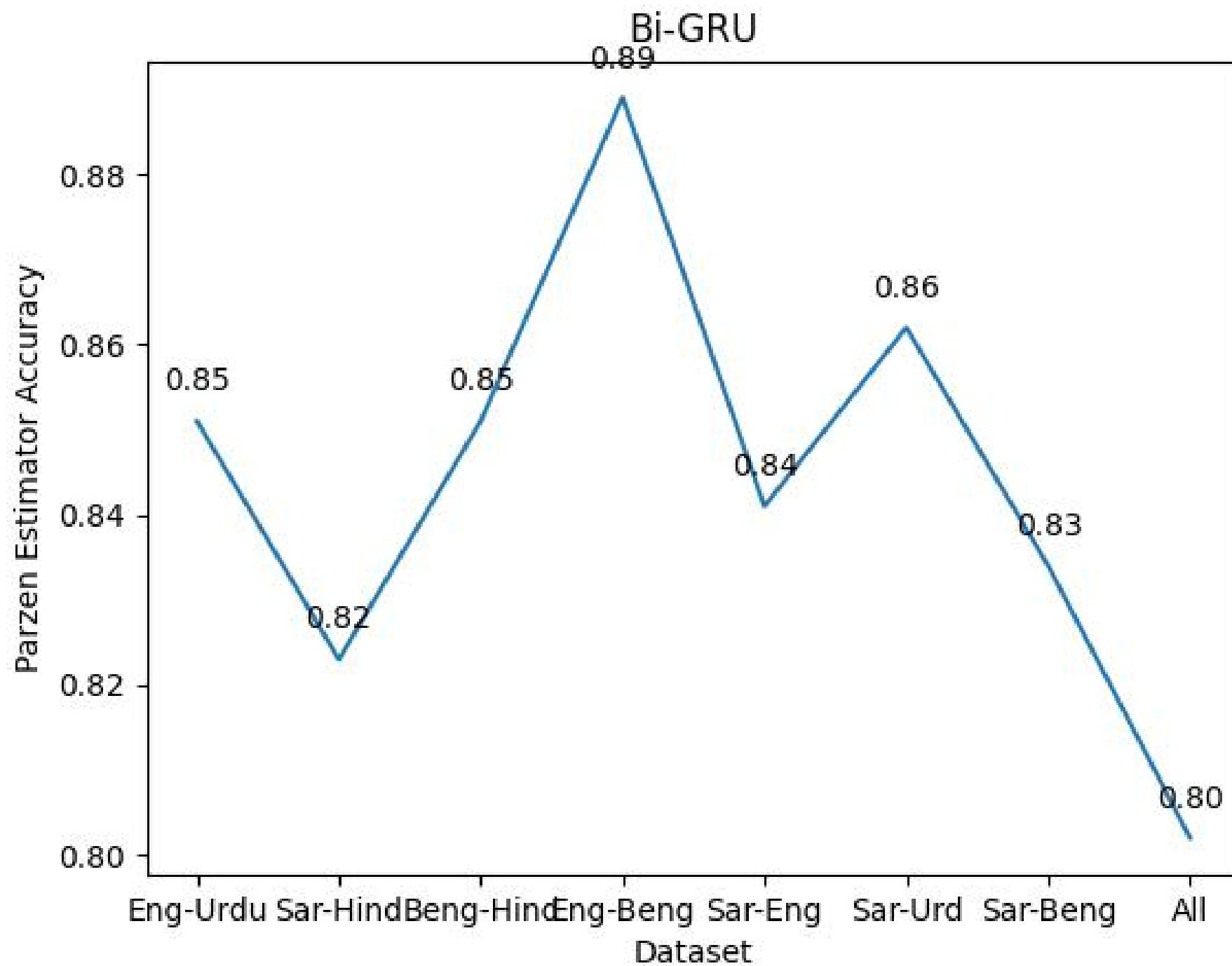

Supplement: Supplementary Materials — (e.g., datasets or results outcomes in the form of graphs) from different stages are provided with the manuscript. The graphs including system training, validation, and the testing outcome of all RNN variants are included in Supplementary Materials. [file 8415333.f1.zip › 8415333.f1/bi gru.pdf]

## Bi-GRU

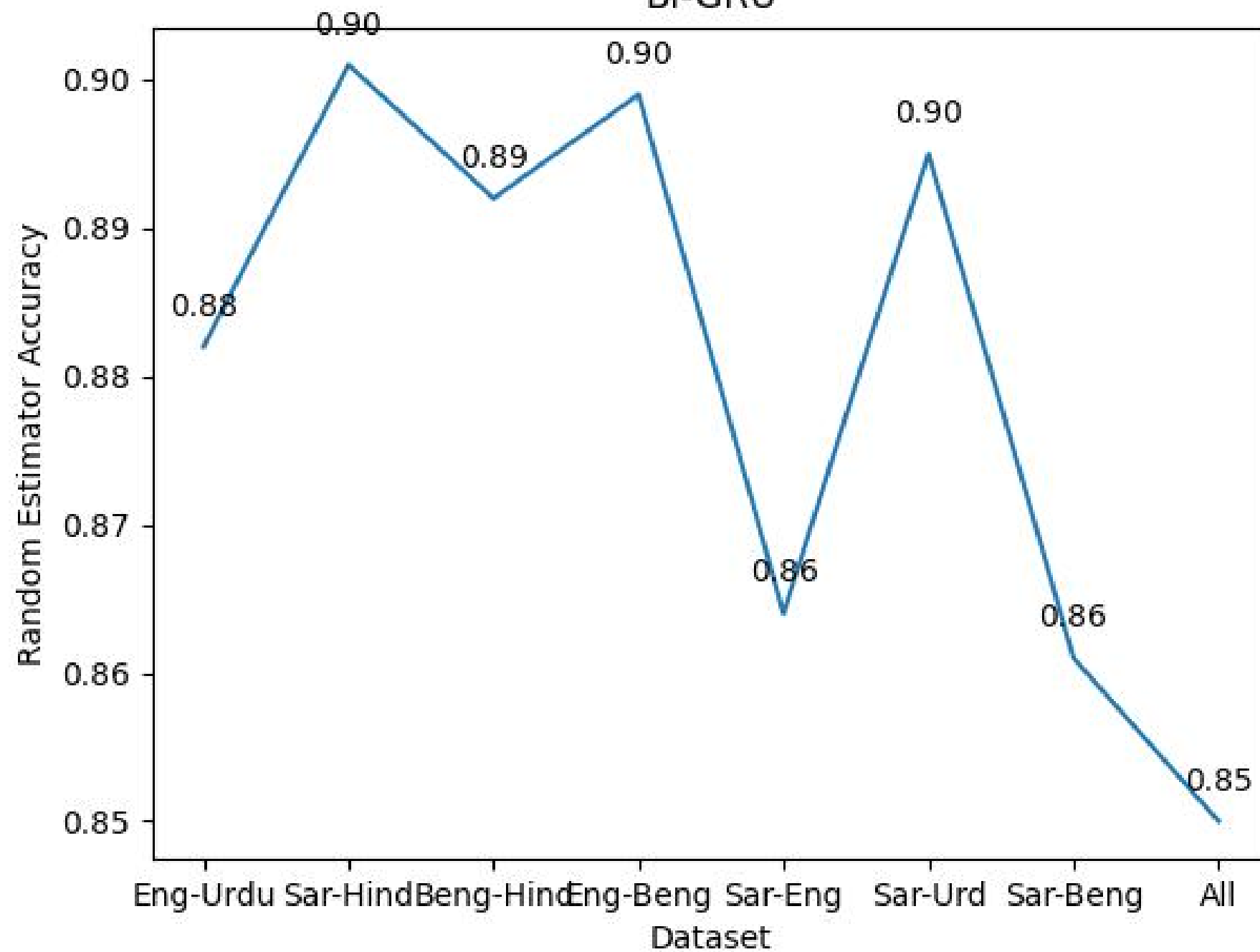

Supplement: Supplementary Materials — (e.g., datasets or results outcomes in the form of graphs) from different stages are provided with the manuscript. The graphs including system training, validation, and the testing outcome of all RNN variants are included in Supplementary Materials. [file 8415333.f1.zip › 8415333.f1/bigrur.pdf]

Bi-LSTM

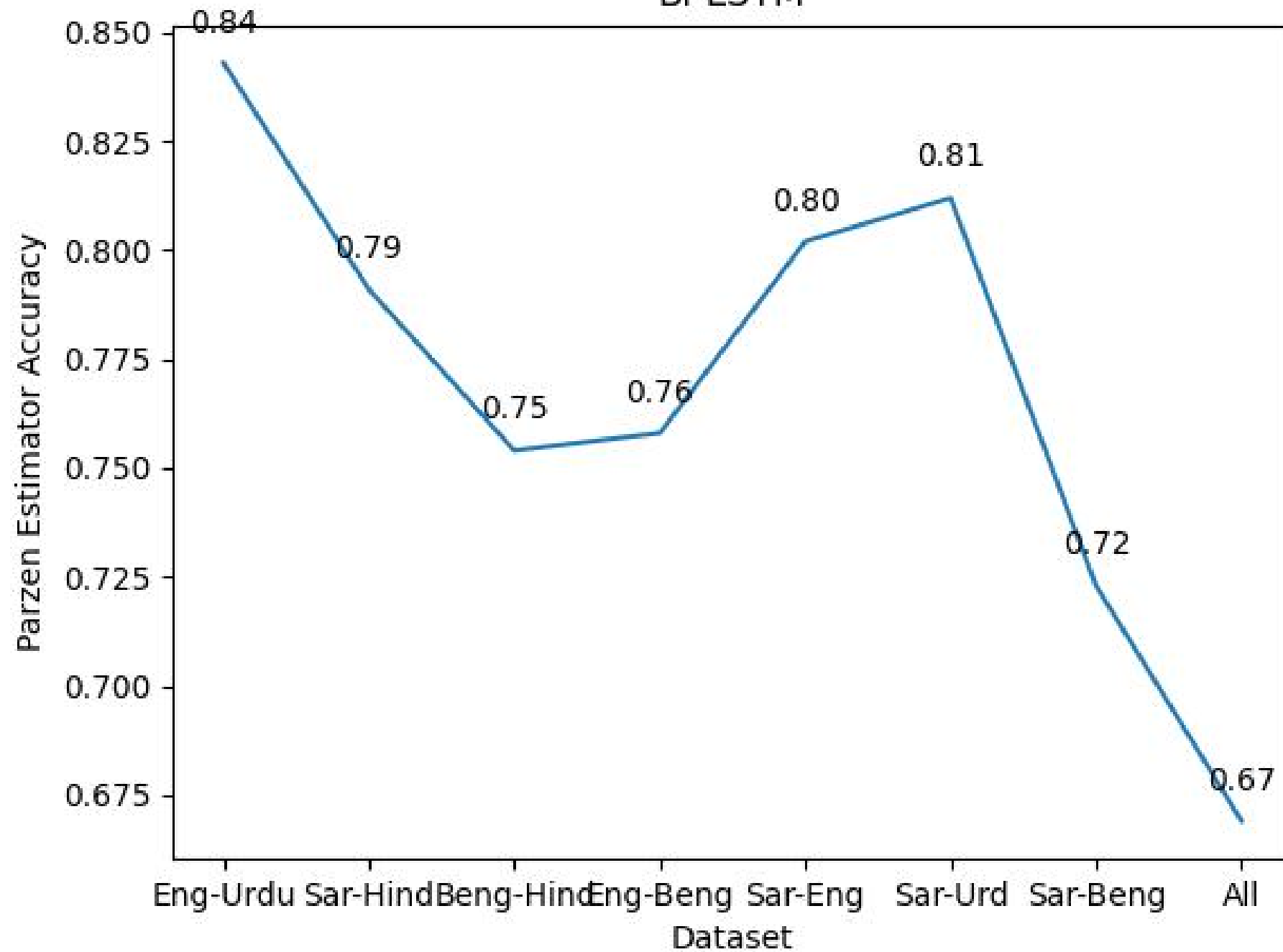

Supplement: Supplementary Materials — (e.g., datasets or results outcomes in the form of graphs) from different stages are provided with the manuscript. The graphs including system training, validation, and the testing outcome of all RNN variants are included in Supplementary Materials. [file 8415333.f1.zip › 8415333.f1/bilstm.pdf]

Bi-LSTM

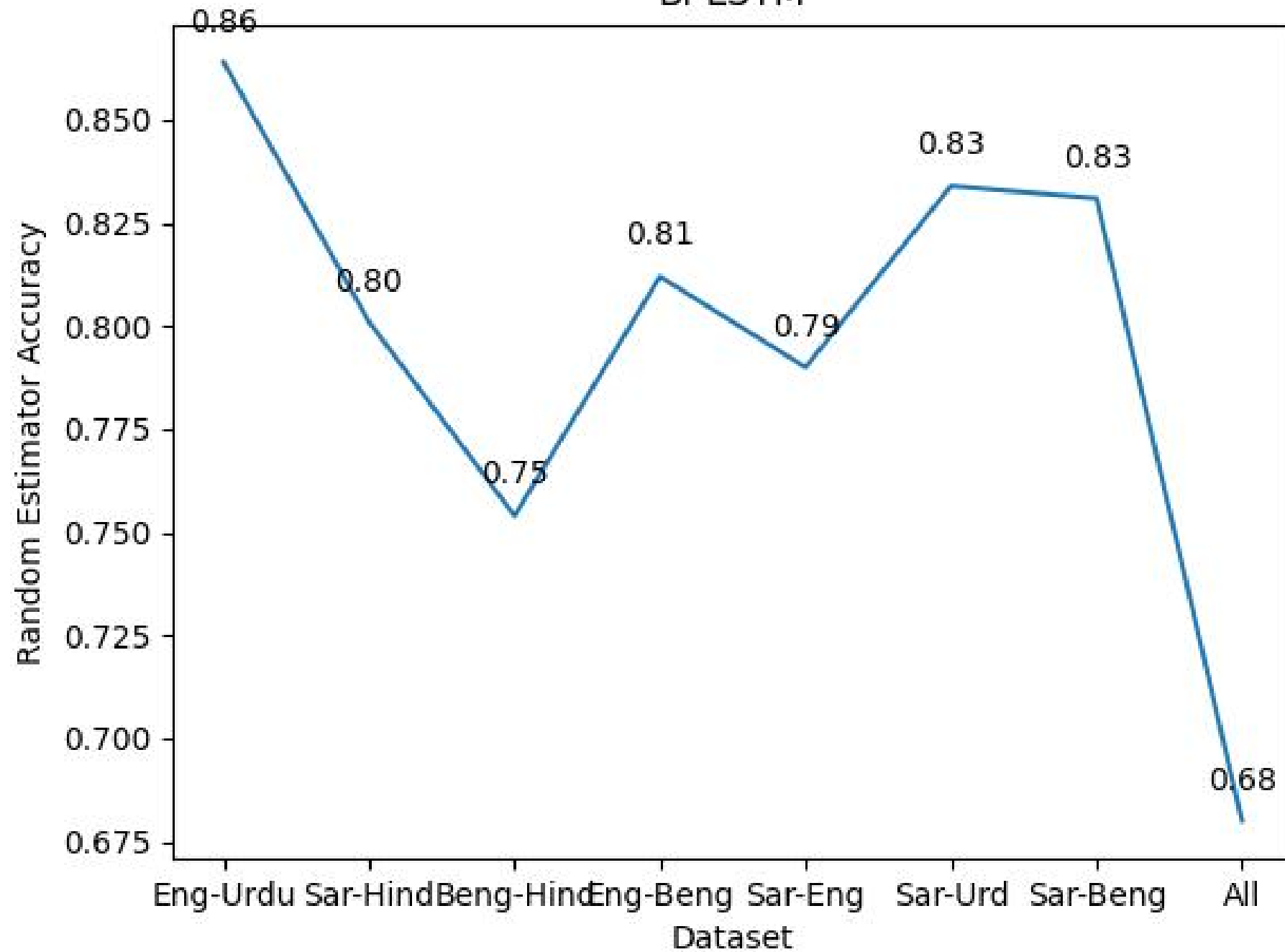

Supplement: Supplementary Materials — (e.g., datasets or results outcomes in the form of graphs) from different stages are provided with the manuscript. The graphs including system training, validation, and the testing outcome of all RNN variants are included in Supplementary Materials. [file 8415333.f1.zip › 8415333.f1/bilstmr.pdf]

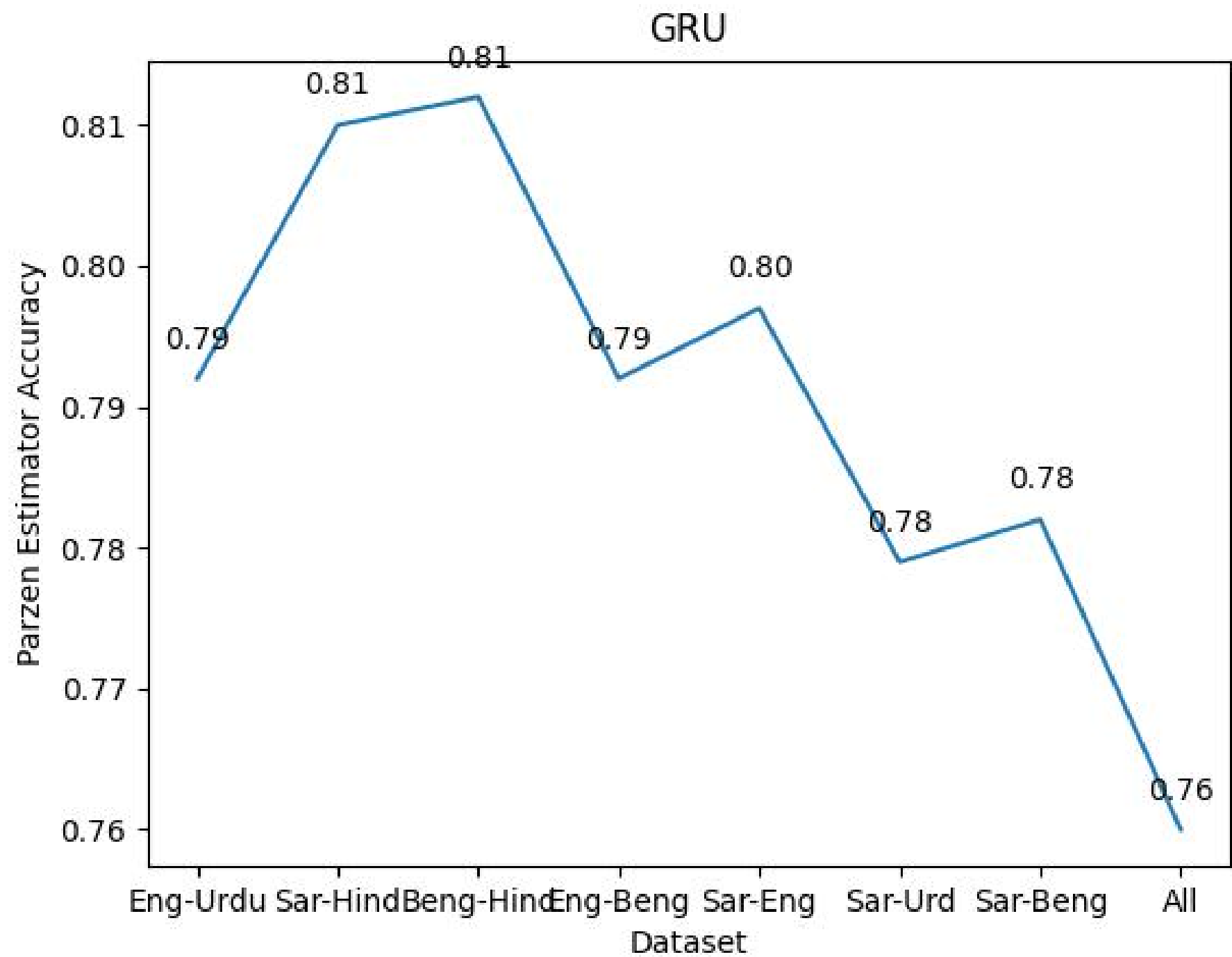

Supplement: Supplementary Materials — (e.g., datasets or results outcomes in the form of graphs) from different stages are provided with the manuscript. The graphs including system training, validation, and the testing outcome of all RNN variants are included in Supplementary Materials. [file 8415333.f1.zip › 8415333.f1/gru.pdf]

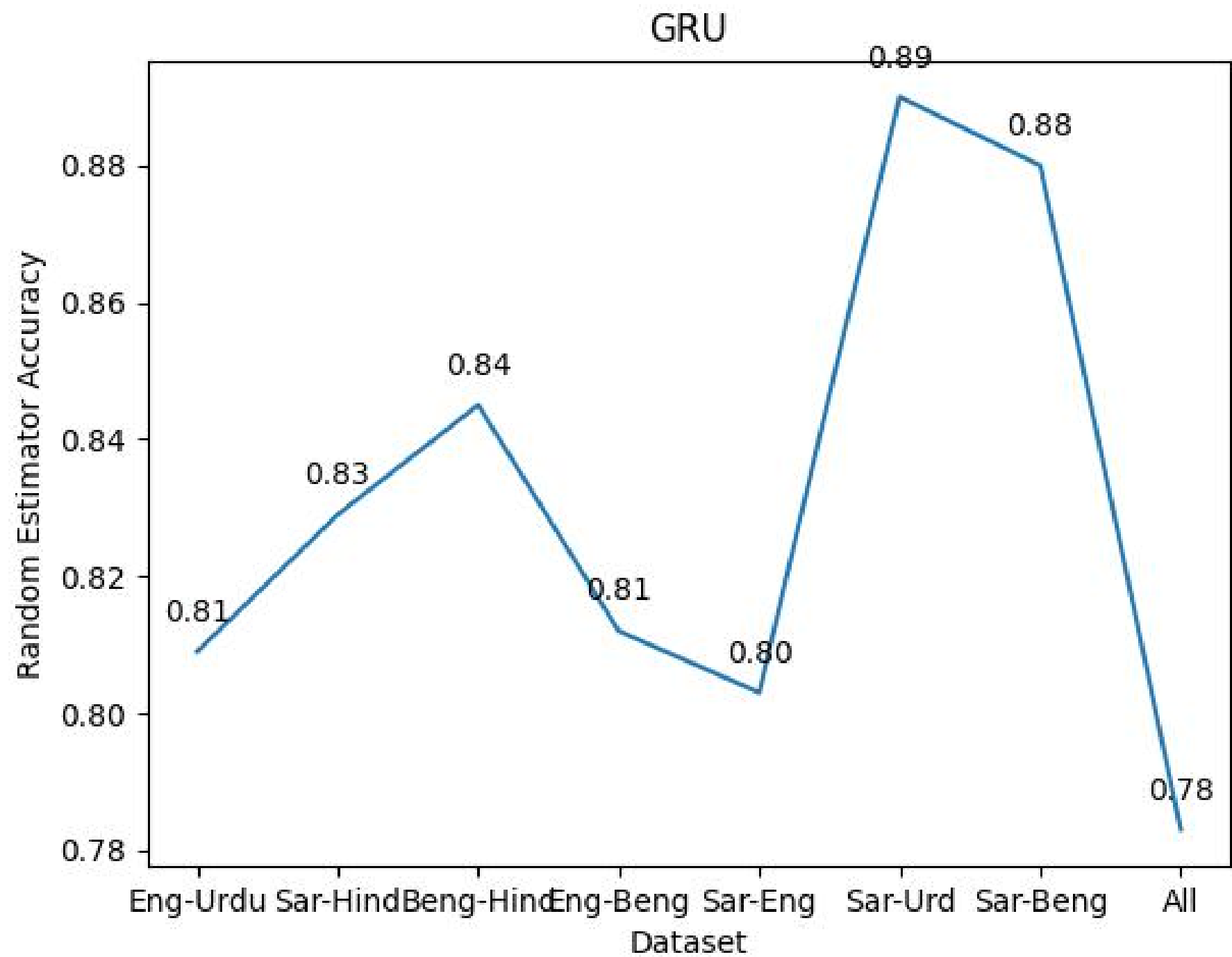

Supplement: Supplementary Materials — (e.g., datasets or results outcomes in the form of graphs) from different stages are provided with the manuscript. The graphs including system training, validation, and the testing outcome of all RNN variants are included in Supplementary Materials. [file 8415333.f1.zip › 8415333.f1/grur.pdf]

## LSTM

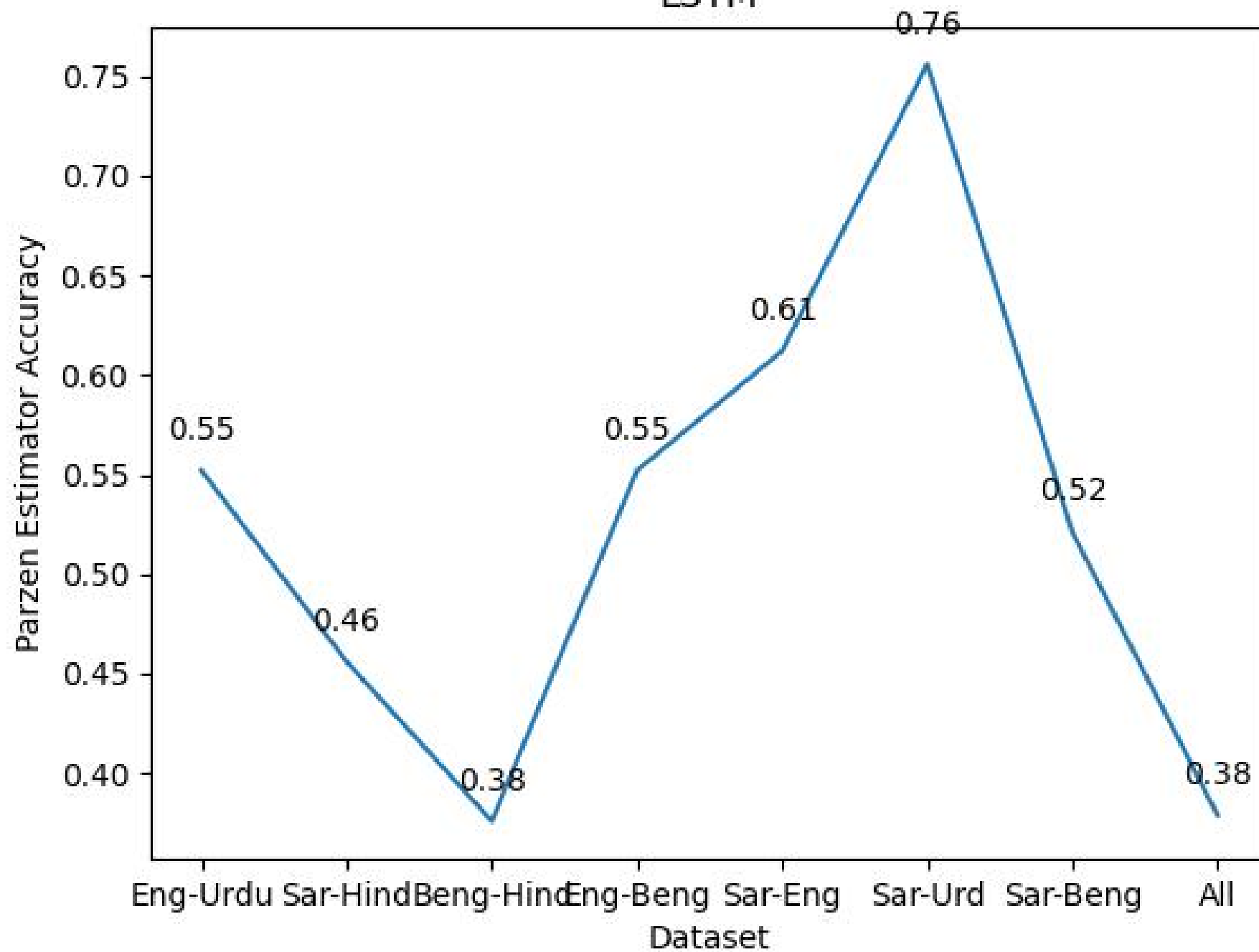

Supplement: Supplementary Materials — (e.g., datasets or results outcomes in the form of graphs) from different stages are provided with the manuscript. The graphs including system training, validation, and the testing outcome of all RNN variants are included in Supplementary Materials. [file 8415333.f1.zip › 8415333.f1/LSTM.pdf]

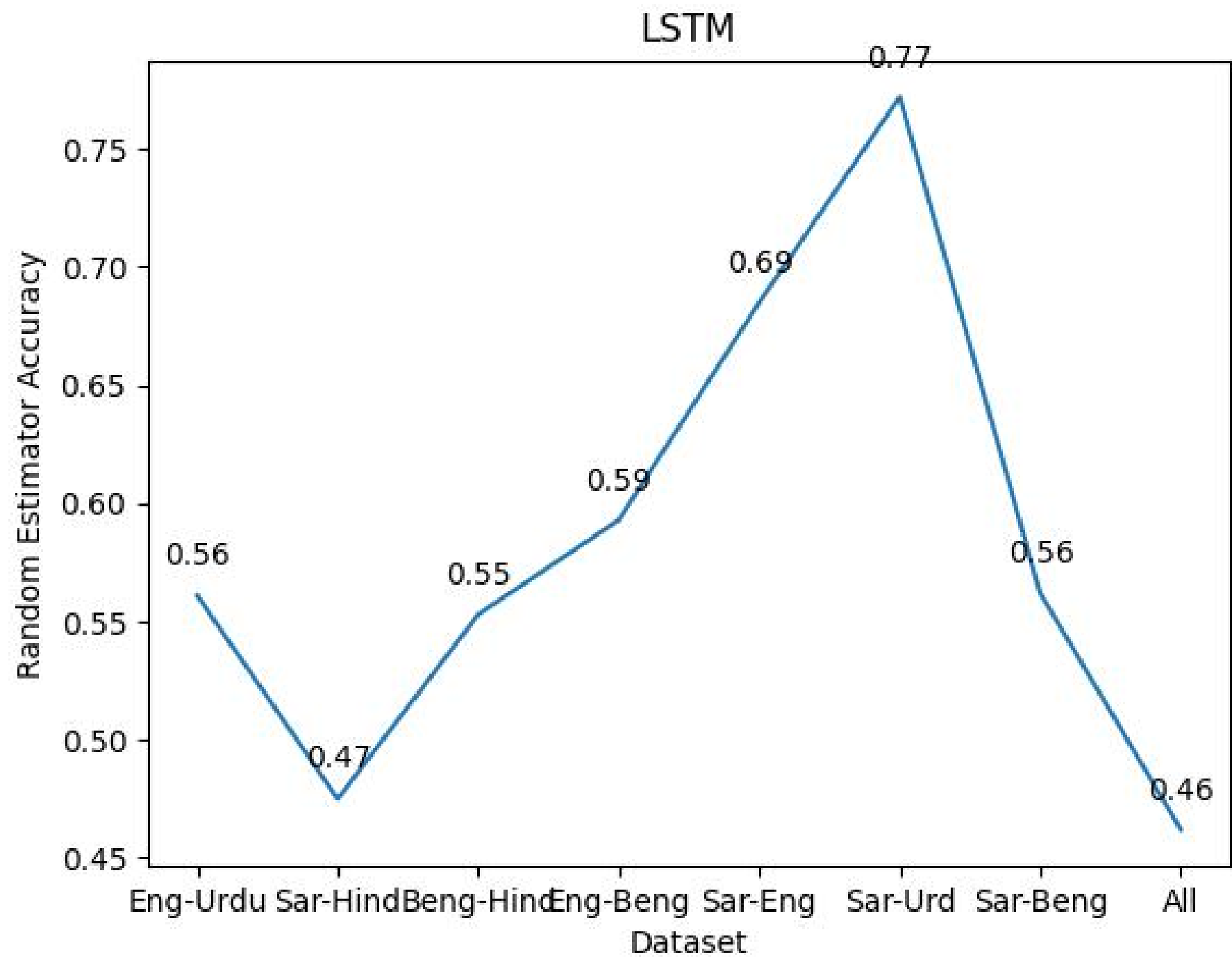

Supplement: Supplementary Materials — (e.g., datasets or results outcomes in the form of graphs) from different stages are provided with the manuscript. The graphs including system training, validation, and the testing outcome of all RNN variants are included in Supplementary Materials. [file 8415333.f1.zip › 8415333.f1/lstmr.pdf]

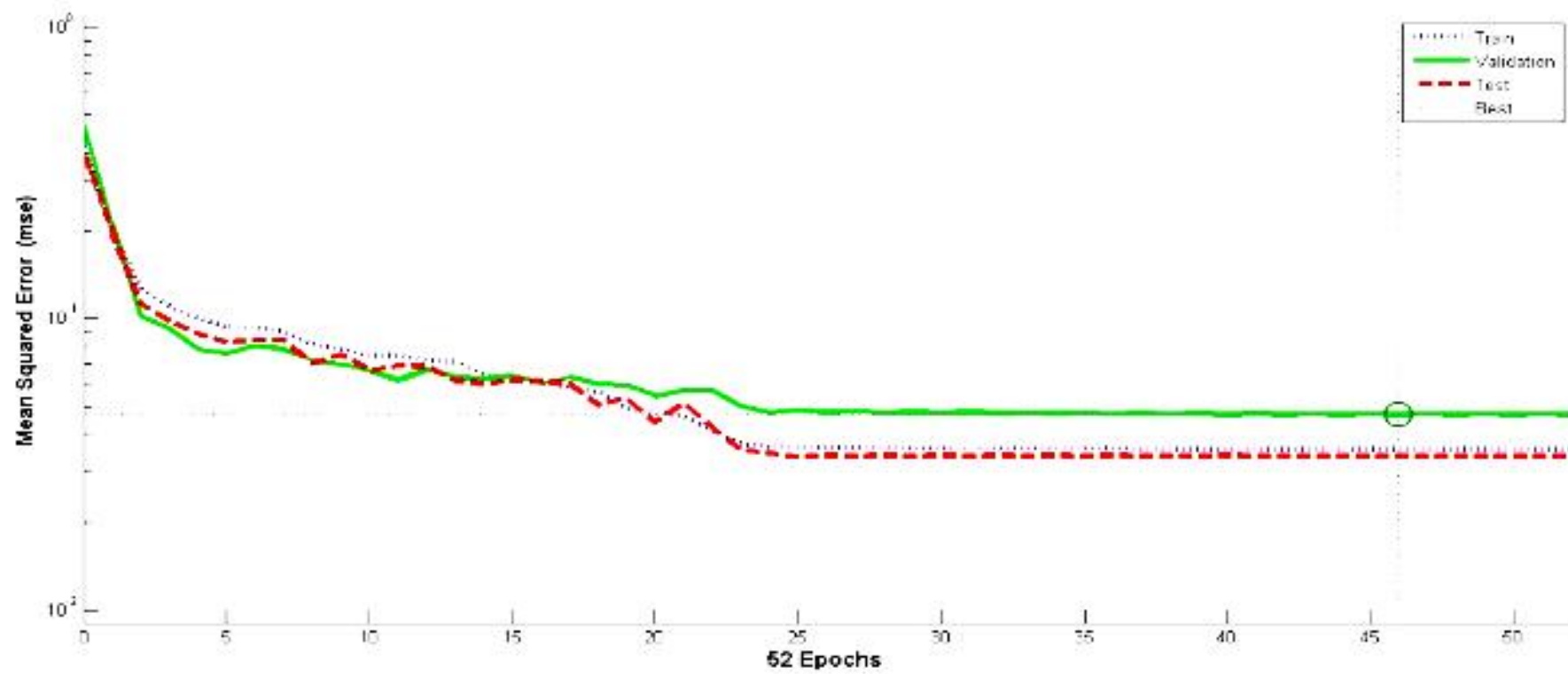

Supplement: Supplementary Materials — (e.g., datasets or results outcomes in the form of graphs) from different stages are provided with the manuscript. The graphs including system training, validation, and the testing outcome of all RNN variants are included in Supplementary Materials. [file 8415333.f1.zip › 8415333.f1/MSE comparison with Training Epochs.pdf]

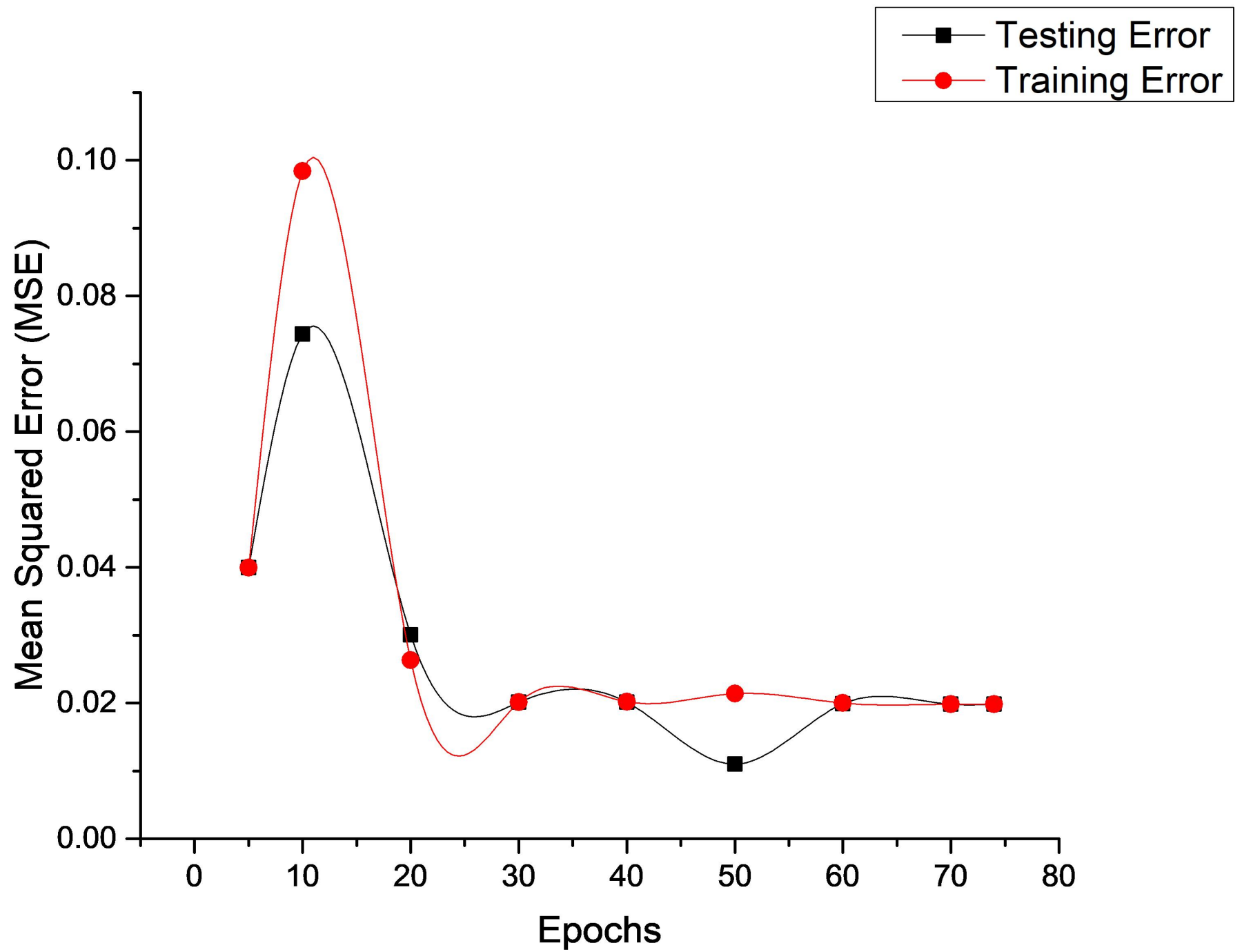

Supplement: Supplementary Materials — (e.g., datasets or results outcomes in the form of graphs) from different stages are provided with the manuscript. The graphs including system training, validation, and the testing outcome of all RNN variants are included in Supplementary Materials. [file 8415333.f1.zip › 8415333.f1/Mse training-epochs.pdf]

## Bi-GRU

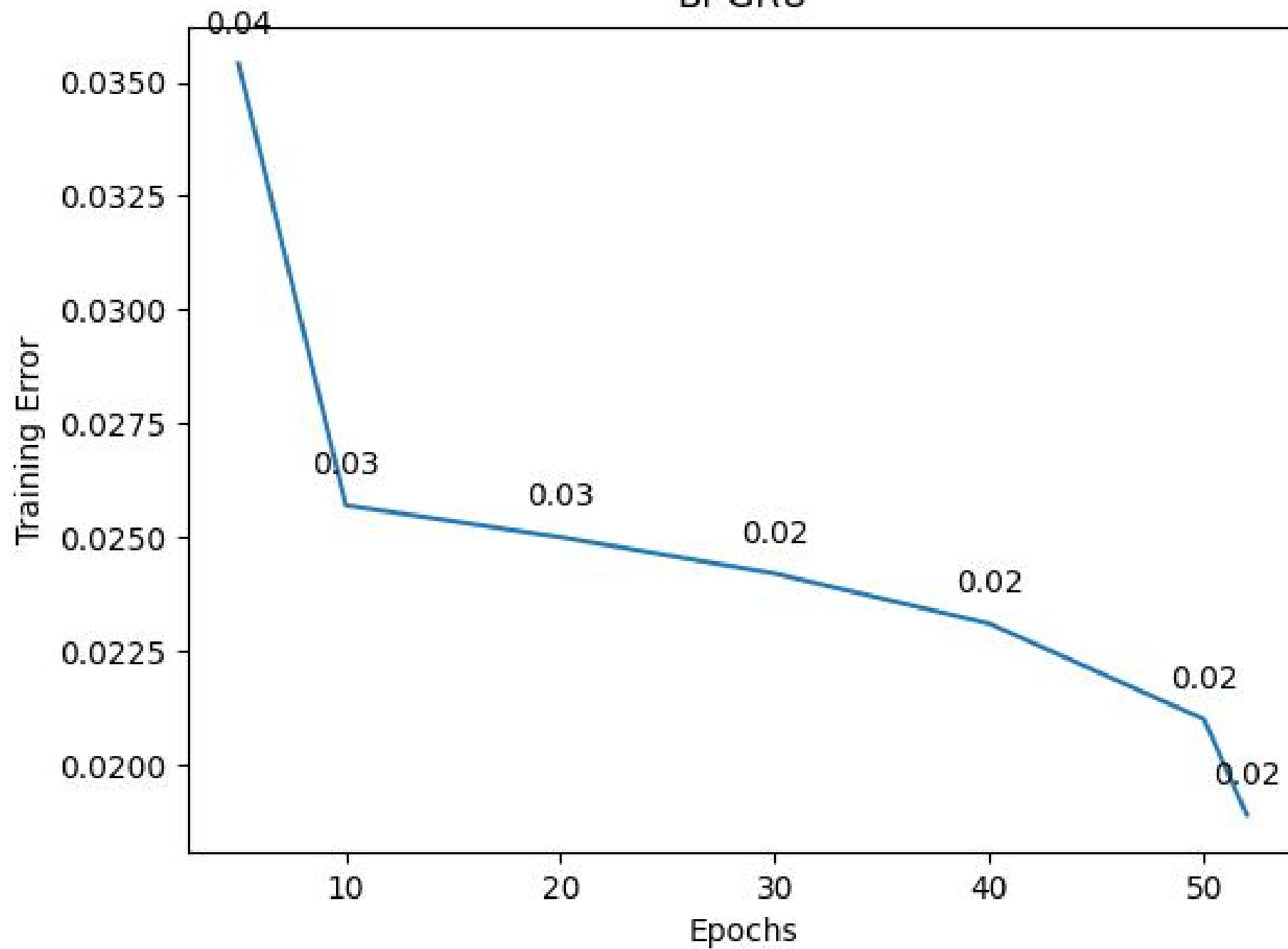

Supplement: Supplementary Materials — (e.g., datasets or results outcomes in the form of graphs) from different stages are provided with the manuscript. The graphs including system training, validation, and the testing outcome of all RNN variants are included in Supplementary Materials. [file 8415333.f1.zip › 8415333.f1/tbigru.pdf]

Bi-LSTM

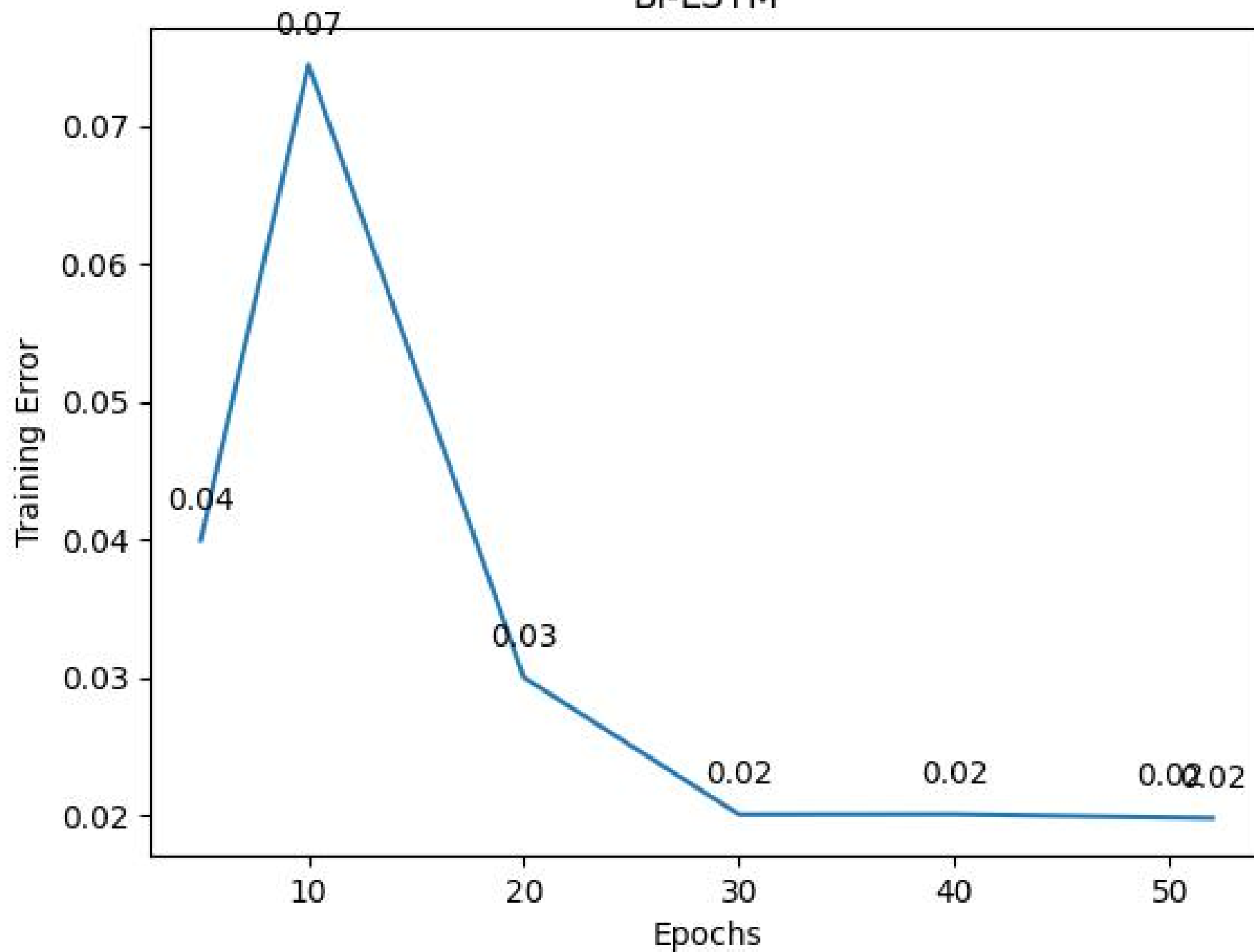

Supplement: Supplementary Materials — (e.g., datasets or results outcomes in the form of graphs) from different stages are provided with the manuscript. The graphs including system training, validation, and the testing outcome of all RNN variants are included in Supplementary Materials. [file 8415333.f1.zip › 8415333.f1/tbilstm.pdf]

## GRU

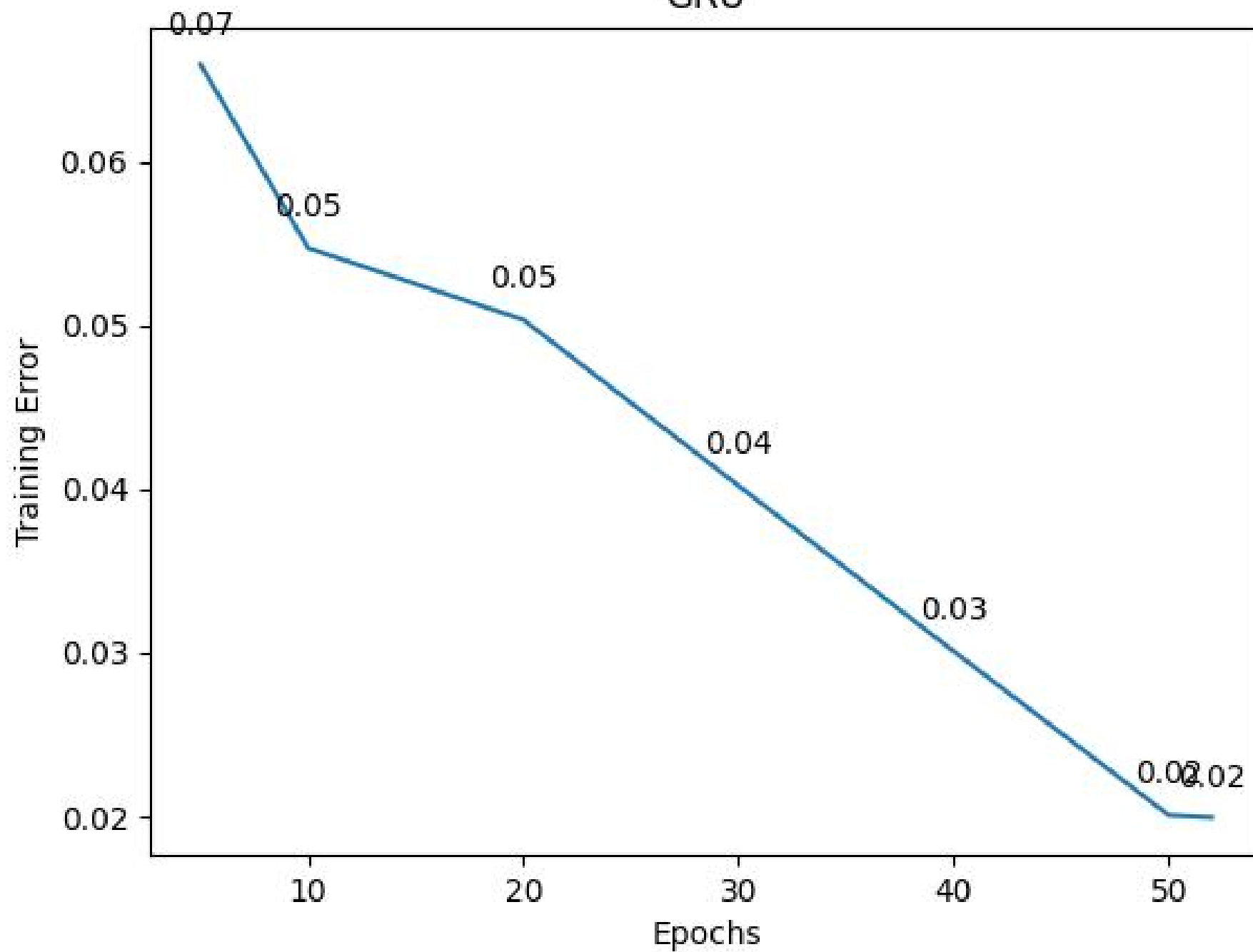

Supplement: Supplementary Materials — (e.g., datasets or results outcomes in the form of graphs) from different stages are provided with the manuscript. The graphs including system training, validation, and the testing outcome of all RNN variants are included in Supplementary Materials. [file 8415333.f1.zip › 8415333.f1/tgru.pdf]

## LSTM

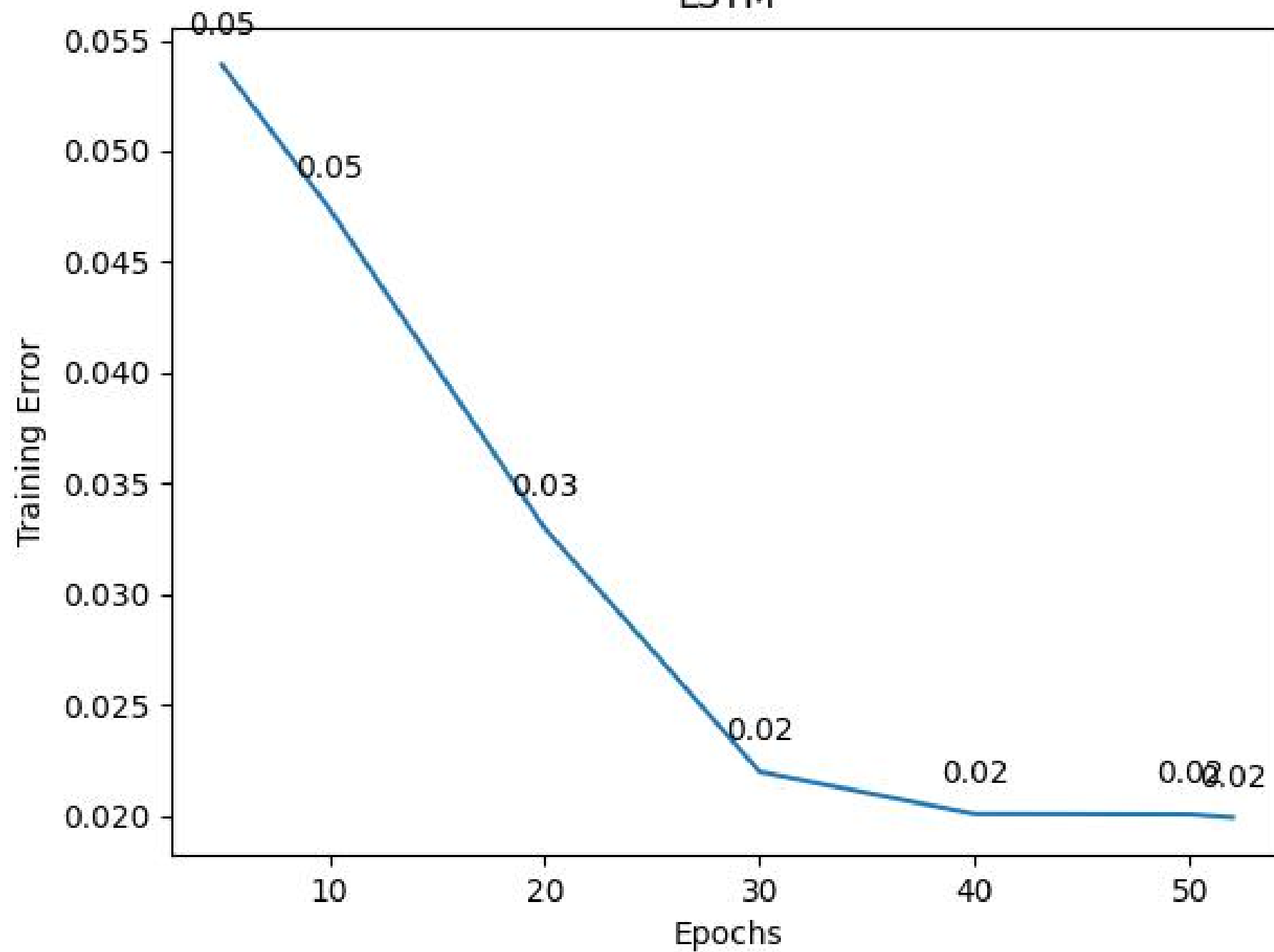

Supplement: Supplementary Materials — (e.g., datasets or results outcomes in the form of graphs) from different stages are provided with the manuscript. The graphs including system training, validation, and the testing outcome of all RNN variants are included in Supplementary Materials. [file 8415333.f1.zip › 8415333.f1/tlstm.pdf]
